# Site-Selective, Photocatalytic Vinylogous Amidation of Enones

**DOI:** 10.1021/acs.orglett.2c03161

**Published:** 2022-11-03

**Authors:** Kitti Franciska Szabó, Katarzyna Goliszewska, Jakub Szurmak, Katarzyna Rybicka-Jasińska, Dorota Gryko

**Affiliations:** Institute of Organic Chemistry, Polish Academy of Sciences, Kasprzaka 44/52, 01-224 Warsaw, Poland

## Abstract

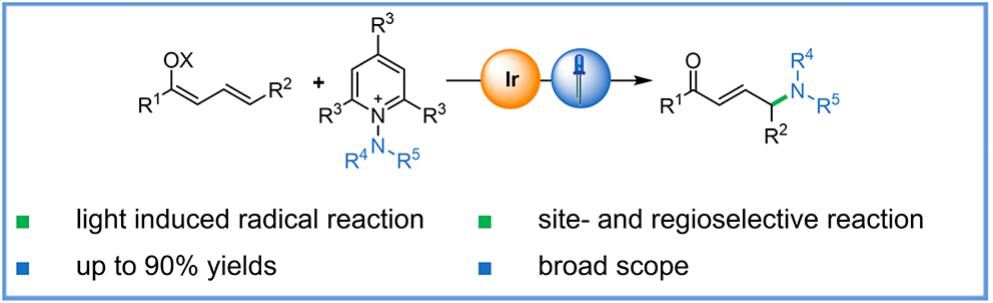

Despite the broad interest in organic compounds possessing a γ-aminocarbonyl motif, limited strategies for their synthesis have been reported. Herein, we describe a mild and efficient method for the site-selective amidation of unsaturated enones with electrophilic N-centered radicals as a key intermediate. The photocatalytic vinylogous reaction of dienolates with *N*-amino pyridinium salts affords γ-amido carbonyl compounds. This process is high-yielding, scalable, and tolerates a broad range of unsaturated α,β-unsaturated carbonyls, including biologically relevant compounds, as starting materials.

The concept of vinylogy, established by Fuson in 1935,^1^ postulates that the influence of a functional group can be propagated through a conjugated system of unsaturated bonds. This phenomenon is particularly important for the functionalization of α,β-unsaturated carbonyl compounds, which are versatile starting materials in organic synthesis.^2−15^ Typically, in vinylogous reactions, π-extended carbonyl derivatives of type **I** are transformed into dienolates **II** that contain two nucleophilic sites (Scheme 1). Consequently, the addition of electrophiles can occur at either α-position (**III**) or more remote γ-position (**IV**).^1,7^ The regio- and stereoselectivity of these transformations are affected by multiple factors, such as the presence of bulky substituents, a catalyst (if any), or the electron density at the nucleophilic carbon sites, and remain one of the most challenging issues that have to be addressed.^1−3,7−13^

**Scheme 1:**
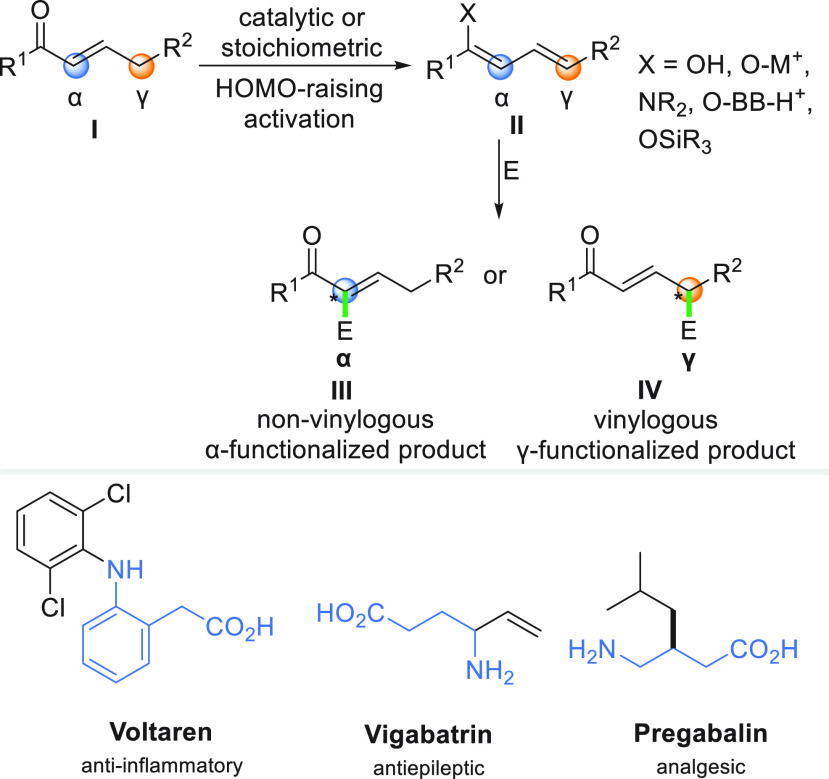
Concept of Vinylogy and Bioactive Molecules Containing a γ-Amino Group^16−18^

In recent years, in addition to the established use of preformed silyl enol ethers, novel activation strategies have been developed for vinylogous transformations.^19−25^ These include iminium/enamine organocatalysis,^19,20,22,26−28^ NHC organocatalysis,^23,24,26^ cooperative organo/metal catalysis,^10,25^ and photocatalysis.^29,30^ Because the application of vinylogy creates an additional reaction site in enolizable π-extended carbonyl systems, it has been widely utilized in the synthesis of distantly substituted carbonyl derivatives.^8,15,31−33^ Among them, γ-amination occupies a particular position as γ-aminocarbonyl motifs are quite ubiquitous in natural compounds, γ-aminobutyric acid (GABA), and bioactive molecules (Scheme 1).^16,34,35^ Currently, the known methods for vinylogous amination mainly utilize tetraazodicarboxylates as a nitrogen source and are often limited in scope. Jørgensen et al. first introduced an organocatalytic approach for the enantioselective γ-amination of dienamines via [4+2] cycloaddition to azodicarboxylates.^19^ Alternatively, dienolates were found to react site-selectively with the same electrophile in the presence of a base.^16^

Significant advances have been made in the field of photoredox catalysis, and a great deal of effort has been spent on expanding the utility of radicals in organic synthesis.^36−41^ In vinylogous transformations, substrates that bear a leaving group at the functionalized position have been mainly utilized.^29,30^ However, despite the broad application of nitrogen-centered radicals in synthetic chemistry,^42−46^ their reactivity in vinylogous reactions has rarely been explored.^44,46−49^ We have recently reported that electrophilic nitrogen-centered radicals generated from *N*-aminopyridinium salts are trapped by enol equivalents to give α-amido carbonyl compounds in excellent yields.^50^ On the basis of the vinylogy principle, we hypothesized that photocatalytic amidation at the γ-position of the enone system with electrophilic amidyl radicals should also be feasible.

Herein, we present the first example of a photocatalytic, vinylogous amidation of extended enolate derivatives. Under visible-light irradiation, silyl dienol ethers react with pyridinium salts in a highly selective manner via a radical mechanism. Our novel procedure opens doors for the site-selective synthesis of various γ-amido-α,β-unsaturated carbonyl compounds.

We initiated our studies by exploring the reactivity of α,β-unsaturated carbonyl compounds under previously developed conditions for the α-amidation.^50^ The model reaction of silyl dienol ether **1a** with *N*-aminopyridinium salt **2a** in the presence of the *fac*-Ir(ppy)_3_ catalyst, under blue-light irradiation, site-selectively gave the desired γ-amidated product **3a** in 65% yield as the only product (Table 1, entry 1). Background experiments confirmed that the desired transformation cannot take place without the Ir photocatalyst and a light source (entries 2–4). Subsequently, several reaction parameters [catalyst loading, substrate ratio, duration, and the power of the light (for details, see the Supporting Information)] were optimized. The yield substantially increased when the salt was used in a slight excess (1.3 equiv, entry 6); moreover, the reaction time was decreased to 1 h.

**Table 1:**
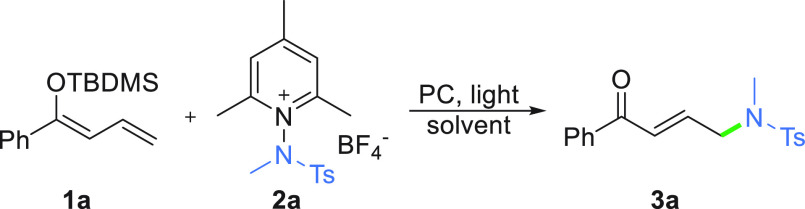
Optimization of the Reaction Conditions^a^

entry	catalyst	catalyst loading (mol %)	light	yield (%)^b^
1^c^	*fac*-Ir(ppy)_3_	1.0	blue	65
2	none	none	blue	trace
3	*fac*-Ir(ppy)_3_	1.0	none	trace
4	none	none	none	0
5	*fac*-Ir(ppy)_3_	1.0	blue	84
6^d^	*fac*-Ir(ppy)_3_	1.0	blue	90

a Reaction conditions: enol **1a** (0.25 mmol), salt **2a** (1.2 equiv), dry MeCN (*c* = 0.05 M), ambient temperature (20–22 °C), 1 h, under an Ar atmosphere, LED light source (446 nm, 6 W). TBDMS = *tert*-butyldimethylsilyl.

b Isolated yield.

c Reaction mixture irradiated for 16 h.

d Salt **2a** (1.3 equiv).

Gratifyingly, decreasing the catalyst loading to 0.75 mol % did not decrease the yield. Overall, irradiation of a solution of **1a** with **2a** (1:1.3 molar ratio) and *fac*-Ir(ppy)_3_ (0.75 mol %) with blue LEDs at room temperature for 1 h gives the *E*-isomer as sole product **3a** in 90% yield.

With the optimized conditions in hand, we examined a set of *N*-aminopyridinium salts and various α,β-unsaturated compounds. Silyl dienol ether **1a** tolerates both *N*-mono- and *N,N*-disubstituted *N*-aminopyridinium salts **2**, giving the desired products in good to high yields [**3a–3f** (Table 2)]. Among N,N-disubstituted derivatives **2a–2d**, similarly to α-amidation reactions,^50^ the most efficient salt **2a** with *N*-Me, *N*-Ts functionality gives the desired product in 90% yield in a site-selective manner, and only the *E*-alkene forms (entry 1). The stereoselectivity of the reaction is, however, affected by the substituents at the amidyl radical. For salts **2b** and **2d** (entries 1 and 4, respectively) with a bulky Boc protecting group, high yields are observed, but a mixture of diastereoisomeric *E*/*Z* dienes (∼6:5 *E*:*Z*) was isolated (entries 2 and 4). With Cbz salt **2c**, the reaction is again fully site- and stereoselective (entries 3 and 5).

**Table 2:**
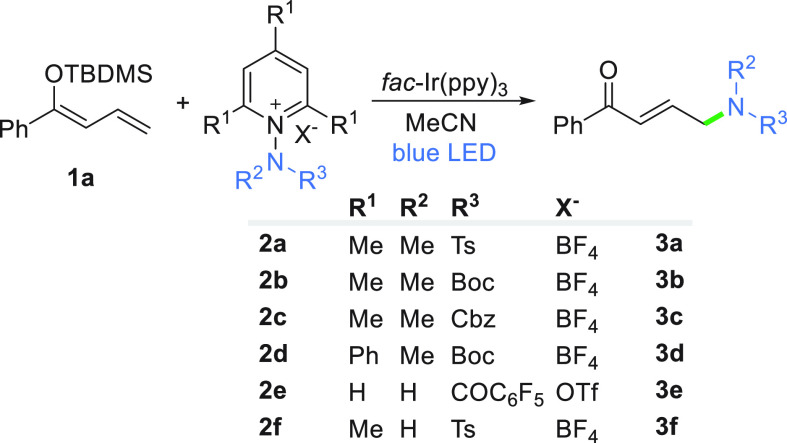
Scope of *N*-Aminopyridinium Salts^a^

entry	salt	*E*:*Z*	product	yield (%)
1	**2a**	*E*	**3a**	90
2	**2b**	6:5	**3b**	76
3	**2c**	*E*	**3c**	46
4	**2d**	6:5	**3d**	74
5	**2e**	*E*	**3e**	48
6	**2f**	*E*	**3f**	74

a Reaction conditions: enol **1a** (0.25 mmol), salt **2a–2f** (1.3 equiv), dry MeCN (*c* = 0.05 M), ambient temperature (20–22 °C), 1 h, under an Ar atmosphere, LED light source (446 nm, 6 W). Times: 1 h for **2a**, **2b**, **2d**, and **2f**; 2 h for **2c**; and 16 h for **2e**.

Various vinylogous substrates are well tolerated (Scheme 2). Aryl-substituted enones with various functional groups with both electron-withdrawing (CN, NO_2_, COMe, and halides) and electron-donating (*tert*-butyl and OMe) groups at the *para* and *meta* positions give products **4–11** in good to excellent yields (60–90%). Principally, the use of silyl enol ether derivatives preferentially generates the γ-product over the α-product due to higher orbital coefficients and higher electrophilic susceptibility.^51^ Furthermore, diphenylbuta-1,3-diene acetate and benzoate exclusively furnish γ-amidated products **12a** and **12b**, respectively, in a similar high yield. Interestingly, in the 1,4-diaryl α,β-unsaturated carbonyl compound series, the α,γ-siteselectivity of the amidation is strongly influenced by the electronic character of the phenyl ring present at the terminal double bond, while the nature of the chalcone phenyl substituent does not have an impact on the process. In particular, having the electron-donating methoxy group at the *para* (**13a**), *meta* (**13b**), or *ortho* (**13c**) position on both phenyl substituents does not alter the reaction outcome, and the desired γ-amidated products form site-selectively. Similarly, substrates with both electron-donating and electron-withdrawing substituents on the aryl rings give only the γ-product provided the methoxy group is in the R^2^ position (**13d**). On the contrary, compounds bearing a phenyl substituent with electron-withdrawing substituents (-CN or -CF_3_) at the *para* position undergo selective α-amidation using either acetyl- or TBDMS-protected dienol ether derivatives, giving product **14a** or **14b**, respectively, as single *Z*-diastereoisomers in moderate yields. However, when the nucleophilicity of the carbonyl group decreases, the diastereoselectivity of the α-amidation decreases. Product **14c** forms as a mixture of *Z/E* diastereoisomers (12:1).

**Scheme 2:**
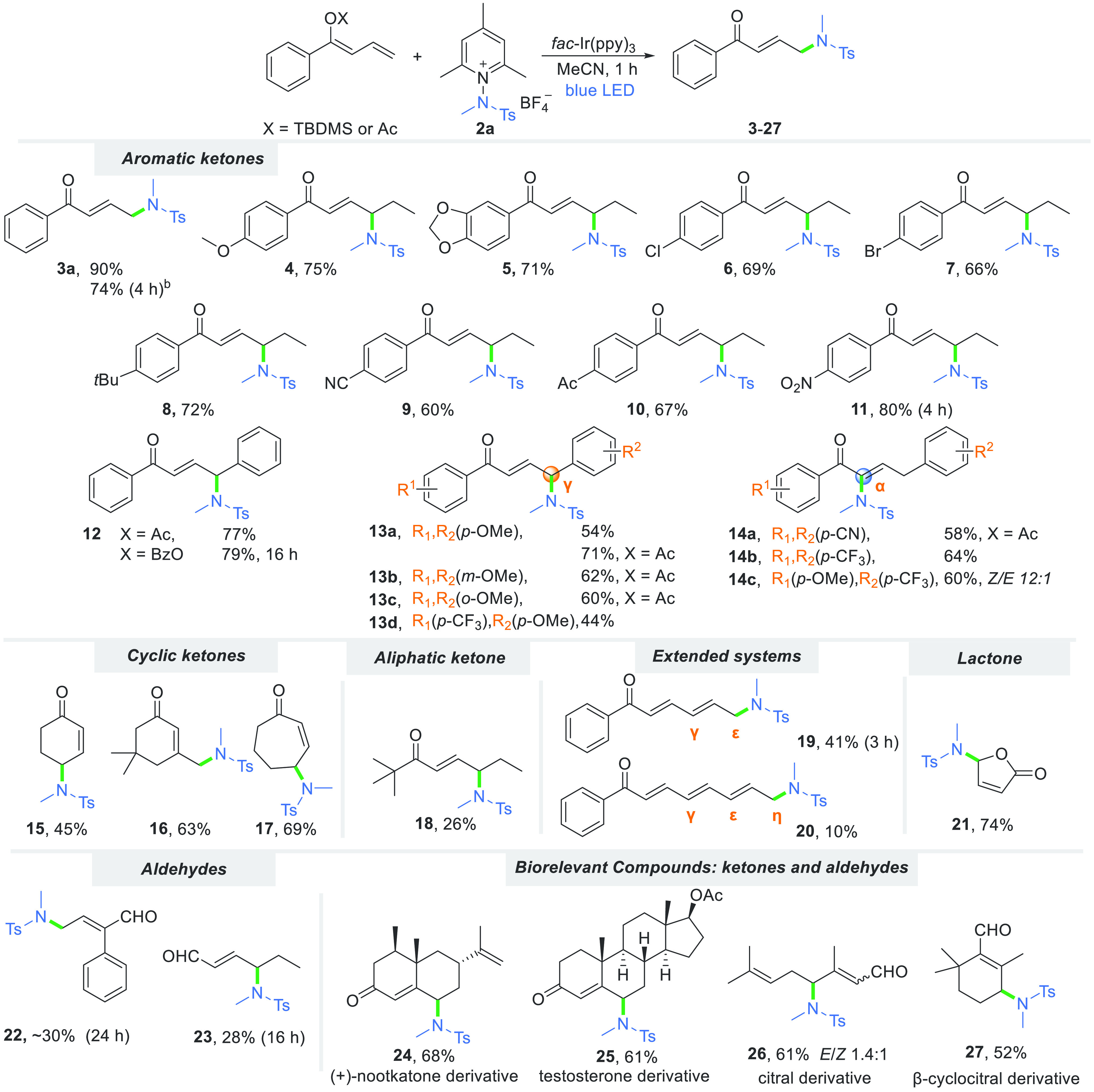
Scope of the amidation of α,β-Unsaturated Carbonyl Compounds Reaction conditions: enol **1a** (0.25 mmol), salt **2a** (1.3 equiv), dry MeCN (*c* = 0.05 M), ambient temperature (20–22 °C), 1 h, under an Ar atmosphere, LED light source (446 nm, 6 W). Unless otherwise noted, X = TBDMS. Reaction performed on a 1 mmol scale.

Furthermore, enols derived from cyclic ketones afford products **15–17** in good yields. Although, in general, the steric hindrance should affect product generation, here this is not the case. For a sterically hindered cyclohexenone derivative, the yield increases in comparison to that of the parent cyclohexenone presumably due to the electron-donating effect imposed by the methyl groups present at the reactive sites (**16**). Increasing the ring size effectively increases the yield. The γ-amidation of aliphatic enones is less effective (**18**, 26%).

Our methodology can be employed for functionalizations of enones with elongated systems of double bonds. Both substrates are compatible with the reaction conditions, although yields for ε and η functionalizations (**19** and **20**, respectively) are lower, due to the lower electron density at these positions. Furthermore, lactones and aldehydes are also suitable starting materials; the latter ones prove, however, to be challenging, with products **22** and **23** forming in lower yields. On the contrary, ester derivatives proved challenging, due to the hydrolysis of the starting dienolate (for details, see the Supporting Information). The utility and effectiveness of the developed method in late-stage functionalization are demonstrated on biologically active compounds such as (+)-nootkatone (**24**), testosterone (**25**), citral (**26**), and β-citral (**27**). In contrast to simple aldehyde dienolates, citral and β-cyclocitral provide products in satisfactory yields, highlighting the robustness of the methodology. We emphasize that in all these cases only the γ-amidated product is obtained, although a mixture of *E*/*Z* dienolate silyl ethers was used as the starting material.

With regard to the mechanism, the addition of TEMPO stops the reaction, thus confirming the radical nature of the reaction. Employing DMPO as a spin trap for N-centered radicals leads to the trapping product as HR-MS confirms (see Figure S3). These results clearly indicate that the developed reaction is radical in nature. Data from the literature,^50,52^ along with the results of control experiments, allow us to propose a plausible light-induced radical reaction pathway for the γ-amidation that is similar to that reported for α-amidation (Scheme 3). The reduction of *N*-aminopyridinium salt **2a** (*E*_1/2_ = −0.70 V vs Ag/AgCl) by Ir(III) in the excited state generates radical **A** via single-electron transfer (SET). Thus, the formed species, **A**, undergoes fragmentation to afford N-centered radical **B** and pyridine as a byproduct. The addition of N-centered radical **B** to dienolate **1a** generates allylic radical **C**, which is oxidized by the Ir(IV) catalyst to allylic cation **D** with the regeneration of the ground state of the Ir(III) catalyst. Removal of the acyl or silyl group affords γ-product **3a**.

**Scheme 3:**
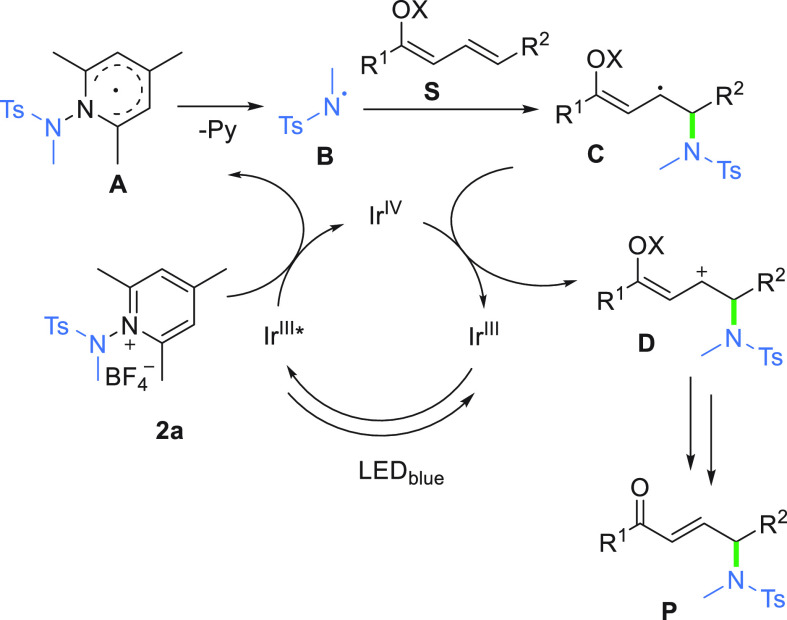
Mechanistic Proposal for the γ-Reactivity of Vinylogous Ketone with *N*-Aminopyridinium Salt

In conclusion, on the basis of the vinylogy principle, we have developed a method for the site-selective amidation of α,β-unsaturated enones with *N*-protected aminopyridinium salts giving access to γ-amidocarbonyl compounds. The reaction of N-centered radical, generated via Ir photocatalysis, with a dienolate intermediate is the key step in this transformation. The advantages of this approach include mild reaction conditions, high site- and stereoselectivity and substrate tolerance, a simple setup, and scalability. In addition, it is suitable for functionalizations of biologically active derivatives.

We believe that the vinylogy strategy may find applications in the design of other radical transformations of α,β-unsaturated compounds.

## Supporting Information Available

Optimization details, experimental procedures, and characterization data for all new compounds (PDF)
