# Supplementary material for: Site-Selective, Photocatalytic Vinylogous Amidation of Enones
Source: Org Lett. 2022 Nov 3;24(44):8120–4. doi: 10.1021/acs.orglett.2c03161 (PMC9664488; doi:10.1021/acs.orglett.2c03161)

Supporting Information

Site-selective, photocatalytic vinylogous amidation of enones

Kitti Franciska Szabó, Katarzyna Goliszewska, Jakub Szurmak, Katarzyna Rybicka-Jasińska*, Dorota Gryko*

*Institute of Organic Chemistry **P**olish Academy of Sciences*

Kasprzaka 44/52, 01-224 Warszaw, Poland

e-mail: dorota.gryko@icho.edu.pl

Table of Contents

1. **General Information**……………………………………………………..………..S6
2. **General synthetic procedure**……………………………………………..…….S8
3. **Optimization details**……………………………………………………...………..S11
4. **Mechanistic considerations** ………………………………………...…………..S13
5. **Scope and limitations, characterization of new compounds**………….……..S15
6. **References** …………………………………………………………..………..S70
7. **NMR spectra**………………………………………………………...…..S71

2*E*,4*E*,6*E*)-1-phenylocta-2,4,6-trien-1-one (**E3**)………………………………….....S71

(*E*)-1-(4-methoxyphenyl)hex-2-en-1-one (**E4**)…………………………………...…S71

(*E*)-1-(benzo[*d*][1,3]dioxol-5-yl)hex-2-en-1-one (**E8**)..S72

(*E*)-4-(hex-2-enoyl)benzonitrile (**E11**)……………………………………………S72

(*E*)-2,2-dimethyloct-4-en-3-one (**E20**)……………………………………………S73

(1*Z*,3*E*)-1,4-bis(4-methoxyphenyl)buta-1,3-dien-1-yl acetate (**S15**)…………...…S73

(*Z*,3*E*)-1,4-bis(4-cyanophenyl)buta-1,3-dien-1-yl acetate (**S18**)…………………..S74

tert-butyl((5,5-dimethyl-3-methylenecyclohex-1-en-1-yl)oxy)

dimethylsilane (**S22**)…………………………………………………………S74

tert-butyl(cyclohepta-1,3-dien-1-yloxy)dimethylsilane (**S23**)……………………S75

(*E*)-1-(4-(tert-butyl)phenyl)hex-2-en-1-one (**E5**)………………………………………S76

(*E*)-1-(4-chlorophenyl)hex-2-en-1-one (**E6**)…………………………………………S77

(*E*)-1-(4-bromophenyl)hex-2-en-1-one (**E7**)……………………………………………S78

(*E*)-1-(4-nitrocyclohexa-1,3-dien-1-yl)hex-2-en-1-one (**E9**)..S79

(*E*)-1-(4-acetylphenyl)hex-2-en-1-one (**E10**)……………………………………...S80

1,4-bis(4-methoxyphenyl)but-2-en-1-one (*Z*/*E* mixture) (**E12**)………………………...S81

1-(4-methoxyphenyl)-4-(4-(trifluoromethyl)phenyl)but-2-en-1-one (*Z*/*E* mixture) (**E13**)..S82

(*E*)-1,4-bis(4-(trifluoromethyl)phenyl)but-2-en-1-one (**E14**)……………………...…...S83

(*E*)-4-(4-methoxyphenyl)-1-(4-(trifluoromethyl)phenyl)but-2-en-1-one (**E15**)………...…S84

tert-butyldimethyl((1-phenylbuta-1,3-dien-1-yl)oxy)silane (*Z*/*E* mixture) (**S1**)……..……S85

tert-butyldimethyl(((3*E*)-1-phenylhexa-1,3,5-trien-1-yl)oxy)silane (*Z*/*E* mixture) (**S2**).….S86

tert-butyldimethyl(((1*Z*,3*E*,5*E*)-1-phenylocta-1,3,5,7-tetraen-1-yl)oxy)silane (**S3**)…..……S88

tert-butyl(((1*Z*,3*E*)-1-(4-methoxyphenyl)hexa-1,3-dien-1-yl)oxy)dimethylsilane (**S4**)……S89

tert-butyl(((1*Z*,3*E*)-1-(4-(tert-butyl)phenyl)hexa-1,3-dien-1-yl)oxy)dimethylsilane (**S5**)….S90

tert-butyl(((1*Z*,3*E*)-1-(4-chlorophenyl)hexa-1,3-dien-1-yl)oxy)dimethylsilane (**S6**)………S91

(((1*Z*,3*E*)-1-(4-bromophenyl)hexa-1,3-dien-1-yl)oxy)(tert-butyl)dimethylsilane (**S7**)…….S92

(((1*Z*,3*E*)-1-(benzo[d][1,3]dioxol-5-yl)hexa-1,3-dien-1-yl)oxy)(tert-butyl)

dimethylsilane (**S8**)…………………………………………………………………S93

tert-butyldimethyl(((1*Z*,3*E*)-1-(4-nitrophenyl)hexa-1,3-dien-1-yl)oxy)silane (**S9**)………..S94

1-(4-((1*Z*,3*E*)-1-((*tert*-butyldimethylsilyl)oxy)hexa-1,3-dien-1-yl)phenyl)

ethan-1-one (**S10**)……………………………………………………………….…….S95

4-((1*Z*,3*E*)-1-((*tert*-butyldimethylsilyl)oxy)hexa-1,3-dien-1-yl)benzonitrile (**S11**)….…….S96

tert-butyl(((1*Z*,3*E*)-1-(4-methoxyphenyl)-4-(4-(trifluoromethyl)phenyl)buta-1,3-dien-1-yl)oxy)dimethylsilane (**S12**)………………………………………………………………..S97

(((1*Z*,3*E*)-1,4-bis(4-(trifluoromethyl)phenyl)buta-1,3-dien-1-yl)oxy)(tert-butyl)

dimethylsilane (**S13**)……………………………………………………………………..S98

tert-butyl(((1*Z*,3*E*)-4-(4-methoxyphenyl)-1-(4-(trifluoromethyl)phenyl)buta-1,3-dien-1-yl)oxy)dimethylsilane (*Z*/*E* mixture) (**S14**)…………………………………………………S99

(1*Z*,3*E*)-1,4-bis(3-methoxyphenyl)buta-1,3-dien-1-yl acetate (**S16**)……………………...S100

(1*Z*,3*E*)-1,4-bis(2-methoxyphenyl)buta-1,3-dien-1-yl acetate (**S17**)……………………...S101

tert-butyl(((3*Z*,5*E*)-2,2-dimethylocta-3,5-dien-3-yl)oxy)dimethylsilane (**S25**)…………...S102

tert-butyl(((4*R*,4a*S*,6*R*)-4,4a-dimethyl-6-(prop-1-en-2-yl)-3,4,4a,5,6,7-hexahydronaphthalen-2-yl)oxy)dimethylsilane (**S26**)…………………………………..…S103

(1*R*,8*R*,9*S*,13*S*,14*S*,17*S*)-3-((*tert*-butyldimethylsilyl)oxy)-1,13-dimethyl-2,7,8,9,10,11,12,13,14,15,16,17-dodecahydro-1*H*-cyclopenta[a]phenanthren-17-yl acetate (**S27**)……………………………………………………………………..…..S104

tert-butyl((3*E*)-hexa-1,3-dien-1-yloxy)dimethylsilane (*Z*/*E* mixture) (**S28**)…………….....S105

tert-butyl(((1*Z*,3*E*)-3,7-dimethylocta-1,3,6-trien-1-yl)oxy)dimethylsilane (**S29**)………....S106

(*E*)-tert-butyldimethyl((2,6,6-trimethylcyclohex-2-en-1-ylidene)methoxy)silane (**S30**)….S107

(*E*)-*tert*-butyldimethyl(2-phenylbuta-1,3-dien-1yl)oxy)silane (**S31**)………………...……S108

(*E*)-*N*,4-dimethyl-*N*-(4-oxo-4-phenylbut-2-en-1-yl)benzenesulfonamide (**3a**)……..…......S109
tert-butyl methyl(4-oxo-4-phenylbut-2-en-1-yl)carbamate (*E*/*Z* mixture) (**3b**) …......…...S110
(*E*)-4-methyl-*N*-(4-oxo-4-phenylbut-2-en-1-yl)benzenesulfonamide (**3f**)..........................S111
(*E*)-2,3,4,5,6-pentafluoro-*N*-(4-oxo-4-phenylbut-2-en-1-yl)benzamide (**3e**).......................S112
benzyl (*E*)-methyl(4-oxo-4-phenylbut-2-en-1-yl)carbamate (**3c**)……………………........S113
N,4-dimethyl-*N*-((2*E*,4*E*)-6oxo-6-phenylhexa-2,4-dien-1-yl)benzenesulfonamide (**19**)......S114
N,4-dimethyl-*N*-((2*E*,4*E*,6*E*)-8-oxo-8-phenylocta-2,4,6-trien-1-yl)
benzenesulfonamide (**20**)………………………………………………………......S115
(*E*)-*N*-(6-(4-methoxyphenyl)-6-oxohex-4-en-3-yl)-*N*,4-
dimethylbenzenesulfonamide (**4**)…………………………...………………........S116
(*E*)-*N*-(6-(4-(*tert*-butyl)phenyl)-6-oxohex-4-en-3-yl)-*N*,4-
dimethylbenzenesulfonamide (**8**)………………………………………………...…S117
(*E*)-*N*-(6-(4-chlorophenyl)-6-oxohex-4-en-3-yl)-*N*,4-
dimethylbenzenesulfonamide (**6**)……..……………………………………………S118
(*E*)-*N*-(6-(4-bromophenyl)-6-oxohex-4-en-3-yl)-*N*,4-
dimethylbenzenesulfonamide (**7**)…………………….…………………….………S119
(*E*)-*N*-(6-(benzo[*d*][1,3]dioxol-5-yl)-6-oxohex-4-en-3-yl)-*N*,4-
dimethylbenzenesulfonamide (**5**)….…………………………………………………S120
(*E*)-*N*,4-dimethyl-*N*-(6-(4-nitrophenyl)-6-oxohex-4-en-3-yl)benzenesulfonamide (**11**)…..S121
(*E*)-*N*-(6-(4-acetylphenyl)-6-oxohex-4-en-3-yl)-*N*,4-
dimethylbenzenesulfonamide (**10**)……………………………………………………S122
(*E*)-*N*-(6-(4-cyanophenyl)-6-oxohex-4-en-3-yl)-*N*,4-
dimethylbenzenesulfonamide (**9**).……………………………………………………S123
(*E*)-*N*-(1,4-bis(4-methoxyphenyl)-4-oxobut-2-en-1-yl)-*N*,4-
dimethylbenzenesulfonamide (**13a**)….………………………………………………S124
(*Z*)-*N*-(1-(4-methoxyphenyl)-1-oxo-4-(4-(trifluoromethyl)phenyl)but-2-en-2-yl)-*N*,4-
dimethylbenzenesulfonamide (**14c**)…………………………………….……………S125
(*Z*)-*N*,4-dimethyl-*N*-(1-oxo-1,4-bis(4-(trifluoromethyl)phenyl)but-2-en-2-
yl)benzenesulfonamide (**14b**)……………………………………………….……...S127
(*Z*)-*N*-(1-(4-methoxyphenyl)-4-oxo-4-(4-(trifluoromethyl)phenyl)but-2-en-1-yl)-*N*,4-
dimethylbenzenesulfonamide (**13d**)…………………………………………………..S128
(*E*)-*N*-(1,4-bis(3-methoxyphenyl)-4-oxobut-2-en-1-yl)-*N*,4-
dimethylbenzenesulfonamide (**13b**)…………………………………………….….S129
(*Z*)-*N*-(1,4-bis(2-methoxyphenyl)-4-oxobut-2-en-1-yl)-*N*,4-

dimethylbenzenesulfonamide (**13c**)…………………………………………………….S130

(*Z*)-*N*-(1,4-bis(4-cyanophenyl)-1-oxobut-2-en-2-yl)-*N*,4-

dimethylbenzenesulfonamide (**14a**)…………………………………………………….S131

(*E*)-*N*,4-dimethyl-*N*-(4-oxo-1,4-diphenylbut-2-en-1-yl)benzenesulfonamide (**12**)......…S132

N,4-dimethyl-*N*-(4-oxocyclohex-2-en-1-yl)benzenesulfonamide (**15**)…………...……S133

N-((5,5-dimethyl-3-oxocyclohex-1-en-1-yl)methyl)-*N*,4-

dimethylbenzenesulfonamide (**16**)…………………………………………...…....S134

N,4-dimethyl-*N*-(4-oxocyclohept-2-en-1-yl)benzenesulfonamide (**17**)..........................S135

N,4-dimethyl-*N*-(5-oxo-2,5-dihydrofuran-2-yl)benzenesulfonamide (**21**)......................S136

N-((3*S*,4a*S*,5*R*)-4a,5-dimethyl-7-oxo-3-(prop-1-en-2-yl)-1,2,3,4,4a,5,6,7-

octahydronaphthalen-1-yl)-*N*,4-dimethylbenzenesulfonamide (**24**)………………..…S137

(8*R*,9*S*,10*R*,13*S*,14*S*,17*S*)-6-((*N*,4-dimethylphenyl)sulfonamido)-10,13-dimethyl-3-oxo-2,3,6,7,8,9,10,11,12,13,14,15,16,17-tetradecahydro-1*H*-cyclopenta[a]phenanthren-17-yl acetate (**25**)……………………………………………………………..…………..S138

(*E*)-*N*-(7,7-dimethyl-6-oxooct-4-en-3-yl)-*N*,4-dimethylbenzenesulfonamide (**18**)…...…S139

(*E*)-*N*,4-dimethyl-*N*-(6-oxohex-4-en-3-yl)benzenesulfonamide (**23**)..............................S140

N-(3,7-dimethyl-1-oxoocta-2,6-dien-4-yl)-*N*,4-dimethylbenzenesulfonamide (**26**)..........S141

N-(3-formyl-2,4,4-trimethylcyclohex-2-en-1-yl)-*N*,4-

dimethylbenzenesulfonamide (**27**)..S142

N,4-dimethyl-*N*-(4-oxo-3-phenylbut-2-en-1-yl)benzenesulfonamide (**22**).......................S143

General information

All solvents and commercially available reagents were purchased as reagent grade and were used without further purification, unless otherwise stated. Yields refer to spectroscopically (^{1}H NMR) homogeneous materials. Reactions were monitored by thin layer chromatography (TLC), using 0.20 mm Merck silica plates (60F-254) or 0.20 mm Merck aluminum oxide plates (60F-254) and visualized using UV-light or cerium molybdate and ninhydrin stain with heat as a developing agent. Colum chromatography was performed using Merck silica gel 60 (230-400 mesh) or Merck Al_2O_3 neutral (50-300 mesh) deactivated with 15 wt% of H_2O.

- NMR spectra were recorded on Bruker 400 MHz, Varian 500 or 600 MHz and calibrated using residual undeuterated solvent ($CHCl_3$ – 7.26 ppm ^{1}H NMR, 77.16 ppm ^{13}C NMR) or TMS as an internal reference.
- High-resolution mass spectra (HRMS) were recorded on a Waters AutoSpec Premier instrument using electron ionization (EI) or a Waters SYNAPT G2-S HDMS instrument using electrospray ionization (ESI) with time-of-flight detector (TOF).
- Elemental analysis (C, H, N, S) were performed using a PERKIN-ELMER 240 Elemental Analyzer.
- Melting points were recorded on a Marienfeld MPM-H2 melting point apparatus and are uncorrected.
- Preparative HPLC separations were performed using Knauer HPLC chromatograph with PDA detector and Preparative column chromatography Knauer EII 100-10 Si column (250 x 20 mm).
- Commercial reagents were purchased from Sigma-Aldrich, Alfa Aesar, TCI and AmBeed, and used as received unless otherwise noted.

1.2 Setup for photoreactions with aluminum cooling block

Photo-induced reactions were performed using a bottom plate irradiated vials in a specially constructed photoreactor with cooling by tap water and LED plate connected to constant current (0.7 A) power supply (Figure S1). The LED plates are commercially available radiators (Fischer Electronic part no. SK 105 100 SA) with 6 epoxy-glued star-cased 3 W LEDs connected in series. Reactions were carried out under blue light irradiation on a single diode (LT-2855 royal blue, λmax: 446 nm, 7W), distance from the reaction vessel: 6 mm.

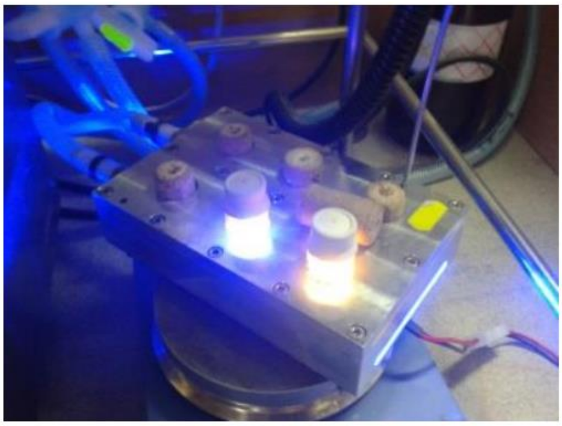

Figure S1.

1. General synthetic procedures

2.1. General synthetic procedure A - preparation of enol ethers

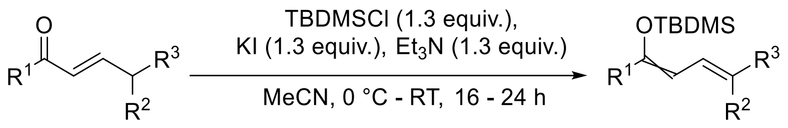

Scheme S1.

To a precooled to 0 °C solution of carbonyl compound (5.0 mmol, 1.0 equiv.) and KI (6.5 mmol, 1.3 equiv.) in anhydrous MeCN (0.5 M) under Ar atmosphere, Et_3N (6.5 mmol, 1.3 equiv) was added dropwise followed by the addition of TBDMSCl (6.5 mmol, 1.3 equiv. in one portion). The mixture was stirred for 16-24 h (determined by TLC) at room temperature. The reaction was quenched with $NaCl_{(sat.)}$ and then extracted with Et_2O. The organic solution was dried over Na_2SO_4, filtered, and concentrated in vacuo. The crude product was passed through deactivated the Al_2O_3 (neutral Al_2O_3 treated with 15 wt% of H_2O) plug using pentane as an eluent. The filtrate after evaporation was used for the next step without further purification.

2.2. General synthetic procedure B - preparation of enol ethers

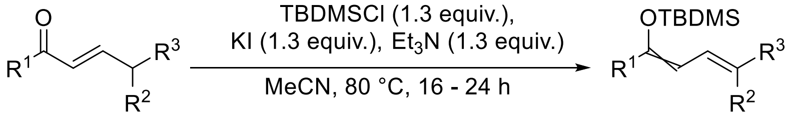

Scheme S2.

To a solution of carbonyl compound (5.0 mmol, 1.0 equiv.) and KI (6.5 mmol, 1.3 equiv.) in anhydrous MeCN (0.5 M) under Ar atmosphere, Et_3N (6.5 mmol, 1.3 equiv) was added dropwise followed by the addition of TBDMSCl (6.5 mmol, 1.3 equiv. in one portion). The reaction mixture was heated to 80 °C using a heating mantle and was stirred for 16-24 h (determined by TLC). The reaction was quenched with $NaCl_{(sat.)}$ and then extracted with Et_2O. The organic solution was dried over Na_2SO_4, filtered, and concentrated in vacuo. The crude product was passed through deactivated the Al_2O_3 (neutral Al_2O_3 treated with 15 wt% of H_2O) plug using pentane as an eluent. The filtrate after evaporation was used for the next step without further purification.

2.3. General synthetic procedure C - visible-light mediated amidation of enols

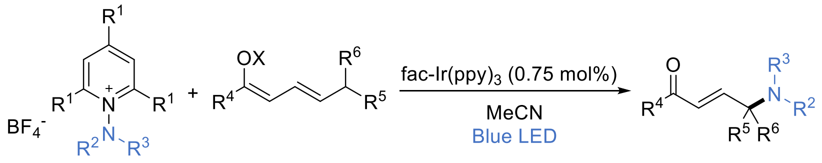

Scheme S3.

A glass vial equipped with a stirring bar and sealed with a septum was charged with an enol (if solid, 0.25 mmol, 1.0 equiv.), *fac*-Ir(ppy)$_3$ (0.01875 mmol, 0.75 mol%) and an *N*-aminopyridinium salt (0.33 mmol, 1.3 equiv.). Anhydrous MeCN (5 ml) was added, and the resulting mixture was degassed by argon bubbling for 20 minutes. Subsequently, (enol if liquid, 0.25 mmol, 1.0 equiv. was added) the reaction mixture was placed in a photoreactor and irradiated with blue LED for the time specified. Then, the mixture was transferred to a round-bottom flask and concentrated *in vacuo*. A crude product was purified by flash column chromatography using hexane/EtOAc mixture as an eluent.

2.4. General synthetic procedure D – preparation of enones

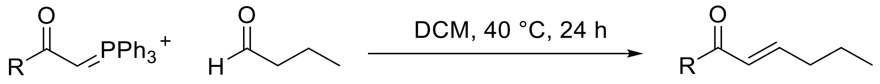

Scheme S4.

To a solution of phosphonium ylide (1.2 eq.) in dry DCM (15 ml) an aldehyde (1 eq.) was added dropwise. The mixture was refluxed (heating mantle as a heat source) until TLC showed full conversion (1-2 days). The solution was cooled, and the solvent was evaporated *in vacuo*. The product was purified by flash column chromatography using hexane, (pentane)/Et$_2$O mixture as eluent.

2.5 Procedure for 1 mmol-scale synthesis of (*E*)-N,4-dimethyl-*N*-(4-oxo-4-phenylbut-2-en-1-yl)benzenesulfonamide (P1)

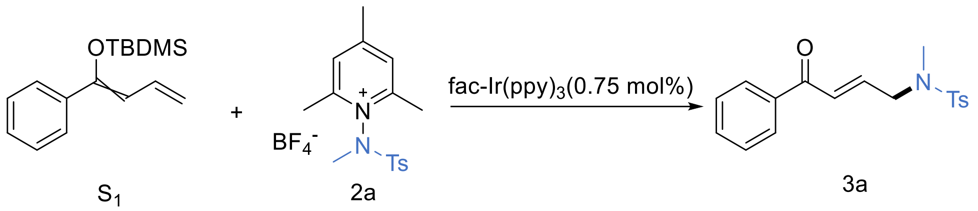

Scheme S5.

Following the general procedure **C** compound **3a** was obtained from tert-butyldimethyl((1-phenylbuta-1,3-dien-1-yl)oxy)silane (1 mmol) and 1-((*N*,4-dimethylphenyl)sulfonamido)-2,4,6-trimethylpyridinium tetrafluoroborate (1.3 mmol) (Figure S2) as yellow oil (245 mg) (**Yield = 74%**).

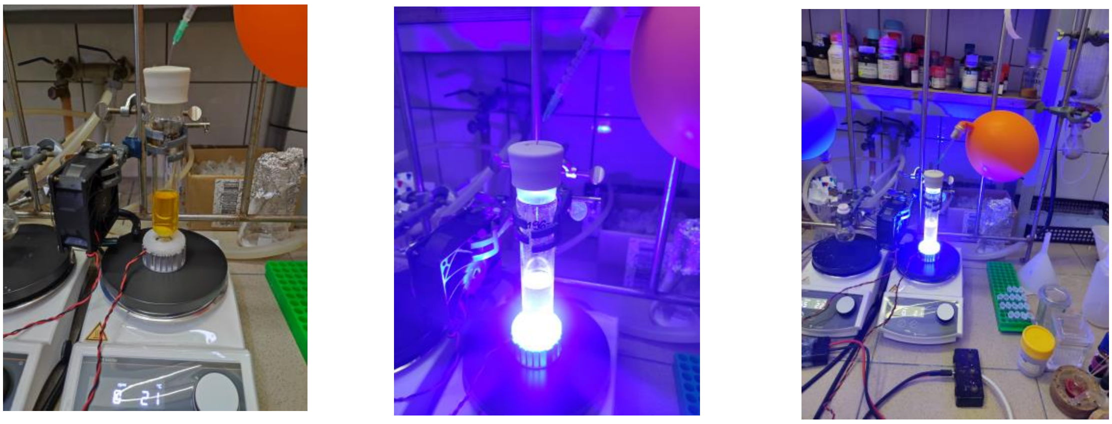

Figure S2.

2. Optimization details

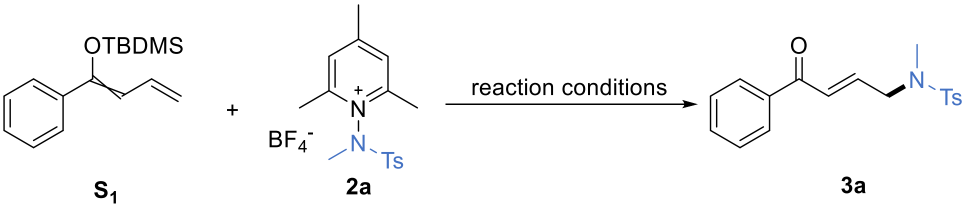

Scheme S5.

3.1 Table S1: Optimization of the substrate ratio[a]

Entry	Enol S1 [mmol]	Pyridinium salt 2a [mmol]	Ratio	Yield 3a[b] [%]
1	0.25	0.25	1:1	76
2	0.25	0.3	1:1.2	84
**3**	**0.325**	**0.25**	**1:1.3**	**90**
4	0.375	0.25	1:1.5	89

[a]Conditions: enol **S1** (0.25 mmol), salt **2a**, PC, MeCN (c = 0.05 M), ambient temperature (20-22 °C), 1 h under argon atmosphere, light source: blue LED diode (446 nm), [b]Isolated yield.

3.2 Table S2: Catalyst investigation and loading[a]

Entry	Catalyst	Catalyst loading [mol%]	Time [h]	Yield of 3a[b] [%]
1	$Ir(ppy)_3$	1	16	65
2	$Ir(ppy)_3$	1	1	84
3	$Ir(ppy)_3$	0.5	1	79
**4**	**$Ir(ppy)_3$**	**0.75**	**1**	**90**
5	$[Ir(dtbbpy)(ppy)_2PF_6$	1	1	20
6	$Ru(bpy)_3Cl_2 \cdot 6H_2O$	1	1	5

[a]Conditions: enol **S1** (0.25 mmol), salt **2a**, PC, MeCN (c = 0.05 M), ambient temperature (20-22 °C), under argon atmosphere, light source: blue LED diode (446 nm), [b]Isolated yield.

3.3 Table S3: Influence of the light power[a]

Entry	Light Power [W]	Time [h]	Yield of 3a[b]
1	3	1	81
**2**	**6**	**1**	**90**
3	6	16	65
4	10	1	84

[a]Conditions: enol **S1** (0.25 mmol), salt **2a**, PC, MeCN (*c* = 0.05 M), ambient temperature (20-22 °C), under argon atmosphere, light source: blue LED diode (446 nm), [b]Isolated yield.

3.4 Table S4: Optimization of the reaction time[a]

Entry	Time [h]	Yield of 3a[b]
**1**	**1**	**90**
2	16	65

[a]Conditions: enol **S1** (0.25 mmol), salt **2a**, PC, MeCN (*c* = 0.05 M), ambient temperature (20-22 °C), under argon atmosphere, light source: blue LED diode (446 nm), [b]Isolated yield.

3. Mechanistic considerations

4.1 Proposed mechanism

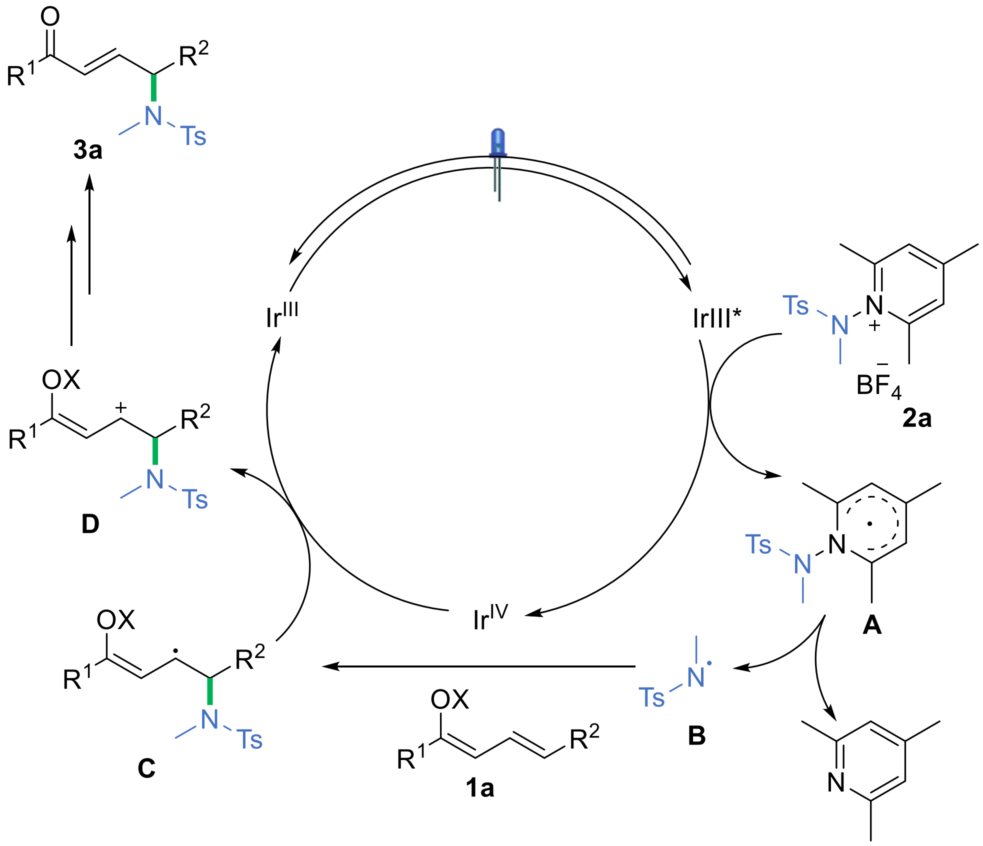

Scheme S6. Proposed reaction mechanism

4.2 Addition of 5,5-dimethyl-1-pyrroline *N*-oxide (DMPO)

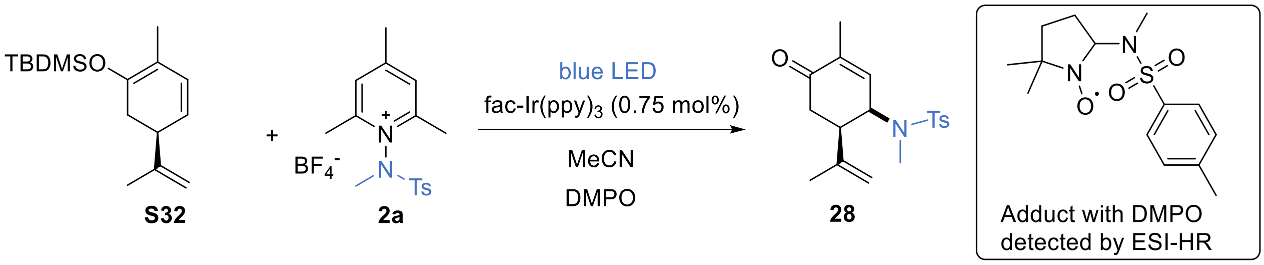

Scheme S7. Experiment with addition of DMPO[a]

[a]Reaction condition: Enol **S32** (0.25 mmol), salt **2a** (1.3 equiv.), $Ir(ppy)_3$ (0.75 mol%), DMPO (0.5 mmol), dry MeCN (c = 0.05 M), ambient temperature (20-22 °C), under Ar atmosphere was irradiated (blue LED, 446 nm)).

The assumed adduct formation with DMPO was confirmed by ESI-HRMS.

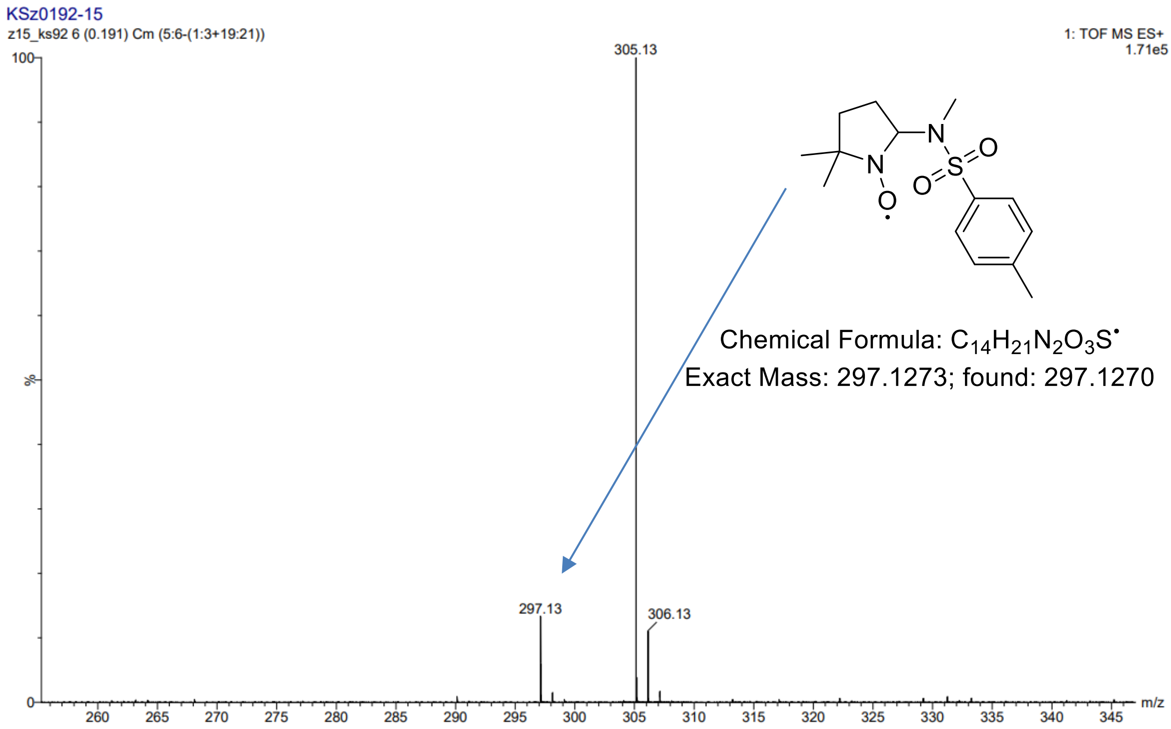

Figure S3. HRMS analysis of adduct form with DMPO

4.3 Addition of (2,2,6,6-tetramethylpiperidin-1-yl)oxyl (TEMPO)

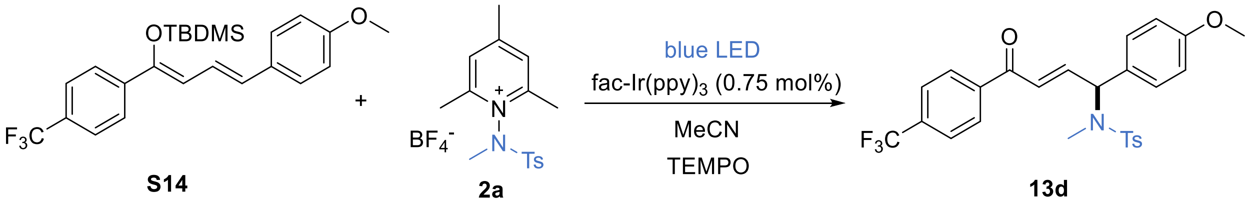

Scheme S8. Experiment with addition of TEMPO[a]

[a]Reaction condition: Enol **S14** (0.25 mmol), salt **2a** (1.3 equiv.), $Ir(ppy)_3$ (0.75 mol%), TEMPO (2 equiv.), dry MeCN (c = 0.05 M), ambient temperature (20-22 °C), under Ar atmosphere was irradiated (blue LED, 446 nm) for 1.5 hours. The reaction was checked by TLC- no product was observed - **reaction stopped**.

5. Scope and limitations, characterization of new compounds

5.1 Unsuccessful examples

ENTRY	SUBSTRATE	OUTCOME	PRODUCT
1	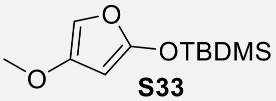 **S33**	**Hydrolysis to ketone**	-
2	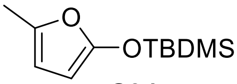 **S34**	**Hydrolysis to ketone**	-
3	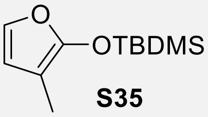 **S35**	**<5% of product**	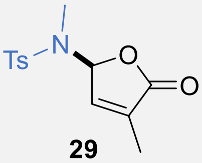 **29**
4	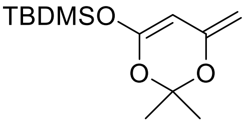 **S36**	**No conversion**	-
5	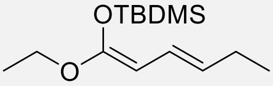 **S37**	**No conversion**	-
6	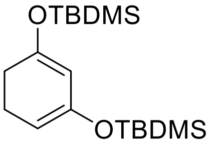 **S38**	**<1% of product**	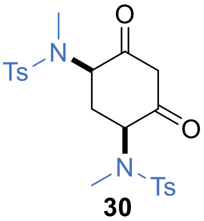 **30**
7	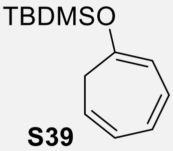 **S39**	**<10% of product**	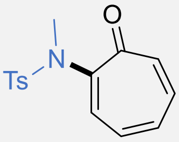 **31**
8	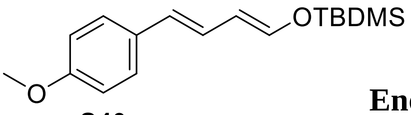 **S40**	**Enol degradation**	-

**9**	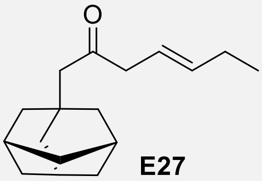 **E27**	**No conversion**	-
**10**	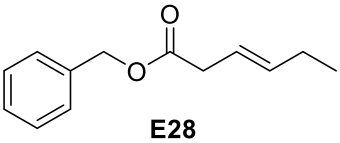 **E28**	**No conversion**	-
**11**	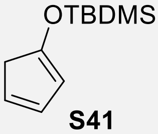 **S41**	**Enol degradation**	-
**12**	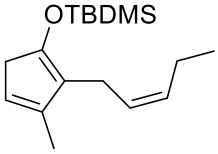 **S42**	**Enol degradation**	-
**13**	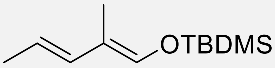 **S43**	**<10% of product**	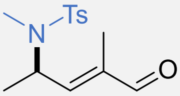 **32**
**16**	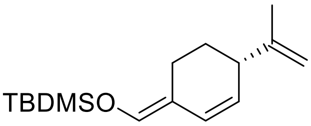 **S44**	**<10% of product**	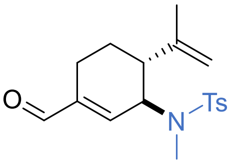 **33**
**17**	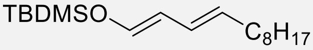 **S45**	**18%**	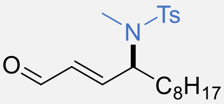 **34**

5.2 Enones

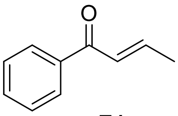
E1

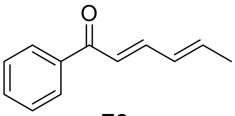
E2

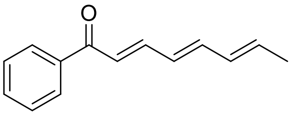
E3

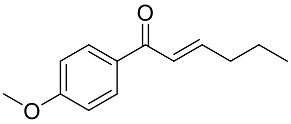
E4

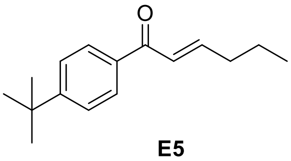
E5

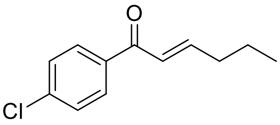
E6

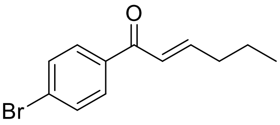
E7

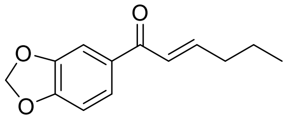
E8

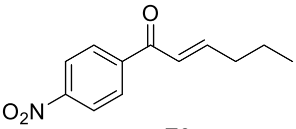
E9

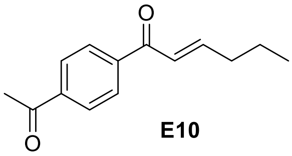
E10

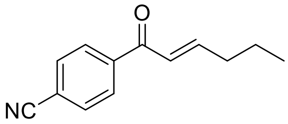
E11

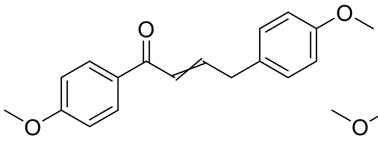
E12

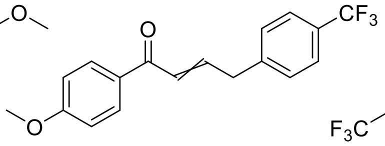
E13

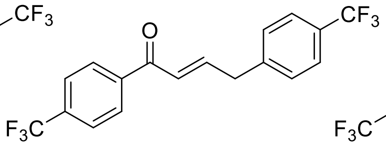
E14

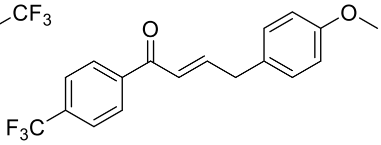
E15

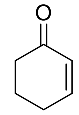
E16

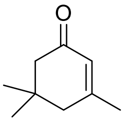
E17

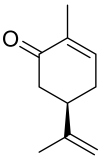
E18

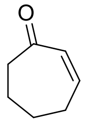
E19

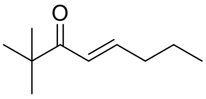
E20

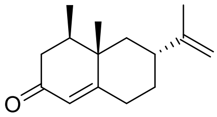
E21

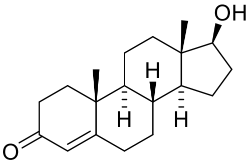
E22

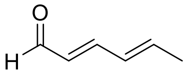
E23

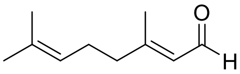
E24

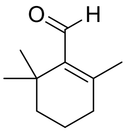
E25

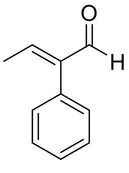
E26

Compounds **E3**, **E9**, **E12**, **E13**, **E14**, **E15** and **E20** were prepared according to general procedure **D**. Compounds **E4**[1], **E5**[1], **E6**[1], **E7**[1], **E8**[1], **E10**[1] and **E11**[1] were prepared according to literature procedure.

Compounds **E1**, **E2**, **E16**, **E17**, **E18**, **E19**, **E21**, **E22**, **E23, E24, E25** and **E26** are commercially available reagents and were used without further purification.

5.2.1 (2*E*,4*E*,6*E*)-1-phenylocta-2,4,6-trien-1-one (E3)

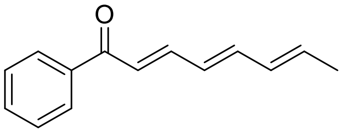

Following the general procedure **D** compound **E3** was obtained from 1-phenyl-2-(triphenyl-λ^5-phosphaneylidene)ethan-1-one (3.7 mmol) and (2*E*,4*E*)-hexa-2,4-dienal (18 mmol). The crude product was purified by column chromatography (5:95 AcOEt:Hex) to afford 0.37 g ketone (**E3**) as a white solid. (**Yield = 50%**).

<u>NMR data matched those reported in the literature</u>.[8]

^{1}H NMR (400 MHz, CDCl$_3$): δ 8.02 – 7.88 (m, 2H, Ph), 7.59 – 7.44 (m, 4H, COCH= and Ph), 6.96 – 5.74 (m, 5H, olefinic CH), 1.85 (d, *J* = 6.7 Hz, 3H, Me) ppm.

5.2.2 (*E*)-1-(4-methoxyphenyl)hex-2-en-1-one (E4)

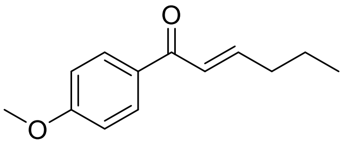

Following the literature procedure[1] compound **E4** was obtained from 1-(4-methoxyphenyl)ethan-1-one (7.5 mmol) and butyraldehyde (15 mmol). The crude product was purified by column chromatography (20:80 AcOEt:Hexane) to afford 0.23 g ketone (**E4**) as a colorless oil. (**Yield = 15% over 3 steps**).

<u>NMR data matched those reported in the literature</u>.[1]

^{1}H NMR (400 MHz, CDCl$_3$): δ 7.87 – 7.85 (m, 2H), 7.44 – 7.42 (m, 2H), 7.06 (dt, *J* = 15.3, 7.0 Hz, 1H), 6.82 (dt, *J* = 15.4, 1.4 Hz, 1H), 2.29 (qd, *J* = 7.3, 1.4 Hz, 2H), 1.56 (h, *J* = 7.4 Hz, 2H), 0.97 (t, *J* = 7.4 Hz, 3H) ppm.

5.2.3 (*E*)-1-(4-(*tert*-butyl)phenyl)hex-2-en-1-one (E5)

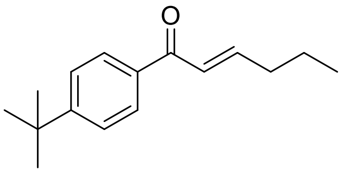

Following the literature procedure[1] compound **E5** was obtained from 1-(4-(tert-butyl)phenyl)ethan-1-one (7.5 mmol) and butyraldehyde (15 mmol). The crude product was purified by column chromatography (20:80 AcOEt:Hexane) to afford 0.95 g ketone (**E5**) as a yellow oil. (**Yield = 56% over 3 steps**).

^{1}H NMR (400 MHz, CDCl$_3$): δ 7.90 – 7.87 (m, 2H), 7.49 – 7.46 (m, 2H), 7.05 (dt, *J* = 15.4, 6.9 Hz, 1H), 6.88 (dt, *J* = 15.4, 1.4 Hz, 1H), 2.29 (qd, *J* = 7.2, 1.4 Hz, 2H), 1.56 (sext., *J* = 7.3 Hz, 2H), 1.35 (s, 9H), 0.97 (t, *J* = 7.4 Hz, 3H) ppm.

^{13}C NMR (126 MHz, CDCl$_3$): δ 190.5, 156.3, 149.2, 135.4, 128.5, 126.0, 125.4, 35.1, 34.8, 31.1, 21.5, 13.8 ppm.

HRMS (ESI) m/z: calcd. For $(C_{16}H_{22}O + Na)^+$: 253.1568, found: 253.1571.

Analytical HPLC:

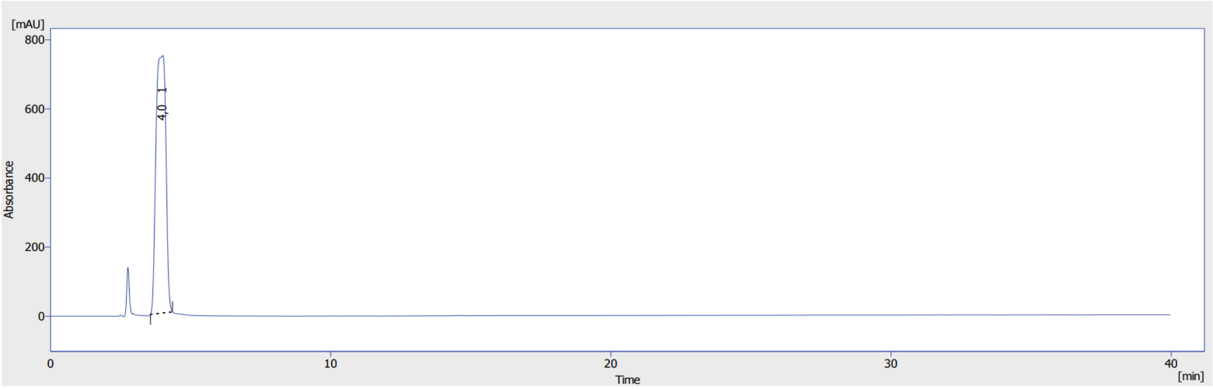

	Reten. Time [min]	Area [mAU.s]	Height [mAU]	Area [%]	Height [%]	W05 [min]	PDA Peak Purity	Compound Name	PDA Best Match Name	PDA Best Match
1	4,000	18330,178	745,353	100,0	100,0	0,42	922			
	Total	18330,178	745,353	100,0	100,0					

5.2.4 (*E*)-1-(4-chlorophenyl)hex-2-en-1-one (E6)

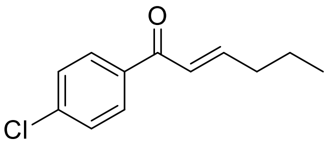

Following the literature procedure[1] compound **E6** was obtained from 1-(4-chlorophenyl)ethan-1-one (7.5 mmol) and butyraldehyde (15 mmol). The crude product was purified by column chromatography (10:90 AcOEt:Hexane) to afford 1.4 g ketone (**E6**) as a colorless oil. (**Yield = 89% over 3 steps**).

^{1}H NMR (500 MHz, CDCl$_3$): δ 7.89 – 7.84 (m, 2H), 7.46 – 7.40 (m, 2H), 7.06 (dt, J = 15.3, 7.0 Hz, 1H), 6.83 (dt, J = 15.4, 1.4 Hz, 1H), 2.29 (qd, J = 7.3, 1.4 Hz, 2H), 1.56 (sext., J = 7.4 Hz, 2H), 0.97 (t, J = 7.4 Hz, 3H) ppm.

^{13}C NMR (126 MHz, CDCl$_3$): δ 189.5, 150.4, 139.0, 136.3, 129.9, 128.8, 125.6, 34.9, 21.4, 13.7 ppm.

HRMS (ESI) m/z: calcd. for $(C_{12}H_{13}ClO + H)^+$: 209.0733, found: 209.0724.

Elemental analysis (%): calcd. for $C_{12}H_{13}ClO$: C 69.07, H 6.28; found: C 68.88, H 6.37.

5.2.5 (*E*)-1-(4-bromophenyl)hex-2-en-1-one (E7)

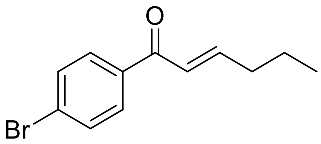

Following the literature procedure[1] compound **E7** was obtained from 1-(4-bromophenyl)ethan-1-one (7.5 mmol) and butyraldehyde (15 mmol). The crude product was purified by column chromatography (20:80 AcOEt:Hexane) to afford 0.74 g ketone (**E7**) as a yellow oil. (**Yield = 39% over 3 steps**).

^{1}H NMR (400 MHz, $CDCl_3$): δ 7.80 – 7.76 (m, 2H), 7.61 – 7.57 (m, 2H), 7.06 (dt, *J* = 15.4, 7.0 Hz, 1H), 6.82 (dt, *J* = 15.4, 1.5 Hz, 1H), 2.29 (qd, *J* = 7.2, 1.5 Hz, 2H), 1.55 (sext., *J* = 7.5 Hz, 2H), 0.97 (t, *J* = 7.4 Hz, 3H) ppm.
^{13}C NMR (126 MHz, $CDCl_3$): δ 189.7, 150.5, 136.7, 131.8, 130.0, 127.6, 125.5, 34.9, 21.4, 13.7 ppm.

HRMS (ESI) m/z: calcd. for $(C_{12}H_{13}BrO - H)^-$: 251.015, found: 251.0063.

Analytical HPLC:

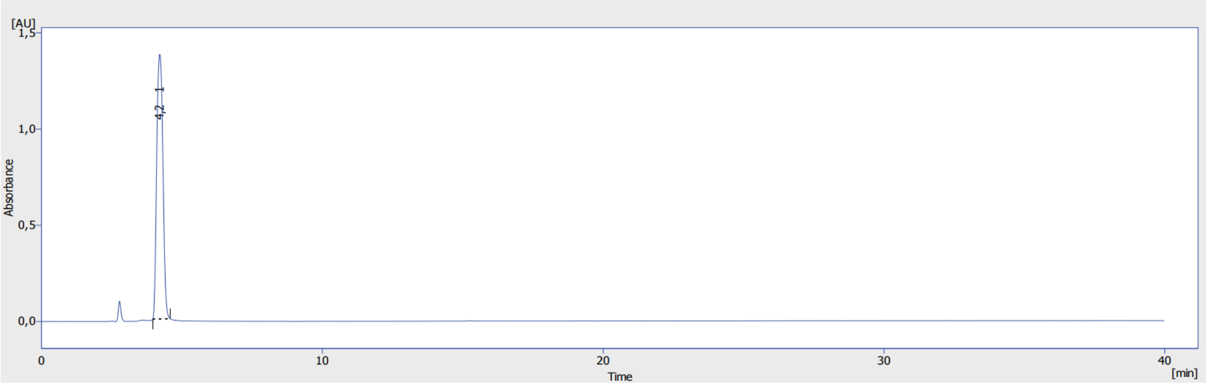

	Reten. Time [min]	Area [mAU.s]	Height [mAU]	Area [%]	Height [%]	W05 [min]	PDA Peak Purity	Compound Name	PDA Best Match Name	PDA Best Match
1	4,217	21087,811	1374,941	100,0	100,0	0,27	882			
	Total	21087,811	1374,941	100,0	100,0					

5.2.6 (*E*)-1-(benzo[*d*][1,3]dioxol-5-yl)hex-2-en-1-one (E8)

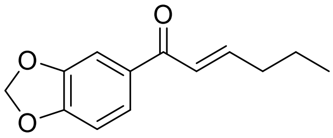

Following the literature procedure[1] compound **E8** was obtained from 1-(benzo[d][1,3]dioxol-5-yl)ethan-1-one (7.5 mmol) and butyraldehyde (15 mmol). The crude product was purified by column chromatography (5:95 Et_2O:Hexane) to afford 1.48 g ketone (**E8**) as a yellow oil. (**Yield = 90% over 3 steps**).

NMR data matched those reported in the literature.[1]

^{1}H NMR (400 MHz, CDCl$_3$): δ 7.54 (dd, *J* = 8.2, 1.7 Hz, 1H), 7.45 – 7.43 (m, 1H), 7.03 (dt, *J* = 15.3, 6.9 Hz, 1H), 6.87 – 6.79 (m, 2H), 6.03 (s, 2H), 2.28 (qd, *J* = 7.1, 1.5 Hz, 2H), 1.60 – 1.50 (m, 2H), 0.97 (t, *J* = 7.4 Hz, 3H) ppm.

5.2.8 (*E*)-1-(4-nitrophenyl)hex-2-en-1-one (E9)

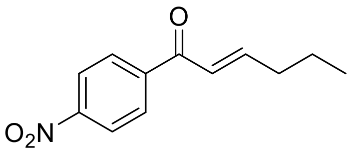

Following the general procedure **D** compound **E9** was obtained from 1-(4-nitrophenyl)-2-(triphenyl-λ^5-phosphaneylidene)ethan-1-one (3.3 mmol) and butyraldehyde (16.5 mmol). The crude product was purified by column chromatography (6:94 Et_2O:Hexane) to afford 0.51 g ketone (**E9**) as a yellow oil. (**Yield = 73% over 2 steps**).

^{1}H NMR (500 MHz, CDCl$_3$): δ 8.32 – 8.28 (m, 2H), 8.05 – 8.01 (m, 2H), 7.10 (dt, *J* = 15.4, 7.0 Hz, 1H), 6.83 (dt, *J* = 15.4, 1.5 Hz, 1H), 2.32 (qd, *J* = 7.2, 1.5 Hz, 2H), 1.56 (sext., *J* = 7.4 Hz, 2H), 0.98 (t, *J* = 7.4 Hz, 3H) ppm.
^{13}C NMR (126 MHz, CDCl$_3$): δ 189.4, 152.2, 150.0, 142.9, 129.4, 125.7, 123.7, 34.9, 21.3, 13.7 ppm.

HRMS (ESI) m/z: calcd. for ($C_{12}H_{13}NO_3$ - H)$^-$: 218.0895, found: 218.0819.

Analytical HPLC:

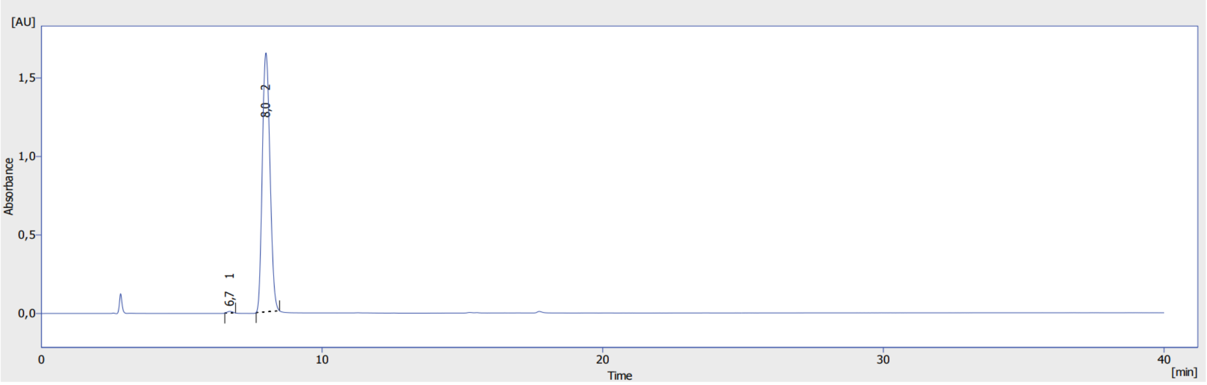

	Reten. Time [min]	Area [mAU.s]	Height [mAU]	Area [%]	Height [%]	W05 [min]	PDA Peak Purity	Compound Name	PDA Best Match Name	PDA Best Match
1	6,717	141,380	10,763	0,4	0,6	0,23	929			
2	8,000	31375,698	1648,726	99,6	99,4	0,32	915			
	Total	31517,077	1659,489	100,0	100,0					

5.2.9 (*E*)-1-(4-acetylphenyl)hex-2-en-1-one (E10)

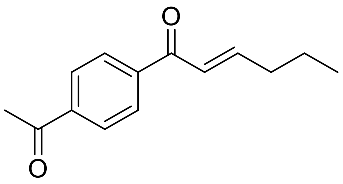

Following the literature procedure[1] compound **E10** was obtained from 1,1'-(1,4-phenylene)bis(ethan-1-one) (6.2 mmol) and butyraldehyde (12.3 mmol). The crude product was purified by column chromatography (20:80 AcOEt:Hexane) to afford 0.27 g ketone (**E10**) as a yellow oil in 20%.

^{1}H NMR (400 MHz, $CDCl_3$): δ 8.04 – 8.00 (m, 2H), 7.99 – 7.94 (m, 2H), 7.07 (dt, *J* = 15.4, 6.9 Hz, 1H), 6.85 (dt, *J* = 15.4, 1.5 Hz, 1H), 2.64 (s, 3H), 2.31 (qd, *J* = 7.3, 1.4 Hz, 2H), 1.56 (sext., *J* = 7.4 Hz, 2H), 0.98 (t, *J* = 7.4 Hz, 3H) ppm.

^{13}C NMR (101 MHz, $CDCl_3$): δ 190.5, 156.3, 149.2, 135.4, 128.5, 126.1, 125.4, 35.1, 34.8, 31.1, 21.5, 13.7 ppm.

HRMS (EI) m/z: calcd. for $C_{14}H_{16}O_2$: 216.1150, found: 216.1142.

Analytical HPLC:

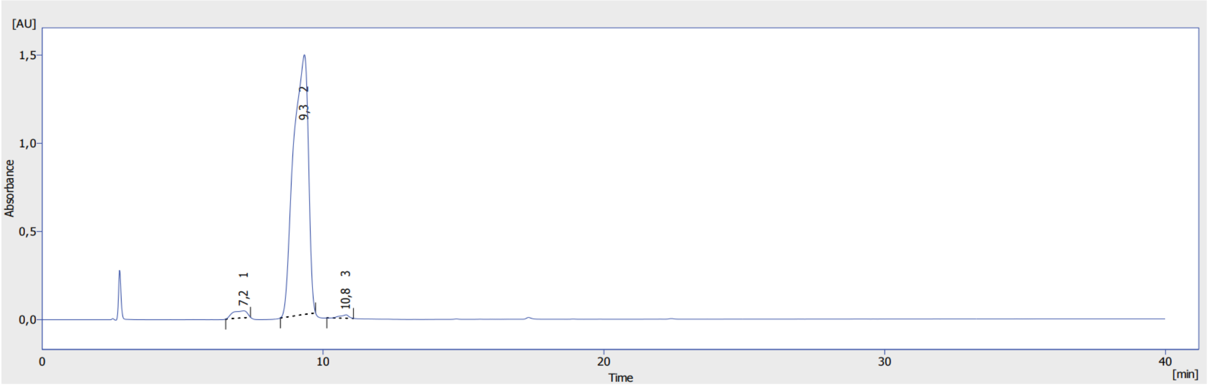

	Reten. Time [min]	Area [mAU.s]	Height [mAU]	Area [%]	Height [%]	W05 [min]	PDA Peak Purity	Compound Name	PDA Best Match Name	PDA Best Match
1	7,183	1465,306	39,317	2,6	2,6	0,63	922			
2	9,333	54118,134	1472,698	96,6	96,2	0,67	922			
3	10,817	433,641	19,324	0,8	1,3	0,43	998			
	Total	56017,081	1531,339	100,0	100,0					

5.2.10 (*E*)-4-(hex-2-enoyl)benzonitrile (E11)

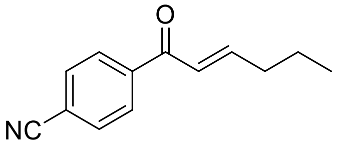

Following the literature procedure[1] compound **E11** was obtained from 4-acetylbenzonitrile (7.5 mmol) and butyraldehyde (15 mmol). The crude product was purified by column chromatography (20:80 AcOEt:Hexane) to afford 0.75 g ketone (**E11**) as a yellow oil. (**Yield = 50% over 3 steps**).

NMR data matched those reported in the literature.[1]

^{1}H NMR (400 MHz, CDCl$_3$): δ 8.00 – 7.95 (m, 2H), 7.78 – 7.74 (m, 2H), 7.09 (dt, *J* = 15.4, 7.0 Hz, 1H), 6.81 (dt, *J* = 15.4, 1.5 Hz, 1H), 2.31 (qd, *J* = 7.2, 1.5 Hz, 2H), 1.55 (sext., *J* = 7.4 Hz, 2H), 0.97 (t, *J* = 7.4 Hz, 3H) ppm.

5.2.11 1,4-bis(4-methoxyphenyl)but-2-en-1-one (*Z*/*E* mixture) (E12)

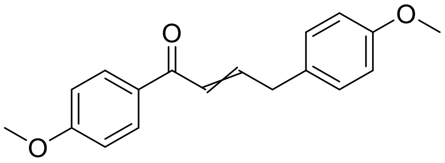

Following the general procedure **D** compound **E12** was obtained from 1-(4-methoxyphenyl)-2-(triphenyl-λ^5-phosphaneylidene)ethan-1-one (2.8 mmol) and 4-methoxybenzaldehyde (2.3 mmol). The crude product was purified by column chromatography (10:90 AcOEt:Hexane) to afford 0.55 g ketone (**E12**) as a white solid. (**Yield = 84% over 3 steps,** mixture of diastereoisomers ratio ~ 4:1).

^{1}H NMR (400 MHz, CDCl$_3$): (major) δ 7.91 (m, 2H), 7.13 (m, 2H), 6.88 (m, 6H), 6.82 3.86 (s, 3H), 3.80 (s, 3H), 3.57 (dd, *J* = 6.6, 1.2, 2H) ppm.

^{13}C NMR (400 MHz, CDCl$_3$): δ 189.0, 163.3, 158.4, 147.1, 132.27, 130.8, 130.6, 130.0, 129.8, 127.4, 126.2, 120.7, 114.1, 113.9, 113.8, 113.7, 55.4, 42.5, 38.1 ppm.

HRMS (ESI) m/z: calcd. for $(C_{18}H_{18}O_3 + Na)^+$: 305.1154, found: 305.1158.

Analytical HPLC:

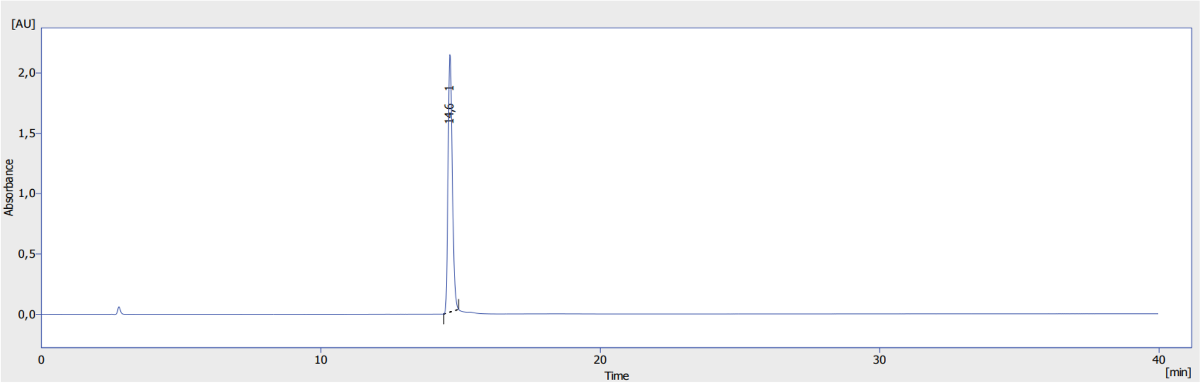

	Reten. Time [min]	Area [mAU.s]	Height [mAU]	Area [%]	Height [%]	W05 [min]	PDA Peak Purity	Compound Name	PDA Best Match Name	PDA Best Match
1	14,617	22597,238	2134,287	100,0	100,0	0,18	836			
	Total	22597,238	2134,287	100,0	100,0					

m.p.: 96-97 °C.

5.2.12 1-(4-methoxyphenyl)-4-(4-(trifluoromethyl)phenyl)but-2-en-1-one (*Z*/*E* mixture) (E13)

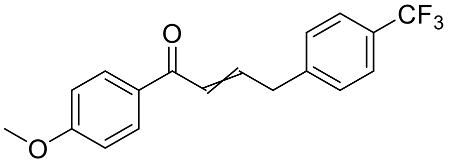

Following the general procedure **D** compound **E13** was obtained from 1-(4-methoxyphenyl)-2-(triphenyl-λ^5-phosphaneylidene)ethan-1-one (3.8 mmol) and 2-(4-(trifluoromethyl)phenyl)acetaldehyde (3.3 mmol). The crude product was purified by column chromatography (10:90 Et_2O:Hexane) to afford 0.58 g ketone (**E13**) as a colorless oil. (**Yield = 55% over 3 steps**, mixture diastereoisomers, ratio ~ 8:1).

^{1}H NMR (400 MHz, CDCl$_3$): (major) δ 7.98 (d, *J* = 8.9 Hz, 2H), 7.54 (d, *J* = 8.2 Hz, 2H), 7.46 (d, *J* = 8.1 Hz, 2H), 6.95 (d, *J* = 8.9 Hz, 2H), 6.58 (m, 2H), 3.88 (m, 5H) ppm.
^{13}C NMR (400 MHz, CDCl$_3$): (major) δ 196.04, 163.74, 140.50, 132.01, 130.60, 129.57, 126.41, 125.94, 125.51, 125.48, 125.45, 125.42, 113.90, 55.49, 42.23 ppm.
HRMS (ESI) m/z: calcd. For $(C_{18}H_{15}F_3O_2 + Na)^+$: 343.0922, found: 343.0921.
Analytical HPLC:

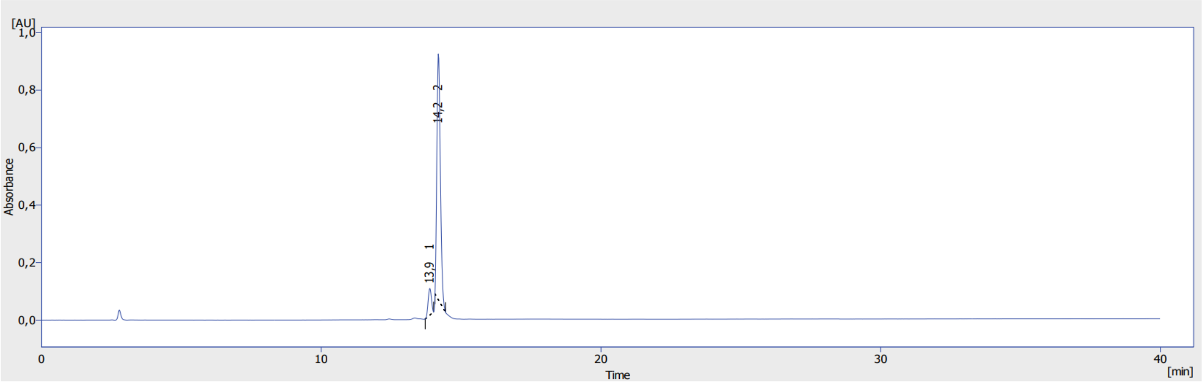

	Reten. Time [min]	Area [mAU.s]	Height [mAU]	Area [%]	Height [%]	W05 [min]	PDA Peak Purity	Compound Name	PDA Best Match Name	PDA Best Match
1	13,883	737,427	93,065	9,0	9,8	0,15	993			
2	14,183	7483,397	853,704	91,0	90,2	0,15	921			
	Total	8220,824	946,769	100,0	100,0					

5.2.13 (*E*)-1,4-bis(4-(trifluoromethyl)phenyl)but-2-en-1-one (E14)

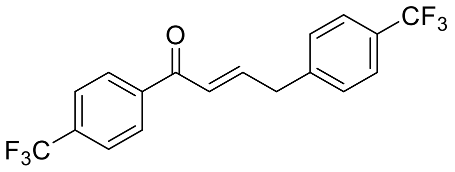

Following the general procedure **D** compound **E14** was obtained from 1-(4-(trifluoromethyl)phenyl)-2-(triphenyl-λ^5-phosphaneylidene)ethan-1-one (3.9 mmol) and 2-(4-(trifluoromethyl)phenyl)acetaldehyde (3.3

mmol). The crude product was purified by column chromatography (20:80 Et_2O:Hexane) to afford 0.27 g ketone (**E14**) as a yellow oil. (**Yield = 23% over 3 steps**).

^{1}H NMR (400 MHz, $CDCl_3$): δ 8.10 (d, *J* = 8.1 Hz, 2H), 7.76 (d, *J* = 8.4 Hz, 2H), 7.57 (d, *J* = 8.4 Hz, 2H), 7.48 (d, *J* = 8.2 Hz, 2H), 6.58 (m, 2H), 3.97 (d, *J* = 5.1 Hz, 2H) ppm.

^{13}C NMR (400 MHz, $CDCl_3$): δ 196.5, 140.2, 139.1, 134.9, 134.6, 132.8, 128.6, 126.5, 125.9, 125.8, 125.6, 125.5, 124.6,124.5, 42.7 ppm.

HRMS (ESI) m/z: calcd. for $(C_{18}H_{12}F_6O\text{-}H)^-$: 357.0792, found: 357.0711.

Analytical HPLC:

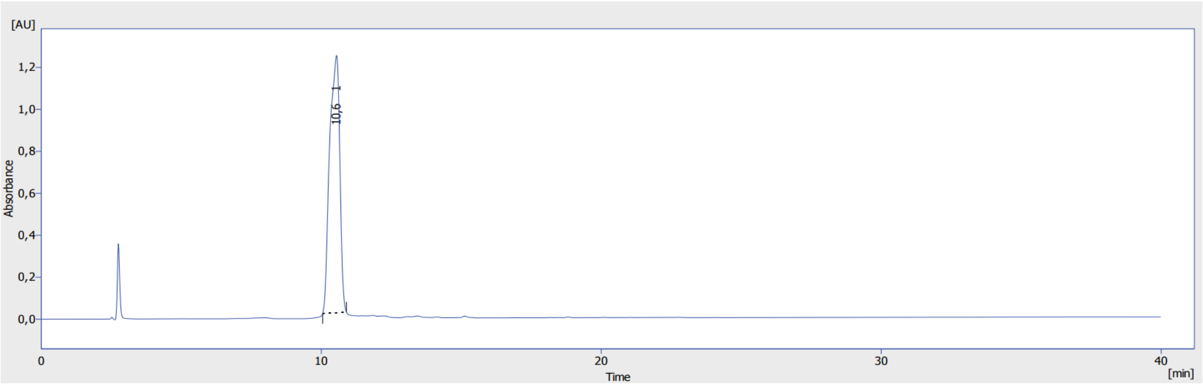

	Reten. Time [min]	Area [mAU.s]	Height [mAU]	Area [%]	Height [%]	W05 [min]	PDA Peak Purity	Compound Name	PDA Best Match Name	PDA Best Match
1	10,550	30631,966	1226,049	100,0	100,0	0,43	940			
	Total	30631,966	1226,049	100,0	100,0					

5.2.14 (*E*)-4-(4-methoxyphenyl)-1-(4-(trifluoromethyl)phenyl)but-2-en-1-one (E15)

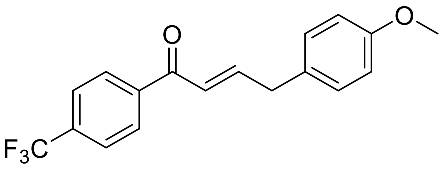

Following the general procedure **D** compound **E15** was obtained from 1-(4-(trifluoromethyl)phenyl)-2-(triphenyl-λ^5-phosphaneylidene)ethan-1-one (3.4 mmol) and 4-methoxybenzaldehyde (2.8 mmol). The crude product was purified by column chromatography (20:80 Et_2O:Hexane) to afford 0.16 g ketone (**E15**) as a yellow solid. (**Yield = 18% over 3 steps**).

^{1}H NMR (400 MHz, $CDCl_3$): δ 8.14 (d, *J* = 8.1 Hz, 1H), 8.00 (d, *J* = 8.1 Hz, 1H), 7.73 (dd, *J* = 16.5, 8.2 Hz, 2H), 7.31 (d, *J* = 8.7 Hz, 1H), 7.20 + 6.29 (dt, *J* = 15.4, 6.7 Hz, dt, *J* = 15.9, 6.84 1H), 7.13 (d, *J* = 8.6 Hz, 1H), 6.89 – 6.84 (m, 2H), 6.80 – 6.49 (m, 1H), 3.90 (dd, *J* = 6.8, 1.3 Hz, 1H), 3.80 (d, *J* = 2.4 Hz, 3H), 3.60 (dd, *J* = 6.5, 0.9 Hz, 1H) ppm.

^{13}C NMR (400 MHz, CDCl$_3$): δ 197.1, 190.0, 158.5, 149.7, 140.7, 139.3, 133.5, 129.8, 129.6, 129.4, 128.8, 128.7, 127.5, 126.2, 125.8, 125.7, 125.6, 125.5, 119.3, 114.2, 11 4.0, 55.3, 43.0, 38.2 ppm.

HRMS (ESI) m/z: calcd. for $(C_{18}H_{15}F_3O_2\text{-}H)^-$: 319.1024, found: 319.0945.

Analytical HPLC:

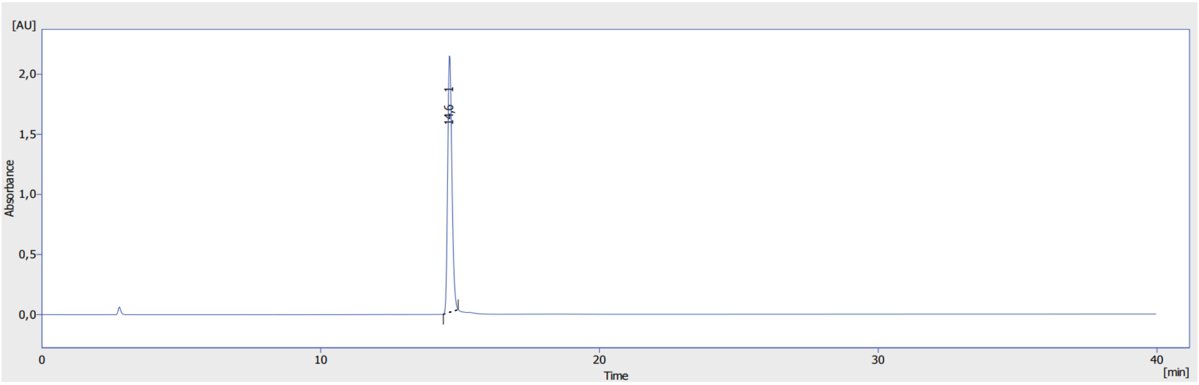

	Reten. Time [min]	Area [mAU.s]	Height [mAU]	Area [%]	Height [%]	W05 [min]	PDA Peak Purity	Compound Name	PDA Best Match Name	PDA Best Match
1	14,617	22597,238	2134,287	100,0	100,0	0,18	836			
	Total	22597,238	2134,287	100,0	100,0					

m.p.: 99-102 °C.

5.2.15 (*E*)-2,2-dimethyloct-4-en-3-one (E20)

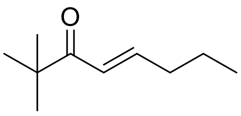

Following the general procedure **D** compound **E20** was obtained from 3,3-dimethyl-1-(triphenyl-λ^5-phosphaneylidene)butan-2-one (4.60 mmol) butyraldehyde (5.52 mmol). The crude product was purified by column chromatography (10:90 Et_2O:Pentane) to afford 0.39 g ketone (**E20**) as a yellow oil. (**Yield = 56% over 2 steps**).

NMR data matched those reported in the literature.[9]

^{1}H NMR (400 MHz, CDCl$_3$): major isomer δ 6.93 (dt, *J* = 15.2, 7.0 Hz, 1H), 6.49 (dt, *J* = 15.2, 1.5 Hz, 1H), 2.18 (qd, *J* = 7.2, 1.5 Hz, 2H), 1.49 (app sx, *J* = 7.4 Hz, 2H), 1.15 (s, 9H), 0.93 (t, *J* = 7.4 Hz, 3H) ppm.

5.3. Enols

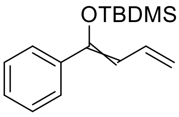

S1

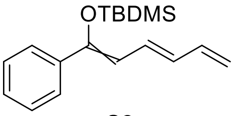

S2

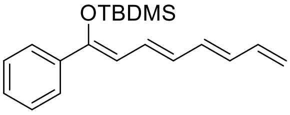

S3

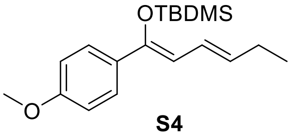

S4

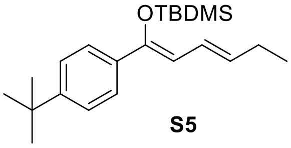

S5

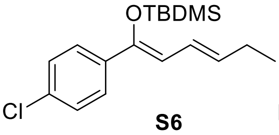

S6

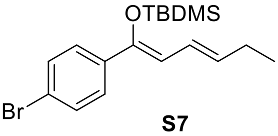

S7

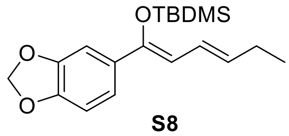

S8

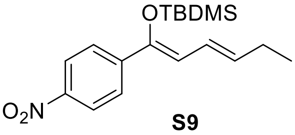

S9

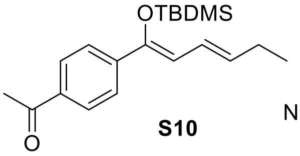

S10

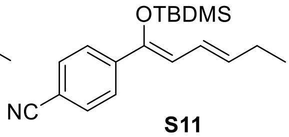

S11

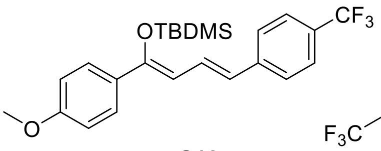

S12

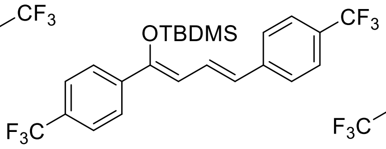

S13

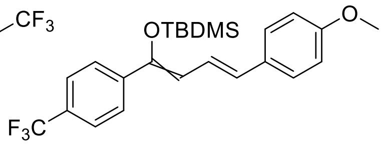

S14

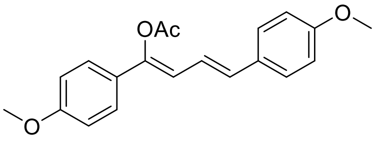

S15

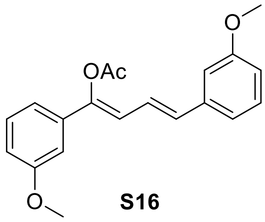

S16

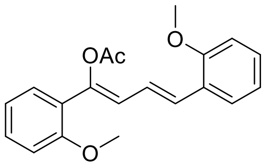

S17

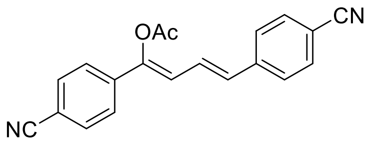

S18

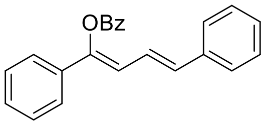

S19

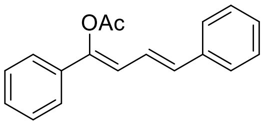

S20

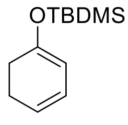

S21

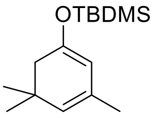

S22

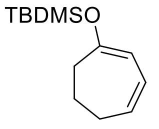

S23

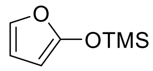

S24

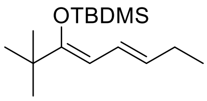

S25

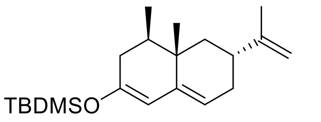

S26

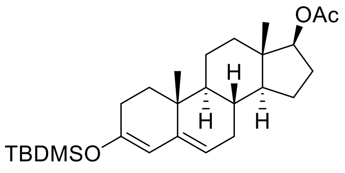

S27

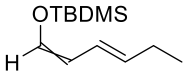

S28

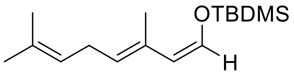

S29

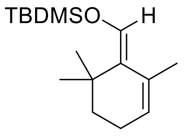

S30

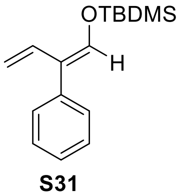

S31

Compounds **S1**, **S2**, **S3**, **S4**, **S5**, **S6**, **S7**, **S8**, **S10**, **S11**, **S12**, **S13**, **S14**, **S27** were prepared according to general procedure **B**.

Compounds **S23**, **S25**, **S28**, **S29**, **S30, S31** and **S32** were prepared according to general procedure **A**.

Compounds **S9**[7], **S15**[2], **S16**[2], **S17**[2], **S18**[2], **S19**[2], **S20**[2], **S21**[2], **S22**[3], **S26**[4] were prepared according to literature procedures.

Compound **S24** is commercially available and was used without further purification.

The crude product was passed through deactivated the Al_2O_3 (neutral Al_2O_3 treated with 15 wt% of H_2O) plug using pentane as an eluent. The filtrate after evaporation was used for the next step without further purification.

5.3.1 *tert*-butyldimethyl((1-phenylbuta-1,3-dien-1-yl)oxy)silane (*Z*/*E* mixture) (S1)

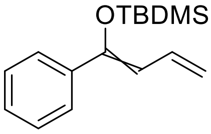

Following the general procedure **B** compound **S1** was obtained from 1-phenylbut-2-en-1-one (5.0 mmol). The reaction mixture was passed through a plug (deactivated, neutral Al_2O_3 treated with 15 wt% of H_2O) using hexane as an eluent. The crude product was used for the next step without further purification (1.18 g of compound **S1** as a colorless oil (**Yield = 91%**, 24 h, *Z*/*E* ratio ~ 1.1:1)).

^{1}H NMR (500 MHz, CDCl$_3$): δ 7.54–7.52 (m, 2H), 7.48–7.42 (m, 2H), 7.40–7.28 (m, 6H), 6.81 (dt, *J* = 17.1, 10.6 Hz, 1H), 6.51 (dt, *J* = 16.8, 10.6 Hz, 1H), 5.97 (d, *J* = 10.8 Hz, 1H), 5.82 (d, *J* = 11.1 Hz, 1H), 5.22 (dd, *J* = 17.1, 1.4 Hz, 1H), 5.13 (dd, *J* = 16.8, 1.5 Hz, 1H), 5.04 (dd, *J* = 10.3, 1.4 Hz, 1H), 4.88 (dd, *J* = 10.3, 1.5 Hz, 1H), 1.05 (s, 9H), 0.97 (s, 9H), 0.12 (s, 6H), 0.02 (s, 6H) ppm.

^{13}C NMR (125 MHz, CDCl$_3$): δ 153.3, 150.8, 138.9, 137.5, 133.7, 132.0, 128.8, 128.2, 128.0, 128.0, 127.9, 125.9, 114.2, 113.2, 112.7, 112.5, 25.9, 25.7, 18.4, 18.2, -4.0, -4.4 ppm.

HRMS (EI, *m/z*): calcd. for $C_{16}H_{24}OSi$: 260.1596; found: 260.1592.

5.3.2 *tert*-butyldimethyl(((3*E*)-1-phenylhexa-1,3,5-trien-1-yl)oxy)silane (*Z*/*E* mixture) (S2)

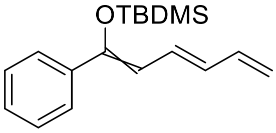

Following the general procedure **B** compound **S2** was obtained from 1-phenylhexa-2,4-dien-1-one (3.5 mmol, prepared according to literature procedure[4]). The reaction mixture was passed through a plug (deactivated, neutral Al_2O_3 treated with 15 wt% of H_2O) using hexane as an eluent. The crude product was used for the next step without further purification (0.68 g of compound **S2** as a yellow oil (**Yield = 68%**, 20 h, *Z*/*E* ratio ~ 1.5:1)).

^{1}H NMR (600 MHz, CDCl$_3$): δ 7.51 – 7.49 (m, 2H), 7.44 – 7.42 (m, 2H), 7.36 (t, *J* = 7.3 Hz, 1H), 7.33 – 7.29 (m, 2H + 1H), 7.26 (t, *J* = 7.3 Hz, 2H), 6.66 (ddd, *J* = 15.1, 11.1, 0.6 Hz, 1H), 6.48 – 6.37 (m, 1H + 1H), 6.34 – 6.15 (m, 1H + 2H), 5.99 (dd, *J* = 11.1, 0.5 Hz, 1H), 5.79 (d, *J* = 11.4 Hz, 1H), 5.20 (dt, *J* = 16.9, 1.8 Hz, 1H), 5.12 (dt, *J* = 16.8, 0.8 Hz, 1H), 5.04 (dt, *J* = 10.1, 0.7 Hz, 1H), 4.97 (dt, *J* = 10.1, 0.7 Hz, 1H), 1.03 (s, 9H), 0.94 (s, 9H), 0.09 (s, 6H), -0.00 (s, 6H) ppm.

^{13}C NMR (150 MHz, CDCl$_3$): δ 153.7, 151.4, 138.7, 137.8, 137.5, 137.4, 130.5, 130.2, 130.0, 128.73, 128.71, 128.3, 128.03, 127.96, 127.9, 125.7, 115.7, 114.8, 112.1, 111.8, 25.9, 25.7, 18.4, 18.2, -4.0, -4.4 ppm.

HRMS (EI, *m/z*): calcd. for $C_{18}H_{26}OSi$: 286.1753; found: 286.1758.

5.3.3 *tert*-butyldimethyl(((1*Z*,3*E*,5*E*)-1-phenylocta-1,3,5,7-tetraen-1-yl)oxy)silane (S3)

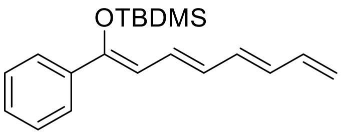

Following the general procedure **B** compound **S3** was obtained from (2*E*,4*E*,6*E*)-1-phenylocta-2,4,6-trien-1-one (2.5 mmol). The reaction mixture was passed through a plug (deactivated, neutral Al_2O_3 treated with 15 wt% of H_2O) using hexane as an eluent. The crude product was used for the next step without further purification (0.29 g of compound **S3** as a yellow oil (**Yield = 38%**)).

^{1}H NMR (400 MHz, CDCl$_3$): δ 7.58 – 7.17 (m, 6H), 6.94 – 4.94 (m, 7H), 1.07 – 0.99 (m, 9H), 0.02 – -0.02 (m, 6H) ppm.

^{13}C NMR (400 MHz, CDCl$_3$): δ 151.13, 138.7,137.3, 134.3, 132.1, 130.7, 130.6, 129.9, 129.1, 128.1, 128.0, 125.7, 116.5, 112.3, 25.9, 25.7, 18.5 ppm.

HRMS (APCI, *m/z*): calcd. for $(C_{20}H_{28}OSi + H)^+$: 313.1988; found: 313.1983.

5.3.4 *tert*-butyl(((1*Z*,3*E*)-1-(4-methoxyphenyl)hexa-1,3-dien-1-yl)oxy)dimethylsilane (S4)

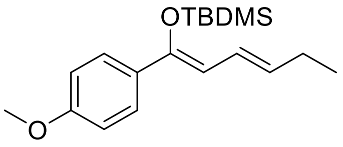

Following the general procedure **B** compound **S4** was obtained from (*E*)-1-(4-methoxyphenyl)hex-2-en-1-one (1.2 mmol). The reaction mixture was passed through a plug (deactivated, neutral Al_2O_3 treated with 15 wt% of H_2O) using hexane as an eluent. The crude product was used for the next step without further purification (0.27 g of compound **S4** as a yellow oil (**Yield = 70%**)).

^{1}H NMR (400 MHz, CDCl$_3$): δ 7.45 – 7.36 (m, 2H), 6.91 – 6.82 (m, 2H), 6.48 – 5.19 (m, 3H), 3.82 (dd, *J* = 7.1, 3.7 Hz, 3H), 2.25 – 2.02 (m, 2H), 1.06 – 1.01 (m, 9H), 0.95 – 0.92 (m, 3H), 0.10 (d, *J* = 15.4 Hz, 2H), 0.00 (d, *J* = 4.8 Hz, 4H) ppm.

^{13}C NMR (500 MHz, CDCl$_3$): δ 159.4, 159.2, 150.2, 148.4, 132.8, 132.4, 131.8, 131.0, 130.4, 130.01, 129.9, 126.9, 125.7, 124.4, 122.8, 113.4, 113.1, 111.5, 110.6, 106.8, 106.0, 55.2, 25.9, 25.7, 21.2, 18.4, 18.2, 14.3, 14.0, 13.6, -4.0, -4.4 ppm.

HRMS (EI, *m/z*): calcd. for ($C_{19}H_{30}O_2Si$ + H)$^+$: 319.2093; found: 319.2078.

5.3.5 *tert*-butyl(((1*Z*,3*E*)-1-(4-(tert-butyl)phenyl)hexa-1,3-dien-1-yl)oxy)dimethylsilane (S5)

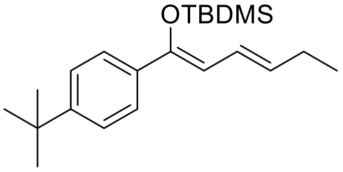

Following the general procedure **B** compound **S5** was obtained from (*E*)-1-(4-(tert-butyl)phenyl)hex-2-en-1-one (2.2 mmol). The reaction mixture was passed through a plug (deactivated, neutral Al_2O_3 treated with 15 wt% of H_2O) using hexane as an eluent. The crude product was used for the next step without further purification (0.35 g of compound **S5** as a yellow oil (**Yield = 47%**)).

^{1}H NMR (400 MHz, CDCl$_3$): δ 7.46 – 7.28 (m, 4H), 6.52 – 5.34 (m, 3H), 2.28 – 2.02 (m, 2H), 1.35 – 1.29 (m, 9H), 1.09 – 0.92 (m, 12H), 0.02 (d, *J* = 4.5 Hz, 6H) ppm.

^{13}C NMR (400 MHz, CDCl$_3$): δ 150.6, 148.6, 136.1, 133.2, 125.5, 125.3, 124.8, 124.5, 111.3, 34.5, 31.3, 25.9, 18.4, 13.6, -3.9 ppm.

HRMS (EI, *m/z*): calcd. for ($C_{22}H_{36}OSi$ + H)$^+$: 345.2614; found: 345.2611.

5.3.6 *tert*-butyl(((1*Z*,3*E*)-1-(4-chlorophenyl)hexa-1,3-dien-1-yl)oxy)dimethylsilane (S6)

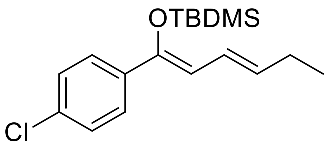

Following the general procedure **B** compound **S6** was obtained from (*E*)-1-(4-chlorophenyl)hex-2-en-1-one (0.96 mmol). The reaction mixture was passed through a plug (deactivated, neutral Al_2O_3 treated with 15 wt% of H_2O) using hexane as an eluent. The crude product was used for the next step without further purification (0.08 g of compound **S6** as a yellow oil (**Yield = 26%**)).

^{1}H NMR (400 MHz, CDCl$_3$): δ 7.45 – 7.27 (m, 4H), 6.55 – 6.31 (m, 1H), 6.15 – 5.95 (m, 1H), 5.81 – 5.59 (m, 1H), 2.25 – 2.03 (m, 2H), 1.09 – 0.78 (m, 12H), 0.14 – -0.06 (m, 6H) ppm.

^{13}C NMR (400 MHz, CDCl$_3$): δ 158.0, 151.1, 149.2, 147.4, 137.9, 137.7, 136.3, 134.4, 133.8, 133.6, 133.4, 133.2, 132.5, 132,2, 131.2, 130.1, 129.9, 129.7, 128.7, 128.2, 128.1, 128.0, 127.0, 126.8, 125.0, 124.5, 124.1, 123.7, 122.4, 112.8, 112.4, 108.0, 107.7, 106.2, 106.1, 25.8, 25.7, 21.5, 21.3, 21.1, 18.4, 18.1, 14.2, 13.9, 13.5, 12.2, -4.0, -4.4 ppm.

HRMS (EI, *m/z*): calcd. for ($C_{18}H_{27}ClOSi$ + H)$^+$: 323.1598; found: 323.1588.

5.3.7 (((1*Z*,3*E*)-1-(4-bromophenyl)hexa-1,3-dien-1-yl)oxy)(tert-butyl)dimethylsilane (S7)

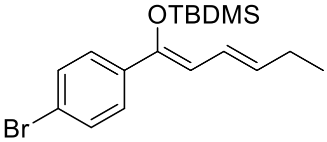

Following the general procedure **B** compound **S7** was obtained from (*E*)-1-(4-bromophenyl)hex-2-en-1-one (2.4 mmol). The reaction mixture was passed through a plug (deactivated, neutral Al_2O_3 treated with 15 wt% of H_2O) using hexane as an eluent. The crude product was used for the next step without further purification (0.73 g of compound **S7** as a yellow oil (**Yield = 83%**)).

^{1}H NMR (400 MHz, CDCl$_3$): δ 7.48 – 7.30 (m, 4H), 6.45 – 5.24 (m, 3H), 2.25 – 2.02 (m, 2H), 1.02 – 1.01 (m, 3H), 0.94 – 0.87 (m, 9H), 0.11 – 0.00 (m, 6H) ppm.

^{13}C NMR (400 MHz, CDCl$_3$): δ 136.8, 134.5, 133.8, 132.6, 131.2, 131.1, 131.0, 130.2, 127.3, 127.1, 125.0, 124.1, 123.7, 122.5, 121.3, 112.9, 112.5, 108.0, 107.8, 26.0, 25.9, 25.7, 21.3, 21.1, 18.4, 18.1, 13.5, -2.9, -4.0, -4.4 ppm.

HRMS (EI, *m/z*): calcd. for $(C_{18}H_{27}BrOSi + H)^+$: 367.1093; found: 367.1084.

5.3.8 (((1*Z*,3*E*)-1-(benzo[*d*][1,3]dioxol-5-yl)hexa-1,3-dien-1-yl)oxy)(tert-butyl)dimethyl silane (S8)

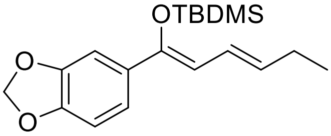

Following the general procedure **B** compound **S8** was obtained from (*E*)-1-(benzo[*d*][1,3]dioxol-5-yl)hex-2-en-1-one (3.2 mmol). The reaction mixture was passed through a plug (deactivated, neutral Al_2O_3 treated with 15 wt% of H_2O) using hexane as an eluent. The crude product was used for the next step without further purification (0.38 g of compound **S8** as a yellow oil (**Yield = 36%**)).

^{1}H NMR (400 MHz, CDCl$_3$): δ 7.05 – 6.90 (m, 2H), 6.81 – 6.71 (m, 1H), 6.48 – 5.17 (m, 5H), 2.26 – 1.99 (m, 2H), 1.10 – 0.87 (m, 12H), 0.12 – -0.01 (m, 6H) ppm.

^{13}C NMR (400 MHz, CDCl$_3$): δ 150.0, 147.4, 147.2, 133.9, 132.8, 131.4, 130.3, 125.5, 122.7, 119.8, 119.5, 111.8, 111.0, 109.0, 107.8, 106.5, 101.0, 25.9, 25.7, 21.2, 18.4, 14.3, -4.0, -4.4 ppm.

HRMS (APCI, *m/z*): calcd. for $(C_{19}H_{28}O_3Si + H)^+$: 333.1886; found: 333.1887.

5.3.9 *tert*-butyldimethyl(((1*Z*,3*E*)-1-(4-nitrophenyl)hexa-1,3-dien-1-yl)oxy)silane (S9)

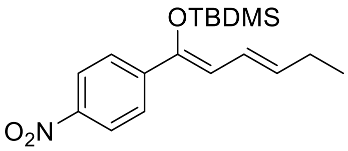

Following the general procedure **B** compound **S9** was obtained from (*E*)-1-(4-nitrocyclohexa-1,3-dien-1-yl)hex-2-en-1-one (1.6 mmol). The reaction mixture was passed through a plug (deactivated, neutral Al_2O_3 treated with 15 wt% of H_2O) using hexane as an eluent. The crude product was used for the next step without further purification (0.45 g of compound **S9** as a yellow oil (**Yield = 85%**)).

^{1}H NMR (400 MHz, CDCl$_3$): (major) δ 8.22 – 8.15 (m, 2H), 7.6 (dd, *J* = 8.8, 3.5 Hz, 2H), 6.16 – 6.01 (m, 2H), 5.92– 5.54 (m, 1H), 2.29 – 2.06 (m, 2H), 0.94 (d, *J* = 8.61 Hz, 9H), 0.12 (d, *J* = 16.12 Hz, 6H), 0.01 (d, *J* = 5.37 Hz, 3H) ppm.

^{13}C NMR (400 MHz, CDCl$_3$): δ 149.5, 147.8, 147.1, 144.4, 144.0, 137.1, 135.9, 134.9, 133.2, 129.4, 129.2, 126.0, 125.7, 124.2, 123.9, 123.5, 123.2, 122.9, 122.2, 115.8, 115.0, 110.8, 110.0, 26.1, 26.0, 25.8, 25.7, 25.6, 21.4, 21.2, 18.2, 18.1, 14.1, 13.7, 13.3, -3.9, -4.4 ppm.

HRMS (APCI, *m/z*): calcd. for $(C_{18}H_{27}NO_3Si + H)^+$: 334.1838; found: 334.1843.

5.3.10 1-(4-((1*Z*,3*E*)-1-((*tert*-butyldimethylsilyl)oxy)hexa-1,3-dien-1-yl)phenyl)ethan-1-one (S10)

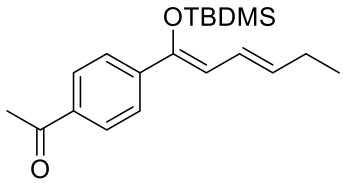

Following the general procedure **B** compound **S10** was obtained from (*E*)-1-(4-acetylphenyl)hex-2-en-1-one (1.3 mmol). The reaction mixture was passed through a plug (deactivated, neutral Al_2O_3 treated with 15 wt% of H_2O) using hexane as an eluent. The crude product was used for the next step without further purification (0.06 g compound **S10** as a yellow oil (**Yield = 15%**)).

^{1}H NMR (500 MHz, CDCl$_3$): δ 7.93 – 7.86 (m, 2H), 7.64 – 7.53 (m, 2H), 6.52 – 5.79 (m, 3H), 2.59 (d, *J* = 3.1 Hz, 3H), 2.29 – 2.09 (m, 2H), 1.10 – 0.99 (m, 12H), 0.01 – -0.02 (m, 6H) ppm.

^{13}C NMR (500 MHz, CDCl$_3$): δ 155.7, 150.1, 133.9,133.2, 132.0, 130.7, 128.5, 128.3, 125.5, 125.2, 124.9, 124.7, 124.4, 122.7, 112.7, 112.2, 107.5, 90.9, 30.3, 29.7, 25.9, 25.7, 18.1, 14.3, 13.5, -2.9, -4.4, -4.6 ppm.

HRMS (APCI, *m/z*): calcd. for $(C_{20}H_{30}O_2Si + H)^+$: 331.2093, found: 331.2099.

5.3.11 4-((1*Z*,3*E*)-1-((*tert*-butyldimethylsilyl)oxy)hexa-1,3-dien-1-yl)benzonitrile (S11)

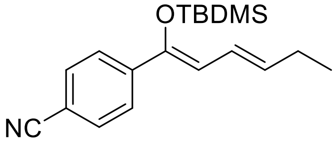

Following the general procedure **B** compound **S11** was obtained from (*E*)-1-(4-acetylphenyl)hex-2-en-1-one (2.5 mmol). The reaction mixture was passed through a plug (deactivated, neutral

Al_2O_3 treated with 15 wt% of H_2O) using hexane as an eluent. The crude product was used for the next step without further purification (0.30 g of compound **S11** as a yellow oil (**Yield = 38%**)).

^{1}H NMR (500 MHz, CDCl$_3$): δ 7.66 – 7.50 (m, 4H), 6.60 – 5.26 (m, 3H), 2.36 – 1.92 (m, 2H), 1.09 – 0.88 (m, 12H), 0.10 – -0.03 (m, 6H) ppm.

^{13}C NMR (500 MHz, CDCl$_3$): δ 143.5, 136.5, 131.9, 131.7, 129.0, 125.7, 123.9, 118.9, 115.0, 110.6, 25.8, 18.4, 13.3, -4.0, -4.4 ppm.

HRMS (APCI, *m/z*): calcd. for $(C_{19}H_{27}NOSi + H)^+$: 314.1940; found: 314.1943

5.3.12 *tert*-butyl(((1*Z*,3*E*)-1-(4-methoxyphenyl)-4-(4-(trifluoromethyl)phenyl)buta-1,3-dien-1-yl)oxy)dimethylsilane (S12)

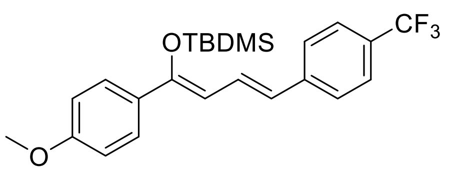

Following the general procedure **B** compound **S12** was obtained from (*E*)-1-(4-methoxyphenyl)-4-(4-(trifluoromethyl)phenyl)but-2-en-1-one (0.9 mmol). The reaction mixture was passed through a plug (deactivated, neutral Al_2O_3 treated with 15 wt% of H_2O) using hexane as an eluent. The crude product was used for the next step without further purification (0.18 g of compound **S12** as a yellow solid (**Yield = 46%**)).

^{1}H NMR (500 MHz, CDCl$_3$): δ 7.55 (d, *J* = 8.1 Hz, 2H), 7.49 – 7.45 (m, 4H), 7.34 (dd, *J* = 15.7, 10.9 Hz, 1H), 6.88 (d, *J* = 8.8 Hz, 2H), 6.50 (d, *J* = 15.8 Hz, 1H), 6.05 (d, *J* = 10.9 Hz, 1H), 3.84 (s, 3H), 1.08 (s, 9H), 0.05 (s, 6H) ppm.

^{13}C NMR (500 MHz, CDCl$_3$): δ 159.9, 152.8, 142.0, 131.0, 128.3, 128.1, 127.3, 127.1, 126.6, 125.8, 125.6, 125.5, 113.6, 113.5, 110.3, 55.3, 30.9, 25.9, 18.5, -4.0 ppm.

HRMS (APCI, *m/z*): calcd. for $(C_{24}H_{29}F_3O_2Si + H)^+$: 435.1967; found: 435.1965.

5.3.13 (((1*Z*,3*E*)-1,4-bis(4-(trifluoromethyl)phenyl)buta-1,3-dien-1-yl)oxy)(*tert*-butyl) dimethylsilane (S13)

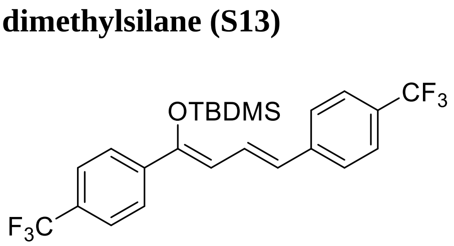

Following the general procedure **B** compound **S13** was obtained from (*E*)-1,4-bis(4-(trifluoromethyl)phenyl)but-2-en-1-one (0.75 mmol). The reaction mixture was passed through a plug (deactivated, neutral Al_2O_3 treated with 15 wt% of H_2O) using hexane as an eluent. The crude product was used for the next step without further purification (0.12 g of compound **S13** as a yellow oil (**Yield = 35%**)).

^{1}H NMR (500 MHz, $CDCl_3$): δ 7.66 (d, *J* = 8.4 Hz, 2H), 7.61 – 7.57 (m, 4H), 7.49 (d, *J* = 8.2 Hz, 2H), 7.32 (dd, *J* = 15.8, 10.9 Hz, 1H), 6.60 (d, *J* = 15.8 Hz, 1H), 6.22 (d, *J* = 10.9 Hz, 1H), 1.09 (s, 9H), 0.5 (s, 6H) ppm.

^{13}C NMR (500 MHz, $CDCl_3$): δ 151.1, 141.9, 141.3, 130.04, 130.02, 129.9,129.7, 129.0, 126.2, 126.1, 125.2, 123.1, 123.0, 113.4, 30.9, 25.8, 18.5, -4.0 ppm.

HRMS (EI, *m/z*): decomposition

5.3.14 *tert*-butyl(((1*Z*,3*E*)-4-(4-methoxyphenyl)-1-(4-(trifluoromethyl)phenyl)buta-1,3-dien-1-yl)oxy)dimethylsilane (*Z*/*E* mixture) (S14)

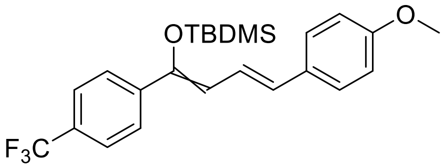

Following the general procedure **B** compound **S14** was obtained from (*E*)-1,4-bis(4-(trifluoromethyl)phenyl)but-2-en-1-one (0.75 mmol). The reaction mixture was passed through a plug (deactivated, neutral Al_2O_3 treated with 15 wt% of H_2O) using hexane as an eluent. The crude product was used for the next step without further purification (0.22 g of compound **S14** as a yellow solid (**Yield = 70 %**, *Z*/*E* mixture, ratio ~ 1:5)).

^{1}H NMR (500 MHz, $CDCl_3$δ 7.80 (d, *J* = 7.9 Hz, 2H), 7.69 (d, *J* = 8.4 Hz, 2H), 7.64 (d, *J* = 8.1 Hz, 2H), 7.67 (d, *J* = 8.4 Hz, 2H), 7.36 (d, *J* = 8.7 Hz, 2H), 7.24 (d, *J* = 8.9 Hz, 2H), 7.09 (dd, *J* = 15.8, 10.9 Hz, 1H), 6.96 (d, *J* = 8.8 Hz, 2H), 6.88 (d, *J* = 8.7 Hz, 2H), 6.84-6.82, (m, 1H), 6.75 (dd, *J* = 13.5, 11.0 Hz, 1H), 6.55 (d, *J* = 15.8 Hz, 1H), 6.20 (d, *J* = 10.9 Hz, 1H), 5.98 (d, *J* = 11.1 Hz, 1H), 3.86 (s, 3H), 3.82 (s, 3H), 1.08 (s, 9H), 0.96 (s, 9H), 0.13 (s, 6H), 0.05 (s, 6H) ppm.

^{13}C NMR (500 MHz, $CDCl_3$): δ 159.1, 148.6, 142.3, 130.7, 130.5, 129.5, 129.3, 128.9, 127.4, 125.5, 125.1, 123.4, 121.9, 114.2, 109.3, 105.9, 55.3, 30.9, 25.9, 18.5, -3.9 ppm.

HRMS (EI, *m/z*): calcd. for $(C_{24}H_{29}F_3O_2Si + H)^+$: 435.1967; found: 435.1969.

5.3.15 (1*Z*,3*E*)-1,4-bis(4-methoxyphenyl)buta-1,3-dien-1-yl acetate (S15)

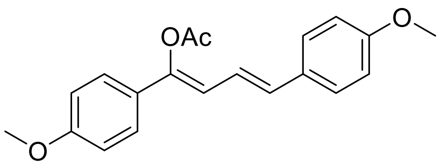

Compound **S15** was obtained from 1-ethynyl-4-methoxybenzene (2.5 mmol) according to literature procedure[2]. The crude was purified by column chromatography (20:80 AcOEt/Hexane) to afford 0.21 g compound **S15** as a colorless oil (**Yield = 52%**).

NMR data matched those reported in the literature.[2]

^{1}H NMR (400 MHz, $CDCl_3$): δ 7.43 – 7.39 (m, 2H), 7.30 – 7.27 (m, 2H), 6.95 – 6.92 (m, 2H), 6.85 – 6.78 (m, 3H), 6.59 (d, *J* = 15.6 Hz, 1H), 6.20 – 6.17 (m, 1H), 3.85 (s, 3H), 3.80 (s, 3H), 2.19 (s, 3H) ppm.

5.3.16 (1*Z*,3*E*)-1,4-bis(3-methoxyphenyl)buta-1,3-dien-1-yl acetate (S16)

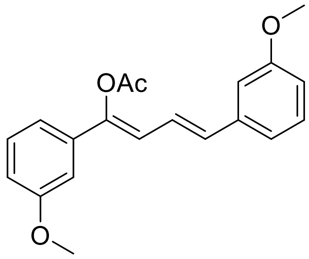

Compound **S16** was obtained from 1-ethynyl-3-methoxybenzene (2.5 mmol) according to literature procedure.[2] The crude was purified by column chromatography (20:80 AcOEt/Hexane) to afford 0.34 g compound **S16** as a yellow oil (**Yield = 83%**).

^{1}H NMR (500 MHz, $CDCl_3$): δ 7.34 (t, *J* = 8.0 Hz, 1H), 7.22 (t, *J* = 7.9 Hz, 1H), 7.09 (dt, *J* = 7.6, 1.0 Hz, 1H), 7.03 – 6.91 (m, 4H), 6.88 (t, *J* = 2.1 Hz, 1H), 6.78 (dd, *J* = 8.2, 0.8 Hz, 1H), 6.64 (d, *J* = 15.6 Hz, 1H), 6.27 (dd, *J* = 11.3, 0.7 Hz, 1H), 3.83 (s, 3H), 3.80 (s, 3H), 2.21 (s, 3H) ppm.

^{13}C NMR (500 MHz, $CDCl_3$): δ 206.8, 169.4, 159.8, 159.6, 148.2, 138.6, 135.8, 134.3, 129.5, 123.6, 121.0, 120.5, 119.1, 114.6, 113.8, 113.2, 112.0, 55.3, 30.9, 21.0 ppm.

HRMS (EI, *m/z*): calcd. for $(C_{20}H_{20}O_4 + Na)^+$: 347.1259; found: 347.1266.

5.3.17 (1*Z*,3*E*)-1,4-bis(2-methoxyphenyl)buta-1,3-dien-1-yl acetate (S17)

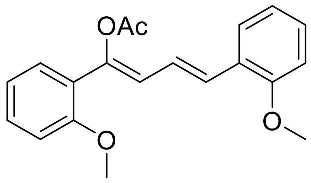

Compound **S17** was obtained from 1-ethynyl-2-methoxybenzene (2.5 mmol) according to literature procedure[2]. The crude was purified by column chromatography (20:80 AcOEt/Hexane) to afford 0.10 g compound **S17** as a yellow oil (**Yield = 23%**, *Z*/*E* ratio ~ 1:1).

^{1}H NMR (500 MHz, $CDCl_3$): δ 7.51 (dd, *J* = 7.7, 1.7 Hz, 1H), 7.41 (dd, *J* = 7.5, 1.8 Hz, 1H), 7.39 – 7.34 (m, 2H), 7.31 – 7.28 (m, 1H), 7.24 (t, *J* = 1.6 Hz, 1H), 7.24 – 7.15 (m, 2H), 7.07 – 6.92 (m, 8H), 6.89 – 6.82 (m, 4H), 6.68 (dd, *J* = 15.8, 11.2 Hz, 1H), 6.36 (dd, *J* = 11.2, 0.6 Hz, 1H), 3.91 (s, 3H), 3.87 (s, 3H), 3.85 (s, 3H), 3.81 (s, 3H), 2.33 (s, 3H), 2.15 (s, 3H) ppm.

^{13}C NMR (500 MHz, $CDCl_3$): δ 169.2, 168.7, 157.3, 157.0, 156.8, 156.7, 145.9, 143.5, 132.1, 130.4, 129.3, 128.8, 128.5, 128.1, 127.7, 126.7, 126.5, 124.4, 123.8, 123.2, 123.1, 122.4, 122.0, 120.7, 120.6, 120.3, 111.5, 111.3, 111.0, 110.9, 55.6, 55.5, 21.1 ppm.

HRMS (EI, *m/z*): calcd. for $(C_{20}H_{20}O_4 + Na)^+$: 347.1259; found: 347.1263.

5.3.18 (1*Z*,3*E*)-1,4-bis(4-cyanophenyl)buta-1,3-dien-1-yl acetate (S18)

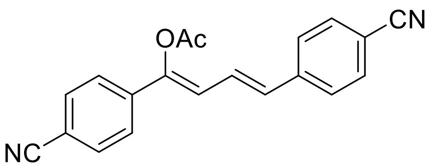

Compound **S18** was obtained from 4-ethynylbenzonitrile (2.5 mmol) according to literature procedure[2]. The crude was purified by column chromatography (30:70 AcOEt/Hexane) to afford 0.35 g compound **S18** as a yellow solid (**Yield = 88%**).

NMR data matched those reported in the literature. [2]

^{1}H NMR (400 MHz, CDCl$_3$): δ 7.73 (d, *J* = 8.5 Hz, 2H), 7.59 – 7.55 (m, 4H), 7.40 (d, *J* = 8.3 Hz, 2H), 6.93 (dd, *J* = 15.5, 11.3 Hz, 1H), 6.71 (d, *J* = 15.6 Hz, 1H), 6.39 (d, *J* = 11.2 Hz, 1H), 2.22 (s, 3H) ppm.

5.3.19 *tert*-butyl((5,5-dimethyl-3-methylenecyclohex-1-en-1-yl)oxy)dimethylsilane (S22)

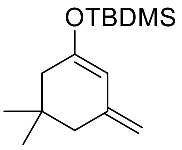

Following the general procedure **A** compound **S22** was obtained from 3,5,5-trimethylcyclohex-2-en-1-one (2.5 mmol). The reaction mixture was passed through a plug (deactivated, neutral Al_2O_3 treated with 15 wt% of H_2O) using hexane as an eluent. The crude product was used for the next step without further purification (0.39 g of compound **S22** as a colorless oil (**Yield = 61%**).

NMR data matched those reported in the literature.[3]

^{1}H NMR (400 MHz, CDCl$_3$): δ 5.47 (s, 1H), 4.61 (s, 1H), 4.56 (s, 1H), 2.00 (t, *J* = 1.3 Hz, 2H), 1.93 (s, 2H), 0.94 (s, 6H), 0.93 (s, 9H), 0.16 (s, 6H) ppm.

5.3.20 *tert*-butyl(cyclohepta-1,3-dien-1-yloxy)dimethylsilane (S23)

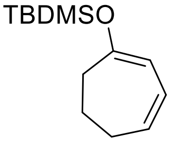

Following the general procedure **A** compound **S23** was obtained from cyclohept-2-en-1-one (5.0 mmol). The reaction mixture was passed through a plug (deactivated, neutral Al_2O_3 treated with 15 wt% of H_2O) using hexane as an eluent. The crude product was used for the next step without further purification (1.1 g of compound **S23** as a colorless oil (**Yield = 99%**)).

NMR data matched those reported in the literature.[10]

^{1}H NMR (400 MHz, CDCl$_3$): δ 5.79 (dt, 1H), 5.61 (dq, 1H), 5.17 (dt, 1H), 2.3 (q, *J* = 6.3 Hz, 2H), 2.2 (q, *J* = 5.9 Hz, 2H), 1.8 (p, *J* = 5.8 Hz, 2H), 0.9 (s, 9H), 0.1 (d, *J* = 14.3 Hz, 6H) ppm.

5.3.21 *tert*-butyl(((3*Z*,5*E*)-2,2-dimethylocta-3,5-dien-3-yl)oxy)dimethylsilane (S25)

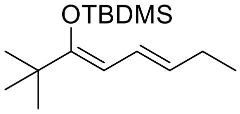

Following the general procedure, **A** compound **S25** was obtained from (*E*)-2,2-dimethyloct-4-en-3-one (1.61 mmol). The reaction mixture was passed through a plug (deactivated, neutral Al_2O_3 treated with 15 wt% of H_2O) using hexane as an eluent. The crude product was used for the next step without further purification (0.16 g of compound **S25** as a colorless oil (**Yield = 38%**)).

^{1}H NMR (400 MHz, $CDCl_3$): δ 6.48 – 6.15 (m, 1H), 5.57 – 5.49 (m, 1H), 5.39 – 5.12 (m, 1H), 2.15 – 2.06 (m, 2H), 1.10 (d, *J* = 10.7 Hz, 9H), 1.01 (dd, *J* = 8.1, 2.7 Hz, 9H), 0.95 (d, *J* = 7.0 Hz, 3H), 0.21 (d, *J* = 2.5 Hz, 3H), 0.19 (d, *J* = 5.9 Hz, 3H) ppm.
^{13}C NMR (126 MHz, $CDCl_3$): 160.5, 131.0, 129.2, 128.4, 124.7, 123.3, 104.8, 103.4, 100.2, 36.7, 29.8, 28.8, 26.0, 21.0, 14.2, 13.9 ppm.
HRMS (APCI, *m/z*): calcd. For $(C_{16}H_{32}OSi + H)^+$: 269.2301, found: 269.2303.

5.3.22 *tert*-butyl(((4*R*,4a*S*,6*R*)-4,4a-dimethyl-6-(prop-1-en-2-yl)-3,4,4a,5,6,7-hexahydronaphthalen-2-yl)oxy)dimethylsilane (S26)

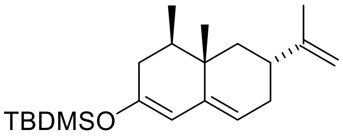

Following the general procedure **B** compound **S26** was obtained from (4*R*,4a*S*,6*R*)-4,4a-dimethyl-6-(prop-1-en-2-yl)-4,4a,5,6,7,8-hexahydronaphthalen-2(3H)-one (2.5 mmol). The reaction mixture was passed through a plug (deactivated, neutral Al_2O_3 treated with 15 wt% of H_2O) using hexane as an eluent. The crude product was used for the next step without further purification (0.80 of compound **S26** as a white solid (**Yield = 96%**)).
^{1}H NMR (400 MHz, $CDCl_3$): δ 5.37 – 5.20 (m, 2H), 4.73 – 4.70 (m, 2H), 2.44 – 2.12 (m, 2H), 2.03 – 1.85 (m, 3H), 1.74-1.71 (m, 3H), 1.68 (d, *J* = 12.3 Hz, 1H), 1.63 – 1.52 (m, 1H), 1.38 – 1.19 (m, 1H), 0.92 (s, 9H), 0.90-0.84 (m, 6H), 0.15 – 0.12 (m, 6H), ppm.
^{13}C NMR (126 MHz, $CDCl_3$): δ 150.8, 150.5, 141.6, 119.9, 118.3, 108.6, 108.5, 106.8, 45.6, 41.5, 40.2, 39.1, 37.4, 36.7, 35.9, 31.4, 31.2, 25.7, 20.9, 20.7, 18.0, 17.5, 14.6, 14.3, -4.2, -4.4 ppm.
HRMS (EI, *m/z*): calcd. for $(C_{21}H_{36}OSi + H)^+$: 333.2614; found: 333.2622.

5.3.23 (1*R*,8*R*,9*S*,13*S*,14*S*,17*S*)-3-((*tert*-butyldimethylsilyl)oxy)-1,13-dimethyl-2,7,8,9,10,11,12,13,14,15,16,17-dodecahydro-1H-cyclopenta[a]phenanthren-17-yl acetate (S27)

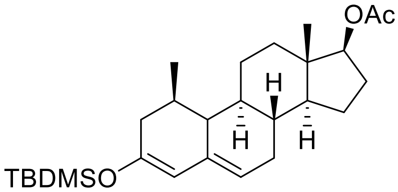

Following the general procedure **B** compound **S27** was obtained from (1*R*,8*R*,9*S*,13*S*,14*S*,17*S*)-1,13-dimethyl-3-oxo 2,3,6,7,8,9,10,11,12,13,14,15,16,17-tetradecahydro-1H-cyclopenta[*a*]phenanthren-17-yl acetate (1.7 mmol). The reaction mixture was passed through a plug (deactivated, neutral Al_2O_3 treated with 15 wt% of H_2O) using hexane as an eluent. The crude product was used for the next step without further purification (0.43 of compound **S27** as a white solid (**Yield = 57%**)).

^{1}H NMR (500 MHz, CDCl$_3$): δ 5.27 (d, *J* = 1.5 Hz, 1H), 5.17 – 5.16 (m, 1H), 4.63 – 4.59 (m, 1H), 2.23 – 2.12 (m, 3H), 2.04 (s, 3H), 1.81-1.75 (m, 2H), 1.66 – 1.00 (m, 12H), 0.97 (s, 3H), 0.93 (s, 9H), 0.83 (s, 3H), 0.15 (d, *J* = 5.0 Hz, 6H) ppm.

^{13}C NMR (126 MHz, CDCl$_3$): δ 171.2, 150.6, 141.3, 118.0, 108.8, 82.8, 51.3, 48.2, 42.5, 36.8, 34.9, 34.1, 31.7, 31.4, 27.6, 25.7, 23.5, 21.2, 20.7, 19.0, 18.0, 12.0, -4.2, -4.4 ppm.

HRMS (EI, *m/z*): calcd. for $(C_{27}H_{44}O_3Si + H)^+$: 445.3138; found: 445.3145.

5.3.24 *tert*-butyl((3*E*)-hexa-1,3-dien-1-yloxy)dimethylsilane (*Z*/*E* mixture) (S28)

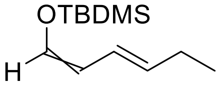

Following the general procedure **A** compound **S28** was obtained from hex-2-enal (5.0 mmol). The reaction mixture was passed through a plug (deactivated, neutral Al_2O_3 treated with 15 wt% of H_2O) using hexane as an eluent. The crude product was used for the next step without further purification (0.84 g of compound **S28** as a colorless oil (**Yield = 79%**, 16 h, *Z*/*E* ratio ~ 1.2:1)).

^{1}H NMR (400 MHz, CDCl$_3$): δ 6.53 (d, *J* = 11.5 Hz, 1H), 6.47 (d, *J* = 11.8 Hz, 1H), 5.97 – 5.78 (m, 2H + 1H), 5.68 (t, *J* = 11.3 Hz, 1H), 5.49 (dt, *J* = 15.2, 6.5 Hz, 1H), 5.19 (dt, *J* = 10.6, 7.4 Hz, 1H), 2.17 – 2.01 (m, 2H + 2H), 1.01 – 0.96 (m, 3H + 3H), 0.93 (s, 9H), 0.92 (s, 9H), 0.16 (s, 6H), 0.14 (s, 6H) ppm.

^{13}C NMR (100 MHz, CDCl$_3$): δ 144.6, 142.8, 131.0, 129.0, 124.9, 123.6, 113.6, 109.2, 25.8, 25.6, 21.0, 18.3, 14.3, 13.8, -5.3 ppm.

HRMS (EI, *m/z*): calcd. for $C_{12}H_{24}OSi$: 212.1596; found: 212.1602.

5.3.25 *tert*-butyl(((1*Z*,3*E*)-3,7-dimethylocta-1,3,6-trien-1-yl)oxy)dimethylsilane (S29)

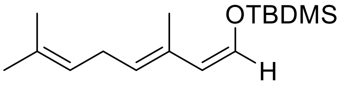

Following the general procedure **A** compound **S29** was obtained from (*E*)-3,7-dimethylocta-2,6-dienal (3.0 mmol). The reaction mixture was passed through a plug (deactivated, neutral Al_2O_3 treated with 15 wt% of H_2O) using hexane as an eluent. The crude product was used for the next step without further purification (0.79 g of compound **S29** as a colorless oil (**Yield = 100%**, 24 h).

^{1}H NMR (400 MHz, $CDCl_3$): δ 6.60 (d, *J* = 12.2 Hz, 1H), 5.74 (dd, *J* = 12.1, 4.7 Hz, 1H), 4.77 (d, *J* = 1.6 Hz, 1H), 4.69 (m, 1H), 2.16 – 2.14 (m, 2H), 1.76 – 1.69 (m, 6H), 1.62 (s, 3H), 0.93 (s, 9H), 0.16 (s, 6H) ppm.

^{13}C NMR (100 MHz, $CDCl_3$): δ 144.0, 142.2, 141.6, 139.8, 131.7, 129.3, 126.0, 125.0, 124.2, 123.1, 122.9, 118.3, 115.4, 112.4, 111.1, 110.4, 109.8, 33.0, 27.1, 26.6, 25.6, 20.6, 18.3, 17.7, -5.2 ppm.

HRMS (EI, *m/z*): decomposition

5.3.26 (*E*)-*tert*-butyldimethyl((2,6,6-trimethylcyclohex-2-en-1-ylidene)methoxy)silane (S30)

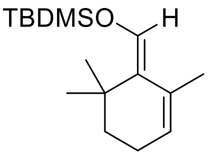

Following the general procedure **A** compound **S30** was obtained from 2,6,6-trimethylcyclohex-1-ene-1-carbaldehyde (3.0 mmol). The reaction mixture was passed through a plug (deactivated, neutral Al_2O_3 treated with 15 wt% of H_2O) using hexane as an eluent. The crude product was used for the next step without further purification (0.76 g of compound **S30** as a colorless oil (**Yield = 95%**, 24 h)).

^{1}H NMR (400 MHz, $CDCl_3$): δ 6.39 (s, 1H), 5.47 (t, *J* = 4.1 Hz, 1H), 2.09 (dt, *J* = 8.8, 4.2 Hz, 2H), 1.77 (m, 3H), 1.45 (t, *J* = 6.2 Hz, 2H), 1.26 (s, 6H), 0.99 (s, 9H), 0.21 (s, 6H).

^{13}C NMR (100 MHz, $CDCl_3$): δ 136.7, 131.0, 125.9, 123.3, 39.5, 33.2, 27.4, 25.7, 22.8, 20.6, 18.2, -5.4 ppm.

HRMS (EI, *m/z*): decomposition

5.3.27 (*E*)-*tert*-butyldimethyl((2-phenylbuta-1,3-dien-1-yl)oxy)silane (S31)

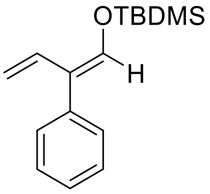

Following the general procedure **A** compound **S31** was obtained from 2-phenylbut-2-enal (3.0 mmol). The reaction mixture was passed through a plug (deactivated, neutral Al_2O_3 treated with 15 wt% of H_2O) using hexane as an eluent. The crude product was used for the next step without further purification (0.78 g of compound **S31** as a colorless oil (**Yield = 100%**, 24 h)).

^{1}H NMR (400 MHz, $CDCl_3$): δ 7.37 – 7.22 (m, 5H), 6.60 (s, 1H), 6.45 – 6.40 (m, 1H), 4.92 – 4.90 (m, 1H), 4.89-4.88 (m, 1H), 0.82 (s, 9H), 0.10 (s, 6H).

^{13}C NMR (100 MHz, $CDCl_3$): δ 141.7, 136.1, 135.2, 130.0, 127.7,126.4, 124.4, 111.0, 25.4, 18.1, -5.4 ppm.

HRMS (EI, *m/z*): calcd. for $C_{16}H_{24}OSi$: 260.1596; found: 260.1608.

5.4. *N*-aminopyridinium salts

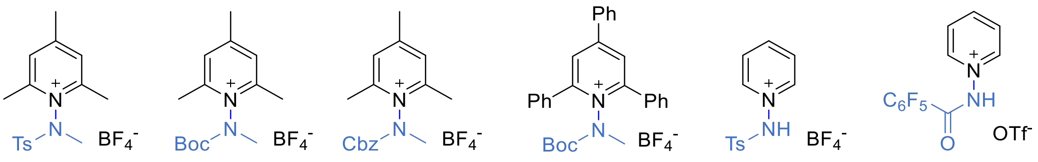

2a 2b 2c 2d 2f 2e

Compounds were prepared following the procedures described previously.[6]

5.5. Scope of visible-light mediated amidation

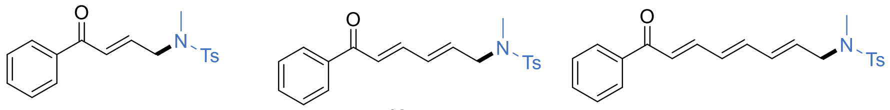

3a 19 20

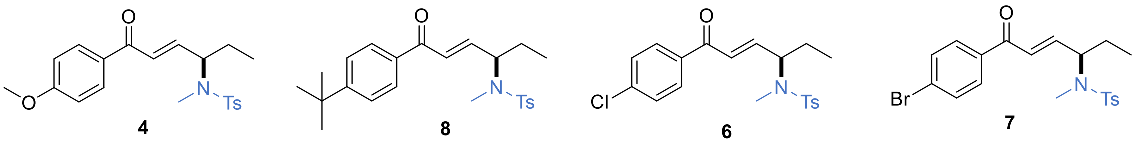

4 8 6 7

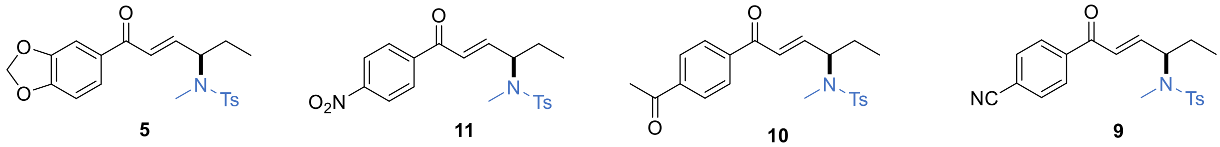

5 11 10 9

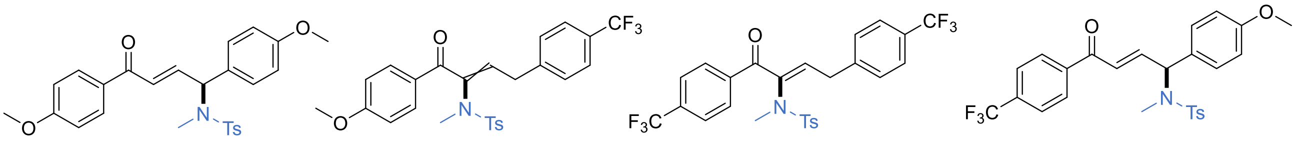

13a 14c 14b 13d

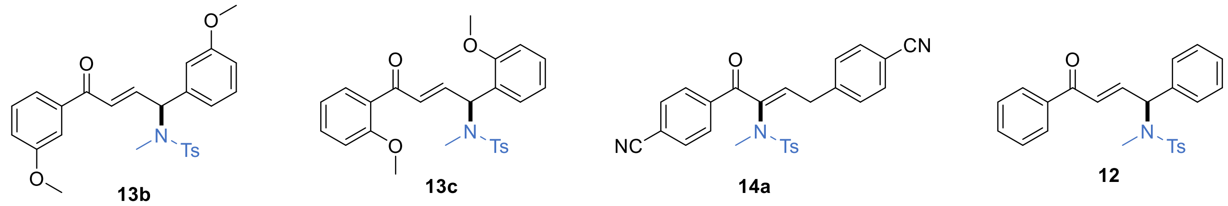

13b 13c 14a 12

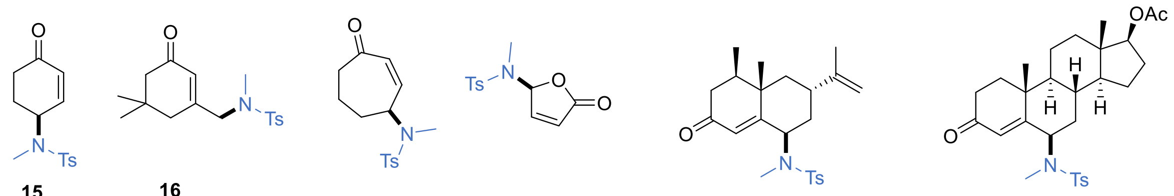

15 16 17 21 24 25

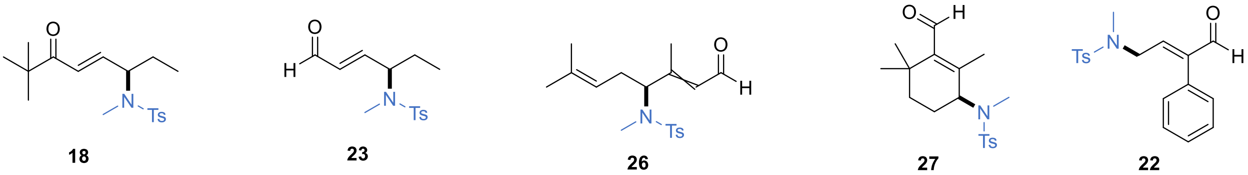

18 23 26 27 22

5.5.1 (*E*)-*N*,4-dimethyl-*N*-(4-oxo-4-phenylbut-2-en-1-yl)benzenesulfonamide (3a)

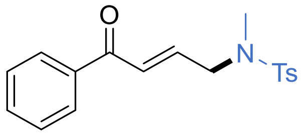

Following the general procedure **C** compound **3a** was obtained from enol **S1** (0.25 mmol) and *N*-aminopyridinium salt **2a** (0.33 mmol). The crude product was purified by column chromatography (20:80 AcOEt/Hexane) to afford 74 mg of compound **3a** as a colorless oil (**Yield = 90%**, 1 h).

^{1}H NMR (400 MHz, CDCl$_3$): δ 7.91 – 7.88 (m, 2H), 7.72 – 7.70 (m, 2H), 7.61 – 7.55 (m, 1H), 7.50 – 7.44 (m, 2H), 7.33 (d, *J* = 8.0 Hz, 2H), 7.05 (dt, *J* = 15.4, 1.7 Hz, 1H), 6.85 (dt, *J* = 15.4, 5.3 Hz, 1H), 3.93 (dd, *J* = 5.3, 1.6 Hz, 2H), 2.78 (s, 3H), 2.43 (s, 3H) ppm.
^{13}C NMR (100 MHz, CDCl$_3$): δ 189.8, 143.8, 141.8, 137.3, 134.4, 133.1, 129.9, 128.6, 128.6, 127.6, 127.4, 51.5, 35.2, 21.5 ppm.
HRMS (ESI, *m/z*): calcd. for ($C_{18}H_{19}NO_3S$ + Na)$^+$: 352.0983; found: 352.0984.
Elemental analysis (%): calcd. for $C_{18}H_{19}NO_3S$: C 65.63, H 5.81, N 4.25, S 9.73; found: C 65.57, H 5.85, N 4.21, S 9.50.

5.5.2 *tert*-butyl methyl(4-oxo-4-phenylbut-2-en-1-yl)carbamate (*E*/*Z* mixture) (3b)

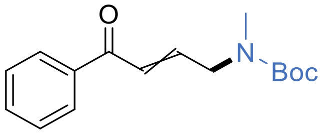

Following the general procedure **C** compound **3b** was obtained from enol **S1** (0.25 mmol) and *N*-aminopyridinium salt **2b** (0.33 mmol). The crude product was purified by column chromatography (20:80 AcOEt/Hexane) to afford 52 mg of compound **3b** as a colorless oil (**Yield = 76%**, 1 h, *E*/*Z* ratio ~ 1.2:1).

^{1}H NMR (500 MHz, CDCl$_3$): δ 7.92 (d, *J* = 7.5 Hz, 2H), 7.57 (t, *J* = 7.2 Hz, 1H), 7.47 (t, *J* = 7.6 Hz, 2H), 7.08 – 6.77 (m, 2H), 4.09 (s, 2H), 2.92 (s, 3H), 1.48 (s, 9H) ppm.
^{13}C NMR (125 MHz, CDCl$_3$): δ 190.5, 190.0, 155.5, 143.8, 137.5, 132.9, 128.6, 128.5, 128.3, 126.3, 125.2, 80.0, 50.5, 49.8, 34.5, 28.4 ppm.
HRMS (ESI, *m/z*): calcd. For ($C_{16}H_{21}NO_3$ + Na)$^+$: 298.1419; found: 298.1423.
Elemental analysis (%): calcd. For $C_{16}H_{21}NO_3$: C 69.79, H 7.69, N 5.09; found: C 69.65, H 7.70, N 5.18.

5.5.3 (*E*)-4-methyl-*N*-(4-oxo-4-phenylbut-2-en-1-yl)benzenesulfonamide (3f)

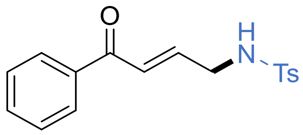

Following the general procedure **C** compound **3f** was obtained from enol **S1** (0.25 mmol) and *N*-aminopyridinium salt **2f** (0.33 mmol). The crude product was purified by column chromatography (20:80 AcOEt/Hexane) to afford 58 mg of compound **3f** as an off-white solid (**Yield = 74%**, 1 h).

^{1}H NMR (400 MHz, CDCl$_3$): δ 7.84 (d, *J* = 7.1 Hz, 2H), 7.78 (d, *J* = 8.3 Hz, 2H), 7.55 (t, *J* = 7.4 Hz, 1H), 7.42 (t, *J* = 7.7 Hz, 2H), 7.28 (d, *J* = 8.1 Hz, 2H), 7.01 (dt, *J* = 15.4, 1.8 Hz, 1H), 6.83 (dt, *J* = 15.4, 4.9 Hz, 1H), 5.30 (t, *J* = 6.3 Hz, 1H), 3.87 (ddd, *J* = 6.3, 4.9, 1.8 Hz, 2H), 2.37 (s, 3H) ppm.

^{13}C NMR (100 MHz, CDCl$_3$): δ 189.8, 143.9, 142.3, 137.2, 136.8, 133.1, 129.9, 128.6, 127.1, 126.4, 44.3, 21.5 ppm.

HRMS (ESI, *m/z*): calcd. for ($C_{17}H_{17}NO_3S$ + Na)$^+$: 338.0827; found: 338.0825.

Elemental analysis (%): calcd. for $C_{17}H_{17}NO_3S$: C 64.74, H 5.43, N 4.44, S 10.17; found: C 65.50, H 5.48, N 4.48, S 10.27.

m.p.: 98-96 °C.

5.5.4 (*E*)-2,3,4,5,6-pentafluoro-*N*-(4-oxo-4-phenylbut-2-en-1-yl)benzamide (3e)

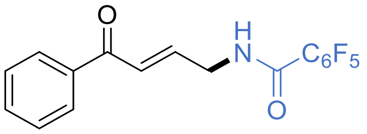

Following the general procedure **C** compound **3e** was obtained from enol **S1** (0.25 mmol) and *N*-aminopyridinium salt **2e** (0.33 mmol). The crude product was purified by column chromatography (20:80 AcOEt/Hexane) to afford 41 mg of compound **3e** as a white solid (**Yield = 46%**, 16 h).

^{1}H NMR (500 MHz, CDCl$_3$): δ 7.95 (d, *J* = 7.4 Hz, 2H), 7.66 (t, *J* = 7.4 Hz, 1H), 7.54 (t, *J* = 7.7 Hz, 2H), 7.15 (dt, *J* = 15.5, 2.0 Hz, 1H), 7.05 (dt, *J* = 15.5, 4.8 Hz, 1H), 6.83 (br s, 1H), 4.42 (t, *J* = 4.8 Hz, 2H) ppm.

^{13}C NMR (125 MHz, CDCl$_3$): 190.2, 157.5, 145.2 (m), 143.2 (m), 142.3, 141.4 (m), 138.7 (m), 137.1, 136.6 (m), 133.3, 128.7, 128.5, 126.3, 111.2 (m), 41.2 ppm.

^{19}F NMR (375 MHz, CDCl$_3$): δ -140.32–140.63 (m, 2F), -150.13 (tt, *J* = 20.8, 2.9 Hz, 1F), -159.55–159.84 (m, 2F).

HRMS (ESI, *m/z*): calcd. for ($C_{17}H_{10}NO_2F_5$ + Na)$^+$: 378.0529; found: 378.0526.

Elemental analysis (%) calcd. for $C_{17}H_{10}NO_2F_5$: C 57.47, H 2.84, N 3.94; found: C 57.75, H 2.85, N 3.87.

5.5.5 benzyl (*E*)-methyl(4-oxo-4-phenylbut-2-en-1-yl)carbamate (3c)

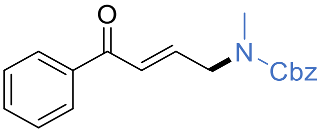

Following the general procedure **C** compound **3c** was obtained from enol **S1** (0.25 mmol) and *N*-aminopyridinium salt **2c** (0.33 mmol). The crude product was purified by column chromatography (20:80 AcOEt/Hexane) to afford 36 mg of compound **3c** as a colorless oil (**Yield = 46%**, 2 h).

^{1}H NMR (500 MHz, $CDCl_3$): δ 7.88 – 7.48 (m, 2H), 7.58 – 7.54 (m, 1H), 7.47 – 7.26 (m, 7H), 6.97 – 6.79 (m, 2H), 5.15 (s, 2H), 4.18 – 4.15 (m, 2H), 3.00 – 2.99 (m, 3H) ppm.

^{13}C NMR (125 MHz, $CDCl_3$): δ 143.0, 133.0, 132.9, 128.6, 128.5, 128.1, 128.0, 126.5, 125.7, 67.4, 50.4, 50.2, 36.4, 35.1, 34.3 ppm.

HRMS (ESI, *m/z*): calcd. for $(C_{19}H_{19}NO_3 + Na)^+$: 332.1263; found: 332.1268.

Analytical HPLC:

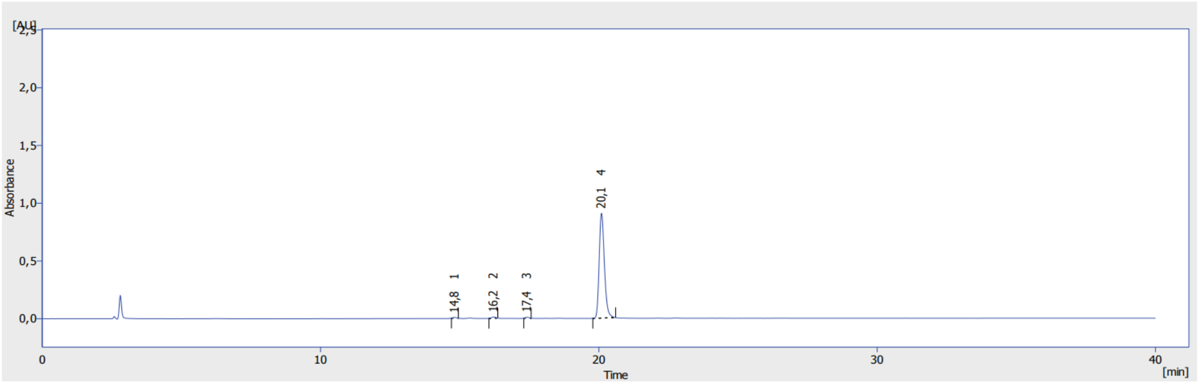

	Reten. Time [min]	Area [mAU.s]	Height [mAU]	Area [%]	Height [%]	W05 [min]	PDA Peak Purity	Compound Name	PDA Best Match Name	PDA Best Match
1	14,817	55,080	6,115	0,5	0,7	0,17	1000			
2	16,217	103,287	10,389	0,9	1,1	0,18	1000			
3	17,417	68,709	7,639	0,6	0,8	0,17	1000			
4	20,100	11794,221	907,218	98,1	97,4	0,22	879			
	Total	12021,296	931,362	100,0	100,0					

5.5.6 *N*,4-dimethyl-*N*-((2*E*,4*E*)-6-oxo-6-phenylhexa-2,4-dien-1-yl)benzenesulfonamide (19)

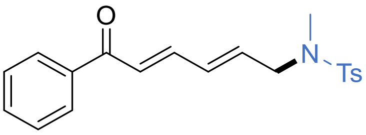

Following the general procedure **C** compound **19** was obtained from enol **S2** (0.25 mmol) and *N*-aminopyridinium salt **2a** (0.33 mmol). The crude product was purified by column chromatography (20:80 AcOEt/Hexane) to afford 36 mg of compound **19** as a yellow oil (**Yield = 41%**, 3 h).

^{1}H NMR (400 MHz, $CDCl_3$): δ 7.93 (d, *J* = 5.1 Hz, 2H), 7.69 (d, *J* = 8.3 Hz, 2H), 7.56 (t, *J* = 7.4 Hz, 1H), 7.47 (t, *J* = 7.5 Hz, 2H), 7.39 – 7.30 (m, 3H), 6.95 (d, *J* = 15.1 Hz, 1H), 6.44 (dd,

J = 14.9, 11.3 Hz, 1H), 6.04 (dt, J = 15.2, 6.2 Hz, 1H), 3.80 (d, J = 6.1 Hz, 2H), 2.71 (s, 3H), 2.44 (s, 3H) ppm.

^{13}C NMR (100 MHz, CDCl$_3$): δ 190.4, 143.7, 142.9, 137.0, 137.3, 134.5, 132.9, 131.9, 129.8, 128.6, 128.4, 127.4, 126.2, 51.9, 34.8, 21.5 ppm.

HRMS (ESI, *m/z*): calcd. For ($C_{20}H_{21}NO_3S$ + Na)$^+$: 378.1140; found: 378.1136.

Elemental analysis (%) calcd. For $C_{20}H_{21}NO_3S$: C 67.58, H 5.95, N 3.94, S 9.02; found: C 67.35, H 5.94, N 4.05, S 8.99.

5.5.7 *N*,4-dimethyl-*N*-((2*E*,4*E*,6*E*)-8-oxo-8-phenylocta-2,4,6-trien-1-yl)benzenesulfonamide (20)

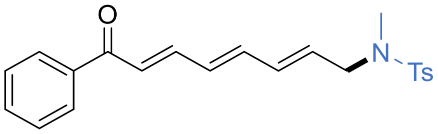

Following the general procedure **C** compound **20** was obtained from enol **S3** (0.25 mmol) and *N*-aminopyridinium salt **2a** (0.33 mmol). The crude product was purified by column chromatography (5:25:70 AcOEt/DCM/Hexane) to afford 8 mg of compound **20** as a yellow oil (**Yield = 10%**, 1 h).

^{1}H NMR (400 MHz, CDCl$_3$): δ 7.93-7.92 (m, 2H), 7.69 – 7.66 (m, 2H), 7.56 – 7.53 (m, 1H), 7.48-7.45 (m, 2H), 7.42 (ddd, J = 14.9, 11.3, 0.5 Hz, 1H), 7.33 – 7.31 (m, 2H), 6.97 (d, J = 14.9 Hz, 1H), 6.60 (dd, J = 14.8, 10.8 Hz, 1H), 6.41 (dd, J = 14.9, 11.3 Hz, 1H), 6.31 – 6.27 (m, 1H), 5.79 (dt, J = 15.11, 6.5 Hz, 1H), 3.73 (d, J = 6.4 Hz, 2H), 2.68 (s, 3H), 2.43 (s, 3H) ppm.

^{13}C NMR (100 MHz, CDCl$_3$): δ 190.4, 144.1, 143.5, 140.3, 138.1, 134.5, 133.2, 132.7, 132.3, 131.3, 129.7, 128.6, 128.3, 127.5, 127.3, 125.8, 52.0, 34.6, 21.5 ppm.

HRMS (ESI, *m/z*): calcd. for ($C_{22}H_{23}NO_3S$ + Na)$^+$: 404.1296, found: 404.1295.

Analytical HPLC:

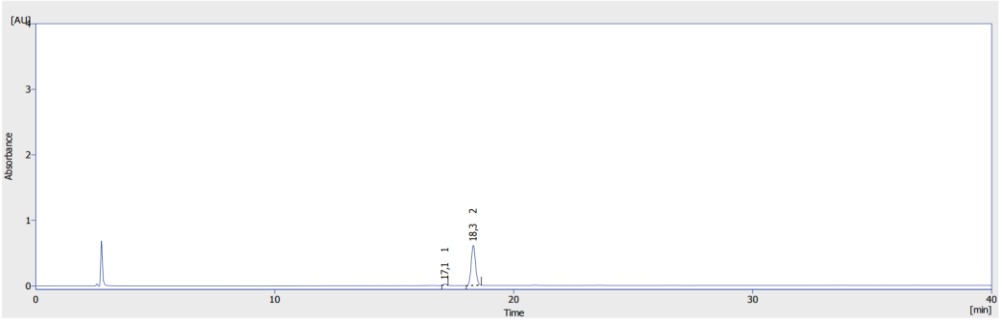

	Reten. Time [min]	Area [mAU.s]	Height [mAU]	Area [%]	Height [%]	W05 [min]	PDA Peak Purity	Compound Name	PDA Best Match Name	PDA Best Match
1	17,133	69,638	7,824	1,8	2,5	0,17	1000			
2	18,317	3851,998	304,984	98,2	97,5	0,22	760			
	Total	3921,636	312,808	100,0	100,0					

5.5.8 (*E*)-*N*-(6-(4-methoxyphenyl)-6-oxohex-4-en-3-yl)-*N*,4-dimethylbenzenesulfonamide (4)

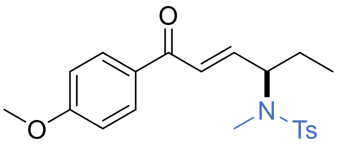

Following the general procedure **C** compound **4** was obtained from enol **S4** (0.25 mmol) and *N*-aminopyridinium salt **2a** (0.33 mmol). The crude product was purified by column chromatography (10:90 AcOEt/Hexane) to afford 73 mg of compound **4** as a colorless oil (**Yield = 75 %**, 1h).

^{1}H NMR (500 MHz, $CDCl_3$): δ 7.81 (d, *J* = 8.8 Hz, 2H), 7.71 (d, *J* = 8.1 Hz, 2H), 7.25 (d, *J* = 8.0 Hz, 2H), 6.93 (d, *J* = 8.8 Hz, 2H), 6.82-6.76 (dd, *J* = 15.7, 1.0 Hz, 1H), 6.66 (dd, *J* = 15.4, 5.9 Hz, 1H), 4.60 (q, *J* = 6.8 Hz, 1H), 3.88 (s, 3H), 2.76 (s, 3H), 2.34 (s, 3H), 1.60 (m, 2H), 0.94 (t, *J* = 7.3 Hz, 3H) ppm.

^{13}C NMR (100 MHz, $CDCl_3$): δ 187.9, 163.6, 143.3, 143.1, 136.9, 130.8, 130.2, 129.6, 127.2, 126.6, 113.8, 59.8, 55.5, 28.6, 24.8, 21.4, 10.8 ppm.

HRMS (ESI, *m/z*): calcd. for $(C_{21}H_{25}NO_4S + Na)^+$: 410.1402, found: 410.1407.

Analytical HPLC:

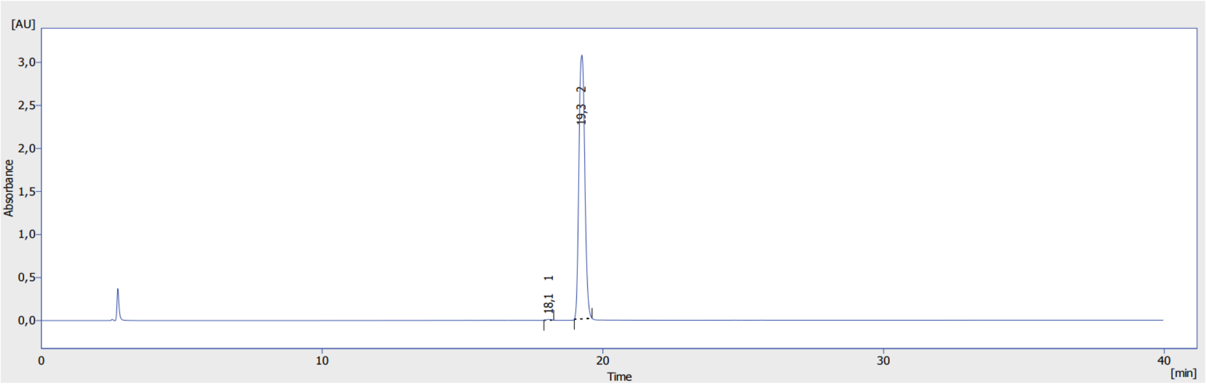

	Reten. Time [min]	Area [mAU.s]	Height [mAU]	Area [%]	Height [%]	W05 [min]	PDA Peak Purity	Compound Name	PDA Best Match Name	PDA Best Match
1	18,083	108,434	10,072	0,2	0,3	0,20	1000			
2	19,250	44875,850	3065,998	99,8	99,7	0,23	811			
	Total	44984,284	3076,070	100,0	100,0					

5.5.9 (*E*)-*N*-(6-(4-(*tert*-butyl)phenyl)-6-oxohex-4-en-3-yl)-*N*,4-dimethylbenzenesulfonamide (8)

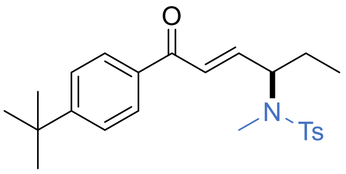

Following the general procedure **C** compound **8** was obtained from enol **S5** (0.25 mmol) and *N*-aminopyridinium salt **2a** (0.33 mmol). The crude product was purified by column chromatography (10:90 AcOEt/Hexane) to afford 74 mg of compound **8** as a colorless oil (Yield = 72 %, 1 h).

^{1}H NMR (400 MHz, $CDCl_3$): δ 7.75-7.68 (m, 4H), 7.49-7.42 (m, 2H), 7.26-7.22 (d, *J* = 8.7 Hz, 2H), 6.77 (dd, *J* = 15.5, 1.2 Hz, 1H), 6.66 (dd, *J* = 15.5, 5.8 Hz, 1H), 4.60 (m, 1H), 2.75 (s, 3H), 2.33 (s, 3H), 1.69-1.57 (m, 2H), 1.35 (s, 9H), 0.94 (t, *J* = 7.4 Hz, 3H) ppm.

^{13}C NMR (100 MHz, $CDCl_3$): δ 189.2, 156.9, 143.5, 143.3, 137.0, 134.7, 129.7, 128.5, 127.2, 126.9, 125.5, 59.8, 35.1, 31.1, 28.7, 24.9, 21.4, 10.8 ppm.

HRMS (ESI, *m/z*): calcd. for $(C_{24}H_{31}NO_3S + Na)^+$: 436.1922, found: 436.1934.

Analytical HPLC:

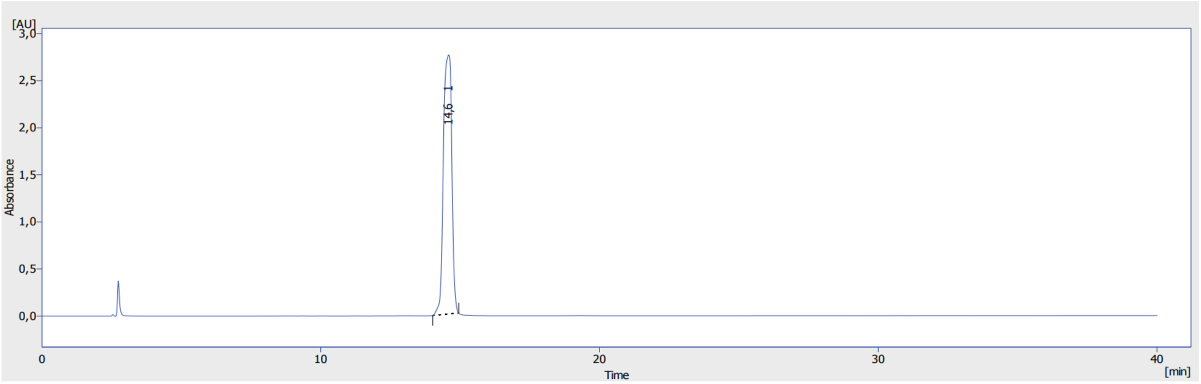

	Reten. Time [min]	Area [mAU.s]	Height [mAU]	Area [%]	Height [%]	W05 [min]	PDA Peak Purity	Compound Name	PDA Best Match Name	PDA Best Match
1	14,583	57649,301	2752,419	100,0	100,0	0,35	797			
	Total	57649,301	2752,419	100,0	100,0					

5.5.10 (*E*)-*N*-(6-(4-chlorophenyl)-6-oxohex-4-en-3-yl)-*N*,4-dimethylbenzenesulfonamide (6)

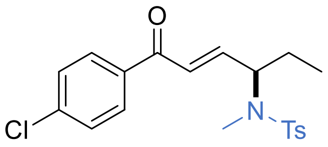

Following the general procedure **C** compound **6** was obtained from enol **S6** (0.25 mmol) and *N*-aminopyridinium salt **2a** (0.33 mmol). The crude product was purified by column chromatography (10:90 AcOEt/Hexane) to afford **68** mg of compound **6** as a yellow oil (**Yield = 69 %**, 1 h).

^{1}H NMR (400 MHz, $CDCl_3$): δ 7.75 – 7.67 (m, 4H), 7.44 – 7.39 (m, 2H), 7.26-7.24 (m 2H), 6.76 (dd, *J* = 15.5, 1.0 Hz, 1H), 6.69 (dd, *J* = 15.5, 5.3 Hz, 1H), 4.65 – 4.53 (m, 1H), 2.74 (s, 3H), 2.35 (s, 3H), 1.68 – 1.55 (m, 2H), 0.93 (t, *J* = 7.4 Hz, 3H) ppm.

^{13}C NMR (100 MHz, $CDCl_3$): δ 188.5, 144.8, 143.4, 139.6, 136.9, 135.6, 129.9, 129.7, 128.9, 127.2, 126.3, 59.7, 28.7, 24.7, 21.4, 10.8 ppm.

HRMS (ESI, *m/z*): calcd. for $(C_{20}H_{22}ClNO_3S + Na)^+$: 414.0907, found: 414.0913.

Analytical HPLC:

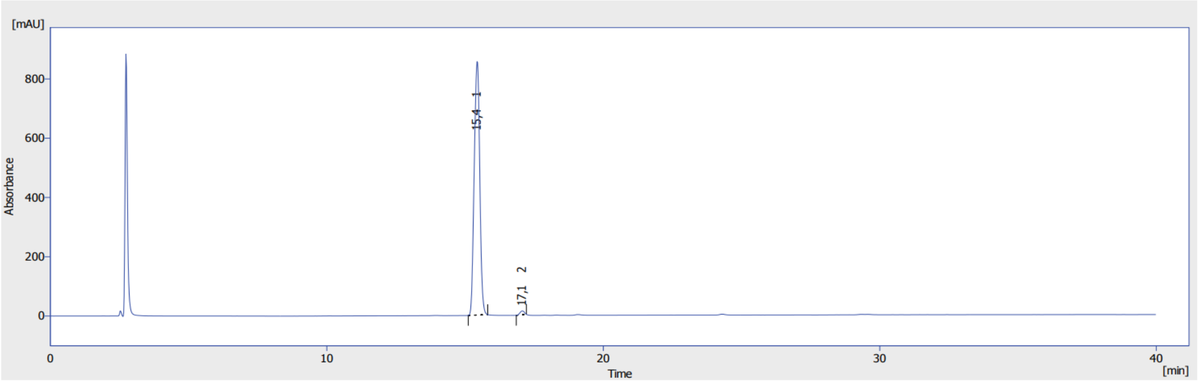

	Reten. Time [min]	Area [mAU.s]	Height [mAU]	Area [%]	Height [%]	W05 [min]	PDA Peak Purity	Compound Name	PDA Best Match Name	PDA Best Match
1	15,433	11935,971	855,053	98,9	98,5	0,25	896			
2	17,067	134,645	12,875	1,1	1,5	0,18	1000			
	Total	12070,616	867,929	100,0	100,0					

5.5.11 (*E*)-*N*-(6-(4-bromophenyl)-6-oxohex-4-en-3-yl)-*N*,4-dimethylbenzenesulfonamide (7)

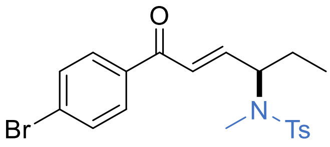

Following the general procedure **C** compound **7** was obtained from enol **S7** (0.25 mmol) and *N*-aminopyridinium salt **2a** (0.33 mmol). The crude product was purified by column chromatography (10:90 AcOEt/Hexane) to afford **72** mg of compound **7** as a yellow oil (**Yield = 66 %**, 1 h).

^{1}H NMR (400 MHz, $CDCl_3$): δ 7.73 – 7.67 (m, 2H), 7.67 – 7.62 (m, 2H), 7.61 – 7.56 (m, 2H), 7.26-7.24 (m, 2H), 6.78 – 6.72 (dd, *J* = 15.6, 0.6 Hz, 1H), 6.69 (dd, *J*= 15.5, 5.0 Hz, 1H), 4.65 – 4.55 (m, 1H), 2.74 (s, 3H), 2.35 (s, 3H), 1.67 – 1.56 (m, 2H), 0.93 (t, *J* = 7.0 Hz, 3H) ppm.

^{13}C NMR (100 MHz, $CDCl_3$): δ 188.7, 144.9, 143.4, 136.9, 136.0, 131.9, 130.0, 129.7, 128.2, 127.2, 126.3, 59.7, 28.7, 24.7, 21.4, 10.8 ppm.

HRMS (ESI, *m/z*): calcd. for $(C_{20}H_{22}BrNO_3S + H)^+$: 436.0582, found: 436.0591.

Analytical HPLC:

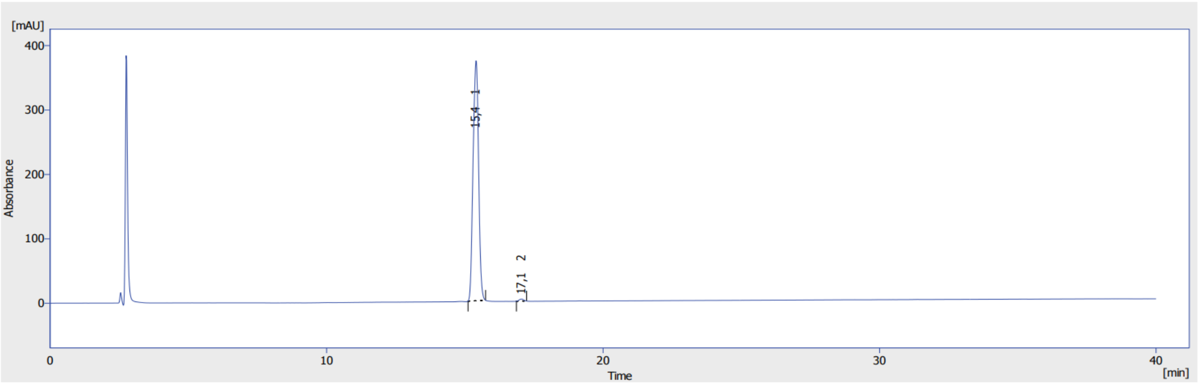

	Reten. Time [min]	Area [mAU.s]	Height [mAU]	Area [%]	Height [%]	W05 [min]	PDA Peak Purity	Compound Name	PDA Best Match Name	PDA Best Match
1	15,400	5163,452	372,508	99,3	99,2	0,23	948			
2	17,050	35,324	3,166	0,7	0,8	0,20	1000			
	Total	5198,776	375,674	100,0	100,0					

5.5.12 (*E*)-*N*-(6-(benzo[*d*][1,3]dioxol-5-yl)-6-oxohex-4-en-3-yl)-*N*,4-dimethylbenzenesulfonamide (5)

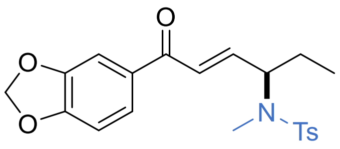

Following the general procedure **C** compound **5** was obtained from enol **S8** (0.25 mmol) and *N*-aminopyridinium salt **2a** (0.33 mmol). The crude product was purified by column chromatography (20:80 AcOEt/Hexane) to afford 71 mg of compound **5** as a colorless oil (**Yield = 71%**, 1 h).

^{1}H NMR (500 MHz, $CDCl_3$): δ 7.71 (d, *J* = 8.2 Hz, 2H), 7.38 (dd, *J* = 8.5, 1.4 Hz, 1H), 7.32 – 7.27 (m, 2H), 7.25 (s, 1H), 6.84 (d, *J* = 8.1 Hz, 1H), 6.73 (d, *J* = 15.1 Hz, 1H), 6.66 (dd, *J* = 15.5, 5.4 Hz, 1H), 6.06 (s, 2H), 4.64 – 4.55 (m, 1H), 2.75 (s, 3H), 2.36 (s, 3H), 1.66 – 1.54 (m, 2H), 0.94 (t, *J* = 7.5 Hz, 3H) ppm.

^{13}C NMR (100 MHz, $CDCl_3$): δ 187.5, 151.9, 148.3, 143.5, 137.0, 132.1, 129.7, 127.2, 126.5, 124.9, 108.3, 107.8, 101.9, 59.8, 28.7, 24.8, 21.4, 10.8 ppm.

HRMS (ESI, *m/z*): calcd. for $(C_{21}H_{23}NO_5S + Na)^+$: 424.1195, found: 424.1199.

Analytical HPLC:

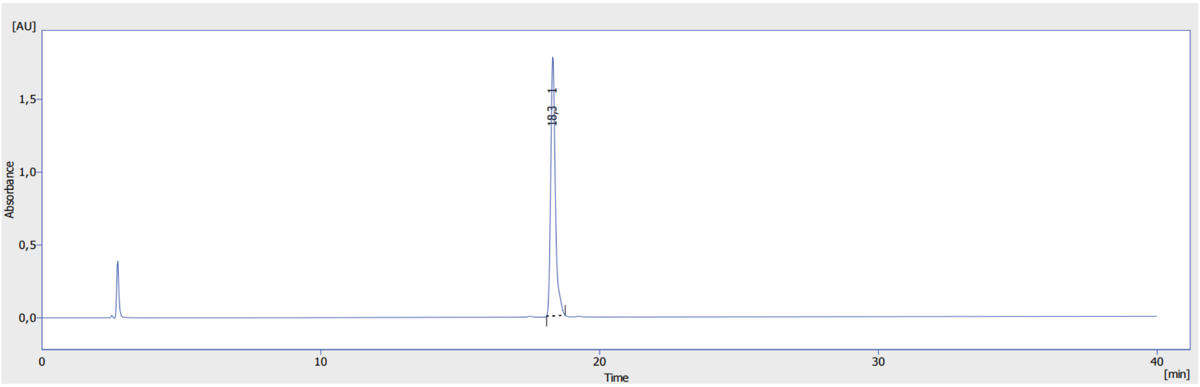

	Reten. Time [min]	Area [mAU.s]	Height [mAU]	Area [%]	Height [%]	W05 [min]	PDA Peak Purity	Compound Name	PDA Best Match Name	PDA Best Match
1	18,317	20529,150	1777,208	100,0	100,0	0,18	785			
	Total	20529,150	1777,208	100,0	100,0					

5.5.13 (*E*)-*N*,4-dimethyl-*N*-(6-(4-nitrophenyl)-6-oxohex-4-en-3-yl)benzenesulfonamide (11)

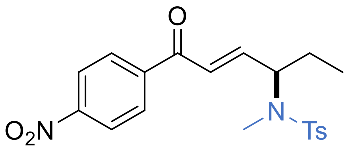

Following the general procedure **C** compound **11** was obtained from enol **S9** (0.25 mmol) and *N*-aminopyridinium salt **2a** (0.33 mmol). The crude product was purified by column chromatography (5:25:70 AcOEt/DCM/Hexane) to afford 81 mg of compound **11** as a yellow oil (**Yield = 80%**, 4 h).

^{1}H NMR (500 MHz, $CDCl_3$): δ 8.30 (d, *J* = 8.9 Hz, 2H), 7.95 (d, *J* = 8.9 Hz, 2H), 7.72 (d, *J* = 8.2 Hz, 2H), 7.29 (d, *J* = 7.9 Hz, 2H), 6.87 – 6.82 (dd, *J* = 15.6, 0.9 Hz, 1H), 6.79 (dd, *J* = 15.5, 5.0 Hz, 1H), 4.64 (dt, *J* = 8.2, 5.7 Hz, 1H), 2.76 (s, 3H), 2.38 (s, 3H), 1.66 (m, 2H), 0.92 (t, *J* = 7.4 Hz, 3H) ppm.

^{13}C NMR (100 MHz, $CDCl_3$): δ 188.3, 150.2, 146.7, 143.5, 142.0, 136.8, 129.7, 129.5, 127.2, 126.1, 123.8, 59.7, 28.7, 24.6, 21.5, 10.8 ppm.

HRMS (ESI, *m/z*): calcd. for $(C_{20}H_{22}N_2O_5S + Na)^+$: 425.1147, found: 425.1151.

Analytical HPLC:

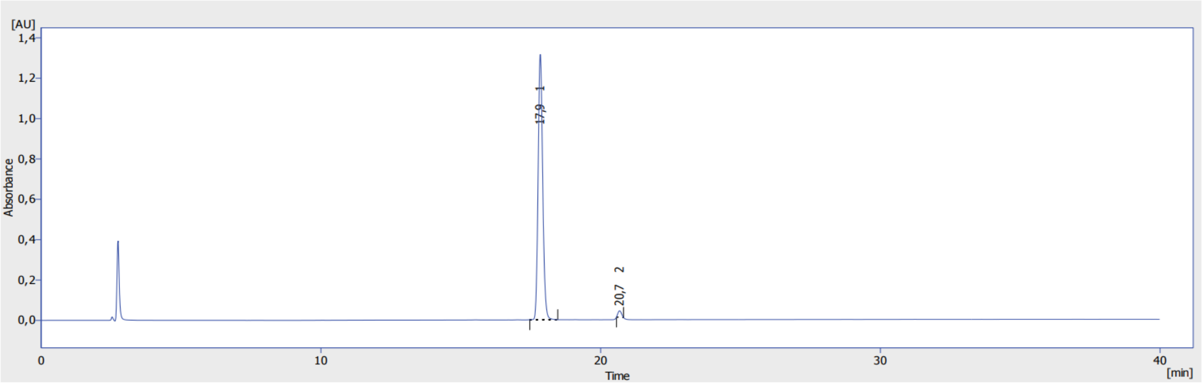

	Reten. Time [min]	Area [mAU.s]	Height [mAU]	Area [%]	Height [%]	W05 [min]	PDA Peak Purity	Compound Name	PDA Best Match Name	PDA Best Match
1	17,850	15011,773	1313,766	98,2	97,7	0,18	820			
2	20,683	270,156	31,579	1,8	2,3	0,17	1000			
	Total	15281,930	1345,345	100,0	100,0					

5.5.14 (*E*)-*N*-(6-(4-acetylphenyl)-6-oxohex-4-en-3-yl)-*N*,4-dimethylbenzenesulfonamide (10)

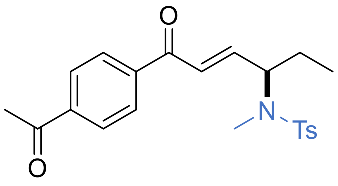

Following the general procedure **C** compound **10** was obtained from enol **S10** (0.15 mmol) and *N*-aminopyridinium salt **2a** (0.2 mmol). The crude product was purified by column chromatography (30:70 Et_2O/Hexane) to afford 34 mg of compound **10** as a yellow oil (**Yield = 67%**, 1 h).

^{1}H NMR (400 MHz, $CDCl_3$): δ 8.03 – 7.98 (m, 2H), 7.87 – 7.81 (m, 2H), 7.72 – 7.66 (m, 2H), 7.26 – 7.23 (m, 2H), 6.80 (dd, *J* = 15.5, 1.1 Hz, 1H), 6.71 (dd, *J* = 15.5, 5.4 Hz, 1H), 4.68 – 4.52 (m, 1H), 2.75 (s, 3H), 2.64 (s, 3H), 2.34 (s, 3H), 1.69 – 1.54 (m, 2H), 0.92 (t, *J* = 7.4 Hz, 3H) ppm.

^{13}C NMR (100 MHz, $CDCl_3$): δ 197.3, 189.3, 145.4, 143.4, 140.6, 140.1, 136.9, 129.7, 128.7, 128.4, 127.2, 126.6, 59.8, 28.7, 26.8, 24.7, 21.4, 10.8 ppm.

HRMS (ESI, *m/z*): calcd. for $(C_{22}H_{25}NO_4S + Na)^+$: 422.1402, found:422.1404.

Analytical HPLC:

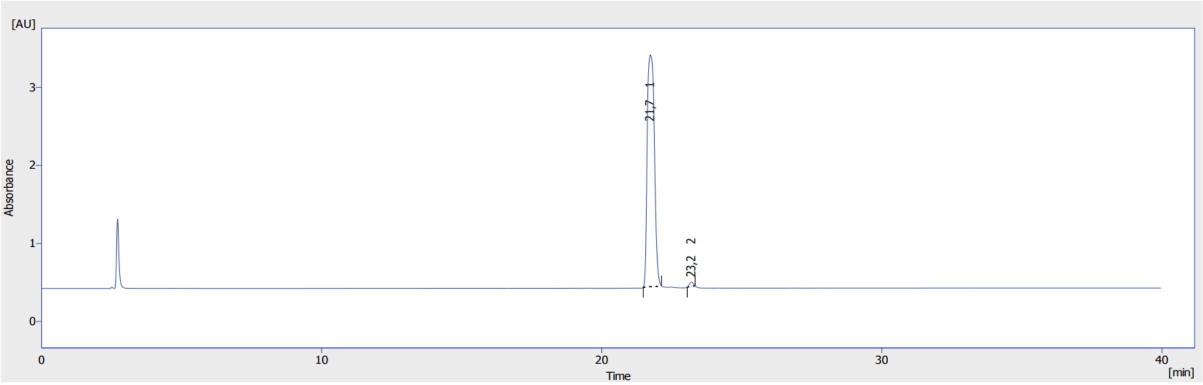

	Reten. Time [min]	Area [mAU.s]	Height [mAU]	Area [%]	Height [%]	W05 [min]	PDA Peak Purity	Compound Name	PDA Best Match Name	PDA Best Match
1	21,733	52725,117	2971,057	99,0	98,1	0,30	820			
2	23,200	522,094	56,156	1,0	1,9	0,18	999			
	Total	53247,211	3027,213	100,0	100,0					

5.5.15 (*E*)-*N*-(6-(4-cyanophenyl)-6-oxohex-4-en-3-yl)-*N*,4-dimethylbenzenesulfonamide (9)

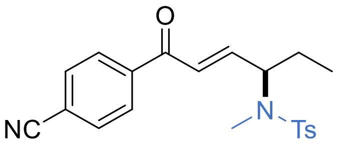

Following the general procedure **C** compound **9** was obtained from enol **S11** (0.25 mmol) and *N*-aminopyridinium salt **2a** (0.33 mmol). The crude product was purified by column chromatography (20:80 AcOEt/Hexane) to afford 58 mg of compound **9** as a colorless oil (**Yield = 60%**, 1 h).

^{1}H NMR (500 MHz, CDCl$_3$): δ 7.89 (d, *J* = 8.3 Hz, 2H), 7.76 (d, *J* = 8.3 Hz, 2H), 7.71 (d, *J* = 8.2 Hz, 2H), 7.28 (d, *J* = 7.8 Hz, 2H), 6.83 (d, *J* = 15.6 Hz, 1H), 6.77 (dd, *J* = 15.5, 5.0 Hz, 1H), 4.62 (q, *J* = 7.0, 6.1 Hz, 1H), 2.75 (s, 3H), 2.38 (s, 3H), 1.64 (m, 2H), 0.92 (t, *J* = 7.3 Hz, 3H) ppm.

^{13}C NMR (100 MHz, CDCl$_3$): δ 188.4, 146.4, 143.5, 140.5, 136.8, 132.4, 129.7, 128.9, 127.2, 126.0, 117.8, 116.3, 59.7, 28.6, 24.6, 21.4, 10.8 ppm.

HRMS (ESI, *m/z*): calcd. for $(C_{21}H_{22}N_2O_3S + Na)^+$: 405.1249, found: 405.1254.

Analytical HPLC:

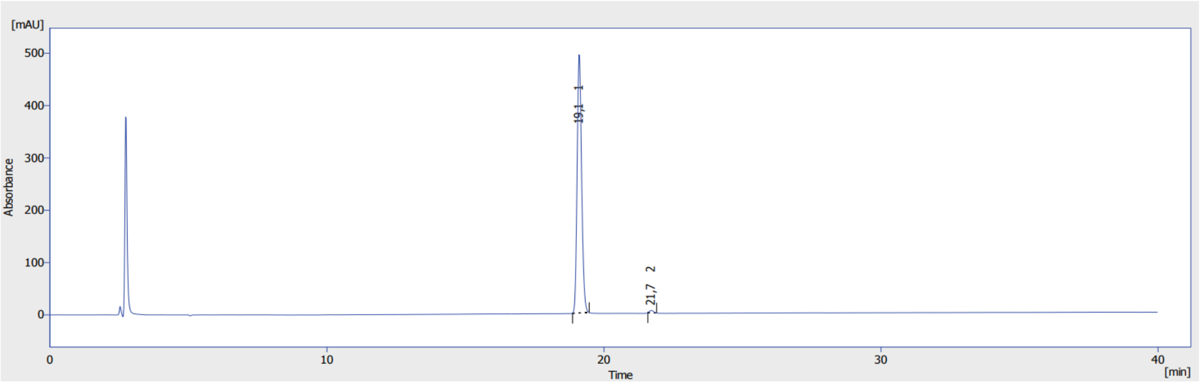

	Reten. Time [min]	Area [mAU.s]	Height [mAU]	Area [%]	Height [%]	W05 [min]	PDA Peak Purity	Compound Name	PDA Best Match Name	PDA Best Match
1	19,100	5559,421	493,409	99,2	99,1	0,18	941			
2	21,700	44,909	4,565	0,8	0,9	0,18	1000			
	Total	5604,331	497,975	100,0	100,0					

5.5.16 (*E*)-*N*-(1,4-bis(4-methoxyphenyl)-4-oxobut-2-en-1-yl)-N,4-dimethylbenzenesulfonamide (13a)

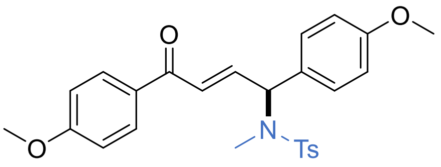

Following the general procedure **C** compound **13a** was obtained from enol **S15** (0.25 mmol) and *N*-aminopyridinium salt **2a** (0.33 mmol). The crude product was purified by column chromatography (15:20:65 AcOEt/DCM/Hexane) to afford 82 mg of compound **13a** as a colorless oil (**Yield = 71%**, 1 h).

^{1}H NMR (400 MHz, CDCl$_3$): δ 7.84 (d, *J* = 8.9 Hz, 2H), 7.72 (d, *J* = 8.2 Hz, 2H), 7.24 (d, *J* = 8.0 Hz, 2H), 7.15 (d, *J* = 8.5 Hz, 2H), 7.02 (dd, *J* = 15.4, 6.2 Hz, 1H), 6.95 (d, *J* = 8.9 Hz, 2H), 6.91 (dd, *J* = 15.4, 1.4 Hz, 1H), 6.84 (d, *J* = 8.8 Hz, 2H), 5.92 (d, *J* = 6.0 Hz, 1H), 3.89 (s, 3H), 3.80 (s, 3H), 2.65 (s, 3H), 2.33 (s, 3H) ppm.

^{13}C NMR (100 MHz, CDCl$_3$): δ 187.6, 163.7, 159.5, 143.4, 141.6, 136.8, 130.9, 130.2, 129.7, 129.5, 128.8, 127.9, 127.3, 114.1, 113.9, 61.0, 55.5, 55.3, 30.0, 21.4 ppm.

HRMS (ESI, *m/z*): calcd. For ($C_{26}H_{27}NO_5S$ + Na)$^+$: 488.1508, found: 488.1511.

Analytical HPLC:

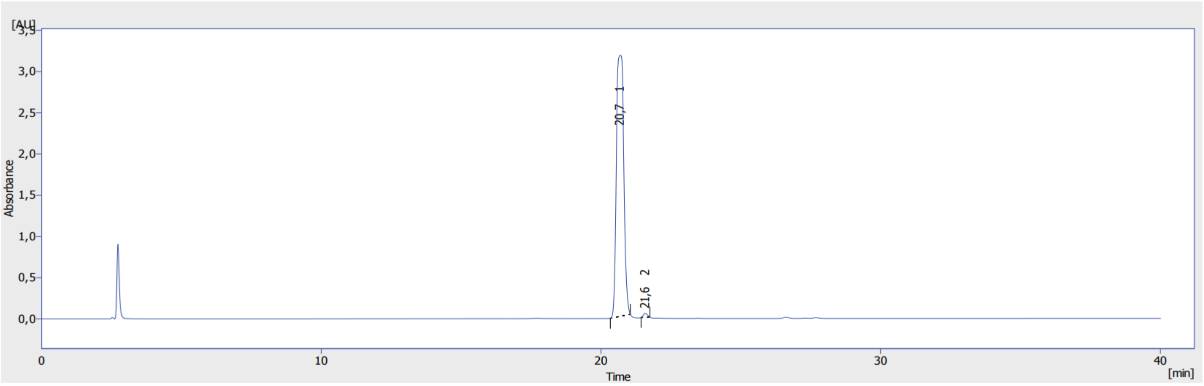

	Reten. Time [min]	Area [mAU.s]	Height [mAU]	Area [%]	Height [%]	W05 [min]	PDA Peak Purity	Compound Name	PDA Best Match Name	PDA Best Match
1	20,683	58010,912	3165,692	99,2	98,5	0,30	797			
2	21,583	486,591	47,542	0,8	1,5	0,18	997			
	Total	58497,503	3213,234	100,0	100,0					

5.5.17 (*Z*)-*N*-(1-(4-methoxyphenyl)-1-oxo-4-(4-(trifluoromethyl)phenyl)but-2-en-2-yl)-*N*,4-dimethylbenzenesulfonamide (14c)

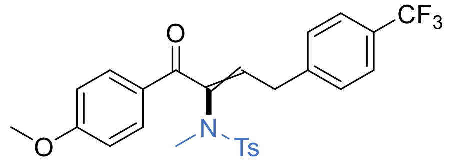

Following the general procedure **C** compound **14c** was obtained from enol **S12** (0.25 mmol) and *N*-aminopyridinium salt **2a** (0.33 mmol). The crude product was purified by column chromatography (5:25:70 AcOEt/DCM/Hexane) to afford 75 mg of compound **14c** as a yellow solid (**Yield = 60%**, *E/Z* ~1:12, 1 h). (Inseparable by column chromatography).

^{1}H NMR (400 MHz, CDCl$_3$): (major isomer) δ 7.86 (d, *J* = 8.8 Hz, 2H), 7.67 (d, *J* = 8.2 Hz, 2H), 7.49 (d, *J* = 8.2 Hz, 2H), 7.40 (d, *J* = 8.2 Hz, 2H), 7.24 (d, *J* = 8.0 Hz, 2H), 6.94 (d, *J* = 8.8 Hz, 2H), 6.64 (t, *J* = 6.9 Hz, 1H), 3.89 (s, 3H), 3.78 (d, *J* = 6.8 Hz, 2H), 3.14 (s, 3H), 2.36 (s, 3H) ppm.

^{13}C NMR (100 MHz, CDCl$_3$): (major isomer) δ 194.9, 163.8, 143.9, 140.6, 140.1, 136.7, 130.4, 129.7, 129.4, 127.4, 127.0, 126.6, 125.4, 125.3, 113.8, 55.5, 37.9, 37.6, 21.4 ppm.

HRMS (ESI, *m/z*): calcd. for $(C_{26}H_{24}F_3NO_4S + Na)^+$: 526.1276, found: 526.1271.

Analytical HPLC: major isomer

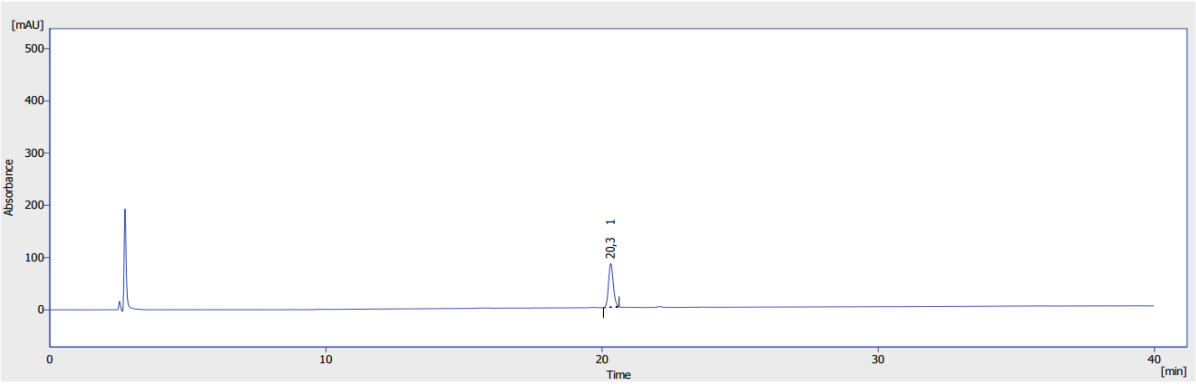

	Reten. Time [min]	Area [mAU.s]	Height [mAU]	Area [%]	Height [%]	W05 [min]	PDA Peak Purity	Compound Name	PDA Best Match Name	PDA Best Match
1	20,317	989,894	83,526	100,0	100,0	0,20	996			
	Total	989,894	83,526	100,0	100,0					

m.p.: 137-140 °C.

5.5.18 (*Z*)-*N*,4-dimethyl-*N*-(1-oxo-1,4-bis(4-(trifluoromethyl)phenyl)but-2-en-2-yl) benzenesulfonamide (14b)

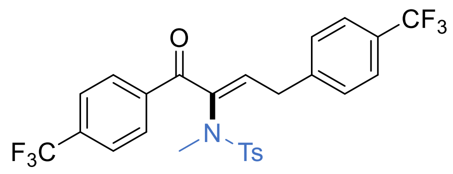

Following the general procedure **C** compound **14b** was obtained from enol **S13** (0.25 mmol) and *N*-aminopyridinium salt **2a** (0.33 mmol). The crude product was purified by column chromatography (20:80 AcOEt/Hexane) to afford 83 mg of compound **14b** as a yellow solid (**Yield = 64%**, 1h).

^{1}H NMR (400 MHz, $CDCl_3$): δ 8.02 (d, *J* = 8.1 Hz, 2H), 7.75 (d, *J* = 8.2 Hz, 2H), 7.67 (d, *J* = 8.2 Hz, 2H), 7.48 (d, *J* = 8.2 Hz, 2H), 7.34 (d, *J* = 8.2 Hz, 2H), 7.24 (d, *J* = 8.0 Hz, 2H), 6.63 (t, *J* = 6.8 Hz, 1H), 3.94 (d, *J* = 6.8 Hz, 2H), 3.12 (s, 3H), 2.35 (s, 3H) ppm.

^{13}C NMR (100 MHz, $CDCl_3$): δ 195.5, 144.0, 140.6, 140.1, 138.9, 136.5, 134.9, 134.7, 130.4, 129.8, 128.5, 127.4, 126.7, 125.8, 125.5, 125.4, 124.6, 122.9, 122.4, 38.6, 37.4, 21.4 ppm.

HRMS (ESI, *m/z*): calcd. for $(C_{26}H_{21}F_6NO_3S + H)^+$: 542.1225, found: 542.1226.

Analytical HPLC:

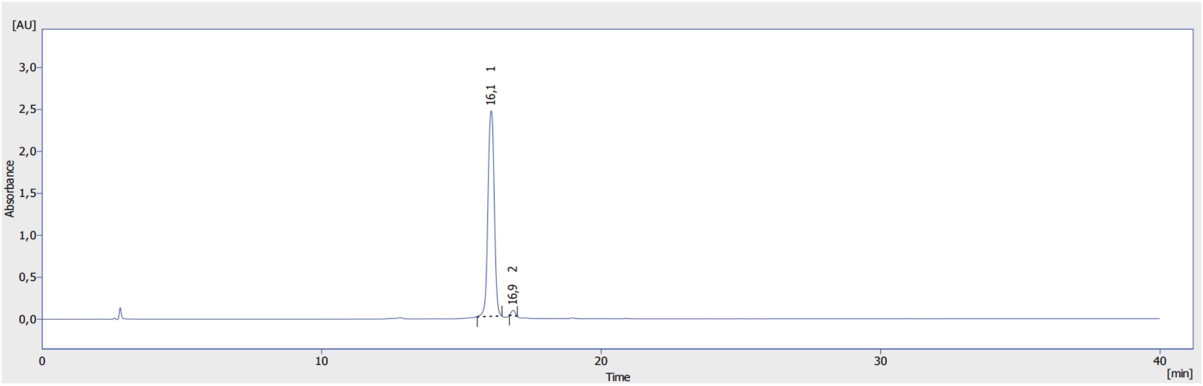

	Reten. Time [min]	Area [mAU.s]	Height [mAU]	Area [%]	Height [%]	W05 [min]	PDA Peak Purity	Compound Name	PDA Best Match Name	PDA Best Match
1	16,067	38288,818	2452,307	98,4	97,5	0,27	804			
2	16,850	625,174	63,251	1,6	2,5	0,18	998			
	Total	38913,992	2515,558	100,0	100,0					

m.p.: 144-146 °C.

5.5.19 (*Z*)-*N*-(1-(4-methoxyphenyl)-4-oxo-4-(4-(trifluoromethyl)phenyl)but-2-en-1-yl)-*N*,4-dimethylbenzenesulfonamide (13d)

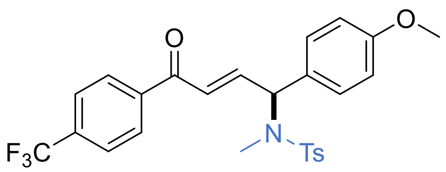

Following the general procedure **C** compound **13d** was obtained from enol **S14** (0.25 mmol) and *N*-aminopyridinium salt **2a** (0.33 mmol). The crude product was purified by column chromatography (20:80 AcOEt/Hexane) to afford 55 mg of compound **13d** as a yellow solid (**Yield = 44%**, 1h).

^{1}H NMR (400 MHz, CDCl$_3$): δ 7.93 (d, *J* = 8.1 Hz, 2H), 7.73 (d, *J* = 8.1 Hz, 4H), 7.27 (d, *J* = 7.8 Hz, 2H), 7.14 – 7.08 (m, 3H), 6.94 – 6.91 (m, 1H), 6.84 (d, *J* = 8.7 Hz, 2H), 5.93 (d, *J* = 5.7 Hz, 1H), 3.79 (s, 3H), 2.64 (s, 3H), 2.35 (s, 3H) ppm.

^{13}C NMR (100 MHz, CDCl$_3$): δ 188.6, 159.6, 144.5, 143.6, 140.0, 136.7, 134.8, 134.5, 134.3, 134.0, 129.7, 129.6, 128.9, 128.2, 127.3, 125.7, 125.6, 124.6, 122.5, 114.2, 61.1, 55.3, 30.1, 21.4 ppm.

HRMS (ESI, *m/z*): calcd. for $(C_{26}H_{24}F_3NO_4S + Na)^+$: 526.1276, found: 526.1278.

Analytical HPLC:

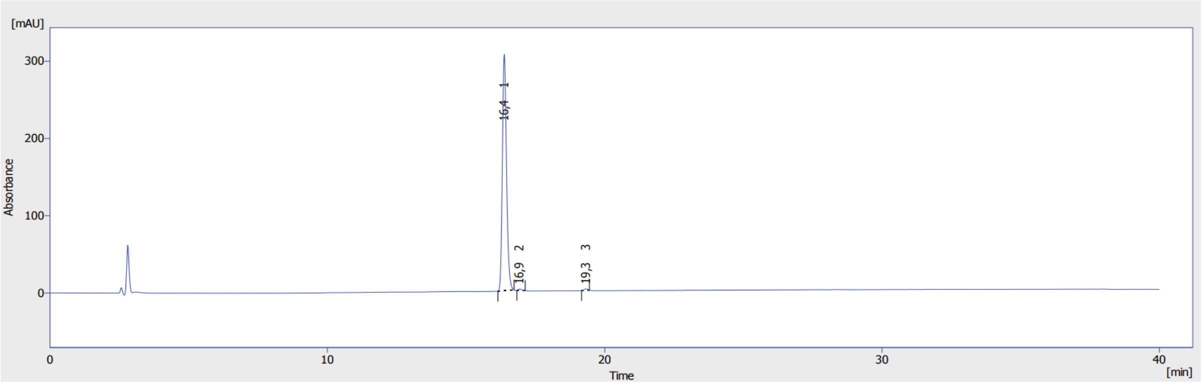

	Reten. Time [min]	Area [mAU.s]	Height [mAU]	Area [%]	Height [%]	W05 [min]	PDA Peak Purity	Compound Name	PDA Best Match Name	PDA Best Match
1	16,383	3174,496	305,962	99,0	98,8	0,17	955			
2	16,933	14,257	1,691	0,4	0,5	0,15	1000			
3	19,333	16,606	1,919	0,5	0,6	0,17	1000			
	Total	3205,358	309,572	100,0	100,0					

m.p.: 125-126 ^{o}C.

5.5.20 (*E*)-*N*-(1,4-bis(3-methoxyphenyl)-4-oxobut-2-en-1-yl)-*N*,4-dimethylbenzenesulfonamide (13b)

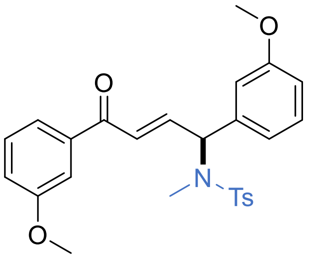

Following the general procedure **C** compound **13b** was obtained from enol **S16** (0.25 mmol) and *N*-aminopyridinium salt **2a** (0.33 mmol). The crude product was purified by column chromatography (30:70 AcOEt/Hexane) to afford 72 mg of compound **13b** as a yellow oil (**Yield = 62%**, 1 h).

^{1}H NMR (400 MHz, CDCl$_3$): δ 7.73 (d, *J* = 8.3 Hz, 2H), 7.42 – 7.39 (m, 1H), 7.38 – 7.34 (m, 2H), 7.26 (s, 1H), 7.23 (d, *J* = 7.8 Hz, 2H), 7.13 (dt, *J* = 6.5, 2.7 Hz, 1H), 7.04 (dd, *J* = 15.4, 6.4 Hz, 1H), 6.89 (dd, *J* = 15.4, 1.4 Hz, 1H), 6.86 – 6.79 (m, 2H), 6.74 (t, *J* = 1.9 Hz, 1H), 5.91 (d, *J* = 6.4 Hz, 1H), 3.87 (s, 3H), 3.75 (s, 3H), 2.68 (s, 3H), 2.33 (s, 3H) ppm.
^{13}C NMR (100 MHz, CDCl$_3$): δ 189.1, 159.9, 143.6, 142.0, 128.6, 138.3, 136.7, 129.7, 129.6, 128.4, 127.3, 121.1, 120.4, 119.7, 113.9, 113.8, 112.9, 61.5, 55.5, 55.2, 30.2, 21.4 ppm.

HRMS (ESI, *m/z*): calcd. for $(C_{26}H_{27}NO_5S + Na)^+$: 488.1508, found: 488.1514.

Analytical HPLC:

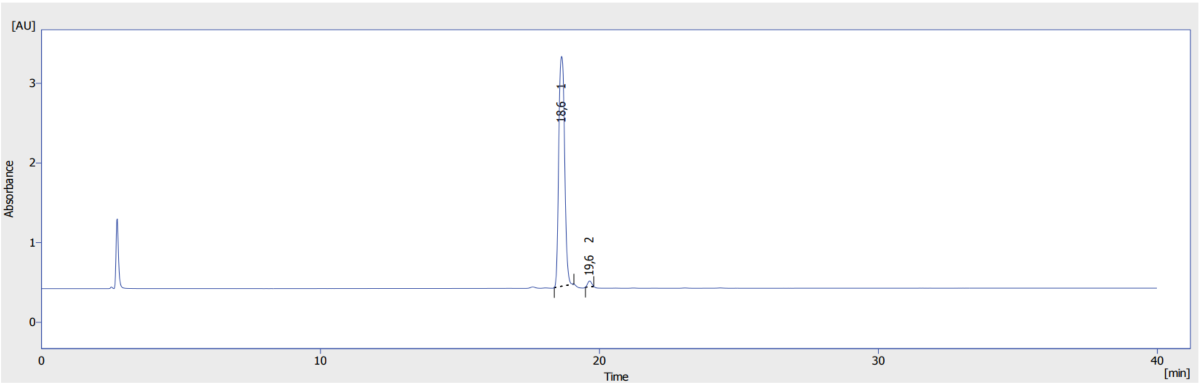

	Reten. Time [min]	Area [mAU.s]	Height [mAU]	Area [%]	Height [%]	W05 [min]	PDA Peak Purity	Compound Name	PDA Best Match Name	PDA Best Match
1	18,650	42171,853	2884,804	98,3	97,5	0,25	795			
2	19,650	714,954	73,826	1,7	2,5	0,18	998			
	Total	42886,807	2958,630	100,0	100,0					

5.5.21 (*Z*)-*N*-(1,4-bis(2-methoxyphenyl)-4-oxobut-2-en-1-yl)-*N*,4-dimethylbenzenesulfonamide (13c)

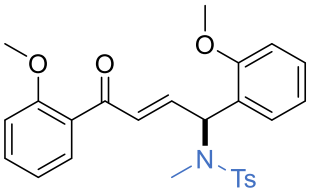

Following the general procedure **C** compound **13c** was obtained from enol **S17** (0.25 mmol) and *N*-aminopyridinium salt **2a** (0.33 mmol). The crude product was purified by column chromatography (30:70 AcOEt/Hexane) to afford 70 mg of compound **13c** as a yellow oil (**Yield = 60%**, 1 h).

^{1}H NMR (400 MHz, CDCl$_3$): δ 7.67 (d, J = 8.2 Hz, 2H), 7.59 (dd, J = 7.6, 1.7 Hz, 1H), 7.45 (ddd, J = 8.4, 7.4, 1.8 Hz, 1H), 7.28 (ddd, J = 8.4, 7.5, 1.8 Hz, 1H), 7.20 – 7.15 (m, 3H), 7.0 (td, J = 7.5, 0.8 Hz, 1H), 6.95 (d, J = 9.1 Hz, 3H), 6.90 (td, J = 7.5, 0.9 Hz, 1H), 6.8 (d, J = 8.2 Hz, 1H), 6.28 (s, 1H), 3.9 (s, 3H), 3.7 (s, 3H), 2.6 (s, 3H), 2.4 (s, 3H) ppm.

^{13}C NMR (100 MHz, CDCl$_3$): δ 192.0, 158.4, 157.1, 142.9, 142.8, 137.3, 133.2, 131.7, 130.5, 130.4, 129.8, 129.2, 128.6, 127.4, 125.0, 120.6, 120.2, 111.5, 110.8, 56.6, 55.6, 55.1, 30.9, 21.4 ppm.

HRMS (ESI, *m/z*): calcd. for ($C_{26}H_{27}NO_5S$ + Na)$^+$: 488.1508, found: 488.1509.

Analytical HPLC:

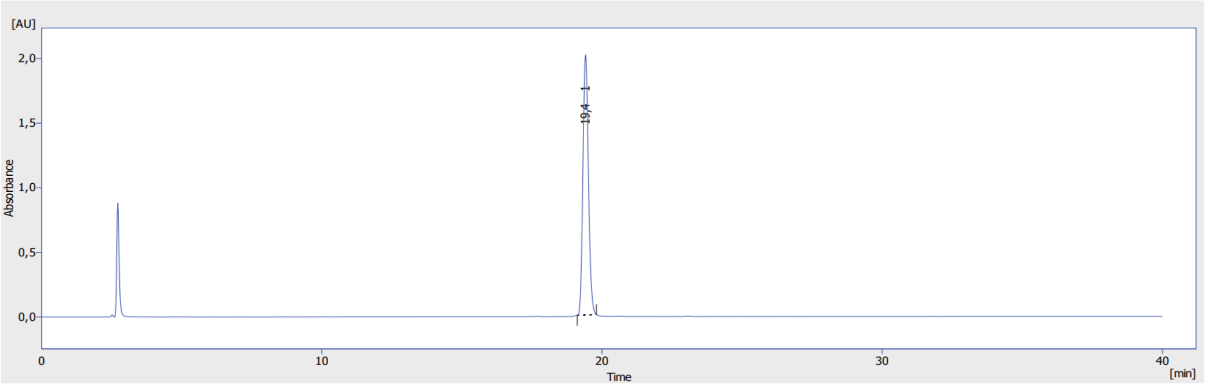

	Reten. Time [min]	Area [mAU.s]	Height [mAU]	Area [%]	Height [%]	W05 [min]	PDA Peak Purity	Compound Name	PDA Best Match Name	PDA Best Match
1	19,417	27359,927	2013,348	100,0	100,0	0,23	837			
	Total	27359,927	2013,348	100,0	100,0					

5.5.22 (*Z*)-*N*-(1,4-bis(4-cyanophenyl)-1-oxobut-2-en-2-yl)-*N*,4-dimethylbenzenesulfon amide (14a)

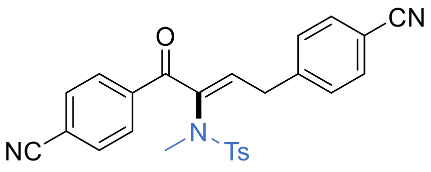

Following the general procedure **C** compound **14a** was obtained from enol **S18** (0.25 mmol) and *N*-aminopyridinium salt **2a** (0.33 mmol). The crude product was purified by column chromatography (30:70 AcOEt/Hexane) to afford 66 mg of compound **14a** as a yellow solid (**Yield = 58%**, 4 h).

^{1}H NMR (500 MHz, $CDCl_3$): δ 7.93 (d, *J* = 8.2 Hz, 2H), 7.71 (d, *J* = 8.2 Hz, 2H), 7.59 (d, *J* = 8.1 Hz, 2H), 7.45 (d, *J* = 8.2 Hz, 2H), 7.24 (d, *J* = 8.3 Hz, 2H), 7.19 (d, *J* = 8.7 Hz, 2H), 6.59 (t, *J* = 6.8 Hz, 1H), 3.86 (d, *J* = 6.8 Hz, 2H), 3.01 (s, 3H), 2.31 (s, 3H) ppm.

^{13}C NMR (100 MHz, $CDCl_3$): δ 195.0, 144.2, 141.0, 140.4, 139.1, 136.3, 132.6, 132.3, 129.8, 128.6, 127.4, 126.9, 126.6, 118.4, 117.7, 116.9, 111.9, 38.6, 37.3, 21.5 ppm.

HRMS (ESI, *m/z*): calcd. for $(C_{26}H_{21}N_3O_3S + Na)^+$: 478.1201, found: 478.1201.

Analytical HPLC: decomposition.

m.p.: 168-170 ^{o}C.

5.5.23 (*E*)-*N*,4-dimethyl-*N*-(4-oxo-1,4-diphenylbut-2-en-1-yl)benzenesulfonamide (12)

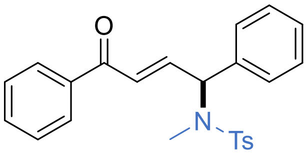

Following the general procedure **C** compound **12** was obtained from enol **S20** (0.25 mmol) and *N*-aminopyridinium salt **2a** (0.33 mmol). The crude product was purified by column chromatography (20:80 AcOEt/Hexane) to afford 80 mg of compound **12** as a yellow oil (**Yield = 79%**, 16 h).

^{1}H NMR (500 MHz, $CDCl_3$): δ 7.81 (d, *J* = 7.5 Hz, 2H), 7.72 (d, *J* = 8.1 Hz, 2H), 7.58 (t, *J* = 7.4 Hz, 1H), 7.46 (t, *J* = 7.7 Hz, 2H), 7.34 – 7.28 (m, 3H), 7.26 – 7.22 (m, 4H), 7.05 (dd, *J* = 15.5, 6.4 Hz, 1H), 6.90 (dd, *J* = 15.5, 0.9 Hz, 1H), 5.96 (d, *J* = 6.4 Hz, 1H), 2.66 (s, 3H), 2.31 (s, 3H) ppm.

^{13}C NMR (125 MHz, $CDCl_3$): δ 189.3, 143.6, 142.1, 137.2, 136.8, 136.7, 133.2, 129.7, 128.8, 128.7, 128.6, 128.3, 128.2, 127.3, 61.5, 30.2, 21.4 ppm.

HRMS (ESI, *m/z*): calcd. for $(C_{24}H_{23}NO_3S + Na)^+$: 428.1296; found: 428.1288.

Elemental analysis (%) calcd. for $C_{24}H_{23}NO_3S$: C 71.09, H 5.72, N 3.45, S 7.91; found: C 70.83, H 5.67, N 3.69, S 8.08.

5.5.24 *N*,4-dimethyl-*N*-(4-oxocyclohex-2-en-1-yl)benzenesulfonamide (15)

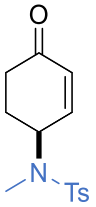

Following the general procedure **C** compound **15** was obtained from enol **S21** (0.25 mmol) and *N*-aminopyridinium salt **2a** (0.33 mmol). The crude product was purified by column chromatography (20:80 Et_2O/Pentane) to afford 31 mg of compound **15** as a white solid (**Yield = 45%**, 1 h).

^{1}H NMR (500 MHz, $CDCl_3$): δ 7.73 (d, *J* = 8.2 Hz, 2H), 7.34 (d, *J* = 8.1 Hz, 2H), 6.45 (dt, *J* = 10.3, 1.7 Hz, 1H), 6.01 (ddd, *J* = 10.3, 2.5, 0.8 Hz, 1H), 4.93 (ddd, *J* = 10.2, 5.4, 2.5 Hz, 1H), 2.76 (s, 3H), 2.59 – 2.37 (m, 5H), 2.08 – 1.95 (m, 2H) ppm.

^{13}C NMR (125 MHz, $CDCl_3$): δ 197.1, 150.2, 143.8, 136.3, 132.2, 130.0, 127.1, 54.5, 36.6, 29.8, 27.2, 21.5 ppm.

HRMS (ESI, *m/z*): calcd. for $(C_{14}H_{17}NO_3S + Na)^+$: 302.0827; found: 302.0818.

Elemental analysis (%) calcd. for $C_{14}H_{17}NO_3S$: C 60.19, H 6.13, N 5.01, S 11.48; found: C 60.13, H 6.11, N 5.22, S 11.56.

5.5.25 *N*-((5,5-dimethyl-3-oxocyclohex-1-en-1-yl)methyl)-*N*,4-dimethylbenzenesulfonamide (16)

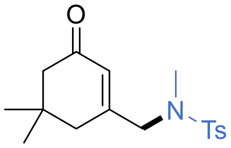

Following the general procedure **C** compound **16** was obtained from enol **S22** (0.25 mmol) and *N*-aminopyridinium salt **2a** (0.33 mmol). The crude product was purified by column chromatography (20:80 Et_2O/Pentane) to afford 51 mg of compound **16** as a yellow solid (**Yield = 63%**, 1 h).

^{1}H NMR (500 MHz, $CDCl_3$): δ 7.68 (d, *J* = 8.3 Hz, 2H), 7.35 (d, *J* = 8.0 Hz, 2H), 5.9 (s, 1H), 3.67 (s, 2H), 2.63 (s, 3H), 2.45 (s, 3H), 2.28 (s, 2H), 2.26 (s, 2H), 1.06 (s, 6H) ppm.

^{13}C NMR (125 MHz, $CDCl_3$): δ 199.4, 156.6, 143.8, 134.0, 129.9, 127.4, 126.8, 55.7, 51.3, 40.8, 34.8, 33.6, 28.2, 21.5 ppm.

HRMS (ESI, *m/z*): calcd. for $(C_{17}H_{23}NO_3S + Na)^+$: 344.1296, found: 344.1304.

Analytical HPLC:

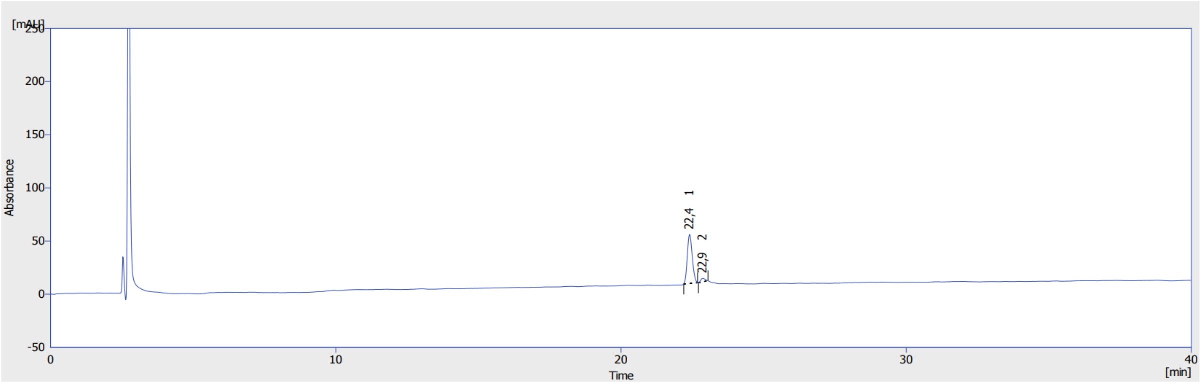

	Reten. Time [min]	Area [mAU.s]	Height [mAU]	Area [%]	Height [%]	W05 [min]	PDA Peak Purity	Compound Name	PDA Best Match Name	PDA Best Match
1	22,400	276,997	23,102	94,2	93,2	0,20	999			
2	22,867	17,185	1,686	5,8	6,8	0,22	1000			
	Total	294,182	24,789	100,0	100,0					

m.p.: 117-119 ^{o}C.

5.5.26 *N*,4-dimethyl-*N*-(4-oxocyclohept-2-en-1-yl)benzenesulfonamide (17)

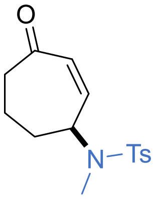

Following the general procedure **C** compound **17** was obtained from enol **S23** (0.25 mmol) and *N*-aminopyridinium salt **2a** (0.33 mmol). The crude product was purified by column chromatography (5:25:70 AcOEt/DCM/Hexane) to afford 50 mg of compound **17** as a yellow oil (**Yield = 69%**, 1 h).

^{1}H NMR (500 MHz, CDCl$_3$): δ 7.69 (d, *J* = 8.2 Hz, 2H), 7.28 (d, *J* = 8.1 Hz, 2H), 6.66 – 6.58 (m, 1H), 5.99 – 5.92 (m, 1H), 4.79 (dd, *J* = 10.6, 4.9 Hz, 1H), 2.83 (s, 3H), 2.55 – 2.43 (m, 2H), 2.42 (s, 3H), 2.05 – 1.79 (m, 4H) ppm.

^{13}C NMR (100 MHz, CDCl$_3$): δ 198.6, 146.4, 143.0, 136.6, 130.8, 129.4, 127.3, 65.7, 30.9, 29.3, 27.9, 23.6, 21.5 ppm.

HRMS (ESI, *m/z*): calcd. for $(C_{15}H_{19}NO_3S + Na)^+$: 316.0983, found: 316.0988.

Analytical HPLC:

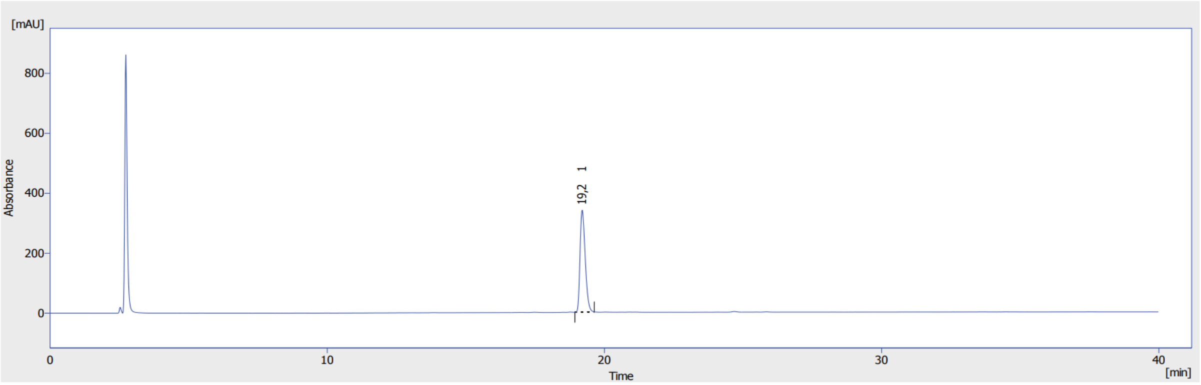

	Reten. Time [min]	Area [mAU.s]	Height [mAU]	Area [%]	Height [%]	W05 [min]	PDA Peak Purity	Compound Name	PDA Best Match Name	PDA Best Match
1	19,200	4400,738	340,387	100,0	100,0	0,22	944			
	Total	4400,738	340,387	100,0	100,0					

5.5.27 *N*,4-dimethyl-*N*-(5-oxo-2,5-dihydrofuran-2-yl)benzenesulfonamide (21)

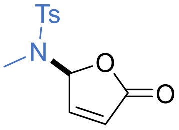

Following the general procedure **C** compound **21** was obtained from enol **S24** (0.25 mmol) and *N*-aminopyridinium salt **2a** (0.33 mmol). The crude product was purified by column chromatography (20:80 AcOEt/Hexane) to afford 50 mg of compound **21** as a white solid (**Yield = 74%**, 1 h).

^{1}H NMR (400 MHz, $CDCl_3$): δ 7.78 (d, *J* = 8.3 Hz, 2H), 7.35 (d, *J* = 8.1 Hz, 2H), 7.29 – 7.24 (m, 1H), 6.87 (t, *J* = 1.7 Hz, 1H), 6.30 (dd, *J* = 5.6, 1.9 Hz, 1H), 2.54 (s, 3H), 2.45 (s, 3H) ppm.
^{13}C NMR (100 MHz, $CDCl_3$): δ 170.0, 151.2, 144.5, 134.1, 129.9, 128.0, 125.7, 88.8, 28.3, 21.6 ppm.
HRMS (ESI, *m/z*): calcd. for $(C_{12}H_{13}NO_4S + Na)^+$: 290.0463; found: 290.0464.
Elemental analysis (%) calcd. for $C_{12}H_{13}NO_4S$: C 53.92, H 4.90, N 5.24, S 12.00; found: C 53.73, H 4.91, N 5.09, S 11.93.
m.p.: 89-92 ^{o}C.

5.5.28 *N*-((3*S*,4a*S*,5*R*)-4a,5-dimethyl-7-oxo-3-(prop-1-en-2-yl)-1,2,3,4,4a,5,6,7-octahydronaphthalen-1-yl)-*N*,4-dimethylbenzenesulfonamide (24)

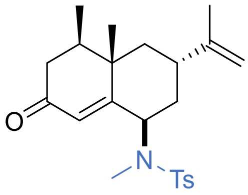

Following the general procedure **C** compound **24** was obtained from enol **S26** (0.25 mmol) and *N*-aminopyridinium salt **2a** (0.33 mmol). The crude product was purified by column chromatography (20:80 AcOEt/Hexane) to afford 68 mg of compound **24** as a white solid (**Yield = 68%**, 1h). Two diastereoisomers formed α/β = 1:7.

Two diastereoisomers: seperable by column chromatography, dr.r: α/β=1:7.

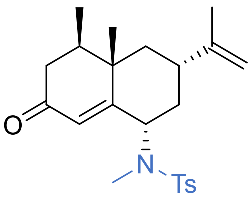

α

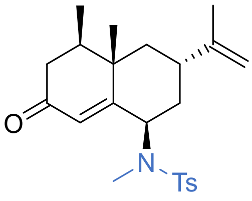

β

^{1}H NMR (400 MHz, $CDCl_3$): (major diastereomer, β) δ 7.68 (d, *J* = 8.3 Hz, 2H), 7.29 (d, *J* = 8.1 Hz, 2H), 5.94 (d, *J* = 2.2 Hz, 1H), 4.83 (ddd, *J* = 13.4, 5.3, 2.1 Hz, 1H), 4.77 – 4.76 (m, 2H), 2.63 (s, 3H), 2.59 – 2.54 (m, 1H), 2.42 (s, 3H), 2.34 – 2.31 (m, 2H), 2.09 – 1.99 (m, 2H), 1.88 (dd, *J* = 13.7, 7.2 Hz, 1H), 1.70 (s, 3H), 1.39 – 1.29 (m, 2H), 1.09 (s, 3H), 0.95 (d, *J* = 6.7 Hz, 3H) ppm.

^{13}C NMR (100 MHz, $CDCl_3$): (major diastereomer, β) δ 198.8, 168.6, 148.2, 143.5, 136.4, 129.8, 127.1, 123.8, 109.8, 55.8, 42.2, 39.9, 39.2, 34.9, 34.3, 29.2, 26.0, 21.5, 20.6, 17.5, 15.0 ppm.

HRMS (ESI, *m/z*): calcd. for $(C_{23}H_{31}NO_3S + Na)^+$: 424.1922, found: 424.1931.

Analytical HPLC: (major diastereomer)

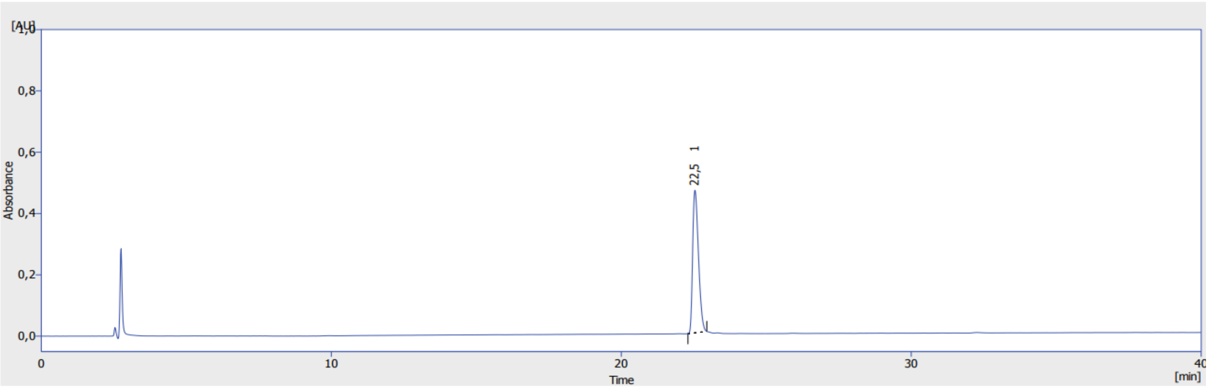

	Reten. Time [min]	Area [mAU.s]	Height [mAU]	Area [%]	Height [%]	W05 [min]	PDA Peak Purity	Compound Name	PDA Best Match Name	PDA Best Match
1	22,533	3221,411	232,495	100,0	100,0	0,23	957			
	Total	3221,411	232,495	100,0	100,0					

m.p.:151-153 °C.

5.5.29 (8*R*,9*S*,10*R*,13*S*,14*S*,17*S*)-6-((*N*,4-dimethylphenyl)sulfonamido)-10,13-dimethyl-3-oxo-2,3,6,7,8,9,10,11,12,13,14,15,16,17-tetradecahydro-1H-cyclopenta[*a*]phenanthren-17-yl acetate (25)

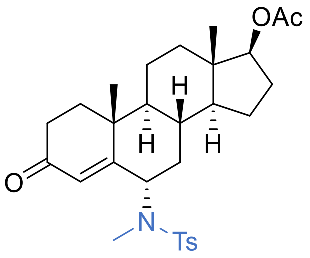

α

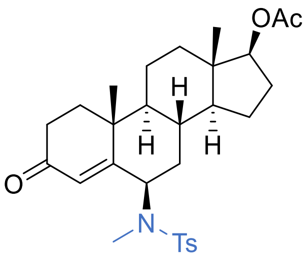

β

Following the general procedure **C** compound **25** was obtained from enol **S27** (0.25 mmol) and *N*-aminopyridinium salt **2a** (0.33 mmol). The crude product was purified by column chromatography (20:80 AcOEt/Hexane) to afford 68 mg of compound **25** as a white solid (**Yield = 61%**, 1h).

Two diastereoisomers: seperable by column chromatography, dr.r: α/β=1.73:1.

^{1}H NMR (400 MHz, $CDCl_3$): (major diastereomer, α) δ 7.67 (d, *J* = 8.2 Hz, 2H), 7.33 (d, *J* = 8.2 Hz, 2H), 5.90 (s, 1H), 4.62 (t, *J* = 8.5 Hz, 1H), 4.45 – 4.42 (m, 1H), 2.65 (s, 3H), 2.52 (ddd, *J* = 18.7, 13.5, 5.3 Hz, 1H), 2.43 (s, 4H), 2.21 – 2.13 (m, 1H), 2.04 (s, 3H), 2.00 – 1.93 (m, 2H), 1.86 (m, 1H), 1.79 (dt, *J* = 13.7, 3.4 Hz, 1H), 1.72 (dd, *J* = 13.3, 5.3 Hz, 1H), 1.60 – 1.56 (m, 2H), 1.51 – 1.46 (m, 1H), 1.42-1.38 (m, 1H), 1.43 – 1.31 (m, 1H), 1.27 – 1.22 (m, 2H), 1.19 (s, 3H), 1.12 – 1.07 (m, 2H), 0.8 (s, 3H) ppm.

^{13}C NMR (100 MHz, $CDCl_3$): (major diastereomer, α) δ 198.8, 171.1, 167.8, 143.7, 135.7, 129.8, 127.2, 124.9, 82.2, 57.0, 49.6, 48.8, 43.4, 38.5, 36.7, 35.3, 33.7, 31.4, 31.2, 27.4, 26.4, 23.1, 21.5, 21.1, 21.0, 18.5, 12.2 ppm.

HRMS (ESI, *m/z*): calcd. for $(C_{29}H_{39}NO_5S + Na)^+$: 536.2447, found: 536.2444.

Analytical HPLC: major diastereomer

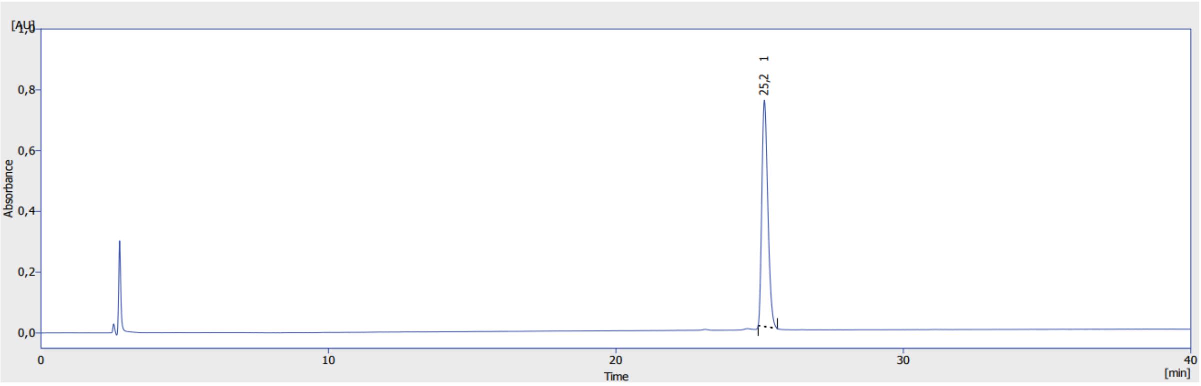

	Reten. Time [min]	Area [mAU.s]	Height [mAU]	Area [%]	Height [%]	W05 [min]	PDA Peak Purity	Compound Name	PDA Best Match Name	PDA Best Match
1	25,167	5394,776	372,341	100,0	100,0	0,25	940			
	Total	5394,776	372,341	100,0	100,0					

m.p.: 170-171 °C.

5.5.30 (*E*)-*N*-(7,7-dimethyl-6-oxooct-4-en-3-yl)-*N*,4-dimethylbenzenesulfonamide (18)

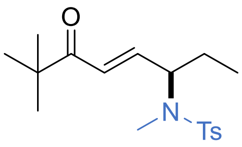

Following the general procedure **C** compound **18** was obtained from enol **S25** (0.25 mmol) and *N*-aminopyridinium salt **2a** (0.33 mmol). The crude product was purified by column chromatography (10:90 Et_2O/Pentane) to afford 22 mg of compound **18** as a colorless oil (**Yield = 26%**, 1 h).

^{1}H NMR (400 MHz, $CDCl_3$): δ 7.67 (d, *J* = 8.1 Hz, 2H), 7.26 (d, *J* = 8.0 Hz, 2H), 6.55 (dd, *J* = 15.4, 6.1 Hz, 1H), 6.39 – 6.33 (dd, *J* = 15.4, 0.9 Hz, 1H), 4.53 – 4.46 (q, *J* = 7.0 Hz, 1H), 2.69 (s, 3H), 2.39 (s, 3H), 1.60-1.52 (m, 2H), 1.06 (s, 9H), 0.91 (t, *J* = 7.3 Hz, 3H) ppm.

^{13}C NMR (100 MHz, $CDCl_3$): δ 203.6, 143.2, 142.3, 137.0, 129.6, 127.1, 125.5, 59.5, 43.0, 28.5, 25.9, 24.9, 21.4, 10.7 ppm.

HRMS (ESI, *m/z*): calcd. For $(C_{18}H_{27}NO_3S + Na)^+$: 360.1609, found: 360.1620.

Analytical HPLC:

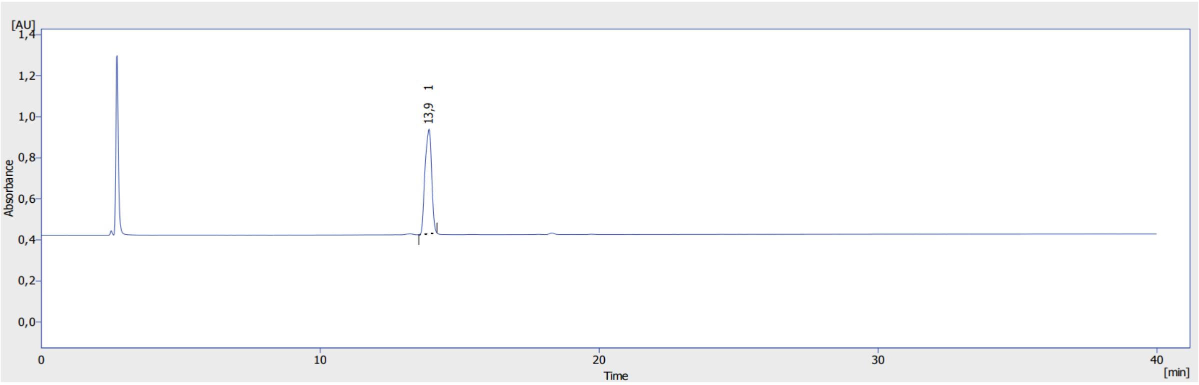

	Reten. Time [min]	Area [mAU.s]	Height [mAU]	Area [%]	Height [%]	W05 [min]	PDA Peak Purity	Compound Name	PDA Best Match Name	PDA Best Match
1	13,900	8333,381	509,664	100,0	100,0	0,30	924			
	Total	8333,381	509,664	100,0	100,0					

5.5.31 (*E*)-*N*,4-dimethyl-*N*-(6-oxohex-4-en-3-yl)benzenesulfonamide (23)

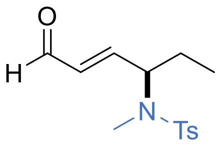

Following the general procedure **C** compound **23** was obtained from enol **S29** (0.25 mmol) and *N*-aminopyridinium salt **2a** (0.33 mmol). The crude product was purified by column chromatography (20:80 AcOEt/Pentane) to afford 20 mg of compound **23** as a colorless oil (**Yield = 28%**, 16 h).

^{1}H NMR (500 MHz, $CDCl_3$): δ 9.41 (d, *J* = 7.6 Hz, 1H), 7.69 (d, *J* = 8.2 Hz, 2H), 7.30 (d, *J* = 8.2 Hz, 2H), 6.48 (dd, *J* = 15.9, 5.4 Hz, 1H), 6.01 (ddd, *J* = 15.9, 7.6, 1.4 Hz, 1H), 4.61 (ddd, *J* = 10.5, 6.6, 1.3 Hz, 1H), 2.71 (s, 3H), 2.42 (s, 3H), 1.75 – 1.50 (m, 2H), 0.91 (t, *J* = 7.4 Hz, 3H) ppm.

^{13}C NMR (125 MHz, $CDCl_3$): δ 192.8, 153.0, 143.6, 136.6, 133.2, 129.7, 127.2, 59.2, 28.6, 24.1, 21.5, 10.7 ppm.

HRMS (ESI, *m/z*): calcd. for $(C_{14}H_{19}NO_3S + Na)^+$: 304.0983; found: 304.0980.

Elemental analysis (%) calcd. for $C_{14}H_{19}NO_3$: C 59.76, H 6.81, N 4.98, S 11.40; found: C 59.73, H 6.86, N 5.12, S 11.52.

5.5.32 *N*-(3,7-dimethyl-1-oxoocta-2,6-dien-4-yl)-*N*,4-dimethylbenzenesulfonamide (26)

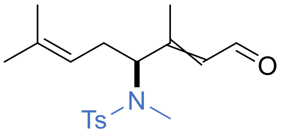

Following the general procedure **C** compound **26** was obtained from enol **S29** (0.25 mmol) and *N*-aminopyridinium salt **2a** (0.33 mmol). The crude product was purified by column chromatography (20:80 Et_2O/Pentane) to afford 51 mg of compound **26** as a colorless oil (**Yield = 61%**, *E*/*Z* ~ 1.38:1. 1 h).

^{1}H NMR (500 MHz, $CDCl_3$): (major) 9.96 (d, *J* = 7.9 Hz, 1H), 7.75-7.72 (m, 2H), 7.33-7.30 (m, 2H), 5.94 (dt, *J* = 7.7, 1.3 Hz, 1H), 5.09 (q, *J* = 7.4, 1.5 Hz, 1H), 4.37 – 4.36 (m, 1H), 3.69 (d, *J* = 1.4 Hz, 2H), 2.65 – 2.63 (m, 3H), 2.42 (6H), 1.68 (d, *J* = 1.3 Hz, 3H), 1.60 (d, *J* = 1.4 Hz, 3H) ppm.

^{13}C NMR (125 MHz, $CDCl_3$): δ 191.4, 190.5, 159.5, 158.7, 143.8, 143.5, 143.4, 136.8, 135.9, 134.7, 134.2, 134.0, 129.9, 129.7, 129.6, 128.8, 128.0, 127.4, 127.3, 122.0, 119.2, 62.6, 55.8, 35.0, 29.3, 29.0, 28.6, 27.6, 27.4, 25.7, 25.6, 21.5, 17.9, 17.8, 16.3 ppm.

HRMS (ESI, *m/z*): calcd. for $(C_{18}H_{25}NO_3S + Na)^+$: 358.1453, found: 358.1454.

Elemental analysis: decomposition.

5.5.33 *N*-(3-formyl-2,4,4-trimethylcyclohex-2-en-1-yl)-*N*,4-dimethylbenzenesulfonamide (27)

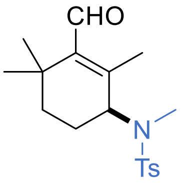

Following the general procedure **C** compound **27** was obtained from enol **S30** (0.25 mmol) and *N*-aminopyridinium salt **2a** (0.33 mmol). The crude product was purified by column chromatography (20:80 Et_2O/Pentane) to afford 43 mg of compound **27** as a colorless oil (**Yield = 52%**, 1 h).

^{1}H NMR (500 MHz, $CDCl_3$): δ 10.13 (s, 1H), 7.74 (d, *J* = 8.2 Hz, 2H), 7.34 (d, *J* = 7.9 Hz, 2H), 4.57 – 4.54 (m, 1H), 2.66 (s, 3H), 2.45 (s, 3H), 2.06 (s, 3H), 1.59 (ddd, *J* = 13.7, 9.8, 2.9 Hz, 1H), 1.49 – 1.41 (m, 3H), 1.18 (d, *J* = 1.3 Hz, 6H) ppm.

^{13}C NMR (125 MHz, $CDCl_3$): δ 192.8, 150.5, 145.0, 143.4, 136.9, 129.8, 127.0, 59.7, 37.6, 33.0, 29.6, 28.5, 26.7, 21.6, 15.0 ppm.

HRMS (EI, *m/z*): calcd. For $C_{18}H_{25}NO_3S$: 335.1555, found: 335.1553.

Analytical HPLC:

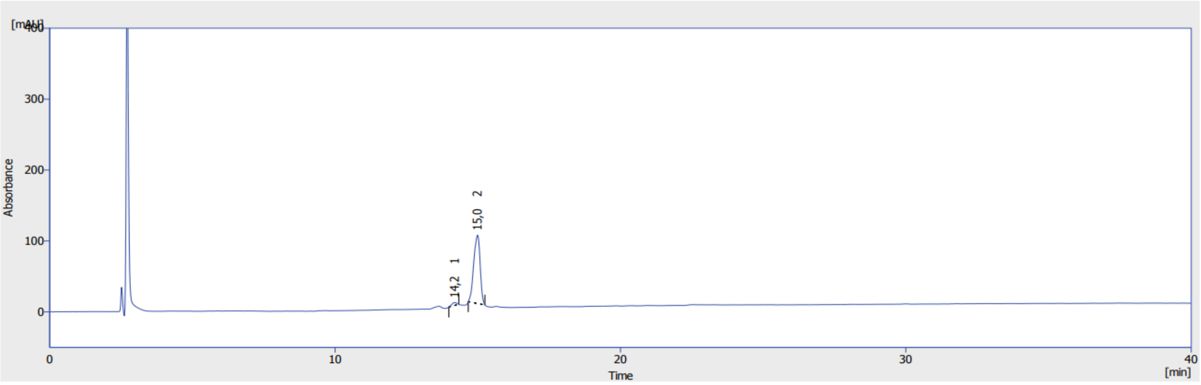

	Reten. Time [min]	Area [mAU.s]	Height [mAU]	Area [%]	Height [%]	W05 [min]	PDA Peak Purity	Compound Name	PDA Best Match Name	PDA Best Match
1	14,200	23,392	1,975	3,0	3,9	0,22	1000			
2	15,000	750,219	48,141	97,0	96,1	0,27	992			
	Total	773,611	50,117	100,0	100,0					

5.5.34 (*Z*)-*N*,4-dimethyl-*N*-(4-oxo-3-phenylbut-2-en-1-yl)benzenesulfonamide (22)

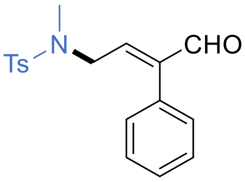

Following the general procedure **C** compound **22** was obtained from enol **S31** (0.25 mmol) and *N*-aminopyridinium salt **2a** (0.33 mmol). The crude product was purified by column chromatography (20:80 Et_2O/Pentane) to afford 36 mg of a mixture of compound **22** and Me-NH-Ts by-product which we were not able to separate flash column chromatography.

^{1}H NMR (500 MHz, $CDCl_3$): δ 9.62 (s, 1H), 7.62 (d, *J* = 8.1 Hz, 2H), 7.42 – 7.34 (m, 3H), 7.29 (d, *J* = 7.9 Hz, 2H), 7.11 – 7.08 (m, 2H), 6.64 (t, *J* = 6.4 Hz, 1H), 3.96 (d, *J* = 6.4 Hz, 2H), 2.69 (s, 3H), 2.42 (s, 3H) ppm.

^{13}C NMR (125 MHz, $CDCl_3$): δ 192.5, 148.4, 145.4, 143.8, 134.3, 131.2, 129.8, 129.7, 129.2, 128.6, 128.5, 127.4, 127.3, 48.7, 35.4, 21.5 ppm.

HRMS (ESI, *m/z*): calcd. for $(C_{18}H_{19}NO_3S + Na)^+$: 352.0983, found: 352.0987.

Analytical HPLC:

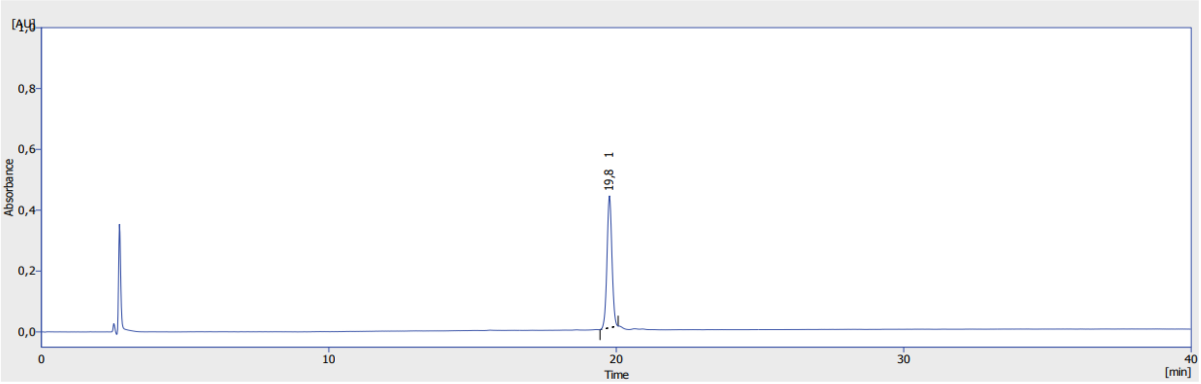

	Reten. Time [min]	Area [mAU.s]	Height [mAU]	Area [%]	Height [%]	W05 [min]	PDA Peak Purity	Compound Name	PDA Best Match Name	PDA Best Match
1	19,767	2606,011	216,766	100,0	100,0	0,20	977			
	Total	2606,011	216,766	100,0	100,0					

References

1. Cumbrias-Martin, J.; Perez-Aguilar M.C.; Mas-Balleste R.; Litta, A. D.; Lattanzi, A.; Sala, G.D.; Fernandez-Salas, J.A.; Aleman, J.; Enantioselective Conjugate Azidation of *α,β*-Unsaturated Ketones under Bifunctional Organocatalysis by Direct Activation of $TMSN_3$. *Adv. Synth. Catal.* **2019**, 361, 4790-4796.
2. Le Paih, J.; Monnier, F.; Dérien, S.; Dixneuf, P. H.; Clot, E.; Eisenstein, O.; Biscarbene-Ruthenium Complexes in Catalysis: Novel Stereoselective Synthesis of (1*E*,3*E*)-1,4-Disubstituted-1,3-dienes via Head-to-Head Coupling of Terminal Alkynes and Addition of Carboxylic Acids. *J. Am. Chem. Soc.* **2003**, *125*, 11964–11975.
3. Liu, X.; Chen, X.; Mohr, J. T.; Copper-Catalyzed γ-Sulfonylation of α,β-Unsaturated Carbonyl Compounds by Means of Silyl Dienol Ethers. *Org. Lett.* **2015**, *17*, 3572–3575.
4. Favara, D.; Omodei-Sale, A.; Consonni, P.; Depaoli, A.; A facile synthesis of trans (+)-4-carboxymethyl-3-ethylazetidin-2-one and its conversion into natural PS-5. *Tetrahedron Lett.* **1982**, *23*, 3105–3108.
5. Herscovici, J.; Boumaïza, L.; Antonakis, K.; Highly stereoselective preparation of conjugated dienones and dienoates via a new knoevnagel approach. *Tetrahedron Lett.* **1991**, *32*, 1791–1794.
6. Goliszewska, K.; Rybicka-Jasińska, K.; Szurmak, J.; Gryko. D.; Visible-Light-Mediated Amination of π-Nucleophiles with *N*-Aminopyridinium Salts. *J. Org. Chem.* **2019**, *84*, 15834–15844.
7. Lukas, R. J.; Muresan, A. Z.; Damaj, M. I.; Blough, B. E.; Huang, X.; Navarro, H. A.; Mascarella, S. W.; Eaton, J. B.; Marxer-Miller, S. K.; Carroll, F. I.; Synthesis and Characterization of in Vitro and in Vivo Profiles of Hydroxybupropion Analogues: Aids to Smoking Cessation. *J. Med. Chem.* **2010**, *53*, 4731-4748.
8. Fukuzawa S.; Tsuruta T.; Fujinami T.; Sakai S.; Reaction of α-halogeno ketones with carbonyl compounds promoted by CeI_3, $CeCl_3$-NaI, or $CeCl_3$-$SnCl_2$. *J. Chem. Soc.* **1987**, 1473-1477.
9. Pennel M. N., Unthank M. G., Turner P., Sheppard T. D., A General Procedure for the Synthesis of Enones via Gold-Catalyzed Meyer-Schuster Rearrangement of Propargylic Alcohols at Room Temperature. *J. Org. Chem.* **2011**, 76, 5, 1479-1482.
10. Zhang B., Davies H. M. L.; Rhodium-Catalyzed Enantioselective [4+2] Cycloadditions of Vinylcarbenes with Dienes. *Angew. Chem. Int. Ed.* **2020**, 59, 4937-4941.

6. NMR spectra

A) Known compounds

(2*E*,4*E*,6*E*)-1-phenylocta-2,4,6-trien-1-one (E3)

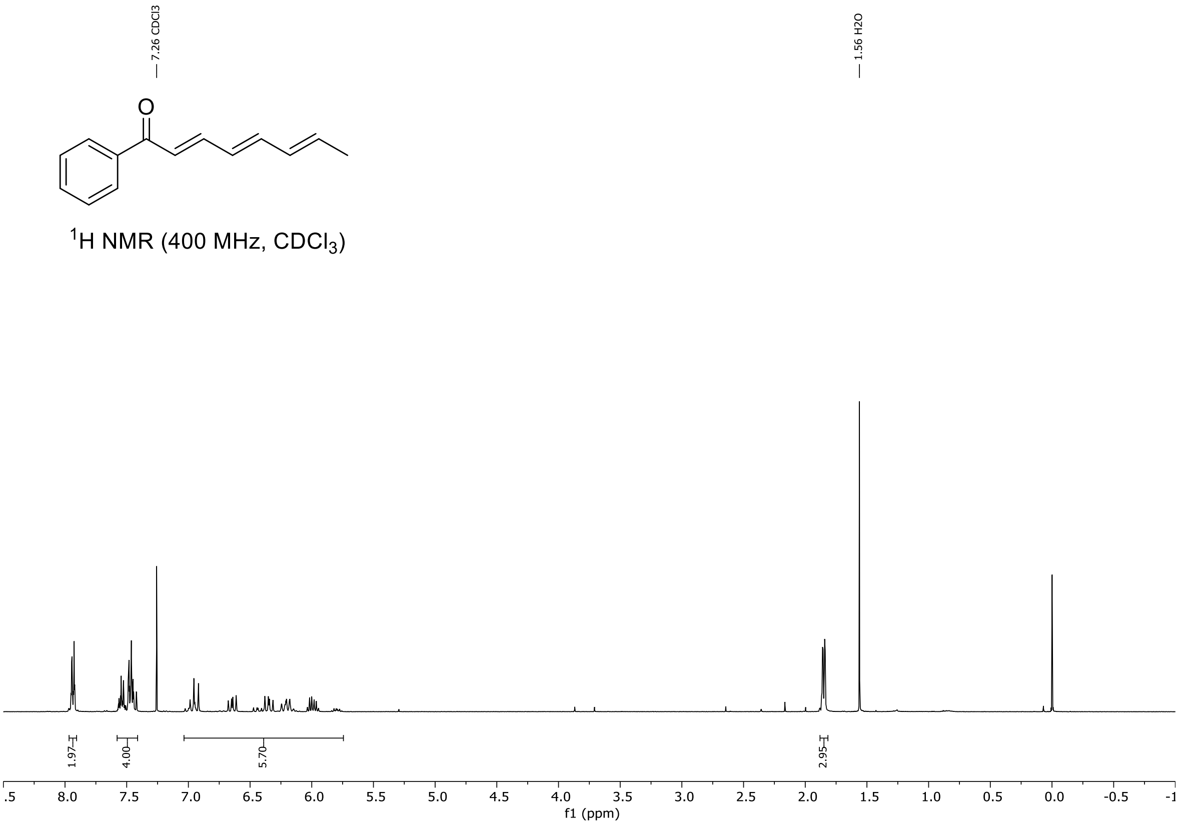

(*E*)-1-(4-methoxyphenyl)hex-2-en-1-one (E4)

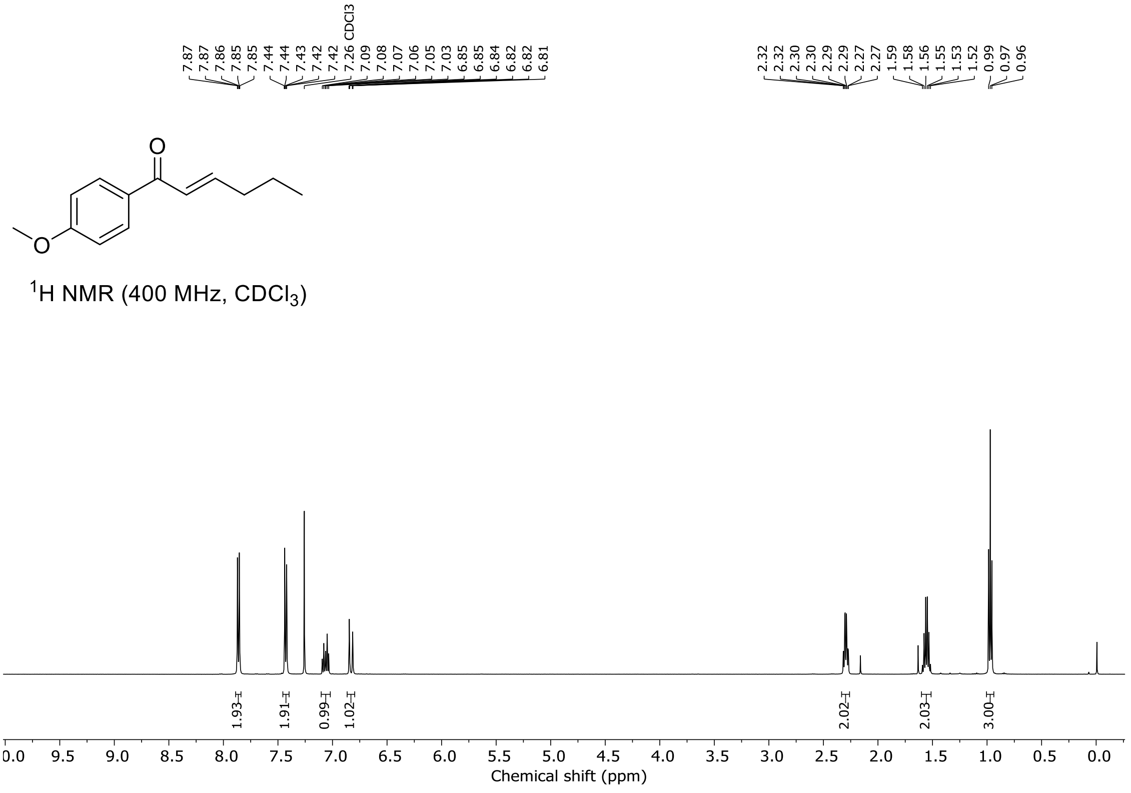

(*E*)-1-(benzo[*d*][1,3]dioxol-5-yl)hex-2-en-1-one (E8)

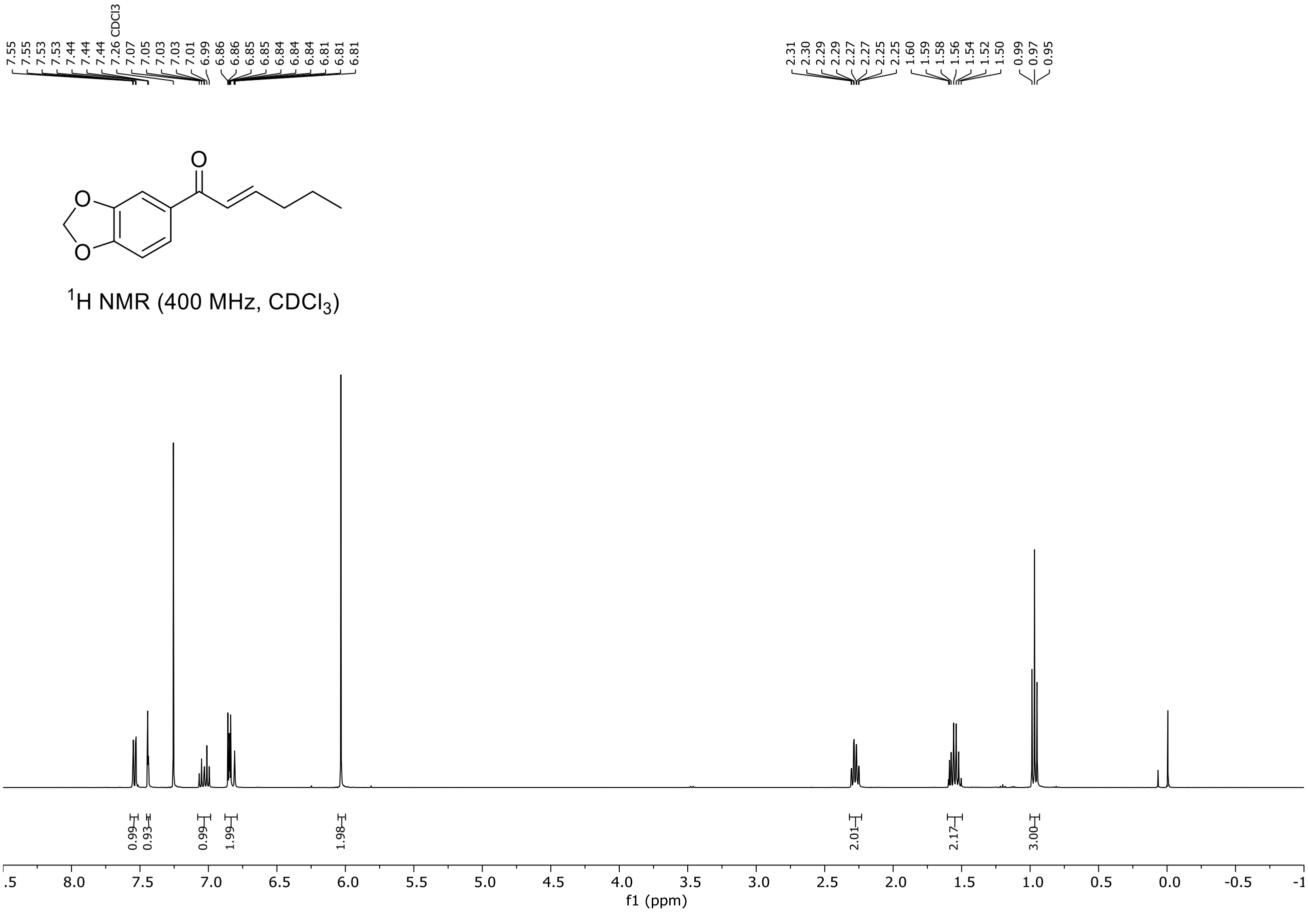

(*E*)-4-(hex-2-enoyl)benzonitrile (E11)

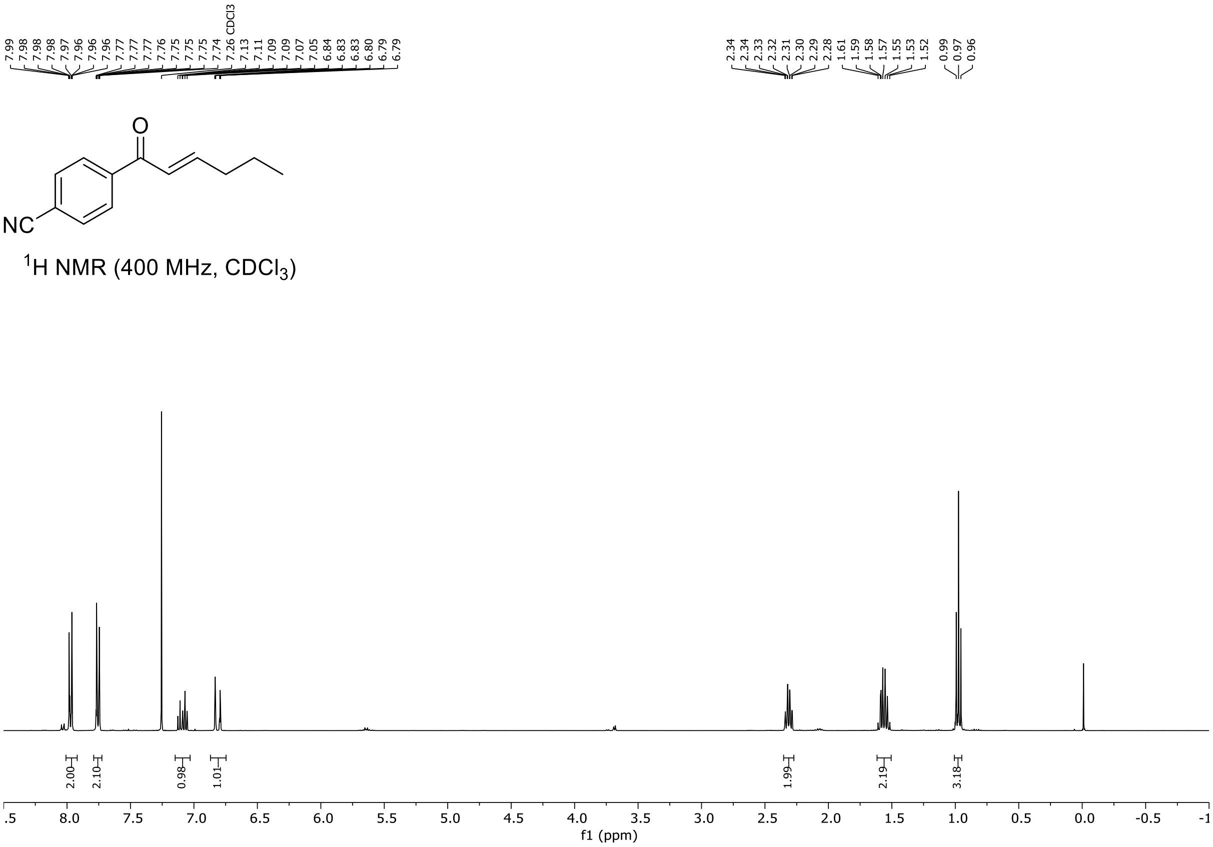

(*E*)-2,2-dimethyloct-4-en-3-one (E20)

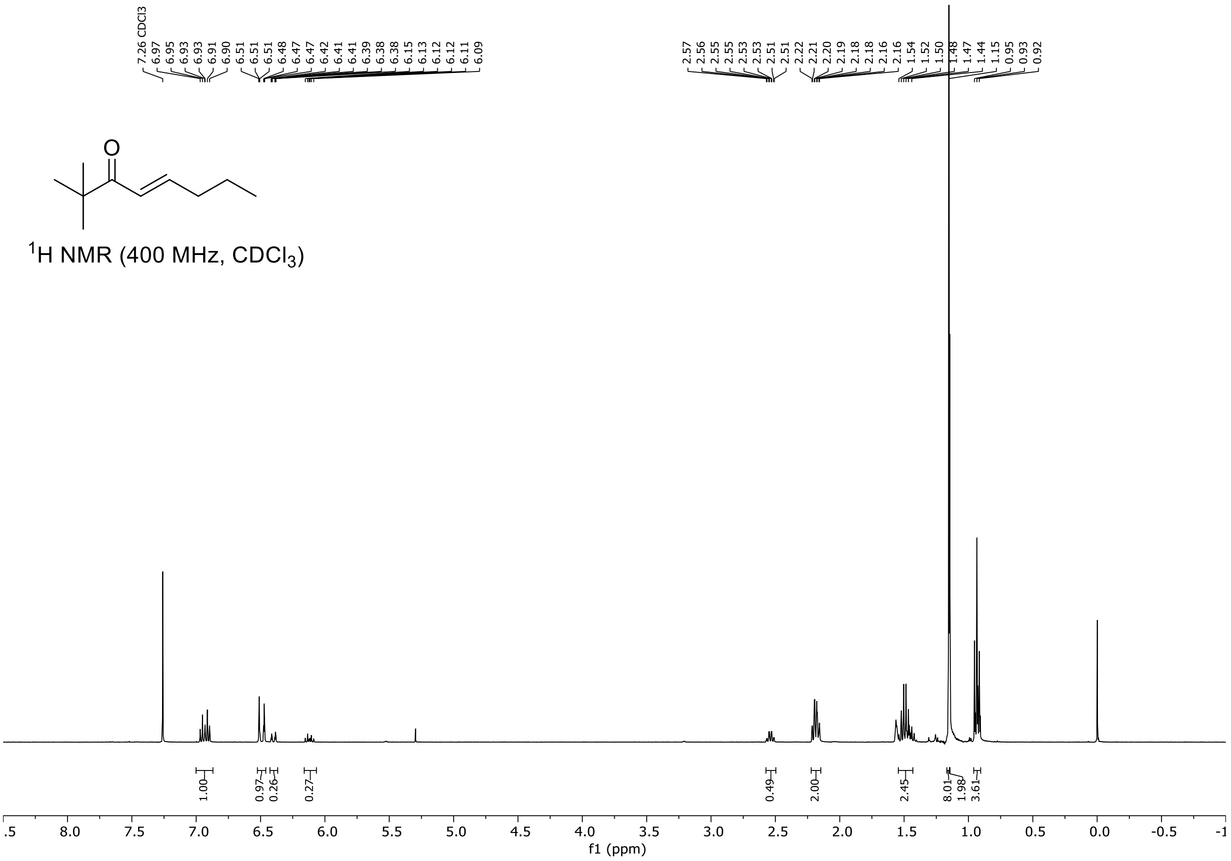

(1*Z*,3*E*)-1,4-bis(4-methoxyphenyl)buta-1,3-dien-1-yl acetate (S15)

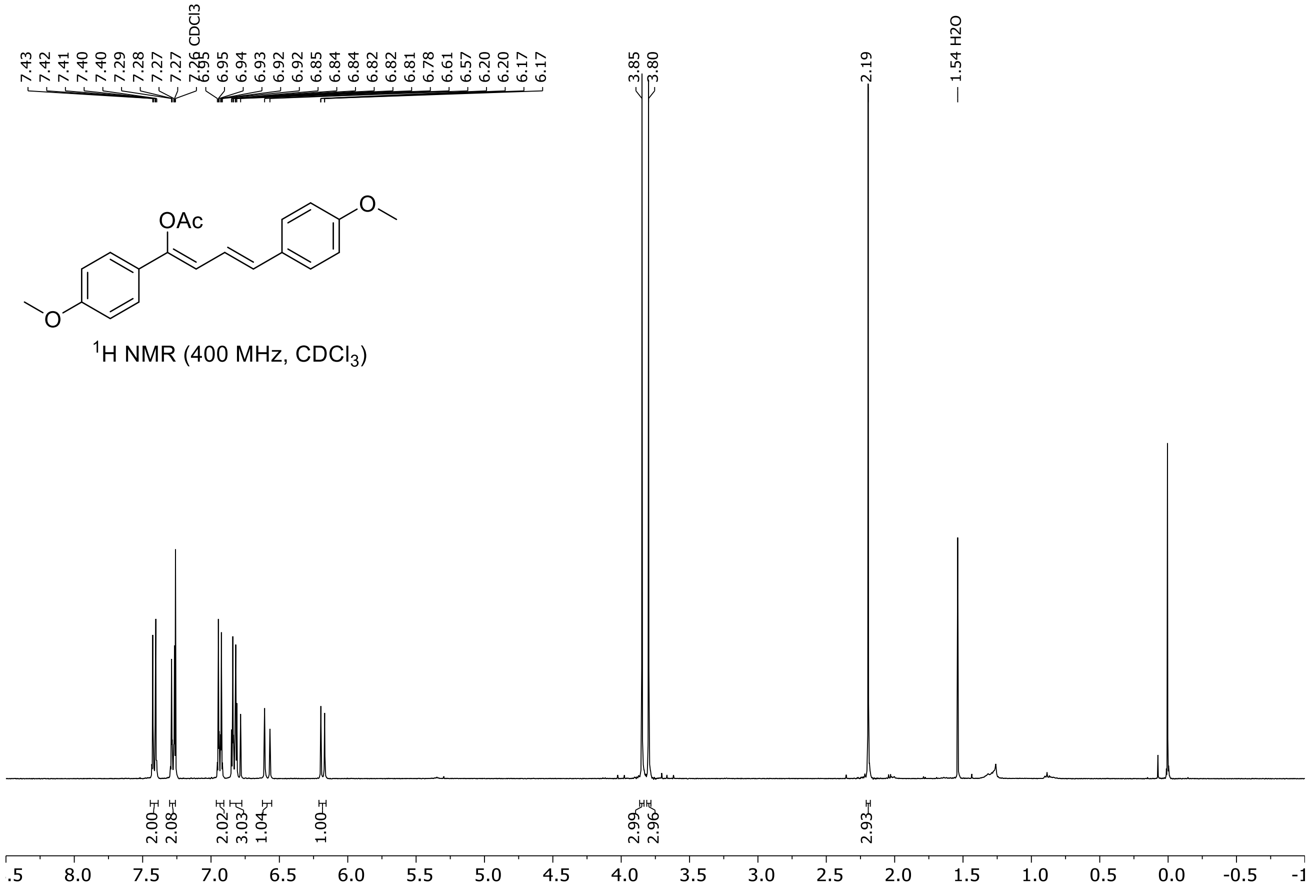

(1*Z*,3*E*)-1,4-bis(4-cyanophenyl)buta-1,3-dien-1-yl acetate (S18)

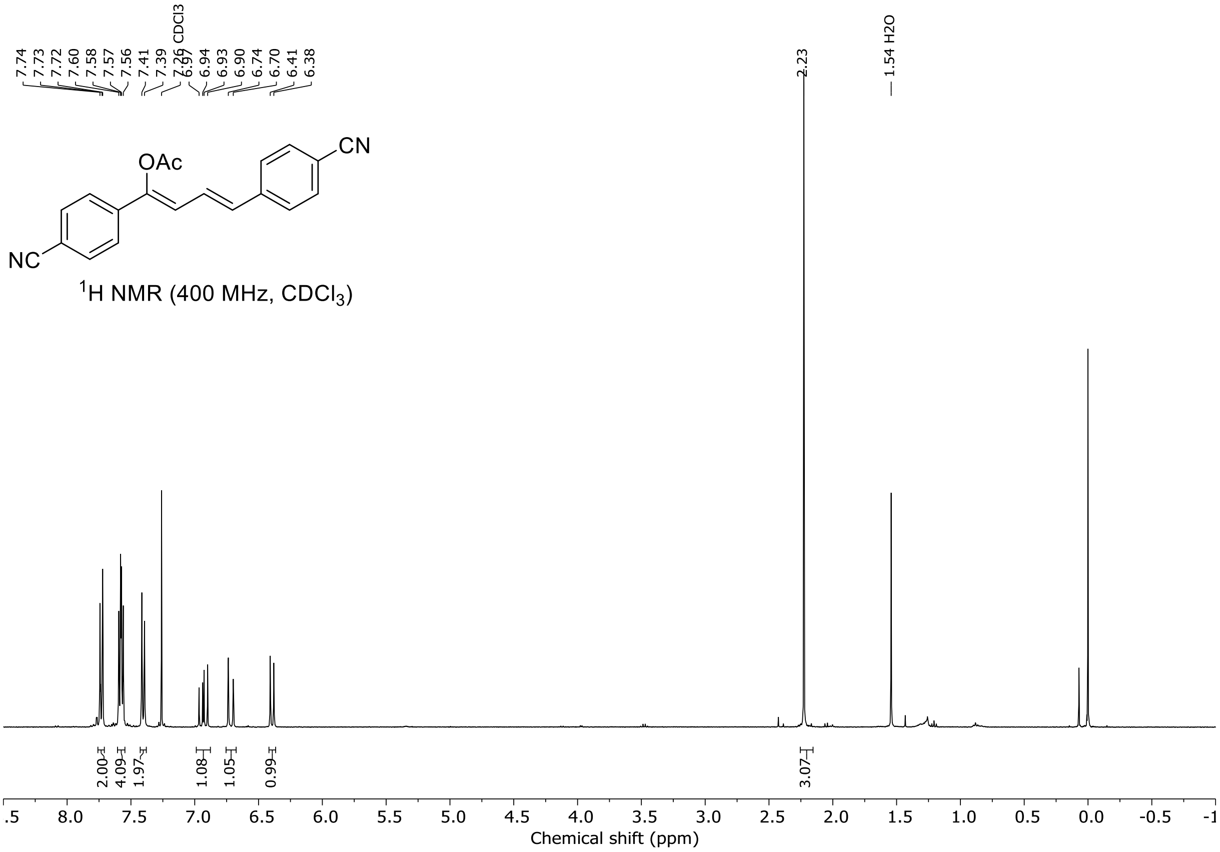

***tert*-butyl((5,5-dimethyl-3-methylenecyclohex-1-en-1-yl)oxy)dimethylsilane (S22)**

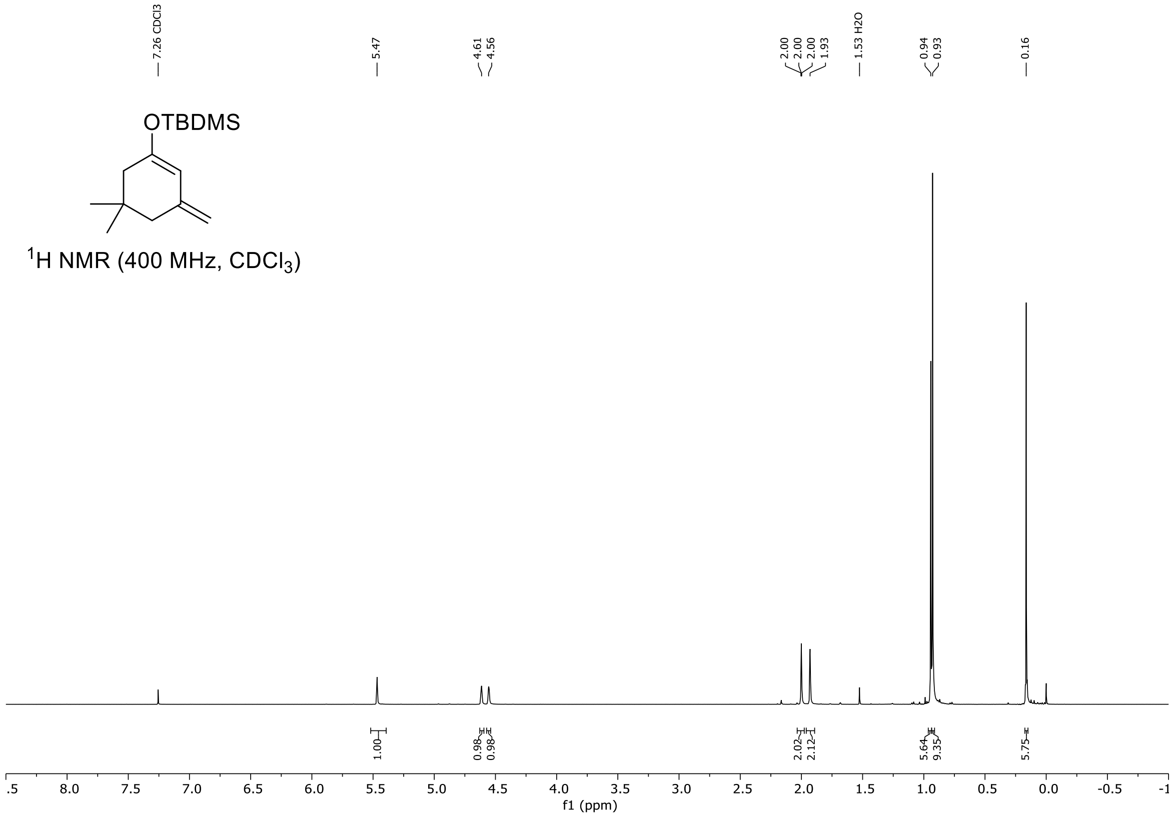

tert-butyl(cyclohepta-1,3-dien-1-yloxy)dimethylsilane (S23)

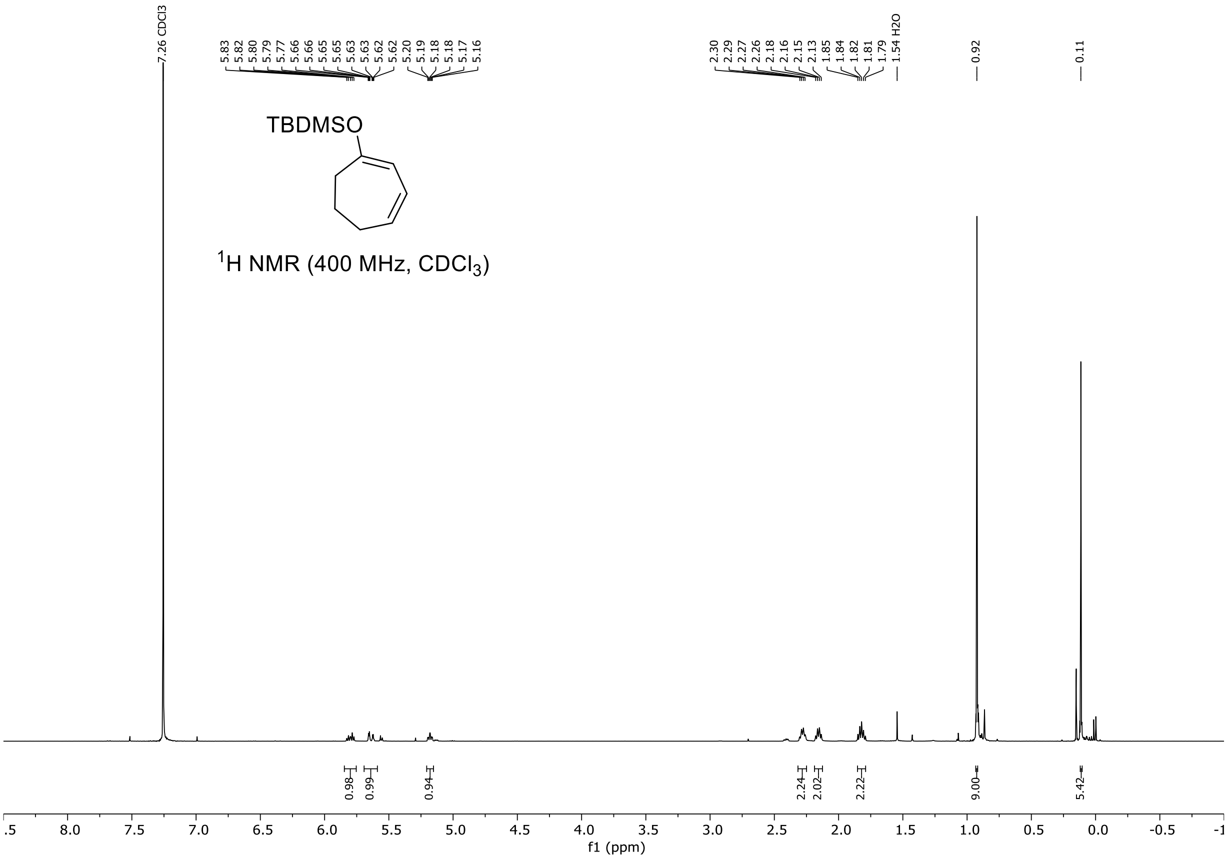

B) Unknown compounds

(*E*)-1-(4-(tert-butyl)phenyl)hex-2-en-1-one (E5)

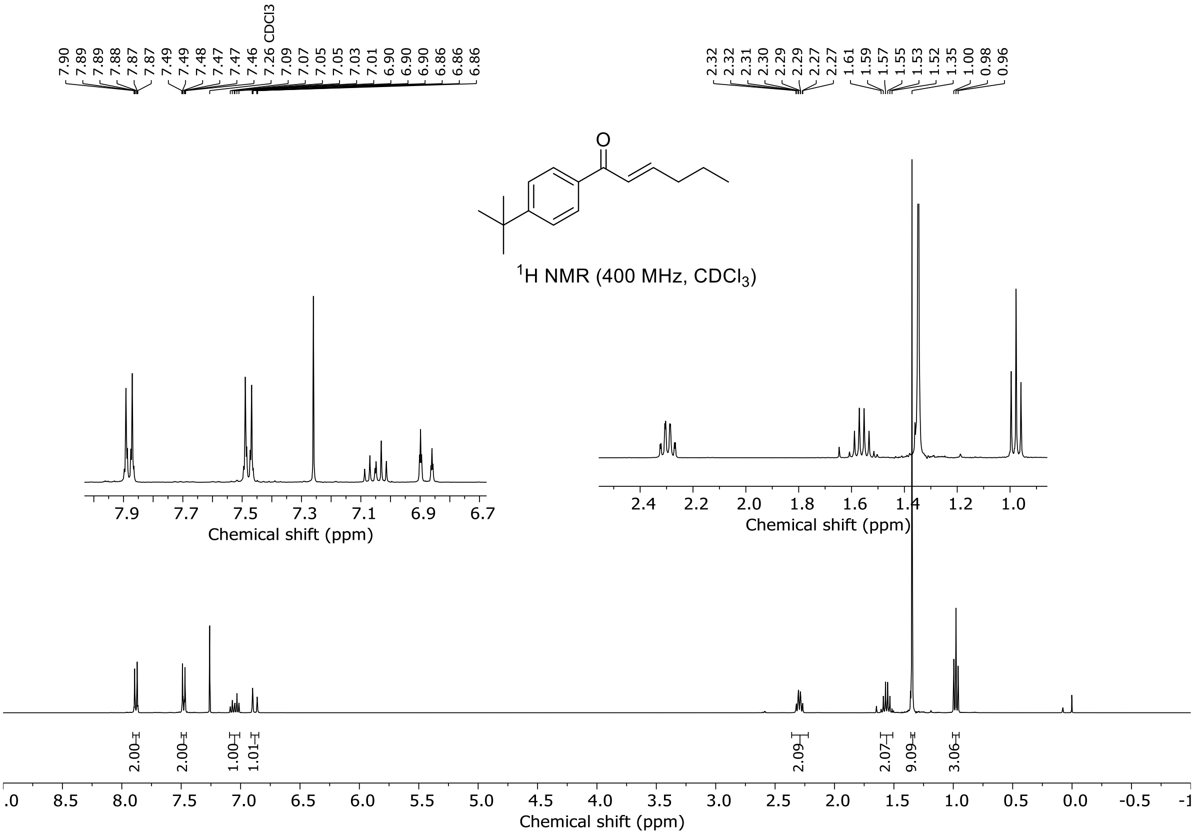

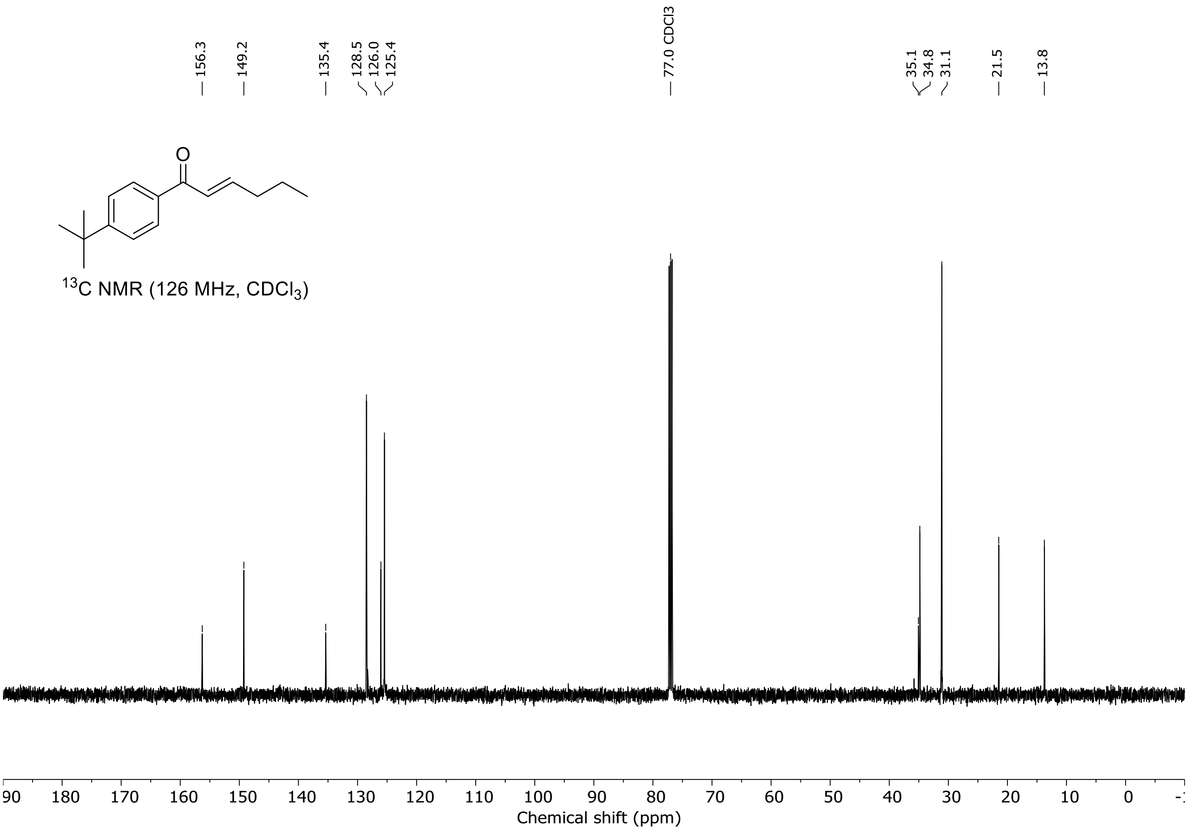

(*E*)-1-(4-chlorophenyl)hex-2-en-1-one (E6)

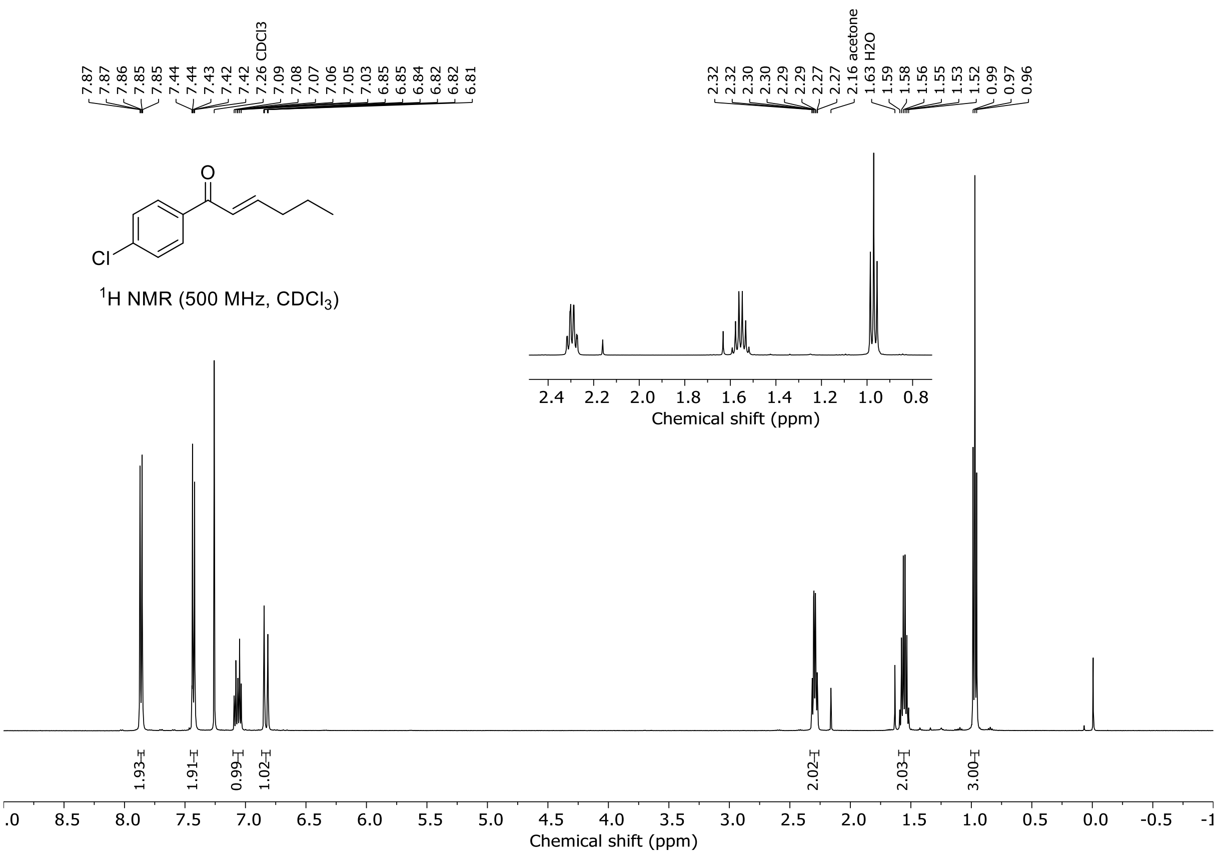

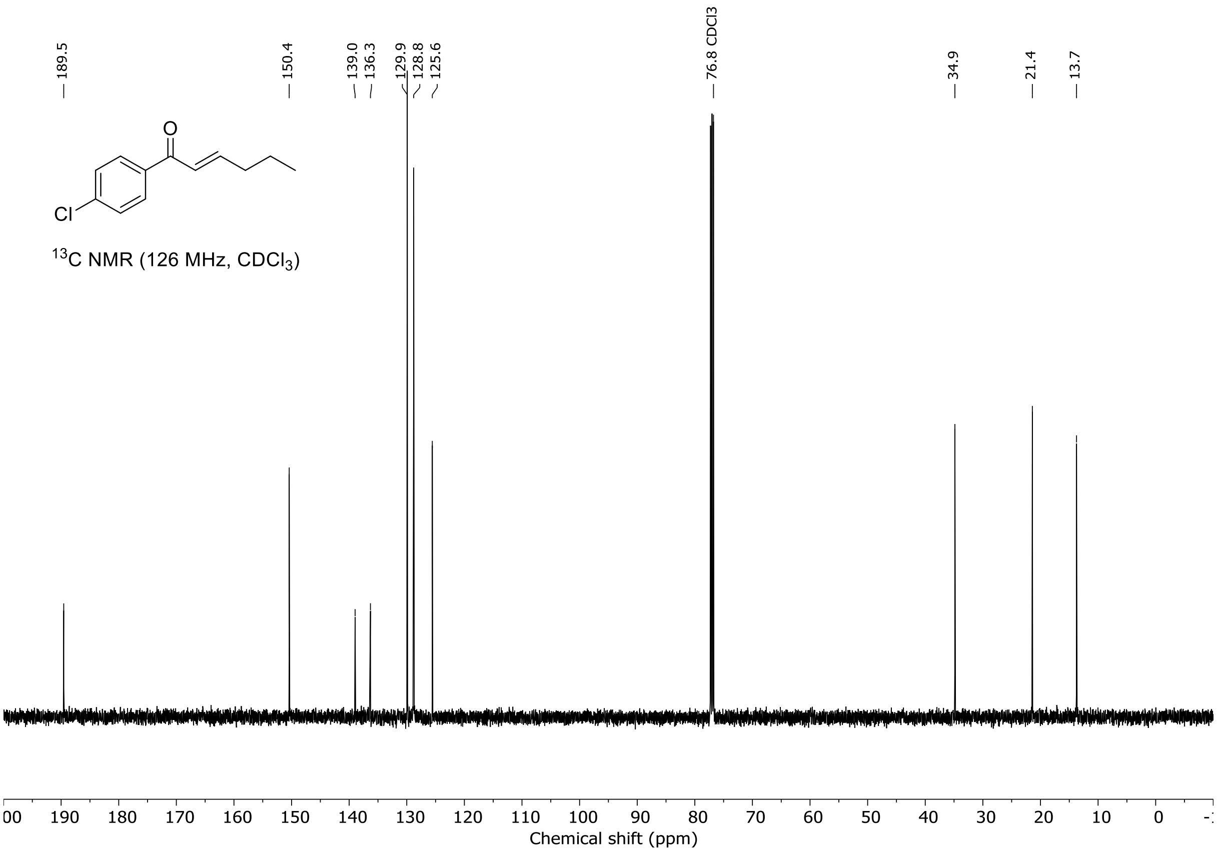

(*E*)-1-(4-bromophenyl)hex-2-en-1-one (E7)

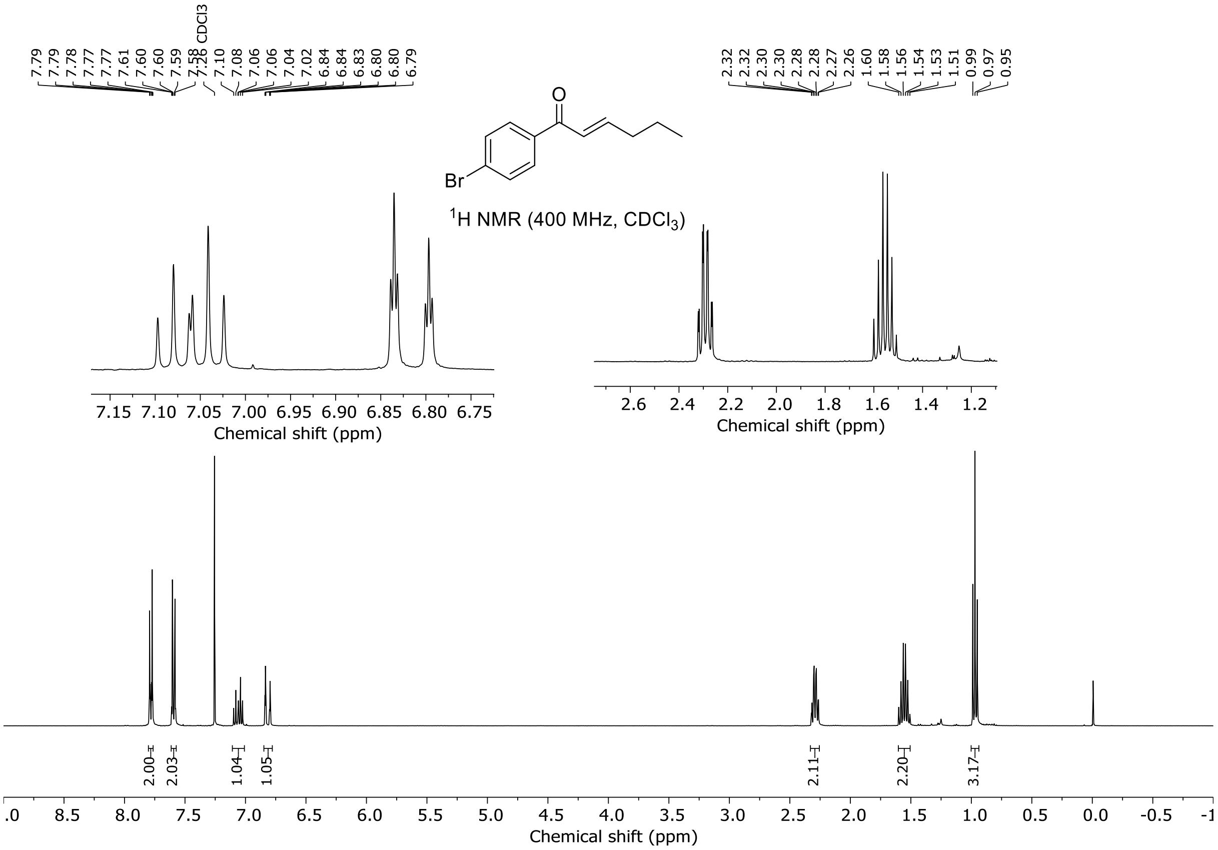

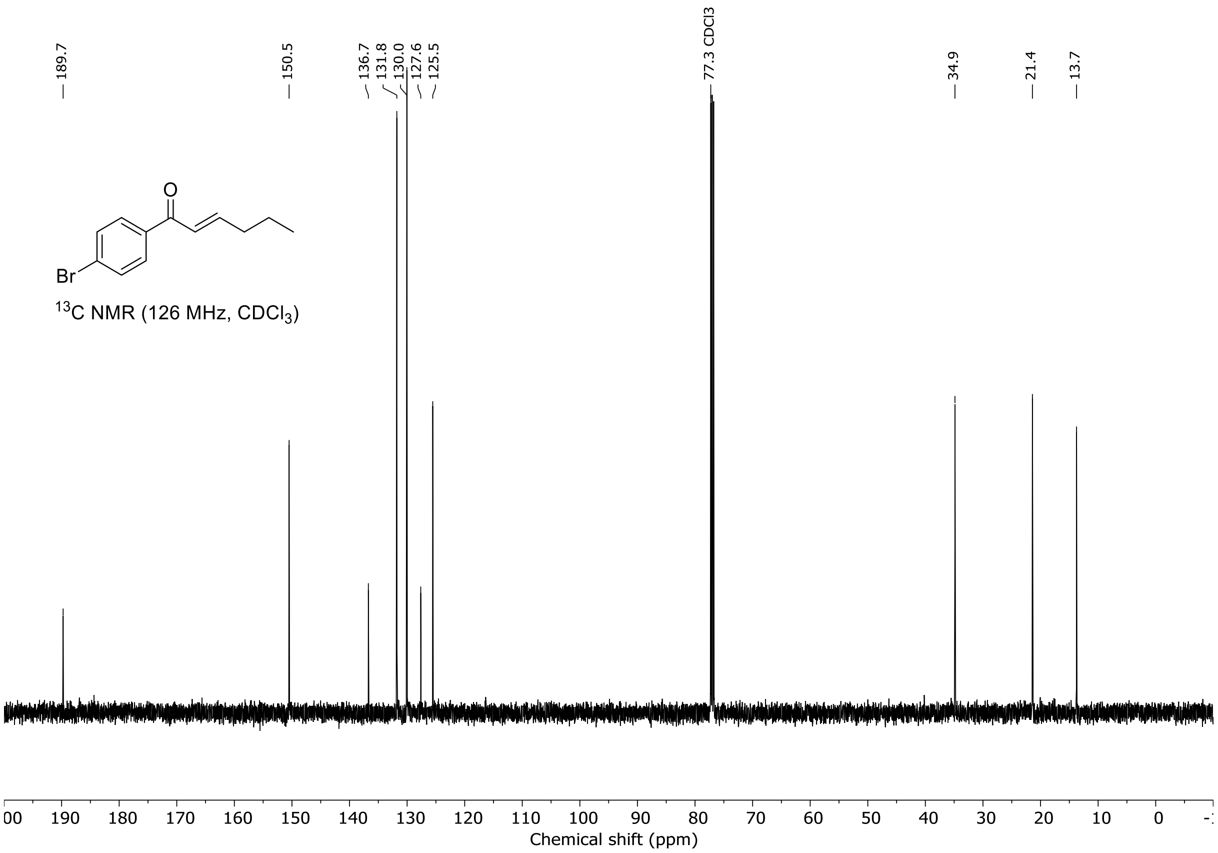

(*E*)-1-(4-nitrocyclohexa-1,3-dien-1-yl)hex-2-en-1-one (E9)

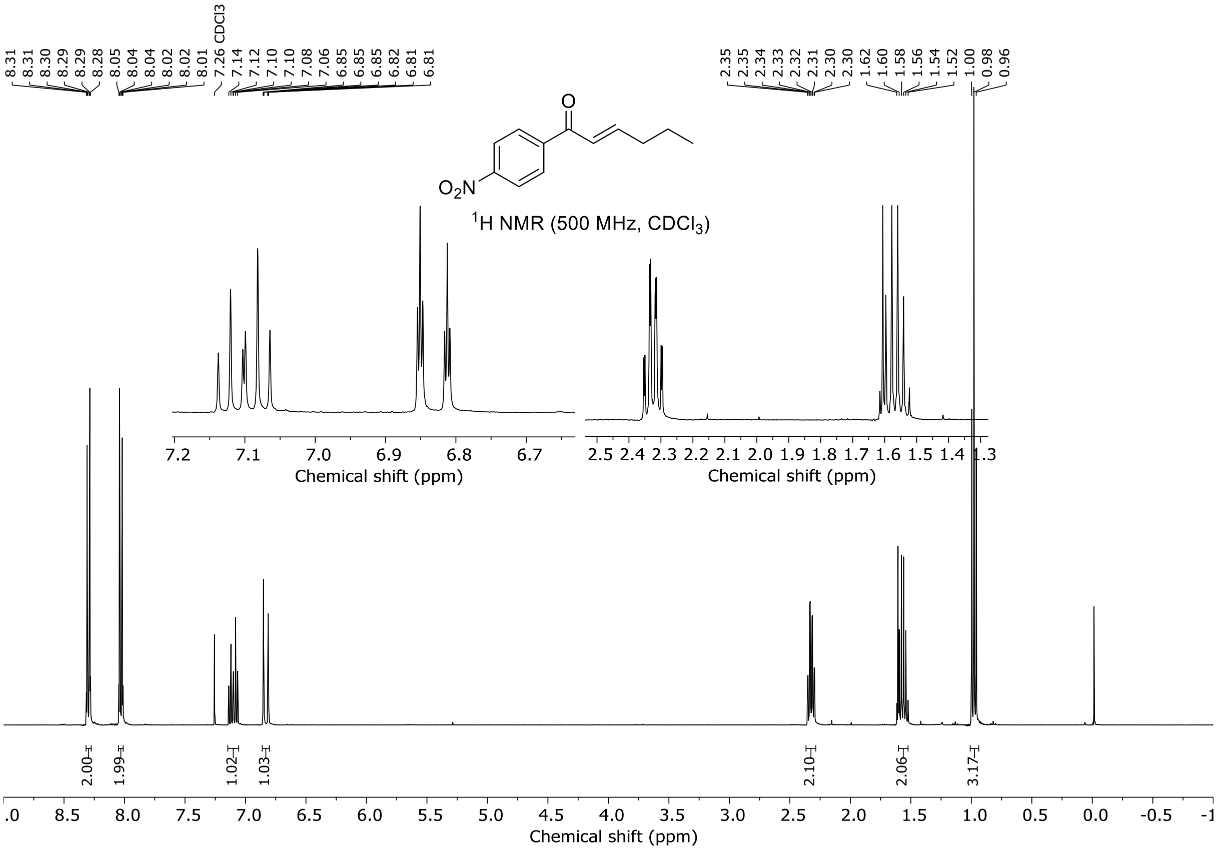

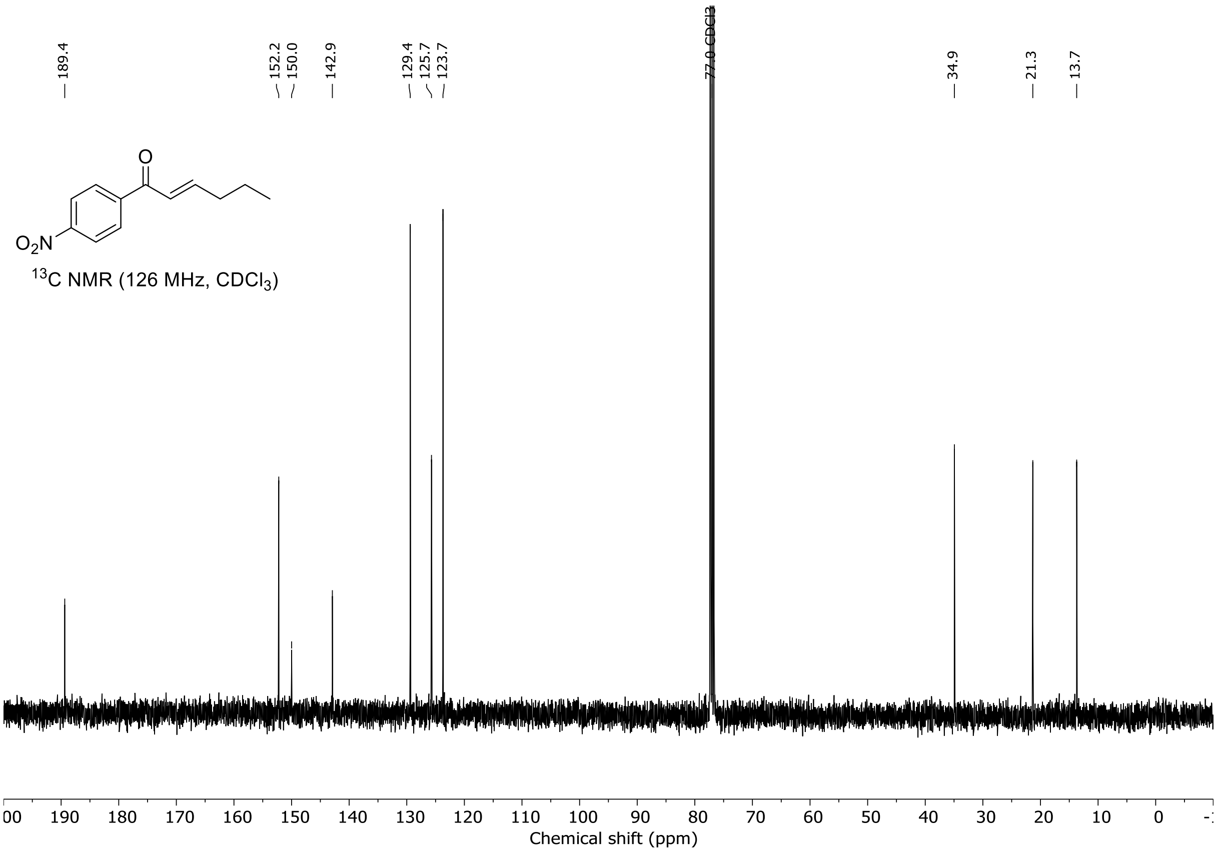

(*E*)-1-(4-acetylphenyl)hex-2-en-1-one (E10)

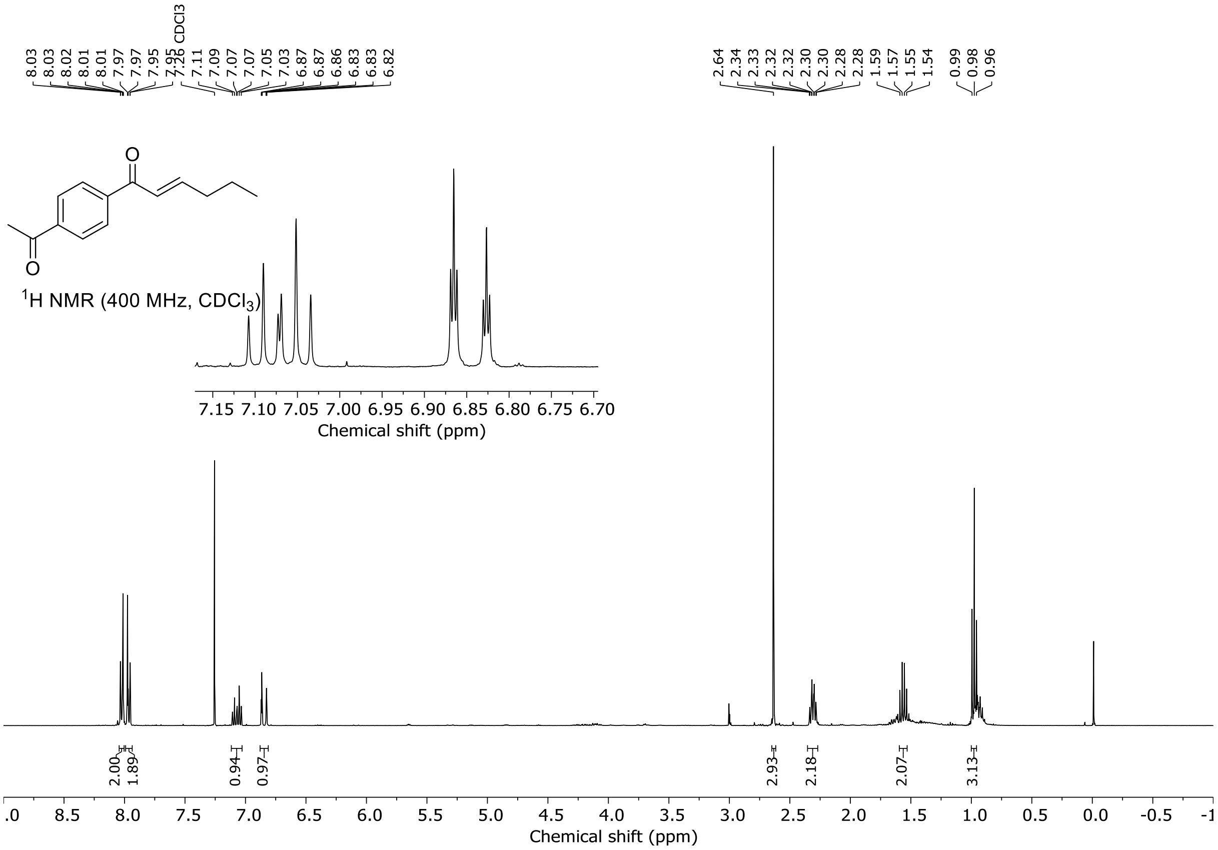

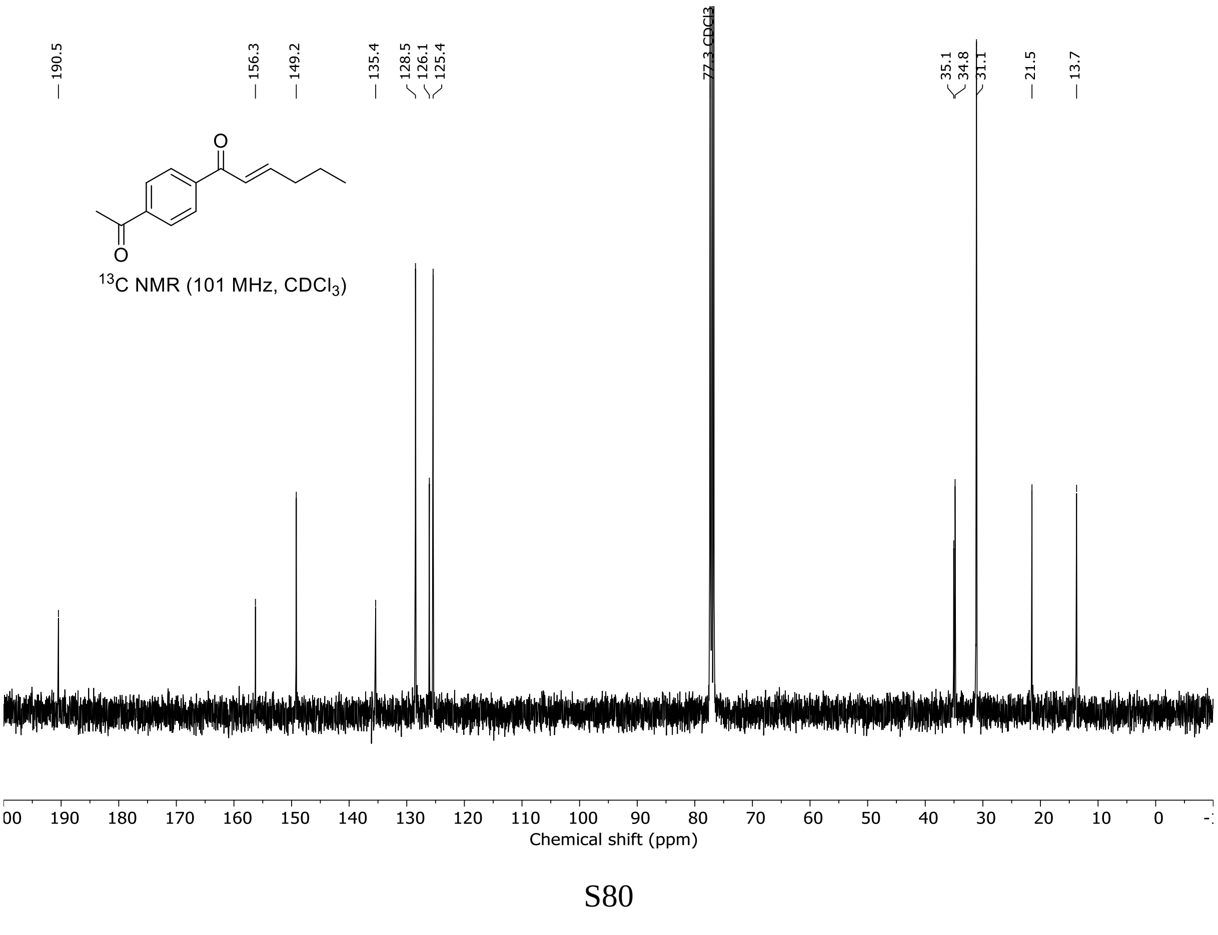

1,4-bis(4-methoxyphenyl)but-2-en-1-one (*Z*/*E* mixture) (E12)

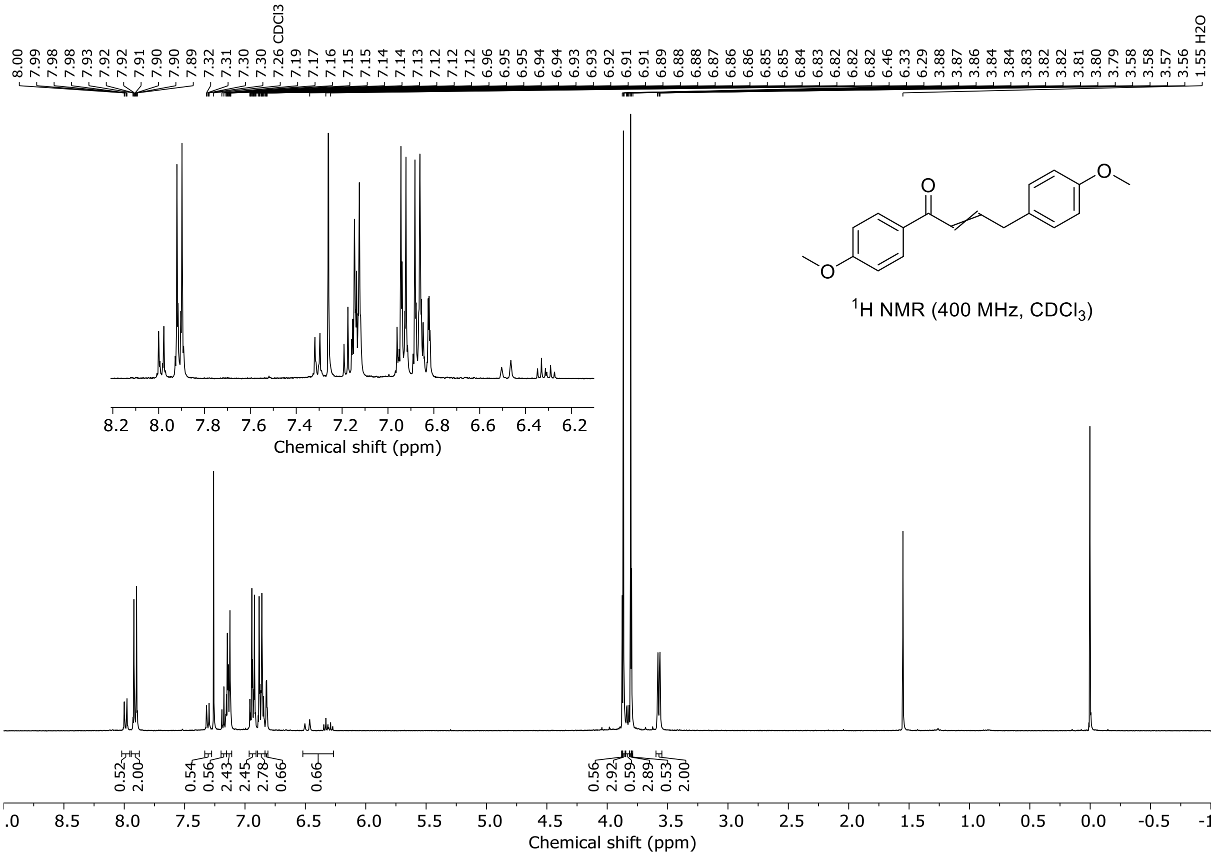

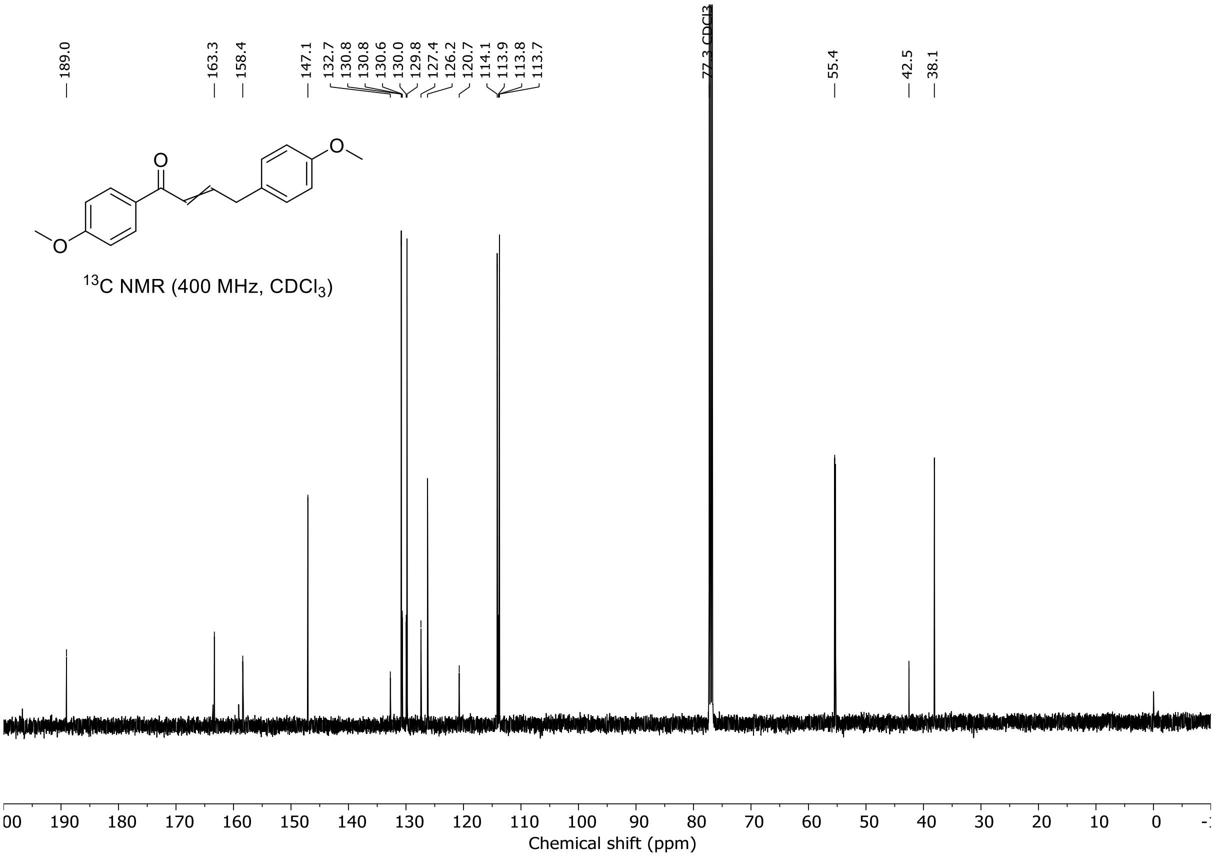

1-(4-methoxyphenyl)-4-(4-(trifluoromethyl)phenyl)but-2-en-1-one (*Z*/*E* mixture) (E13)

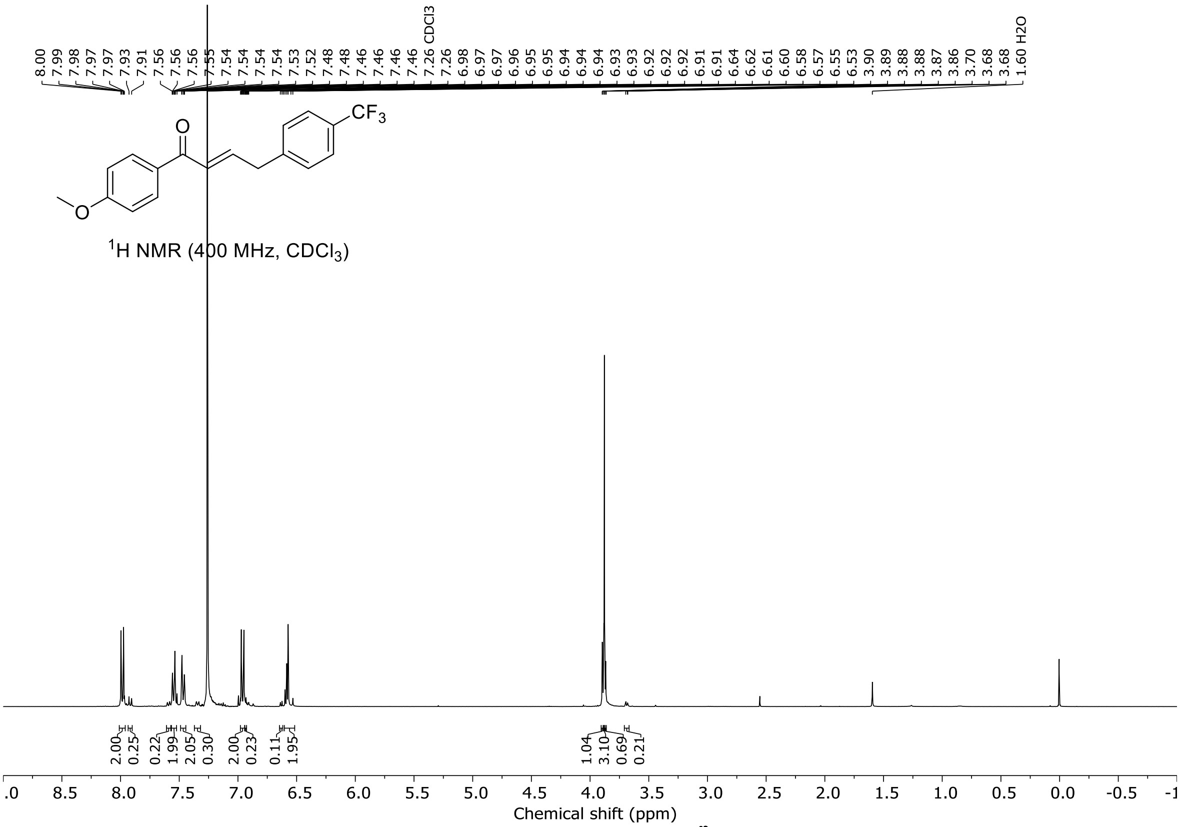

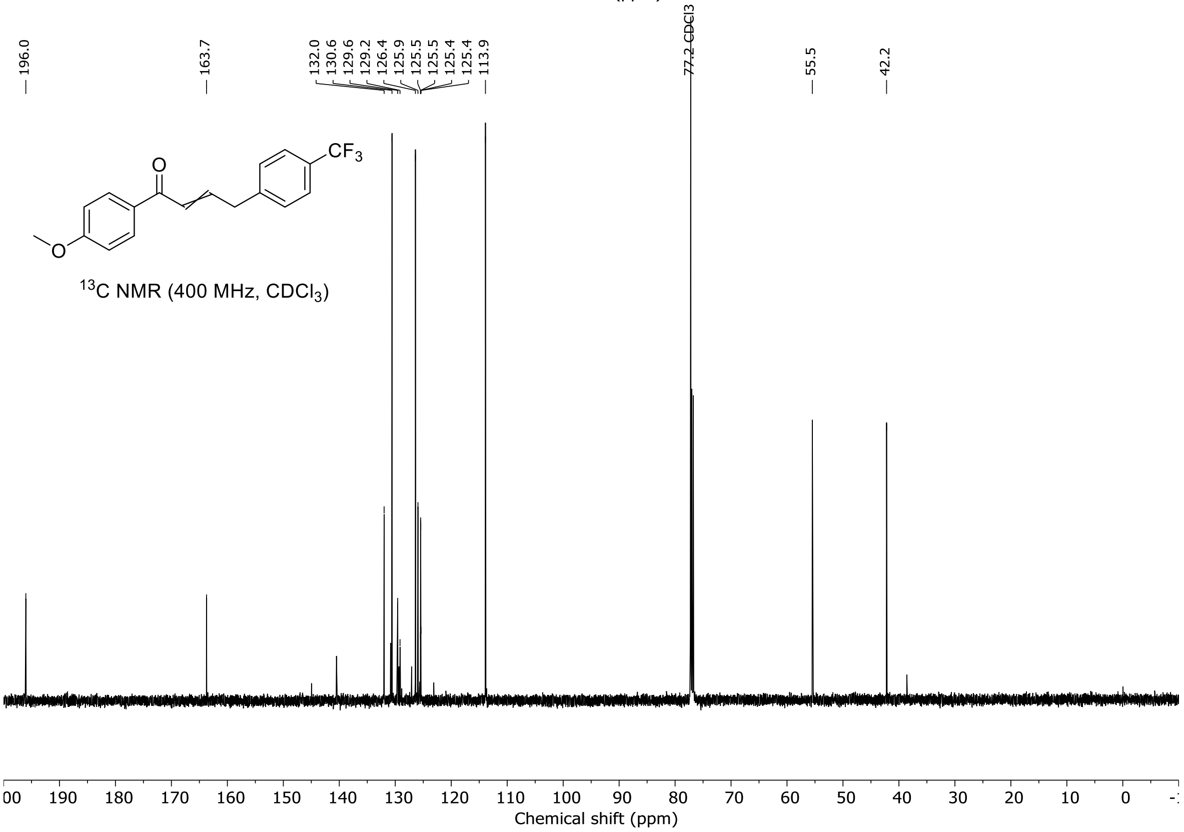

(*E*)-1,4-bis(4-(trifluoromethyl)phenyl)but-2-en-1-one (E14)

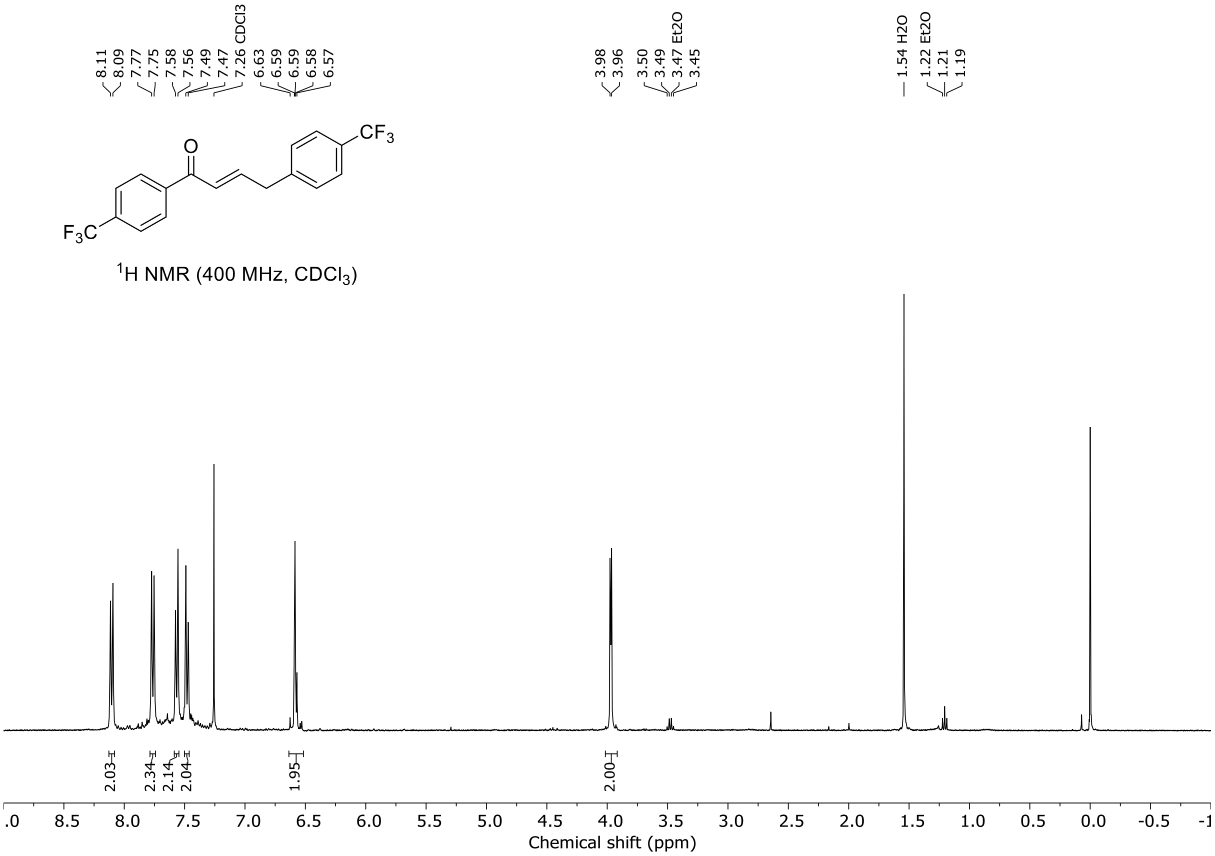

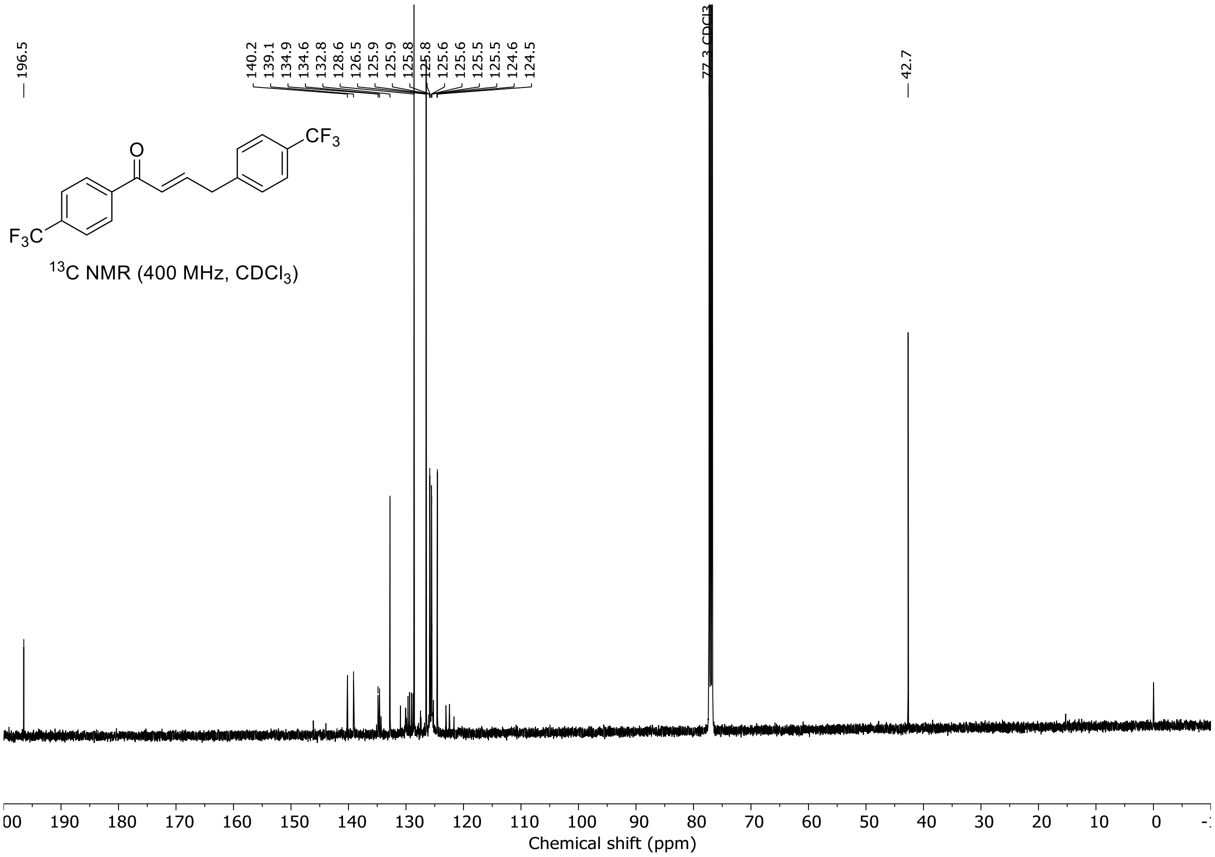

(*E*)-4-(4-methoxyphenyl)-1-(4-(trifluoromethyl)phenyl)but-2-en-1-one (E15)

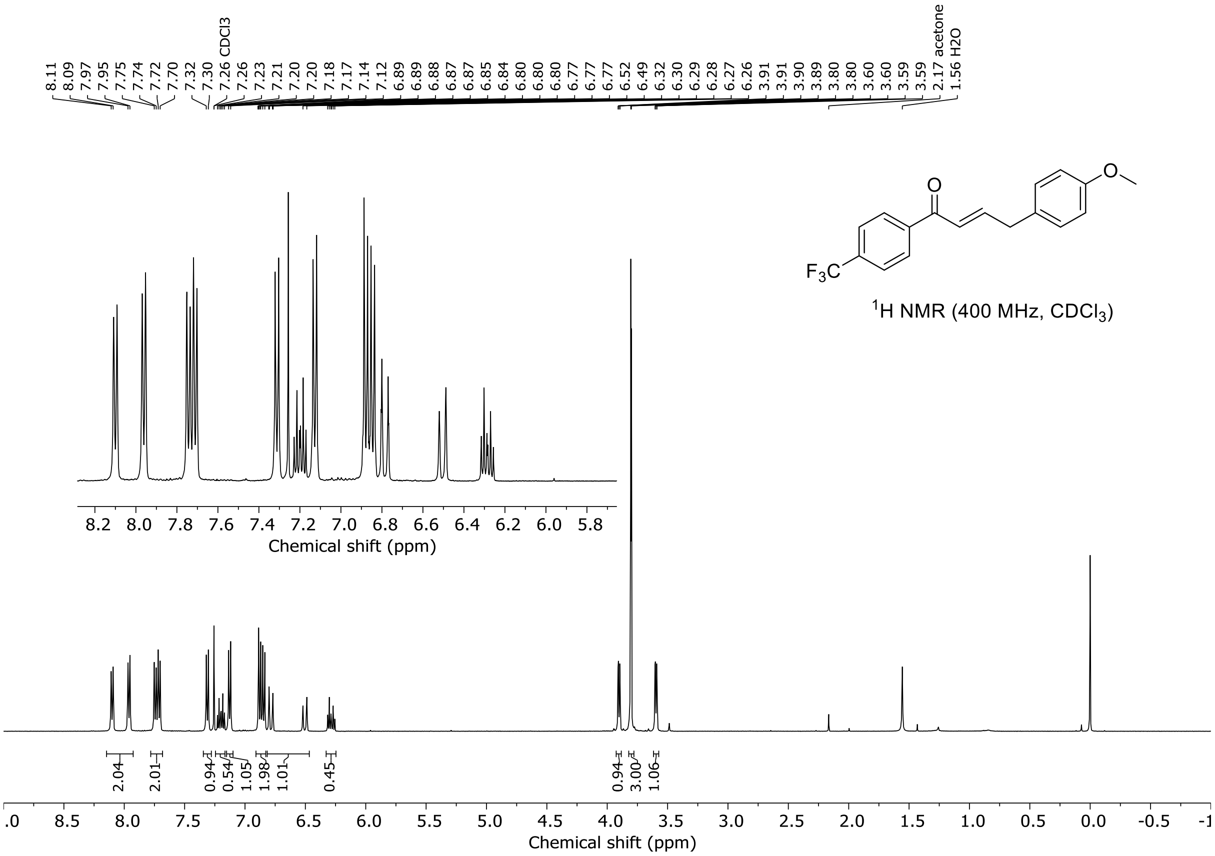

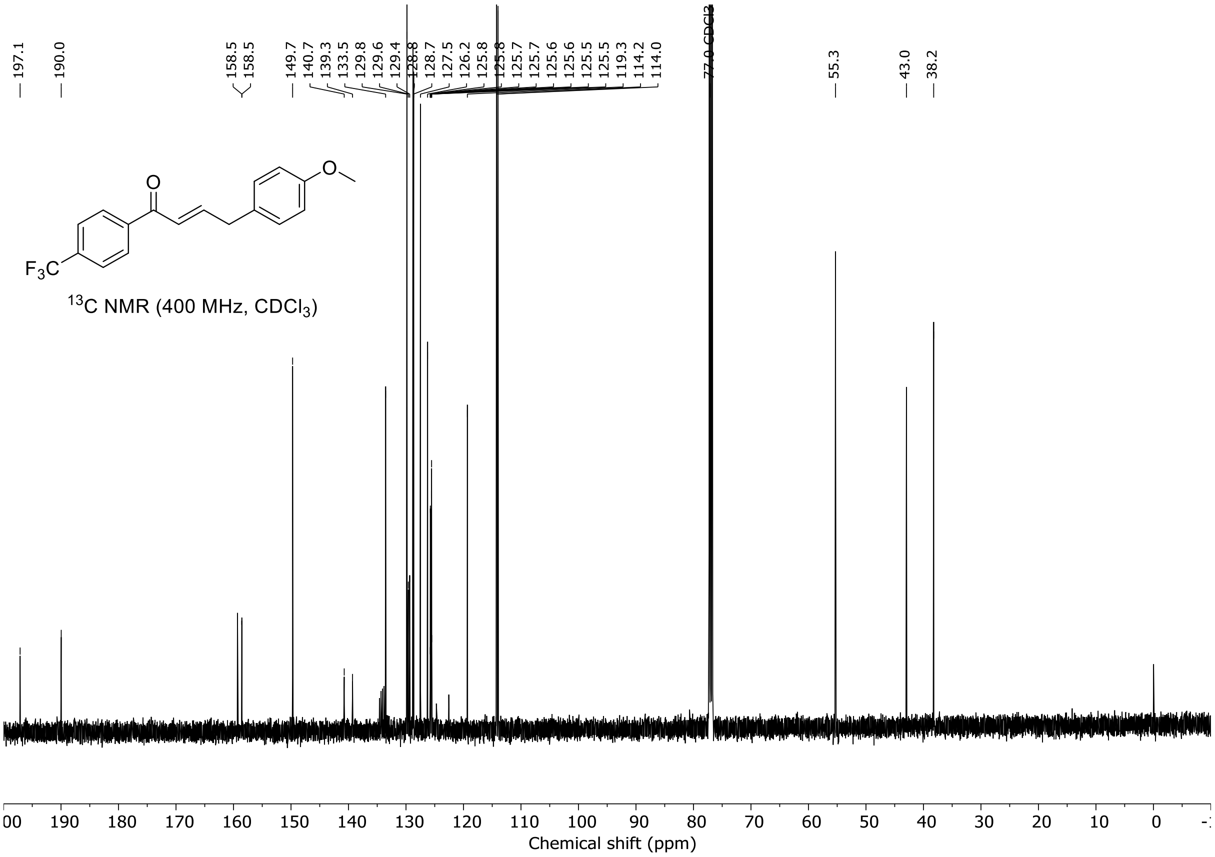

tert-butyldimethyl((1-phenylbuta-1,3-dien-1-yl)oxy)silane (Z/E mixture) (S1)

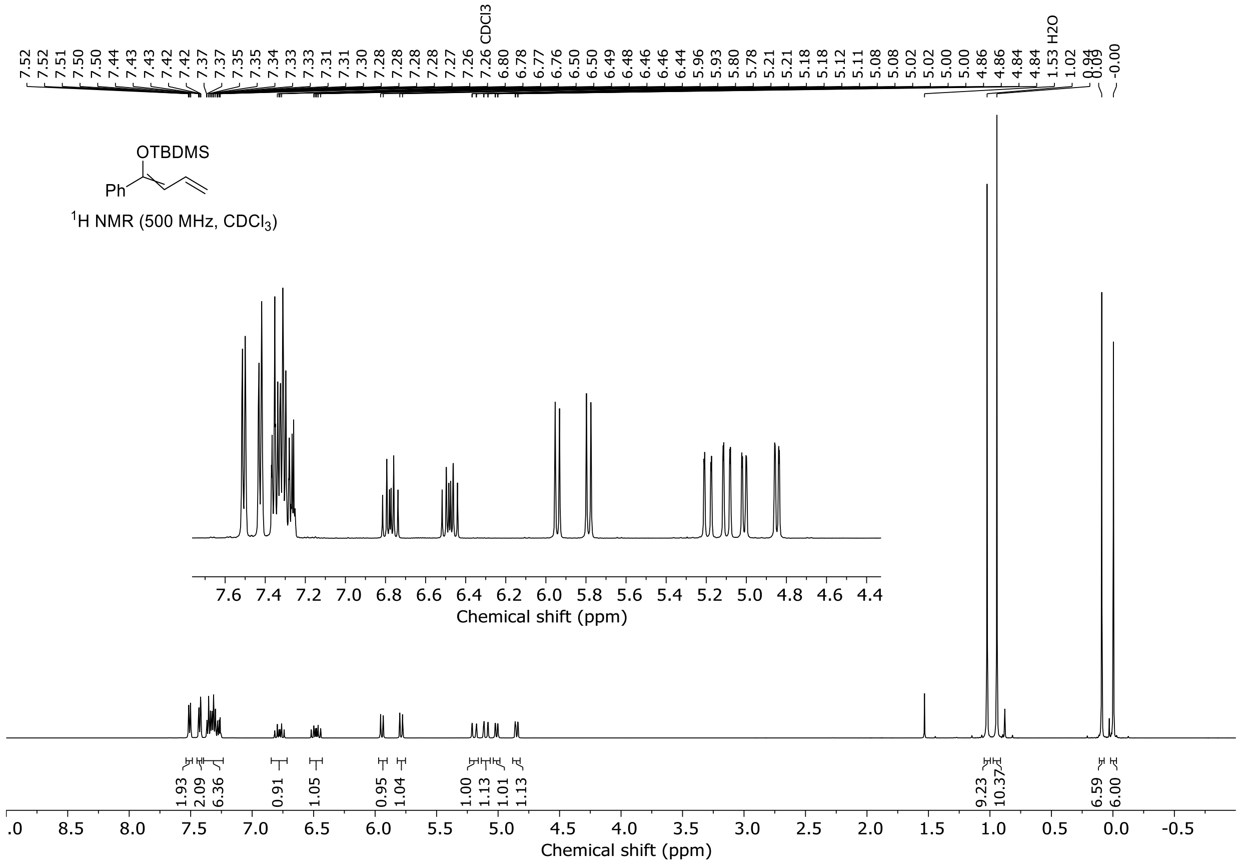

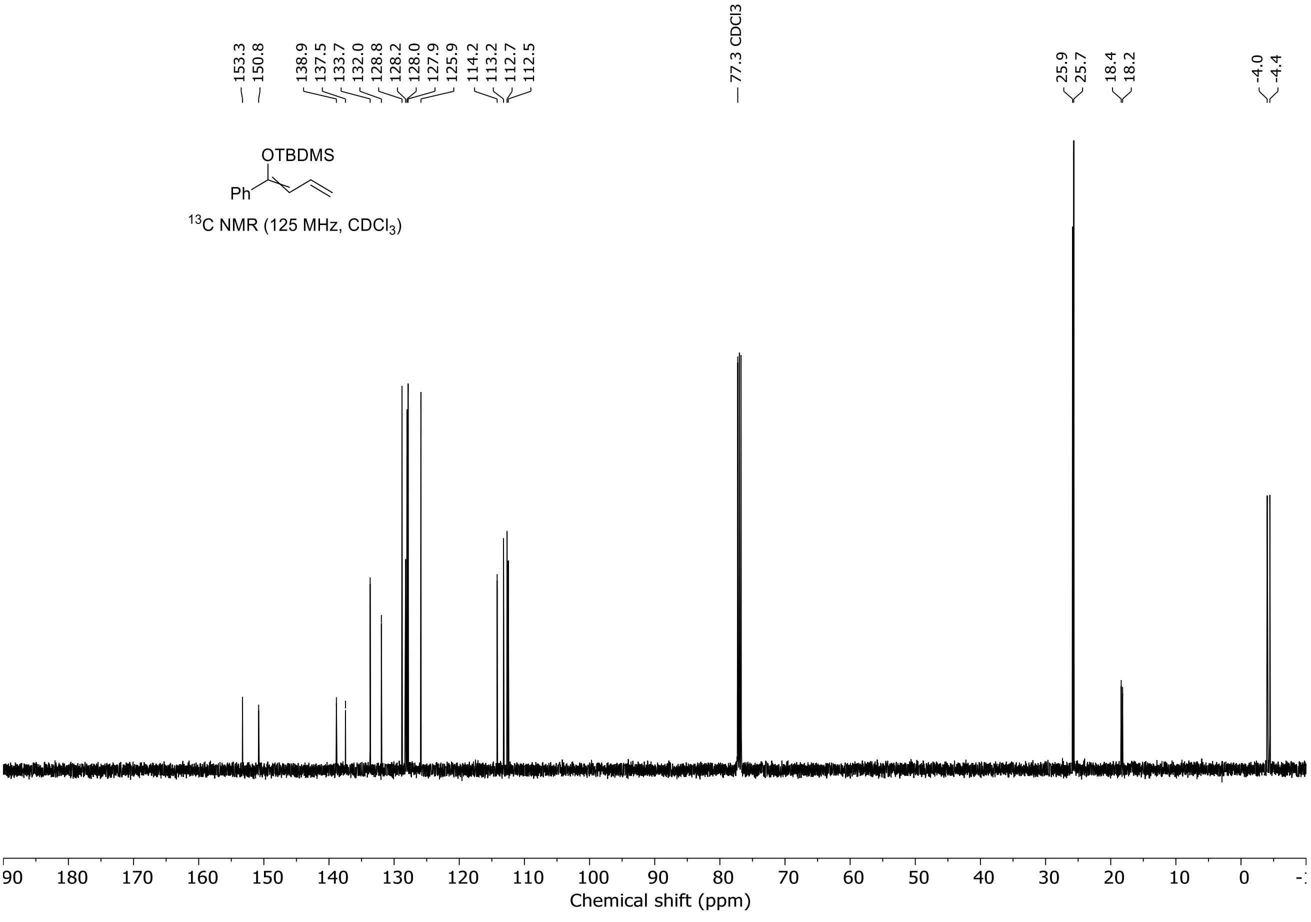

tert-butyldimethyl((1-phenylhexa-1,3,5-trien-1-yl)oxy)silane (_Z_/_E_ mixture) (S2)

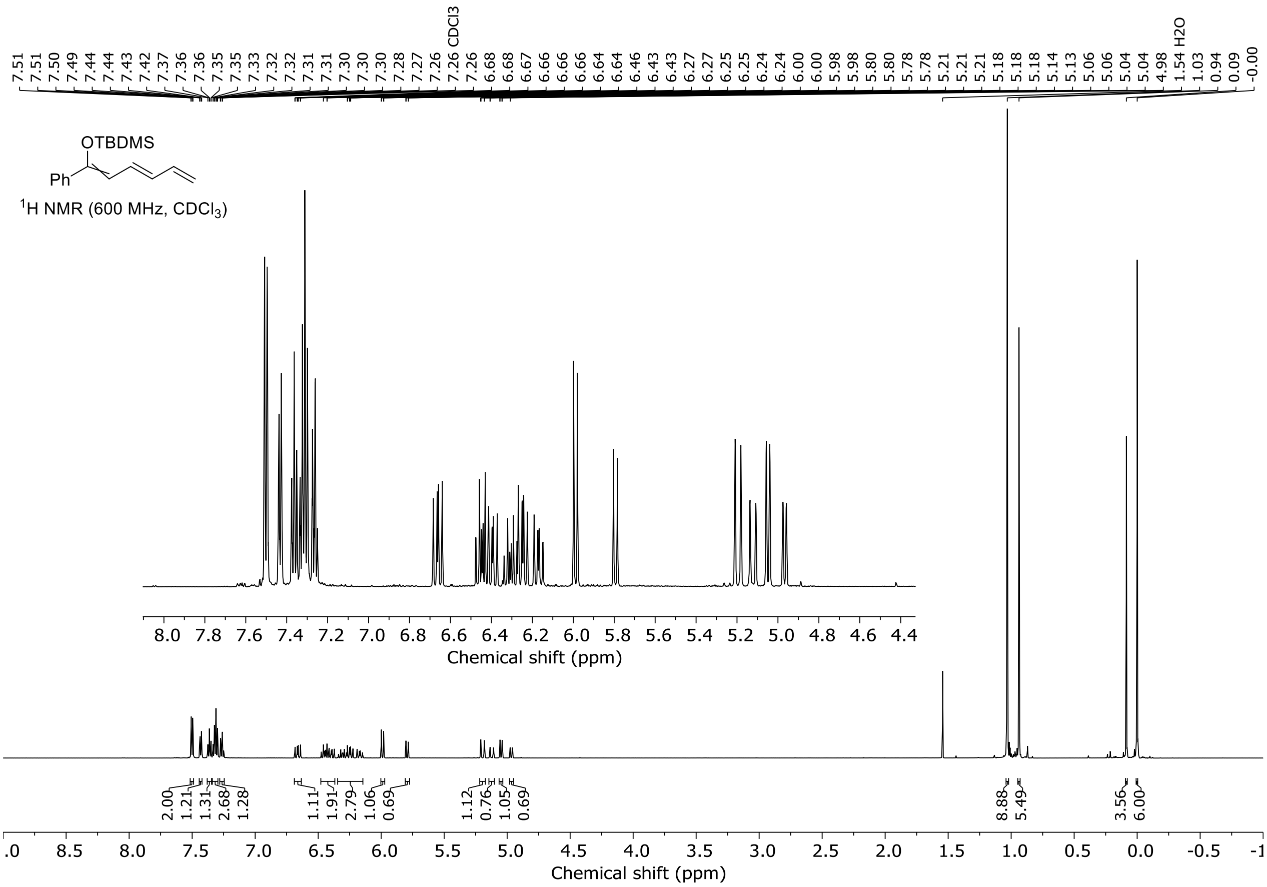

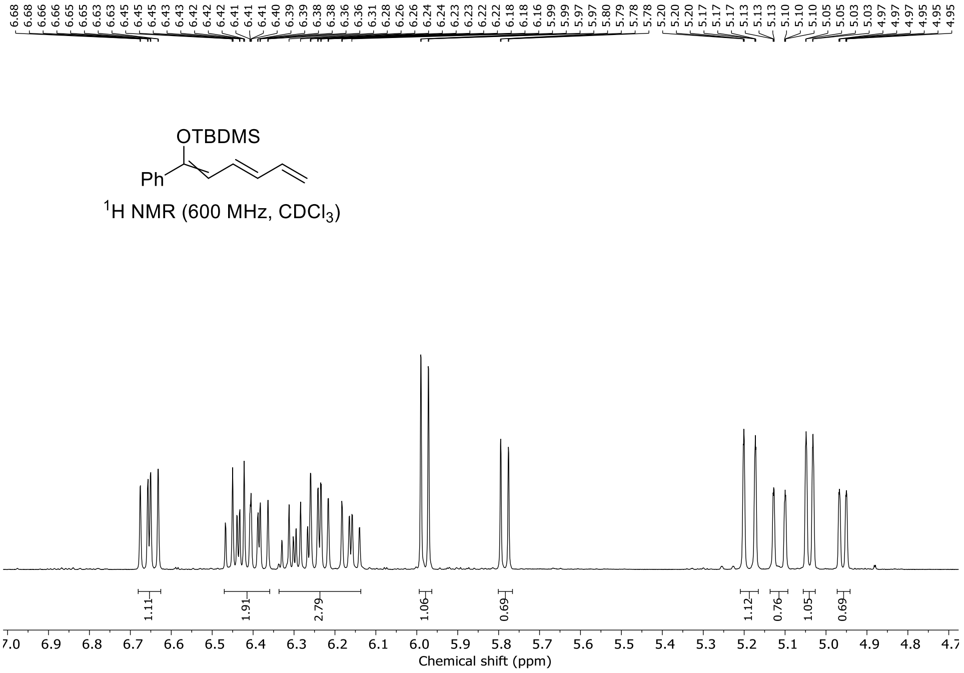

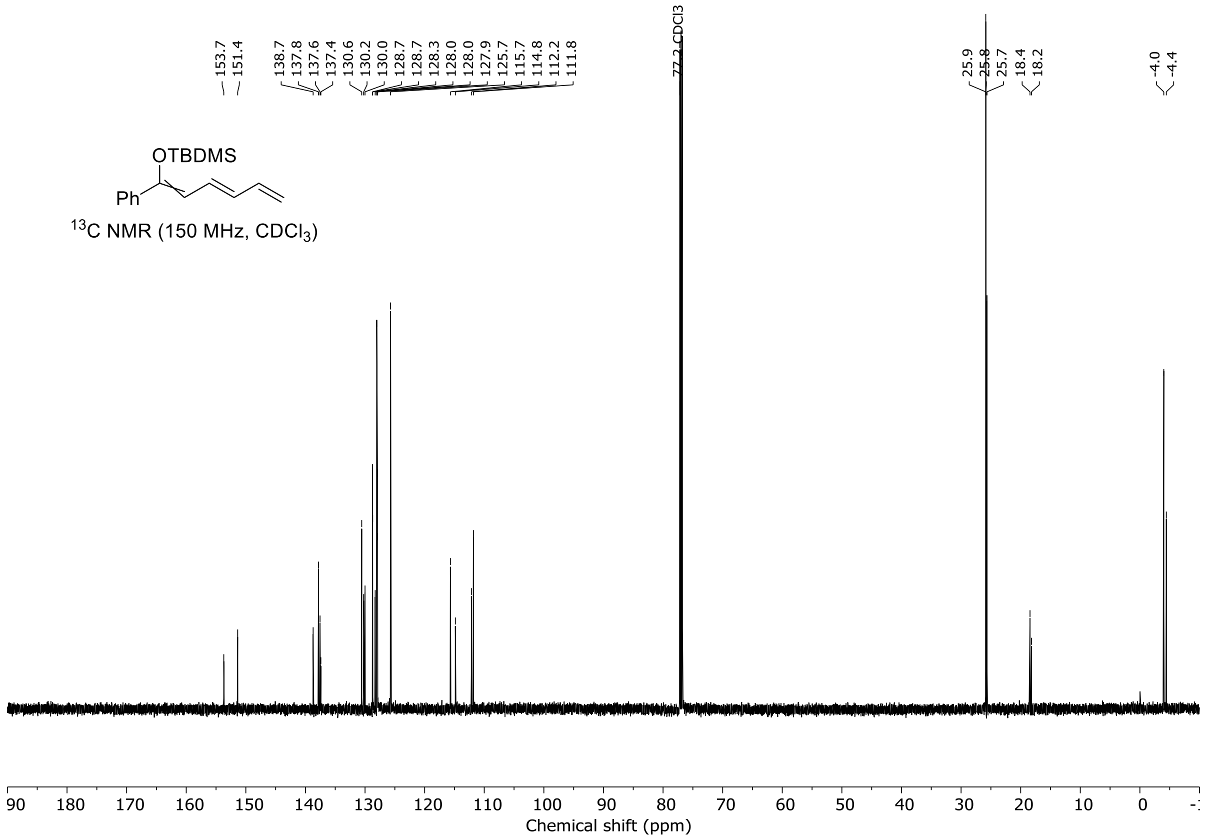
OTBDMS
Ph
13C NMR (150 MHz, CDCl3)
153.7
151.4
138.7
137.8
137.6
137.4
130.6
130.2
130.0
128.7
128.7
128.3
128.0
128.0
127.9
125.7
115.7
114.8
112.2
111.8
77.2 CDCl3
25.9
25.8
25.7
18.4
18.2
-4.0
-4.4
Chemical shift (ppm)

***tert*-butyldimethyl((1-phenylocta-1,3,5,7-tetraen-1-yl)oxy)silane (S3, crude)**

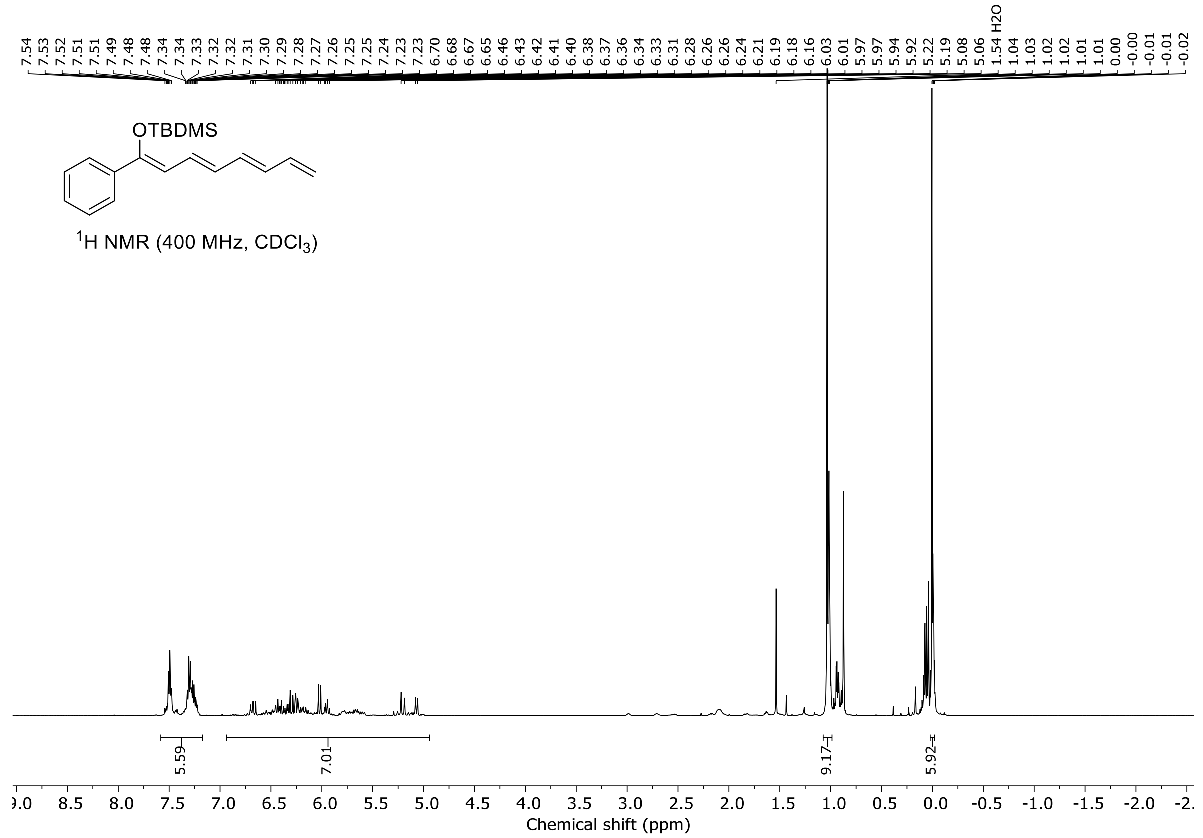

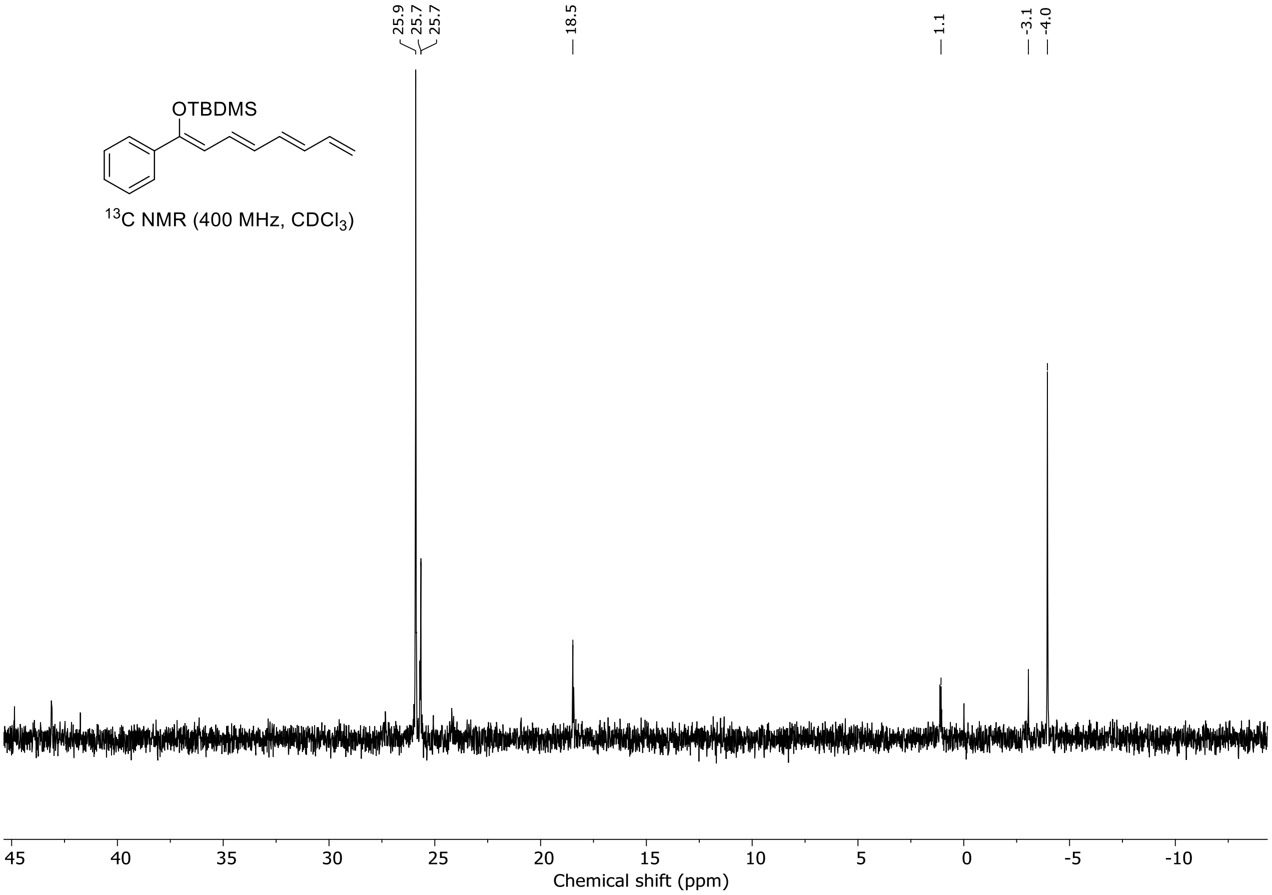

***tert*-butyl(((1-(4-methoxyphenyl)hexa-1,3-dien-1-yl)oxy)dimethylsilane (S4, crude)**

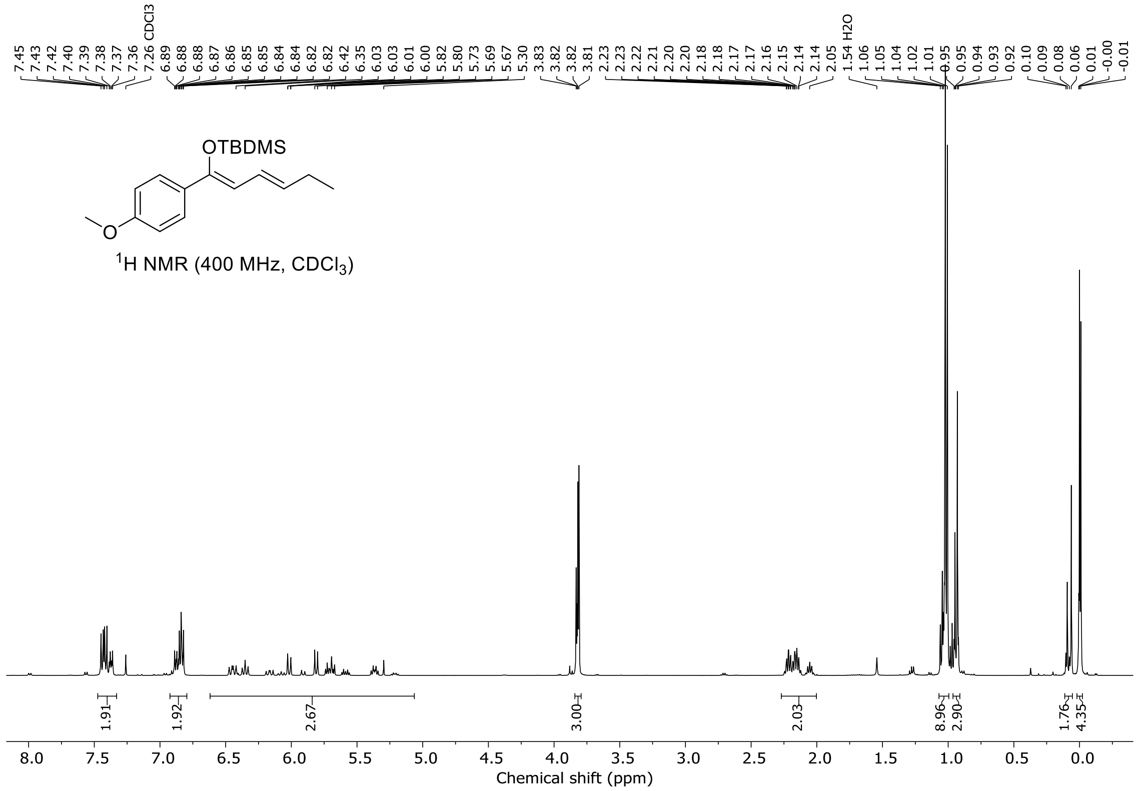

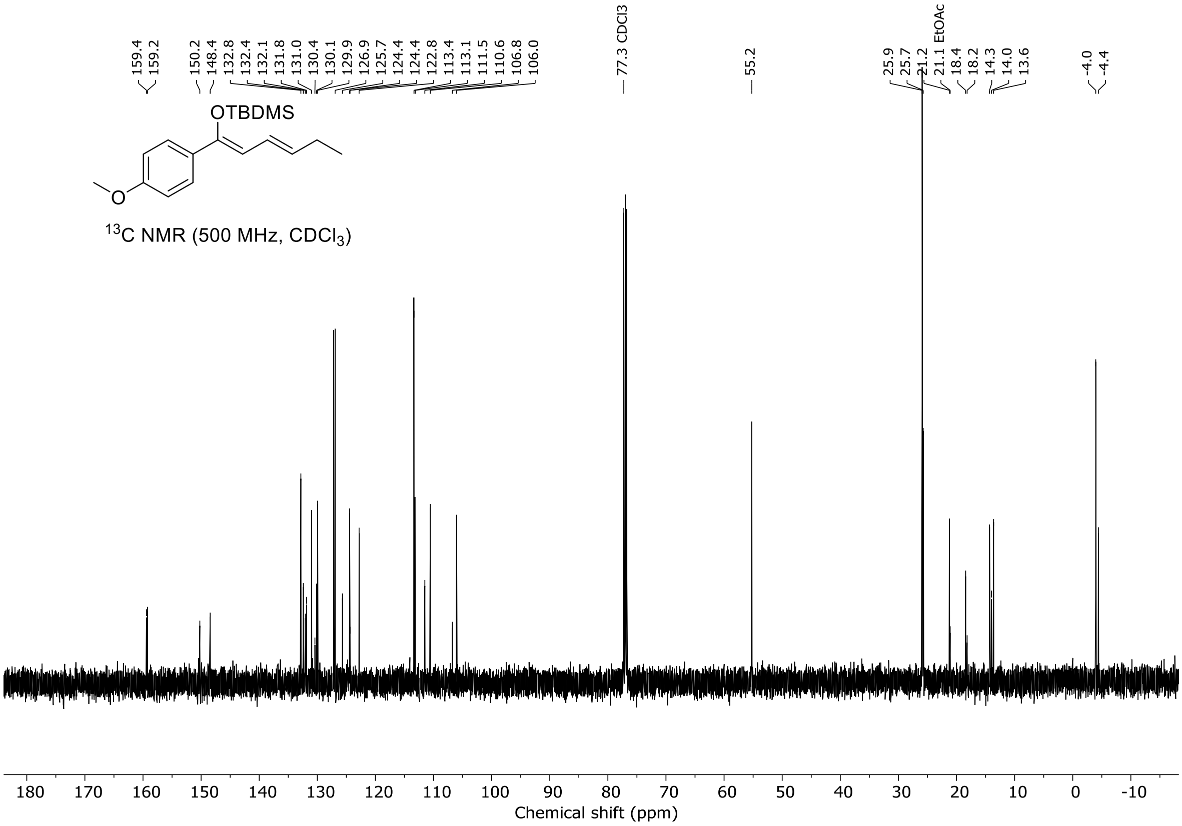

tert-butyl((1-(4-(tert-butyl)phenyl)hexa-1,3-dien-1-yl)oxy)dimethylsilane (S5, crude)

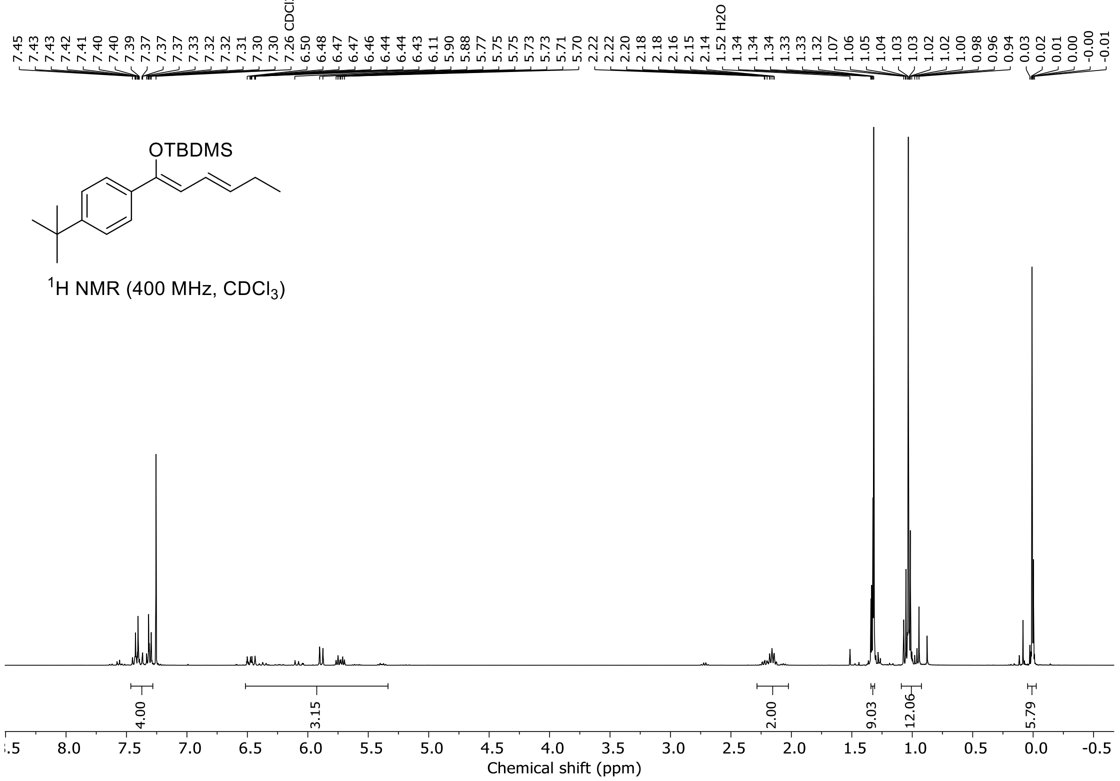

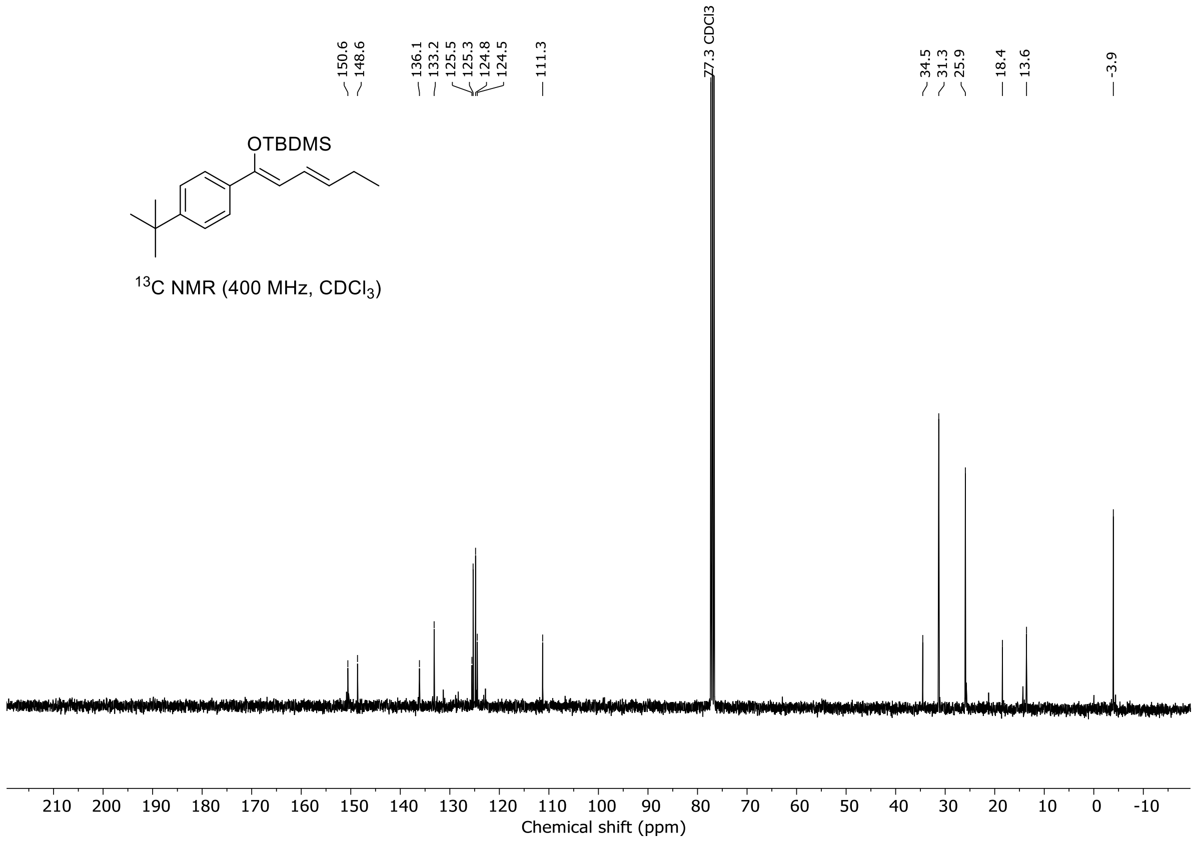

tert-butyl((1-(4-chlorophenyl)hexa-1,3-dien-1-yl)oxy)dimethylsilane (S6, crude)

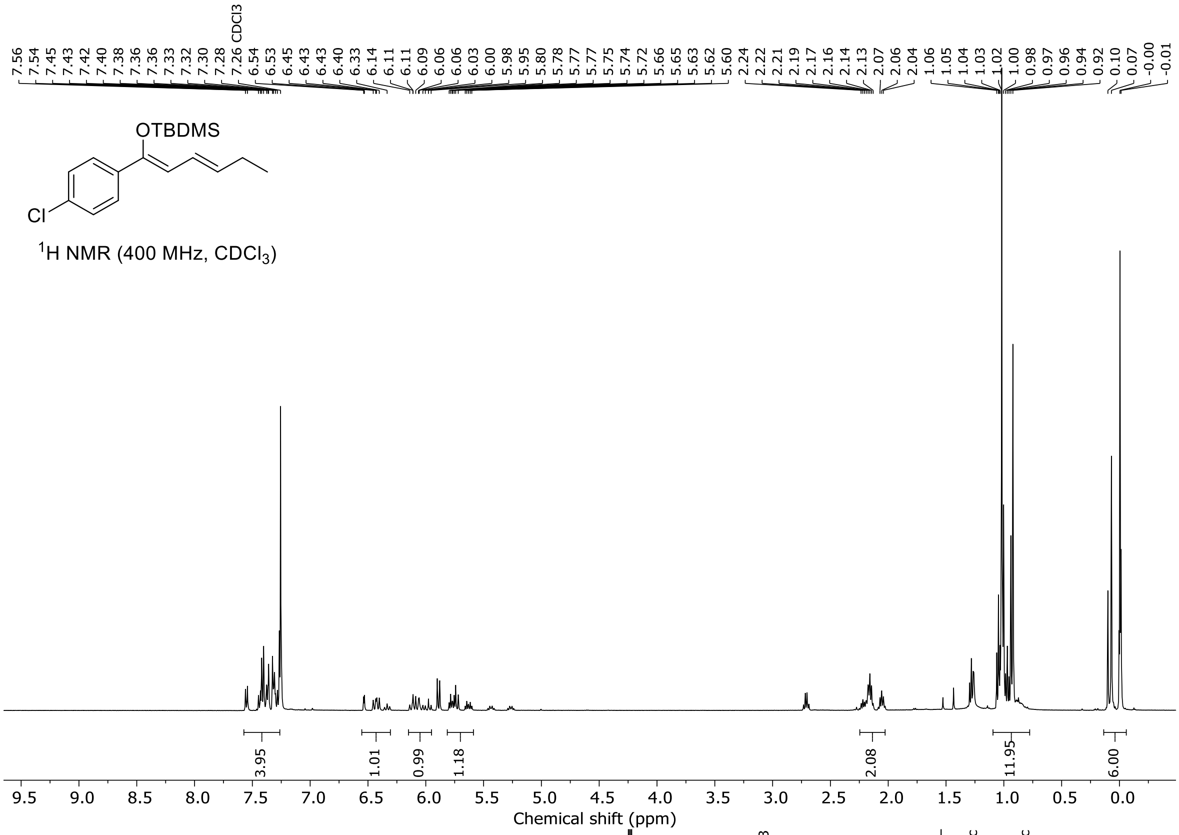

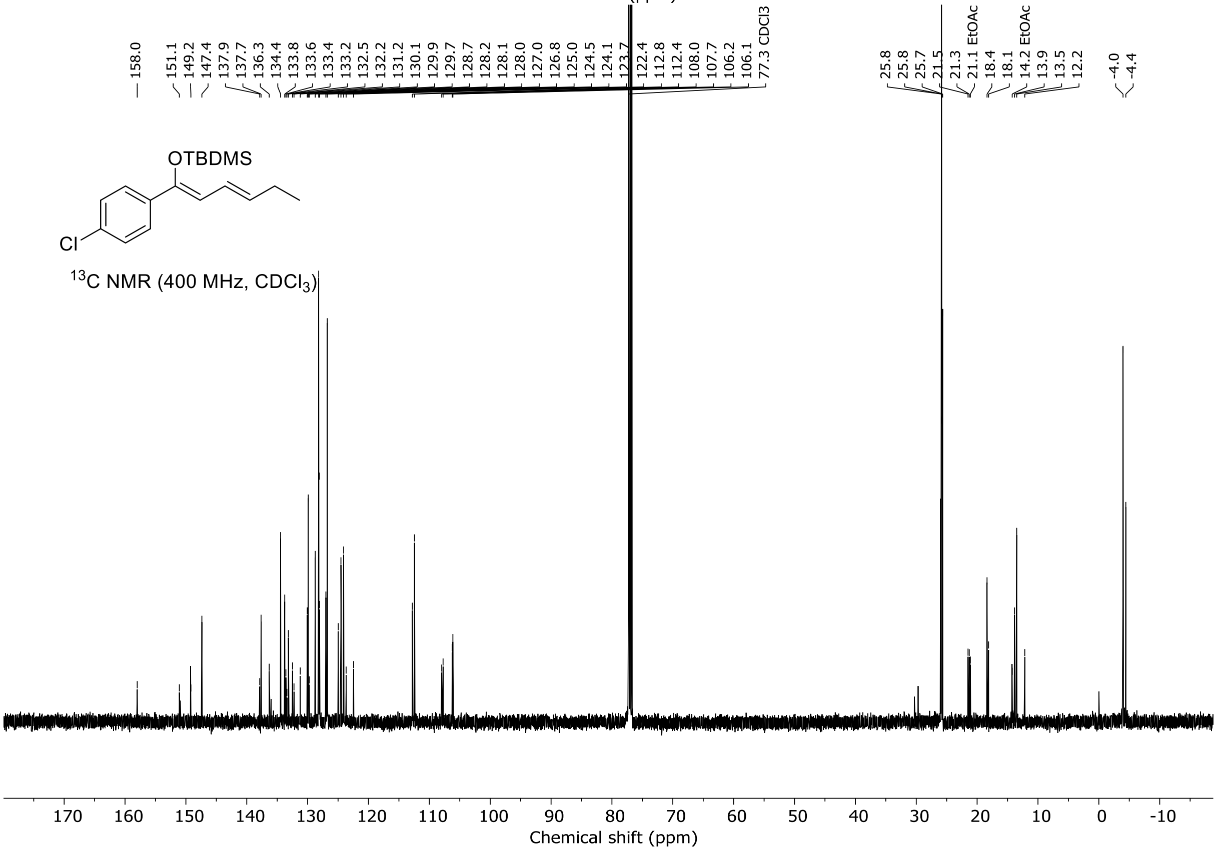

((1-(4-bromophenyl)hexa-1,3-dien-1-yl)oxy)(tert-butyl)dimethylsilane (S7, crude)

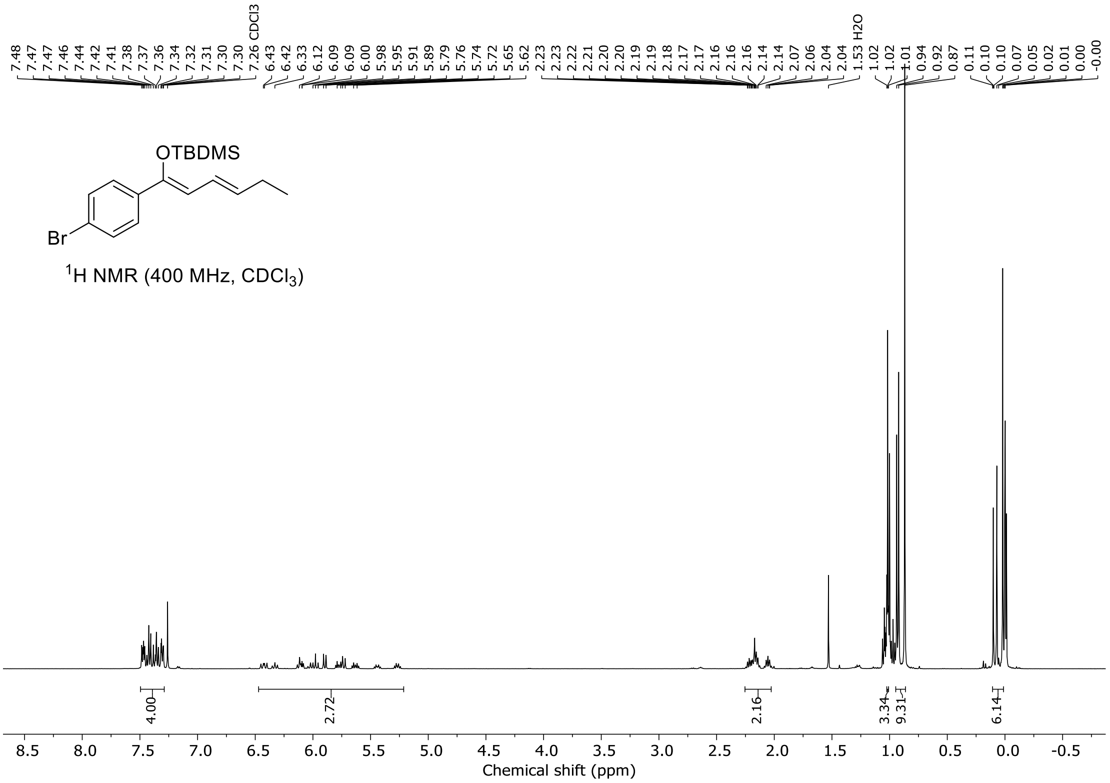

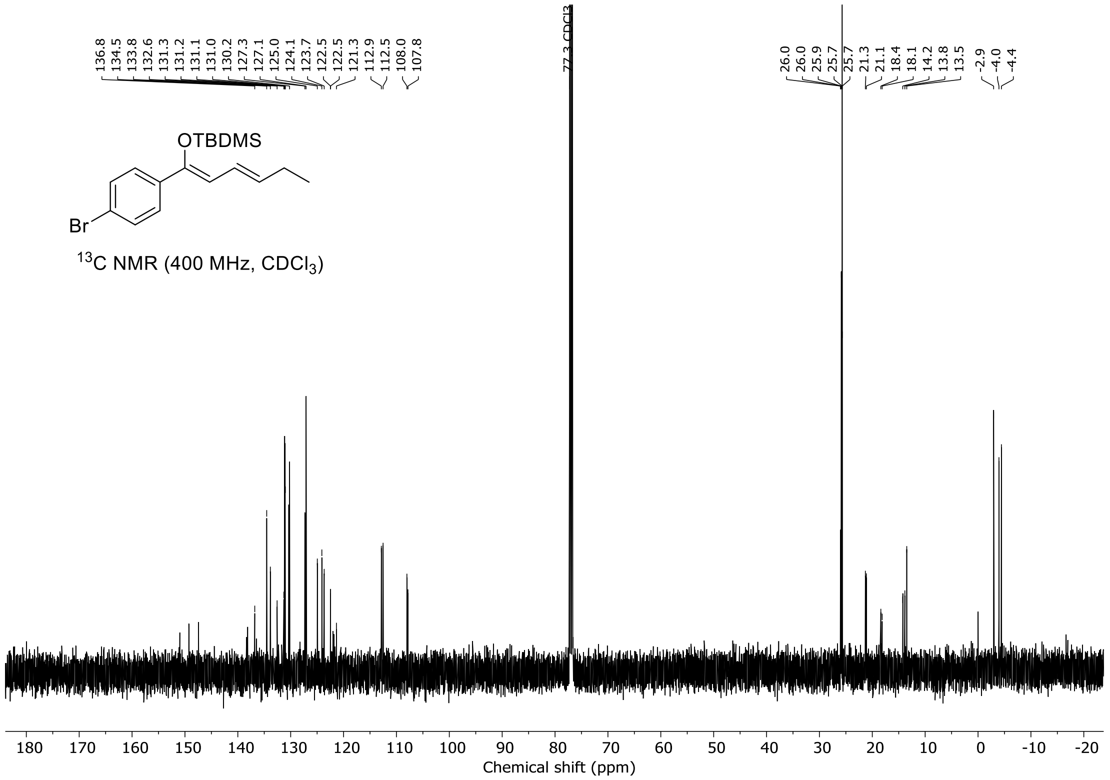

((1-(benzo[d][1,3]dioxol-5-yl)hexa-1,3-dien-1-yl)oxy)(tert-butyl)dimethylsilane (S8, crude)

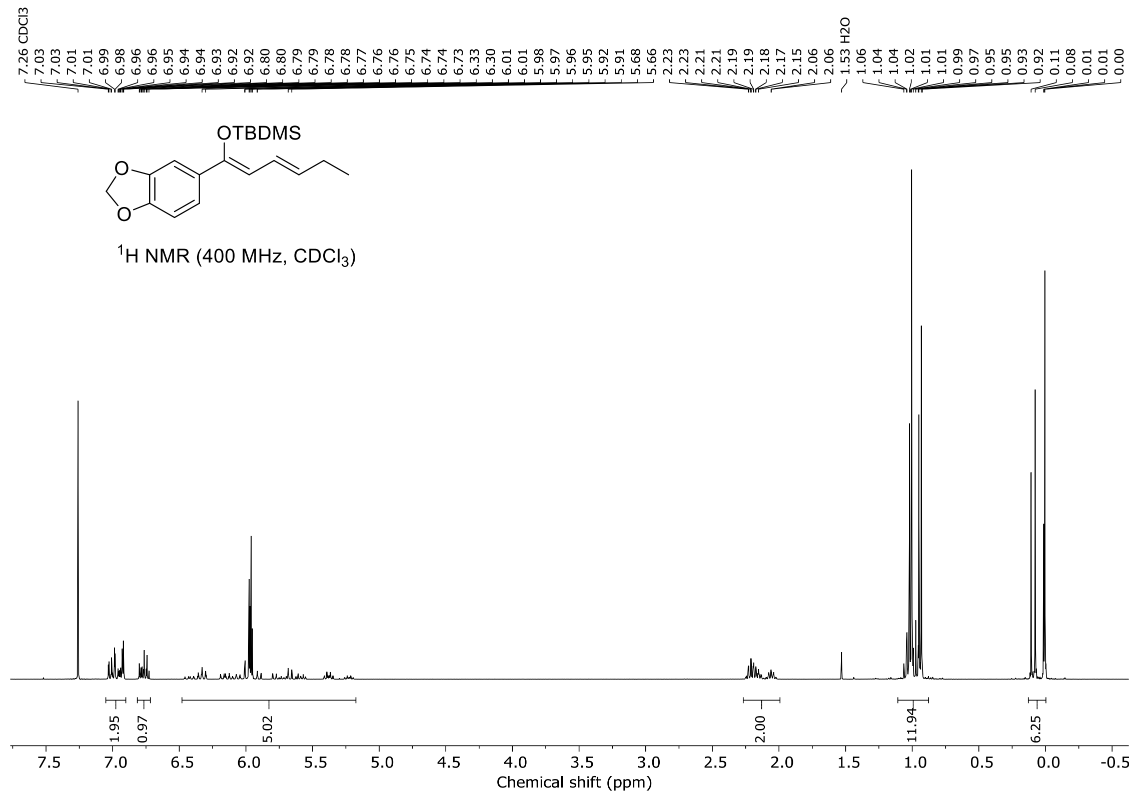

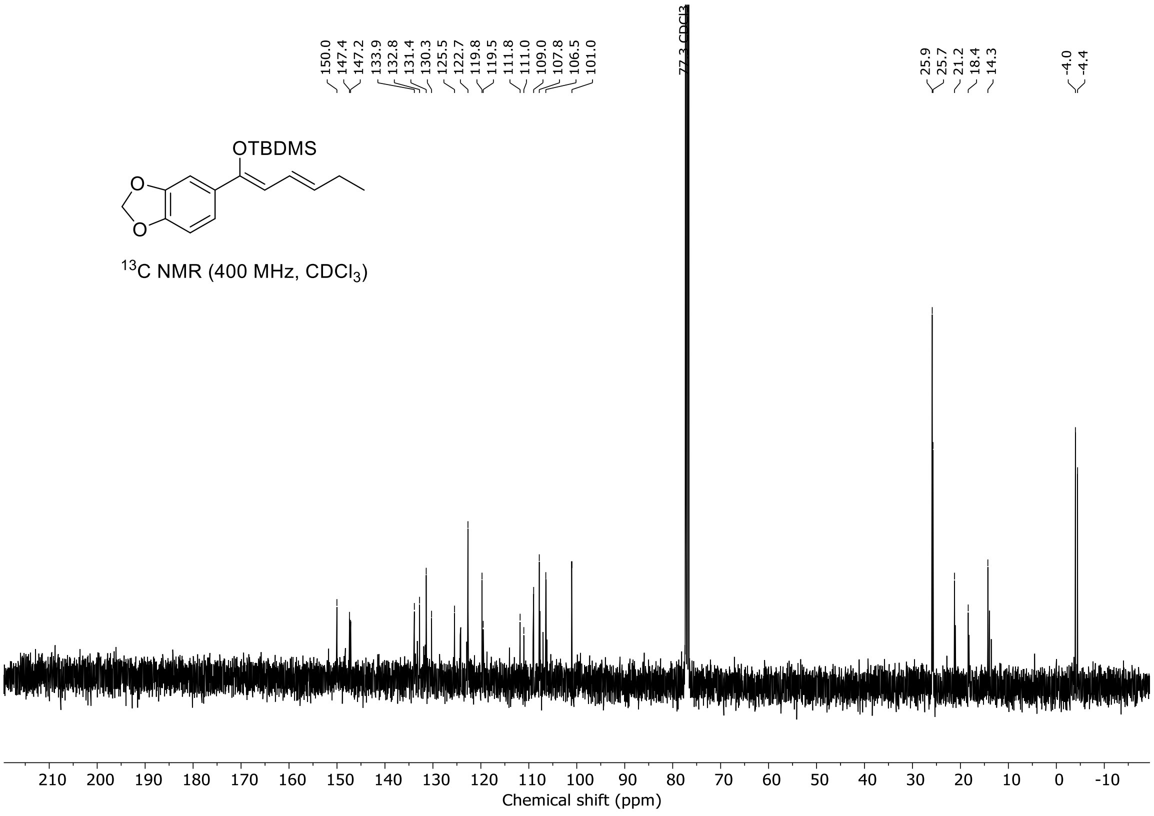

tert-butyldimethyl((1-(4-nitrophenyl)hexa-1,3-dien-1-yl)oxy)silane (S9, crude)

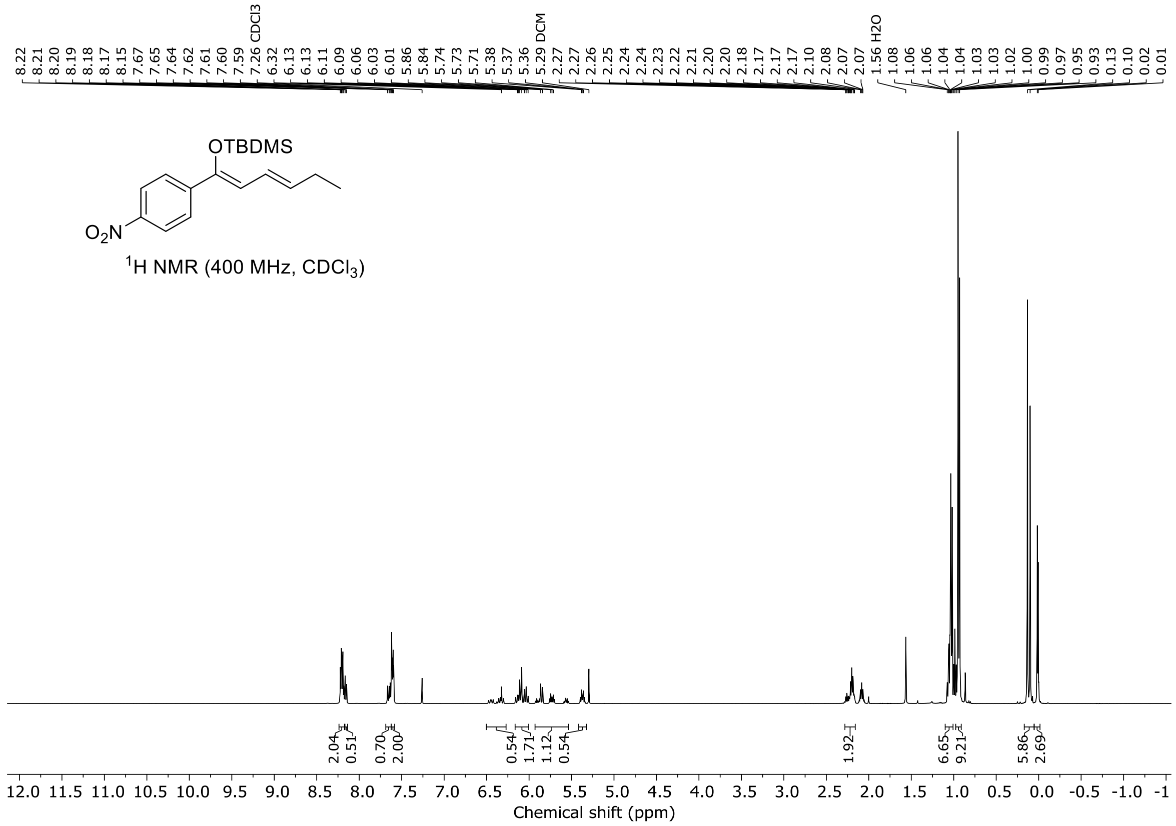

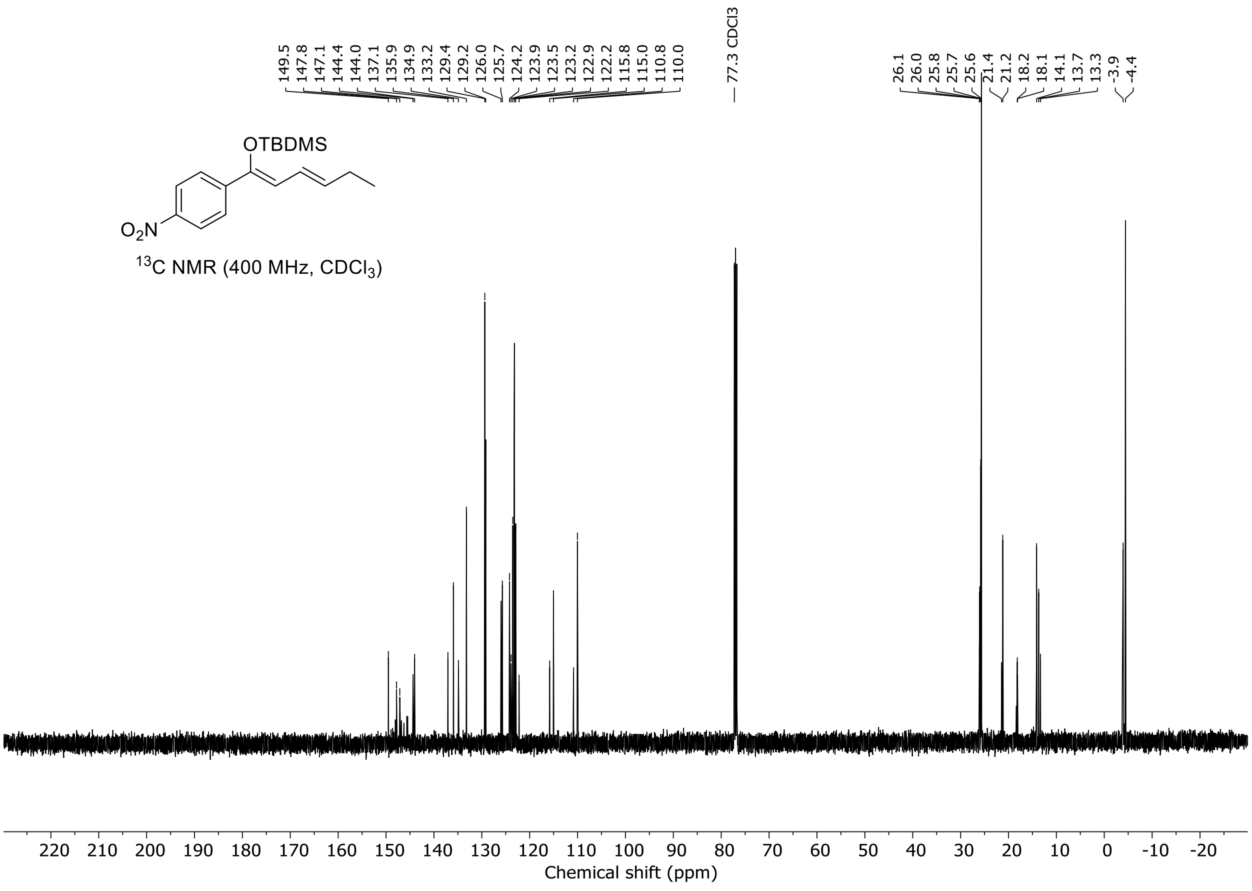

1-(4-(1-((*tert*-butyldimethylsilyl)oxy)hexa-1,3-dien-1-yl)phenyl)ethan-1-one (S10, crude)

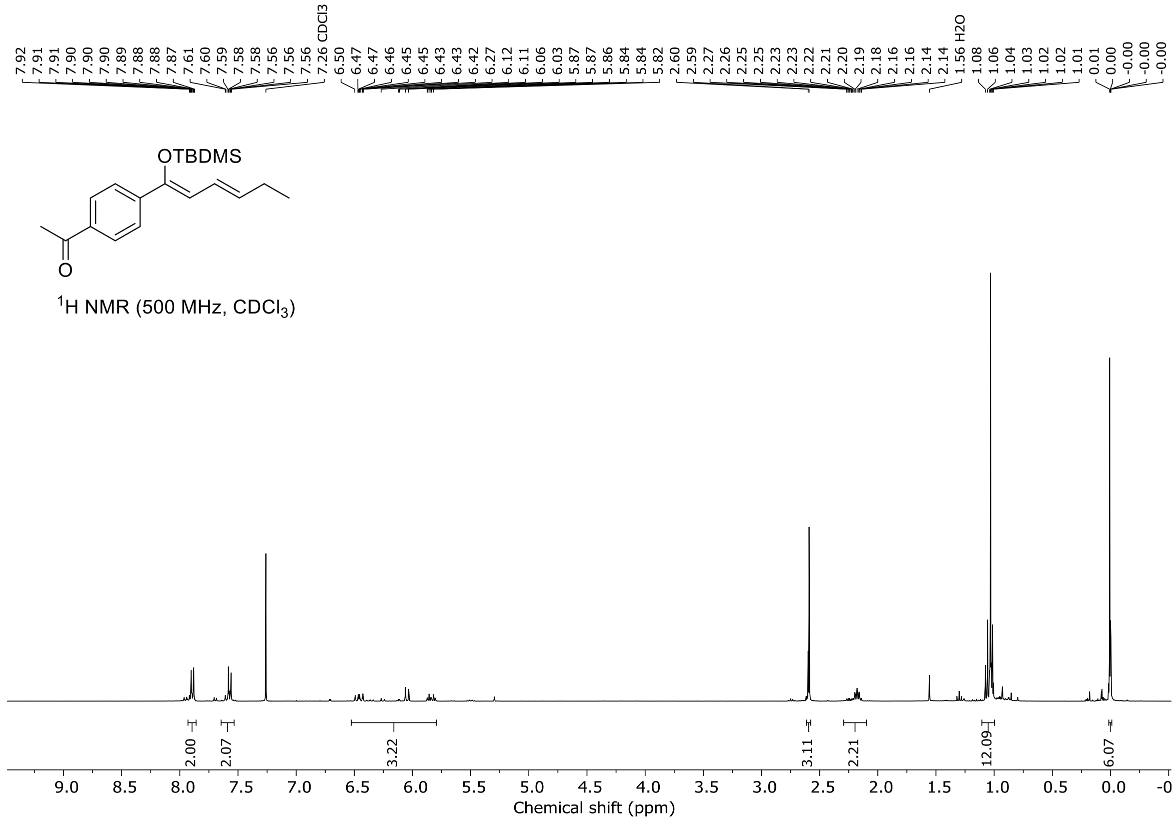

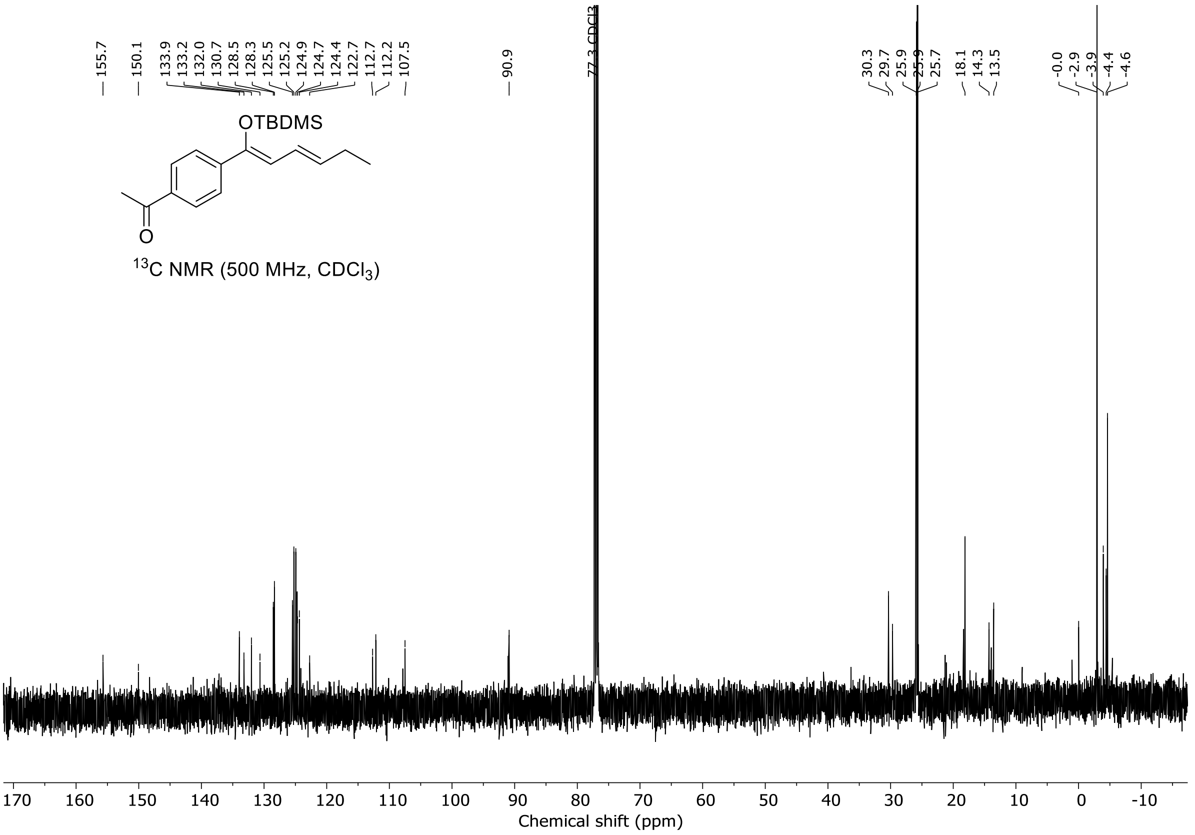

4-(1-((*tert*-butyldimethylsilyl)oxy)hexa-1,3-dien-1-yl)benzonitrile (S11, crude)

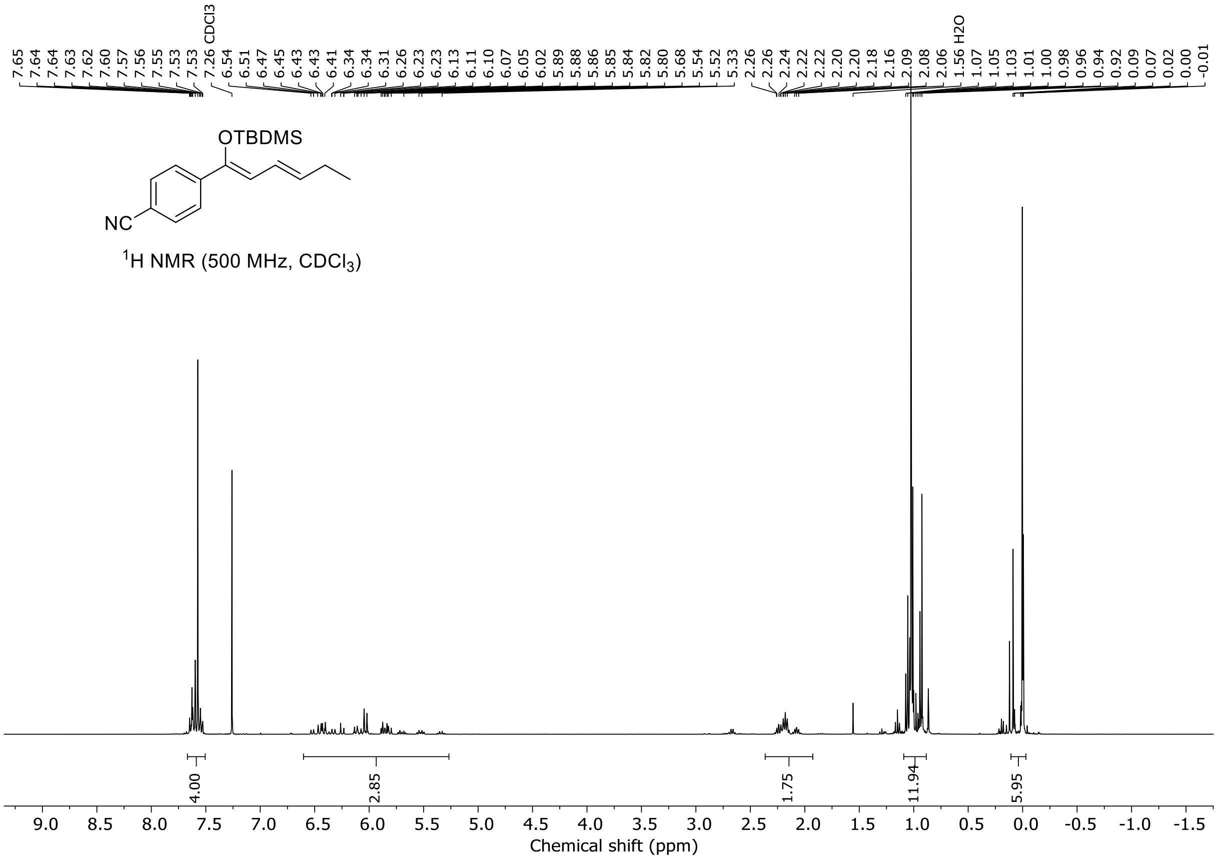

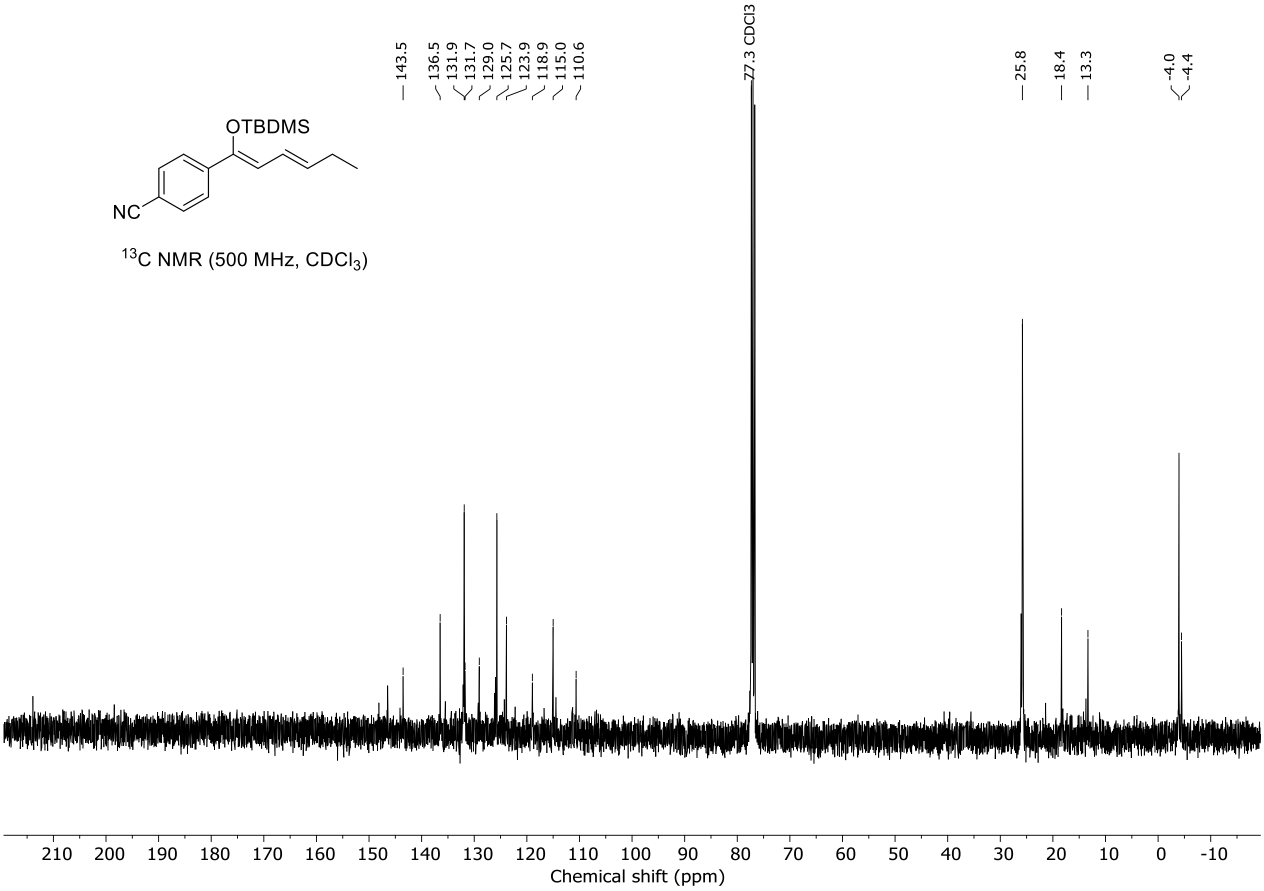

***tert*-butyl(((1-(4-methoxyphenyl)-4-(4-(trifluoromethyl)phenyl)buta-1,3-dien-1-yl)oxy)dimethylsilane (S12, crude)**

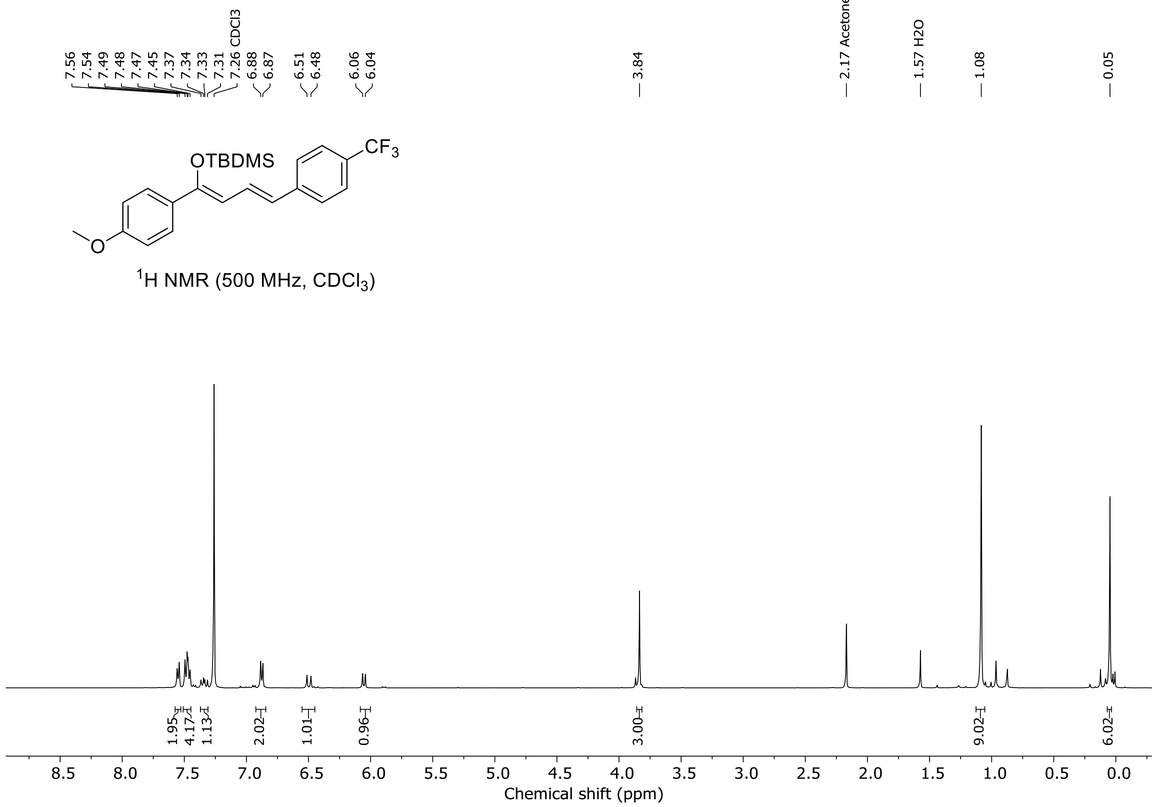

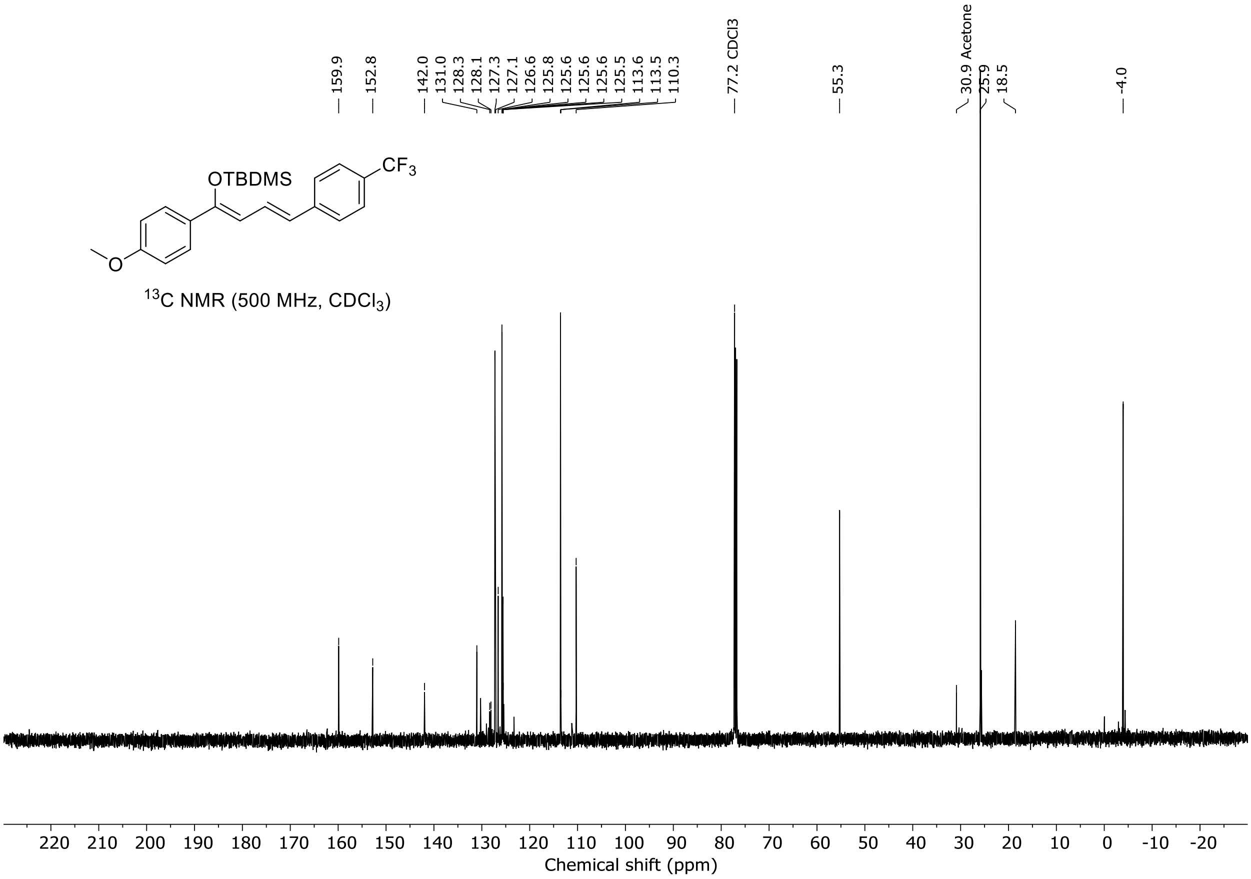

((1,4-bis(4-(trifluoromethyl)phenyl)buta-1,3-dien-1-yl)oxy)(tert-butyl)dimethylsilane (S13, crude)

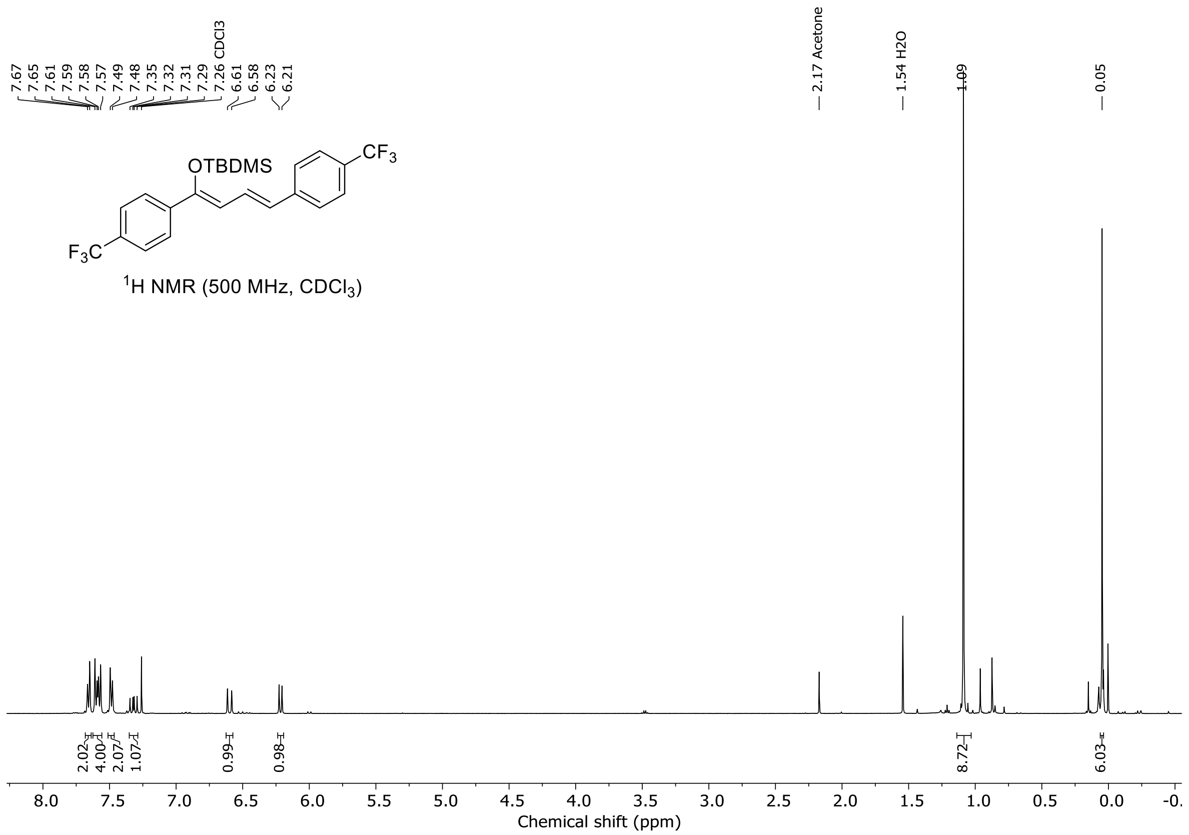

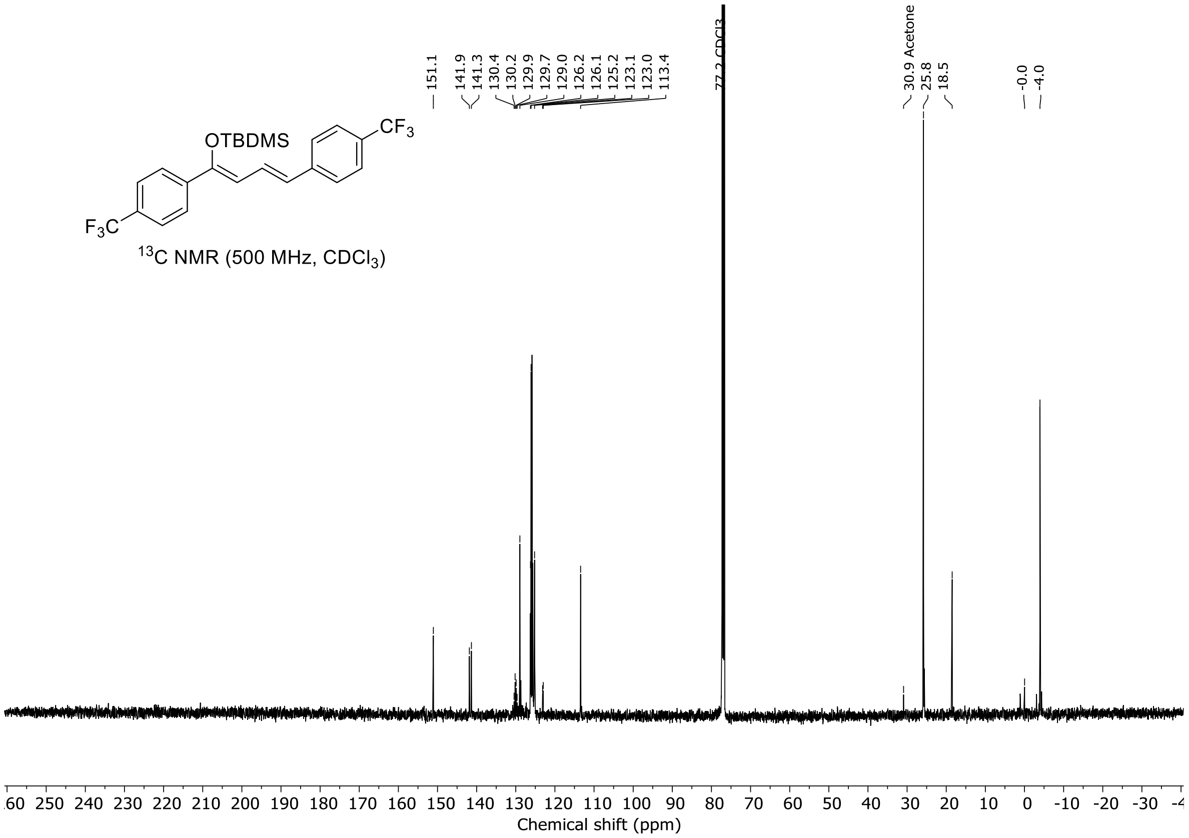

***tert*-butyl((4-(4-methoxyphenyl)-1-(4-(trifluoromethyl)phenyl)buta-1,3-dien-1-yl)oxy)dimethylsilane (*Z*/*E* mixture) (S14, crude)**

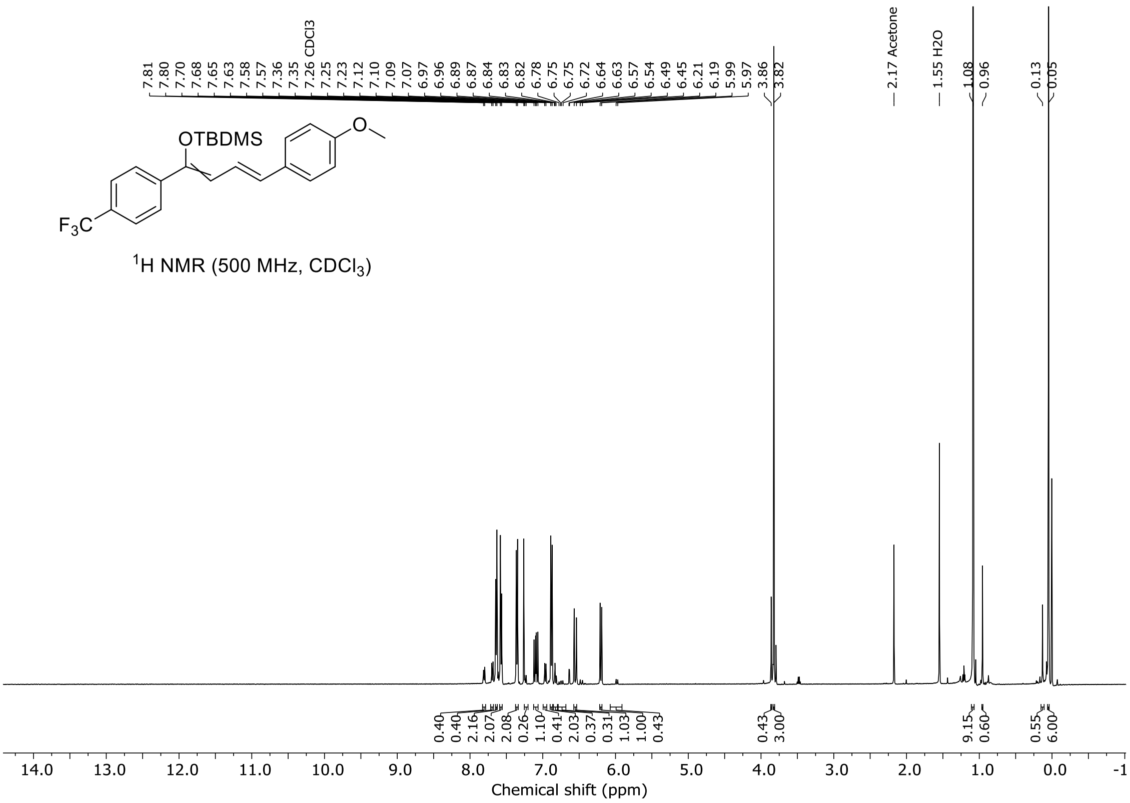

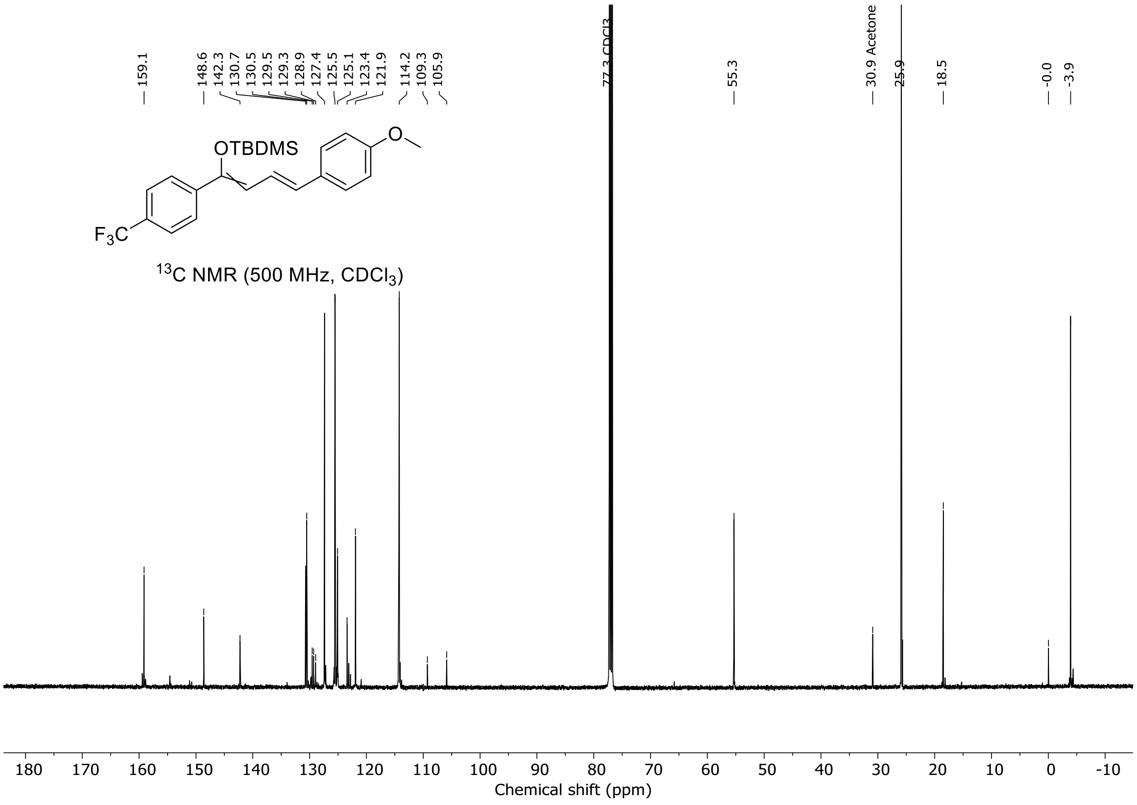

(1*Z*,3*E*)-1,4-bis(3-methoxyphenyl)buta-1,3-dien-1-yl acetate (S16)

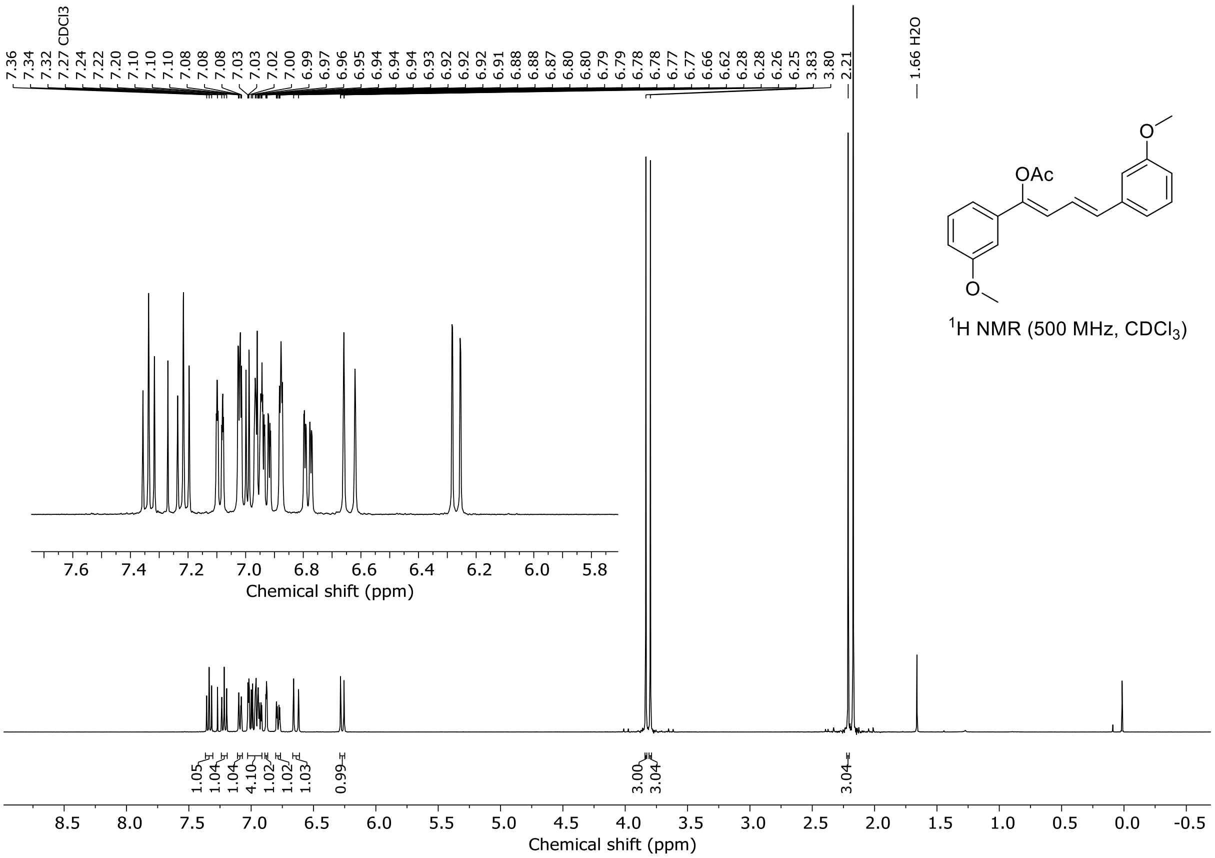

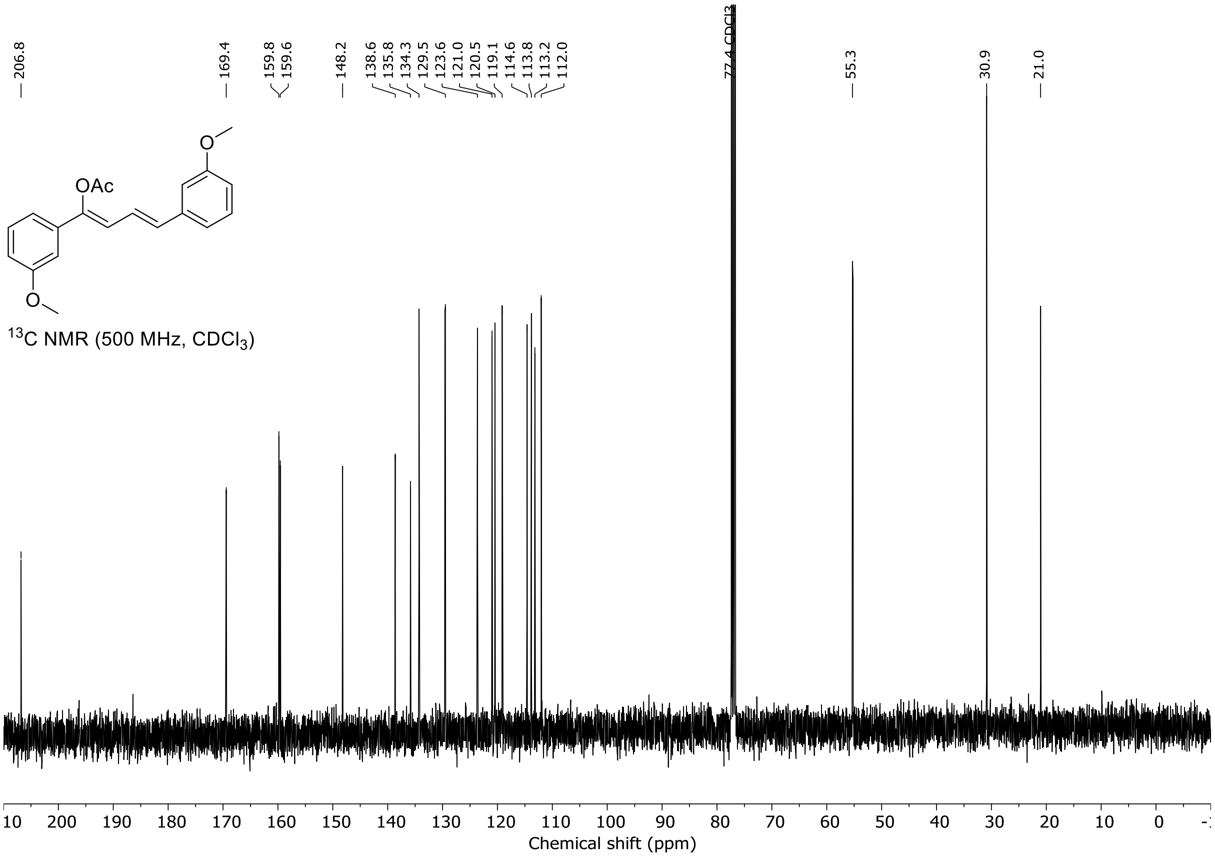

1,4-bis(2-methoxyphenyl)buta-1,3-dien-1-yl acetate (S17)

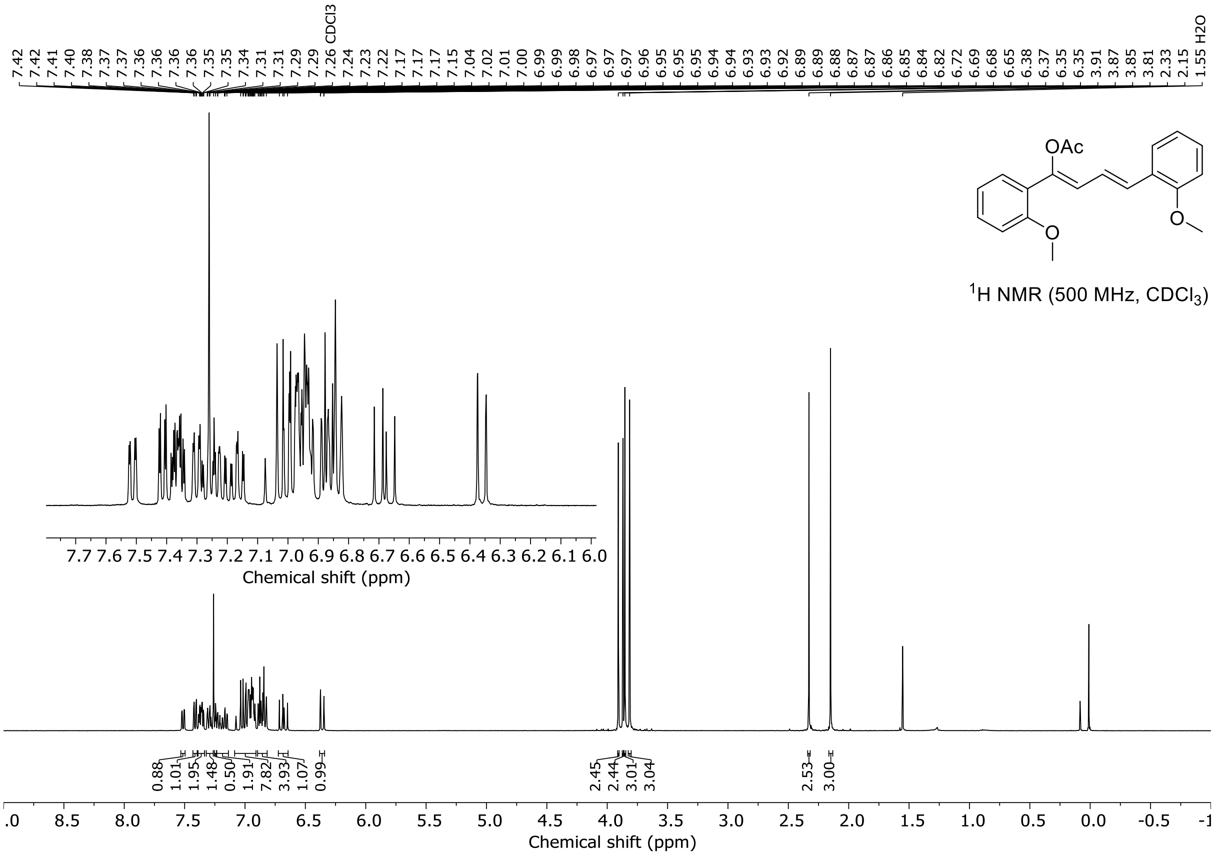

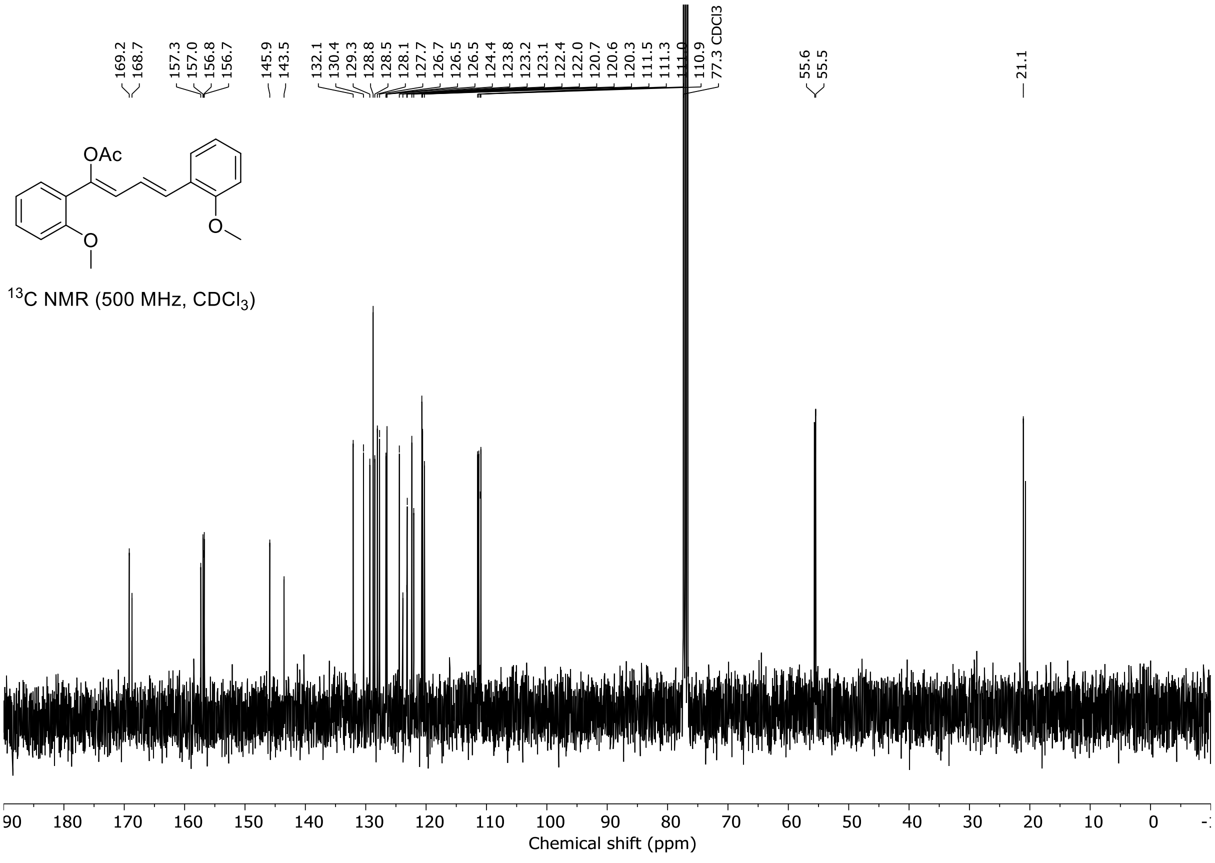

tert-butyl((2,2-dimethylocta-3,5-dien-3-yl)oxy)dimethylsilane (S25, crude)

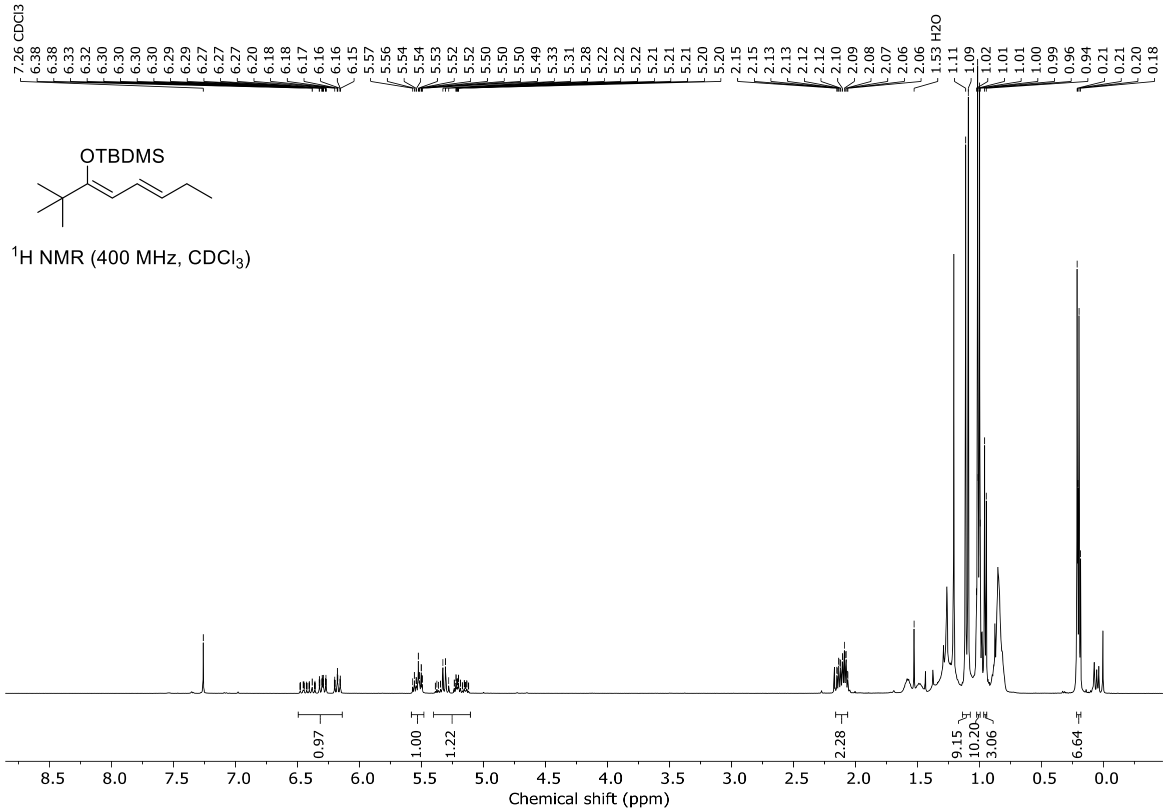

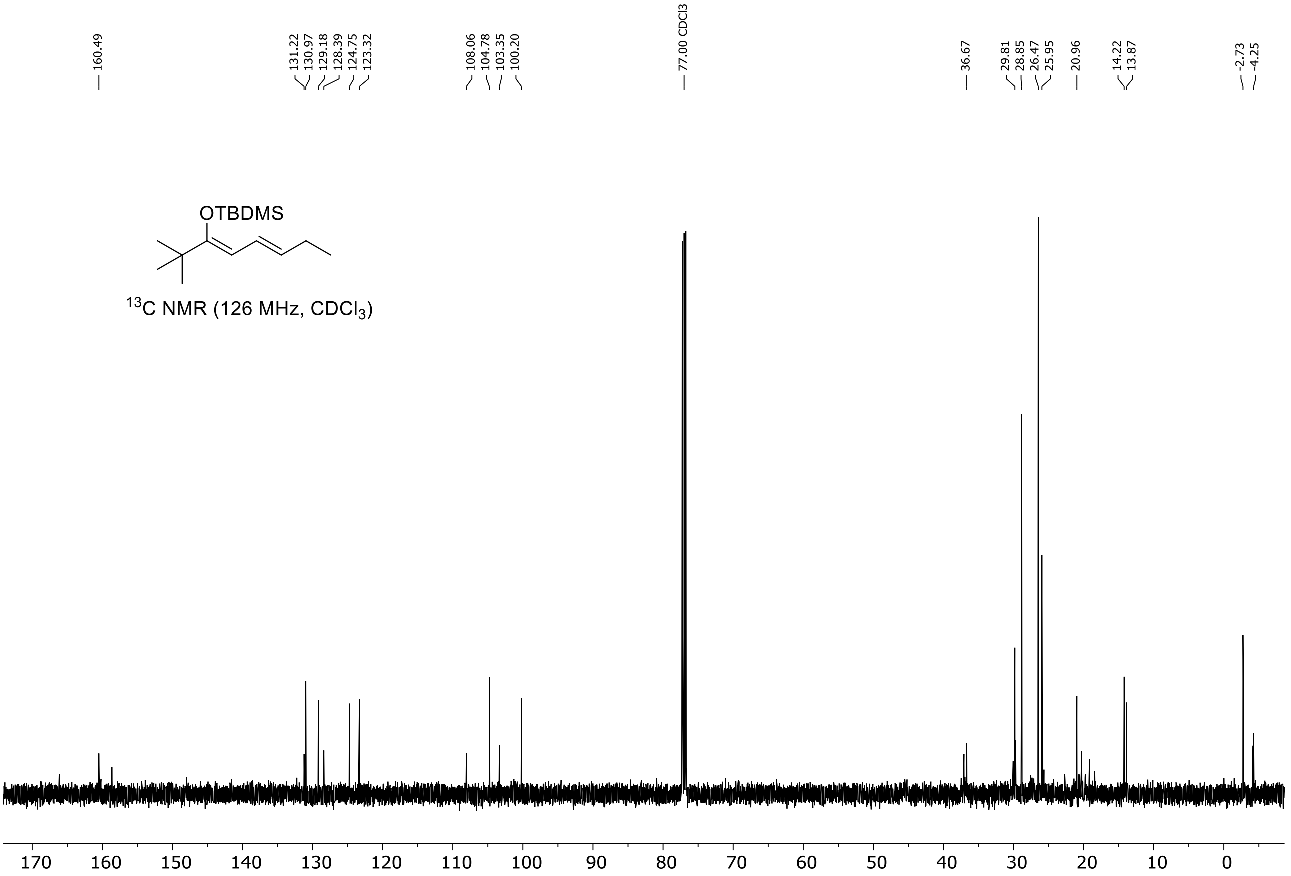

tert-butyl(((4_R_,4a_S_,6_R_)-4,4a-dimethyl-6-(prop-1-en-2-yl)-3,4,4a,5,6,7-hexahydronaphthalen-2-yl)oxy)dimethylsilane (S26, crude)

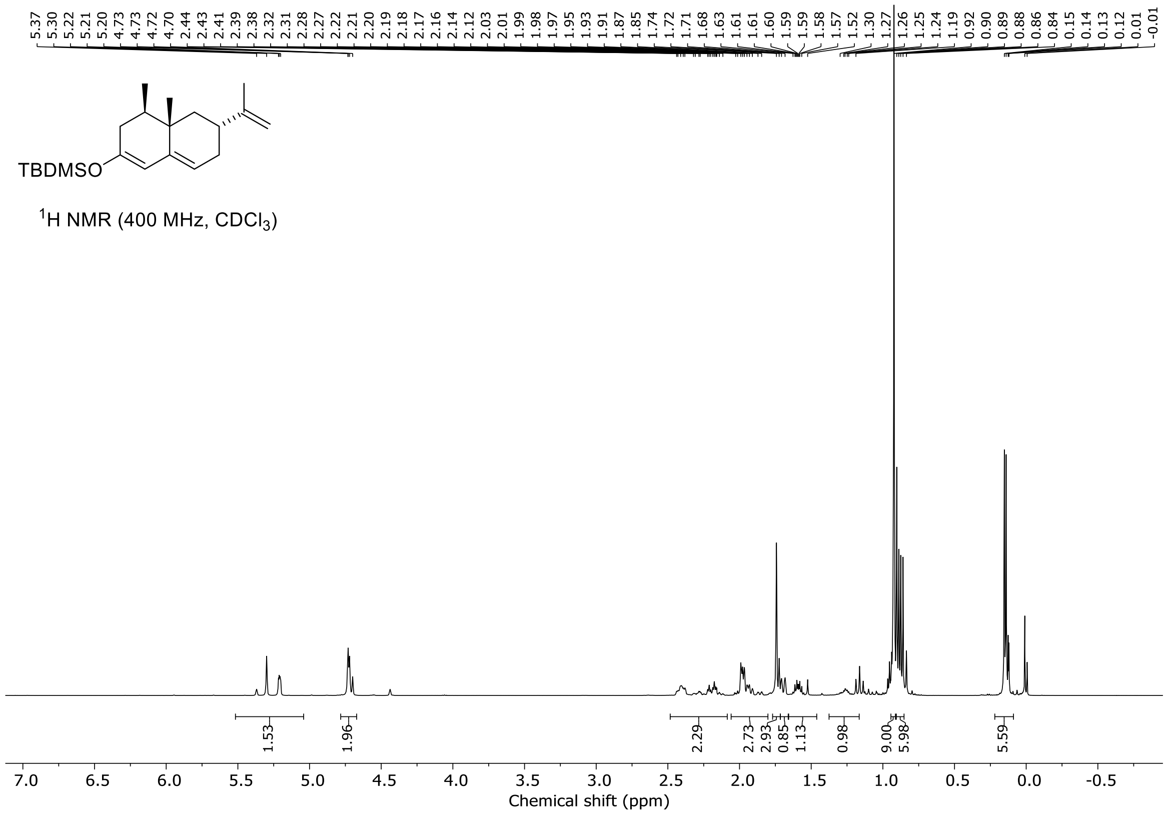

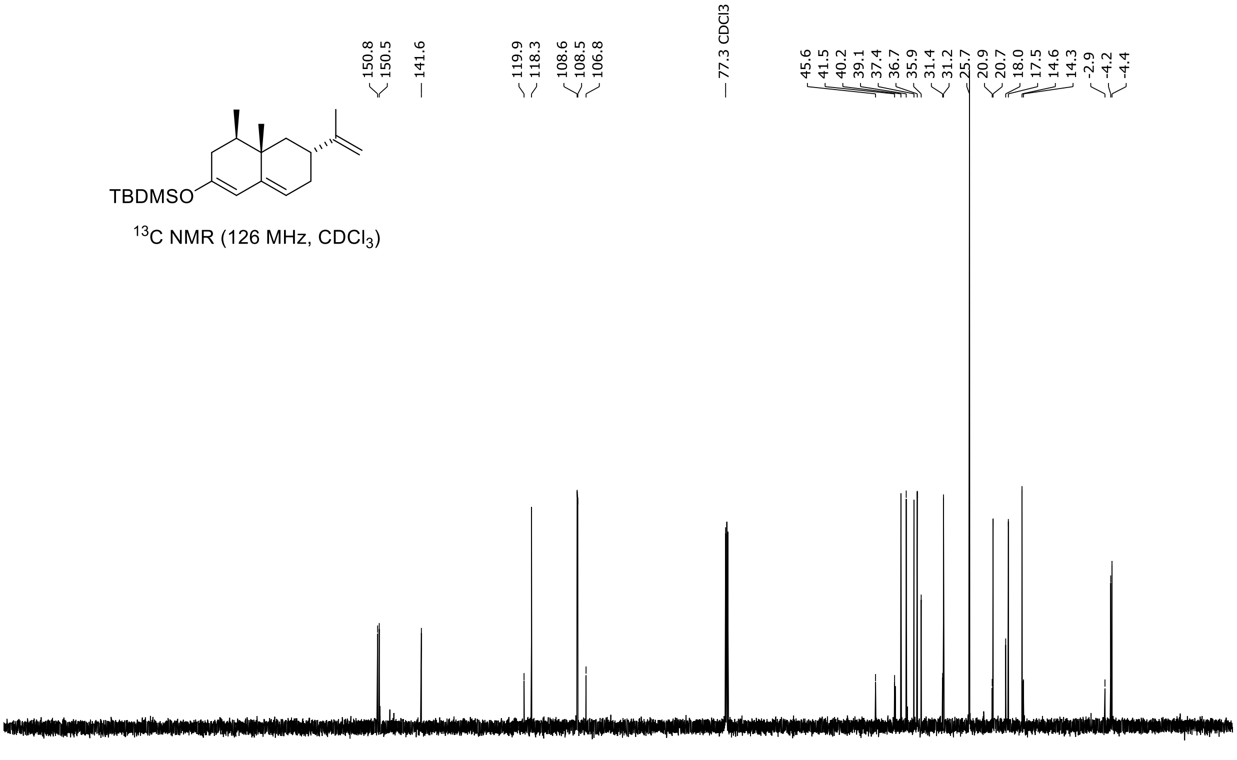

(1*R*,8*R*,9*S*,13*S*,14*S*,17*S*)-3-((*tert*-butyldimethylsilyl)oxy)-1,13-dimethyl-2,7,8,9,10,11,12,13,14,15,16,17-dodecahydro-1H-cyclopenta[a]phenanthren-17-yl acetate (S27, crude)

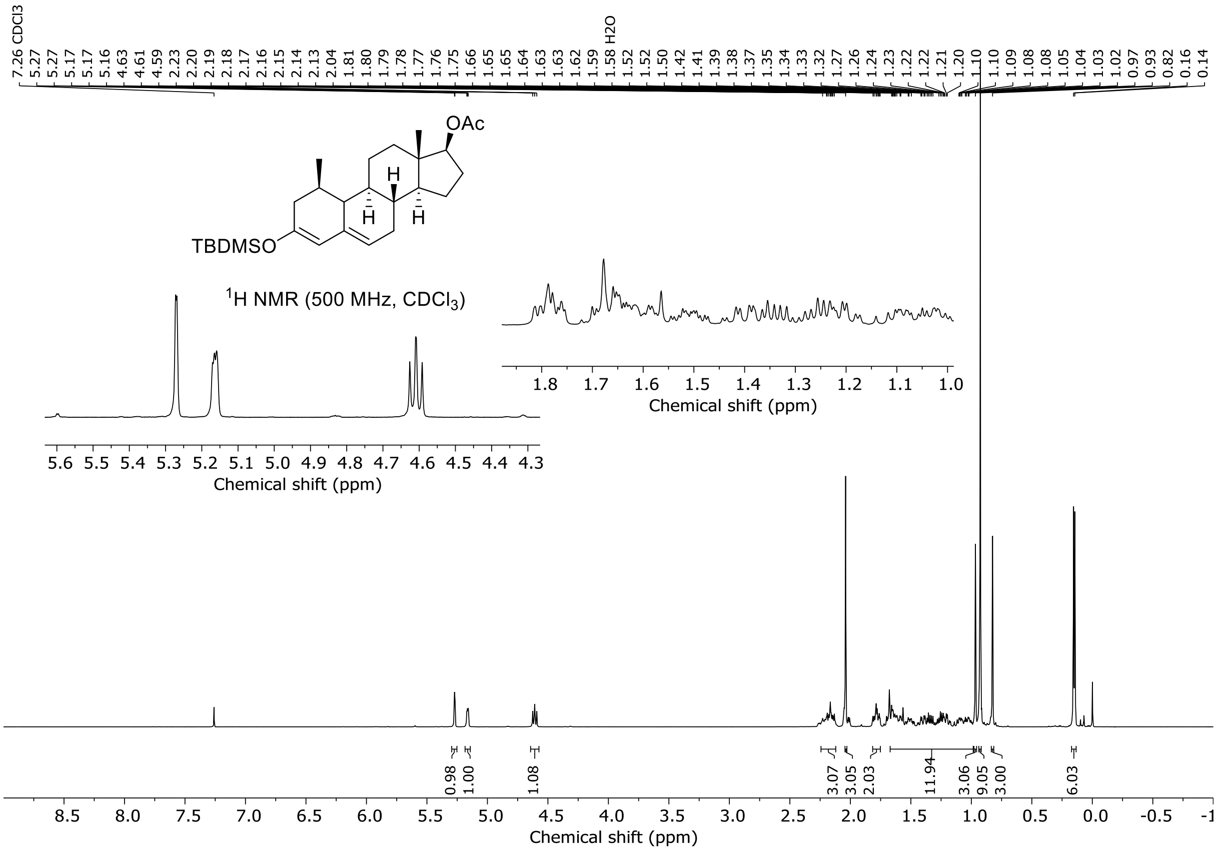

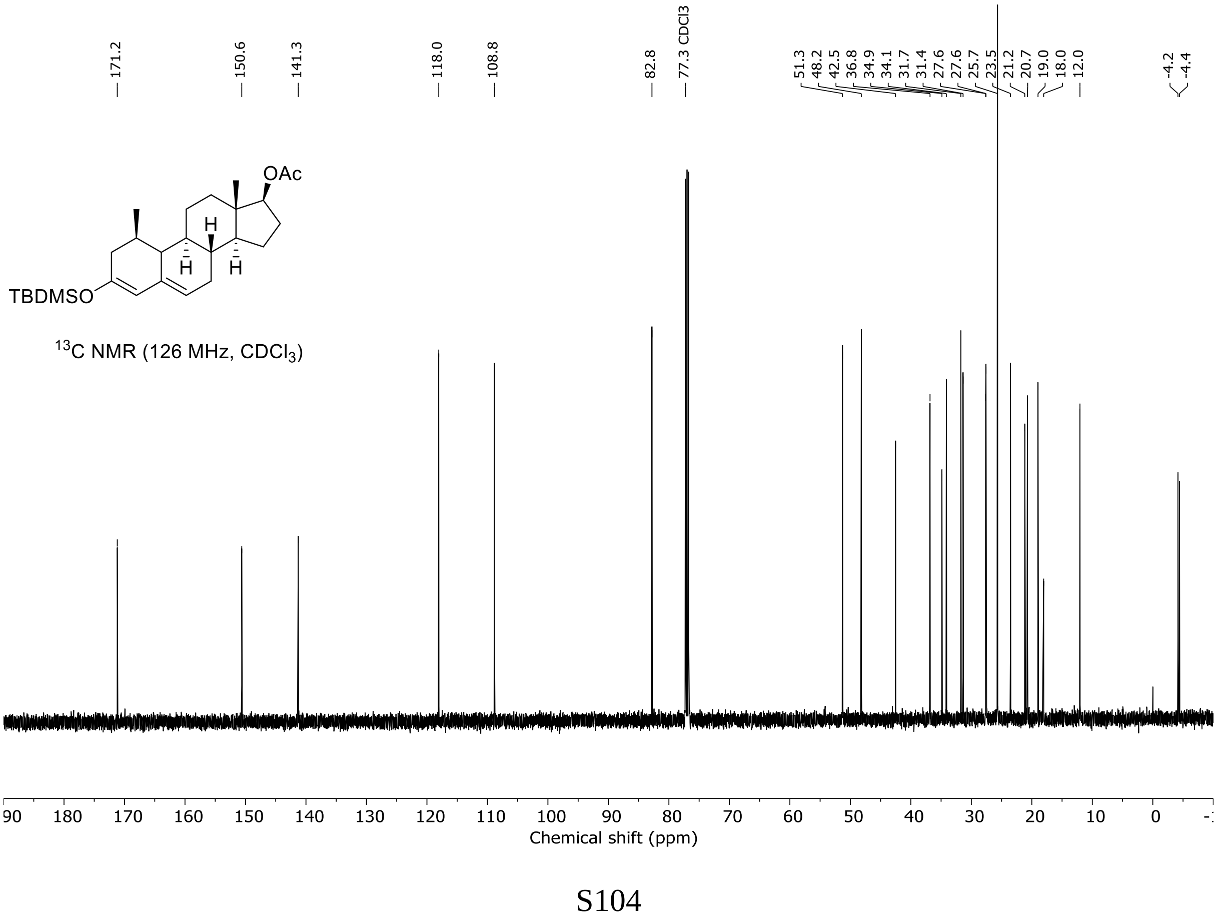

tert-butyl((3_E_)-hexa-1,3-dien-1-yloxy)dimethylsilane (S28, crude)

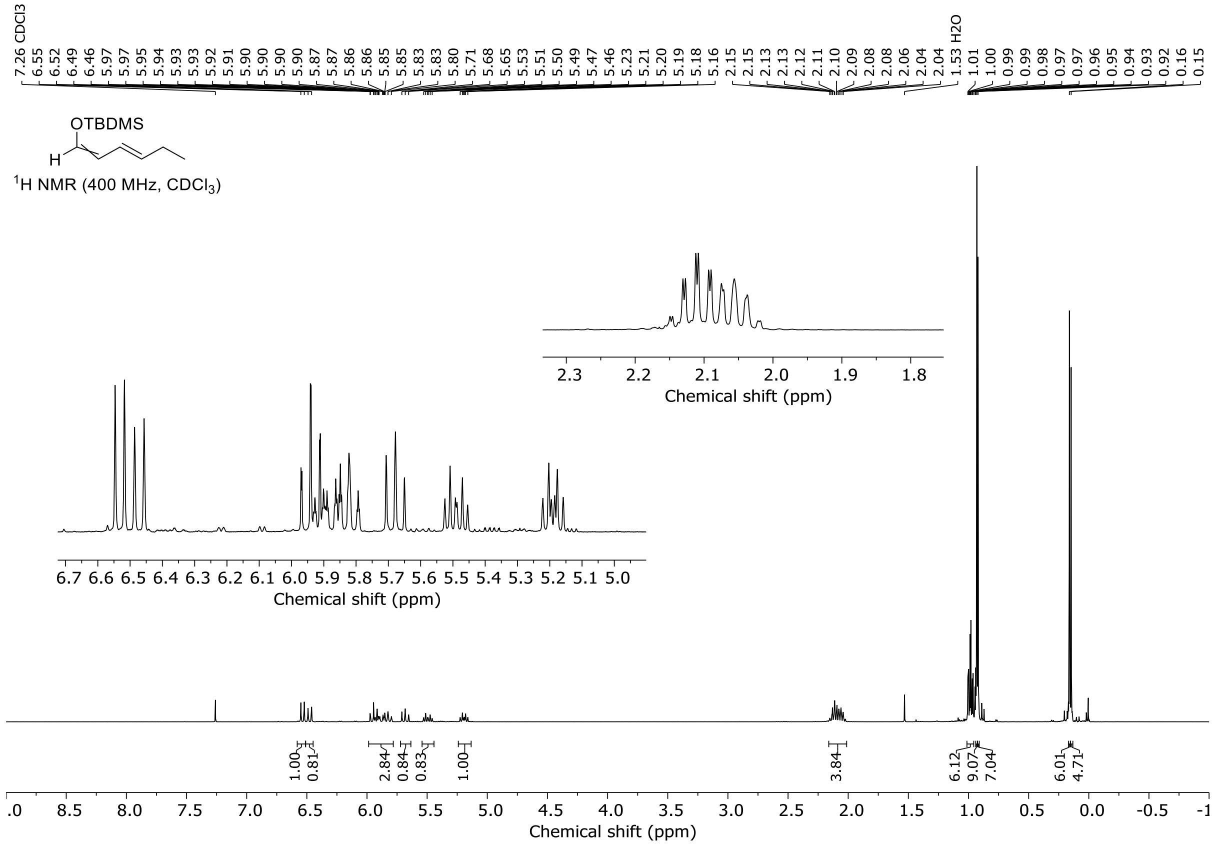

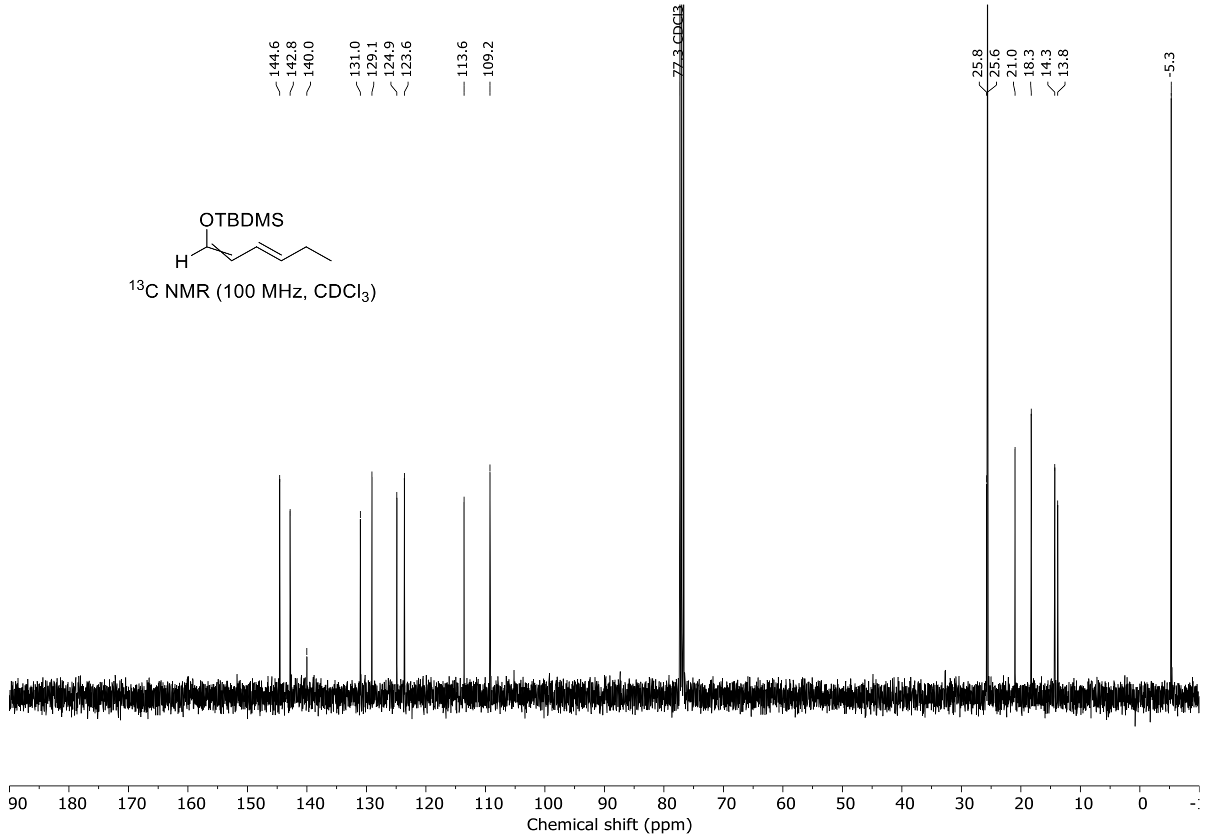

tert-butyl((3,7-dimethylocta-1,3,6-trien-1-yl)oxy)dimethylsilane (S29, crude)

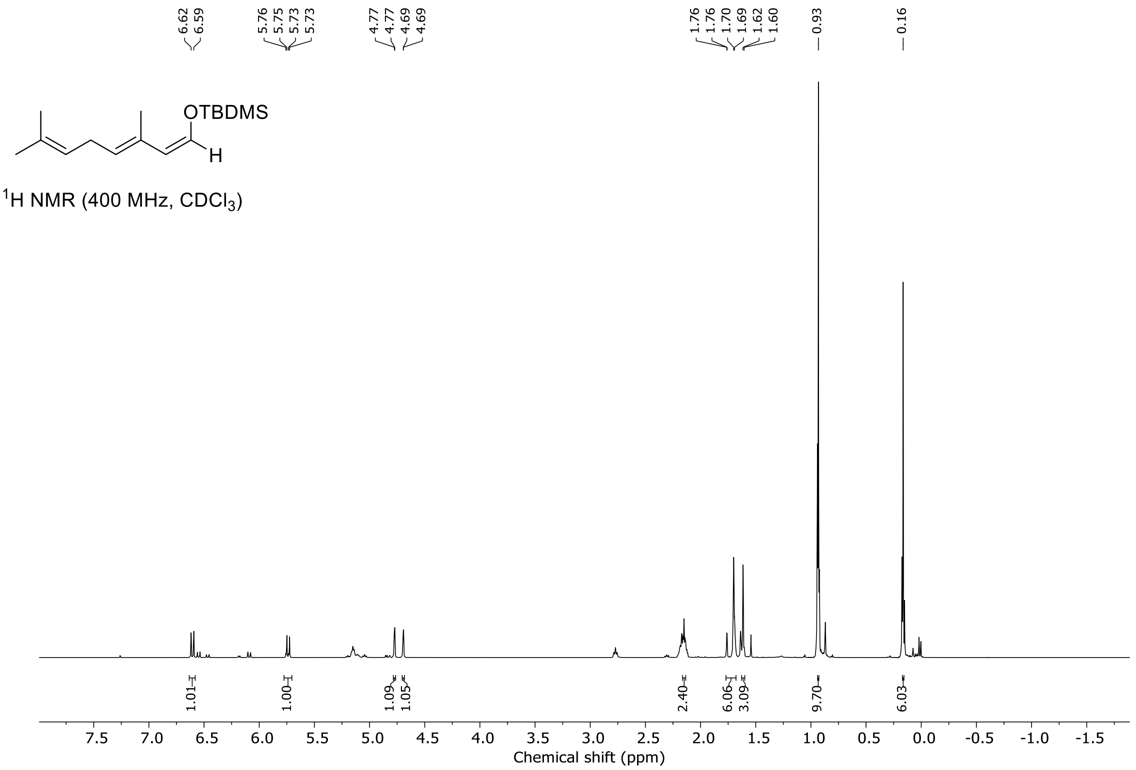

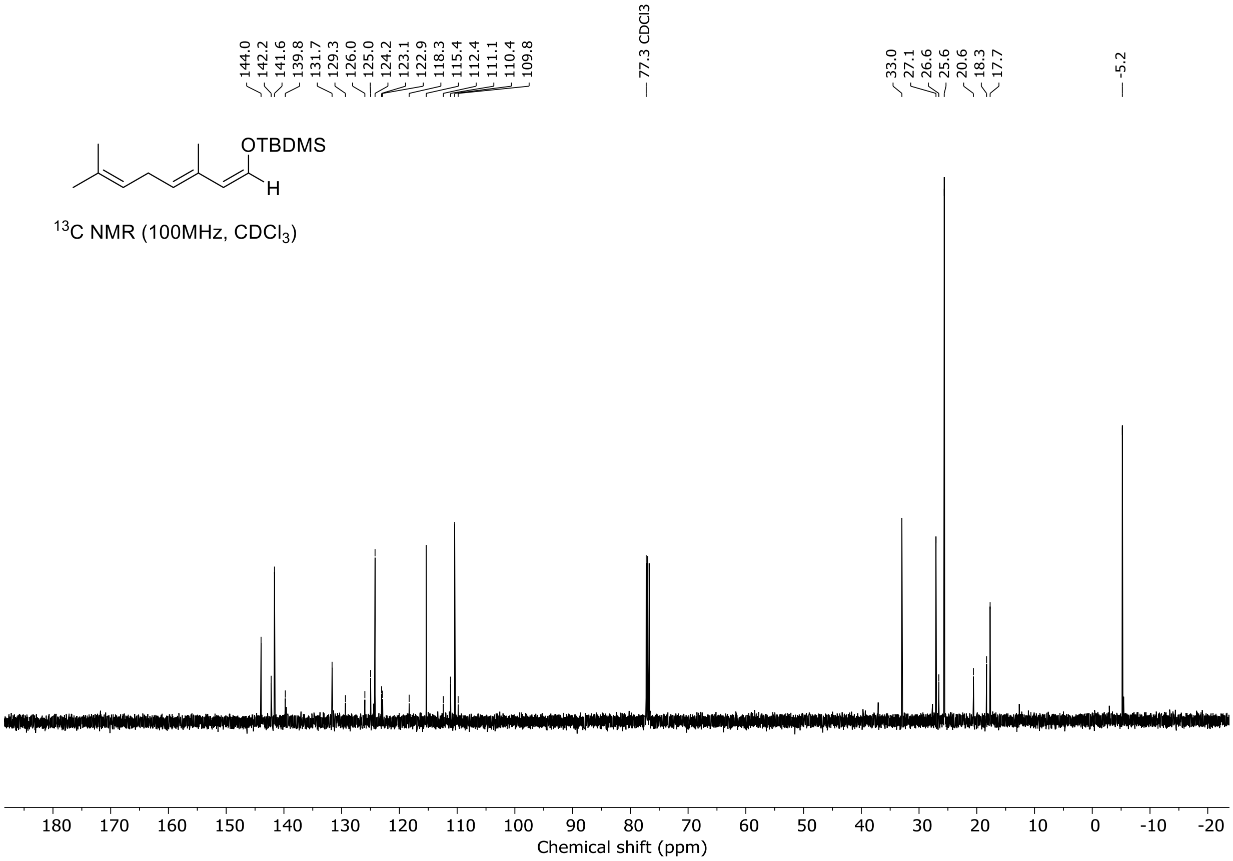

***tert*-butyldimethyl((2,6,6-trimethylcyclohex-2-en-1-ylidene)methoxy)silane (S30, crude)**

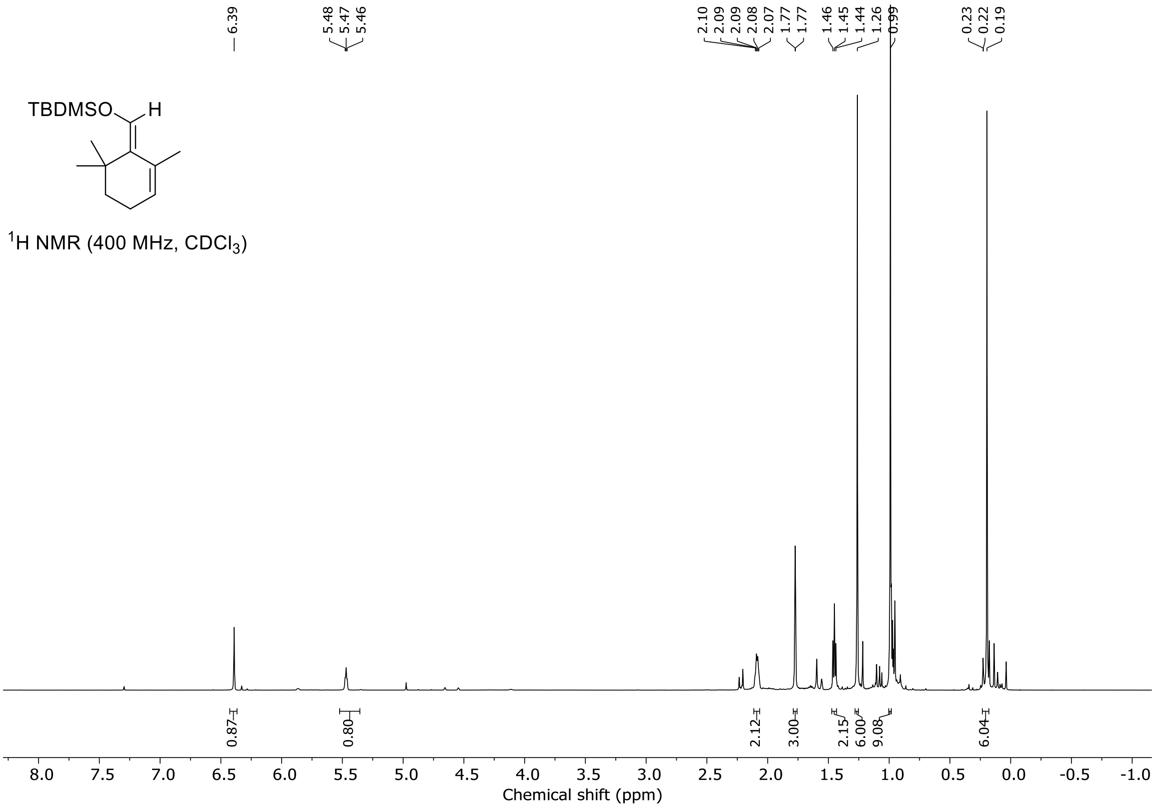

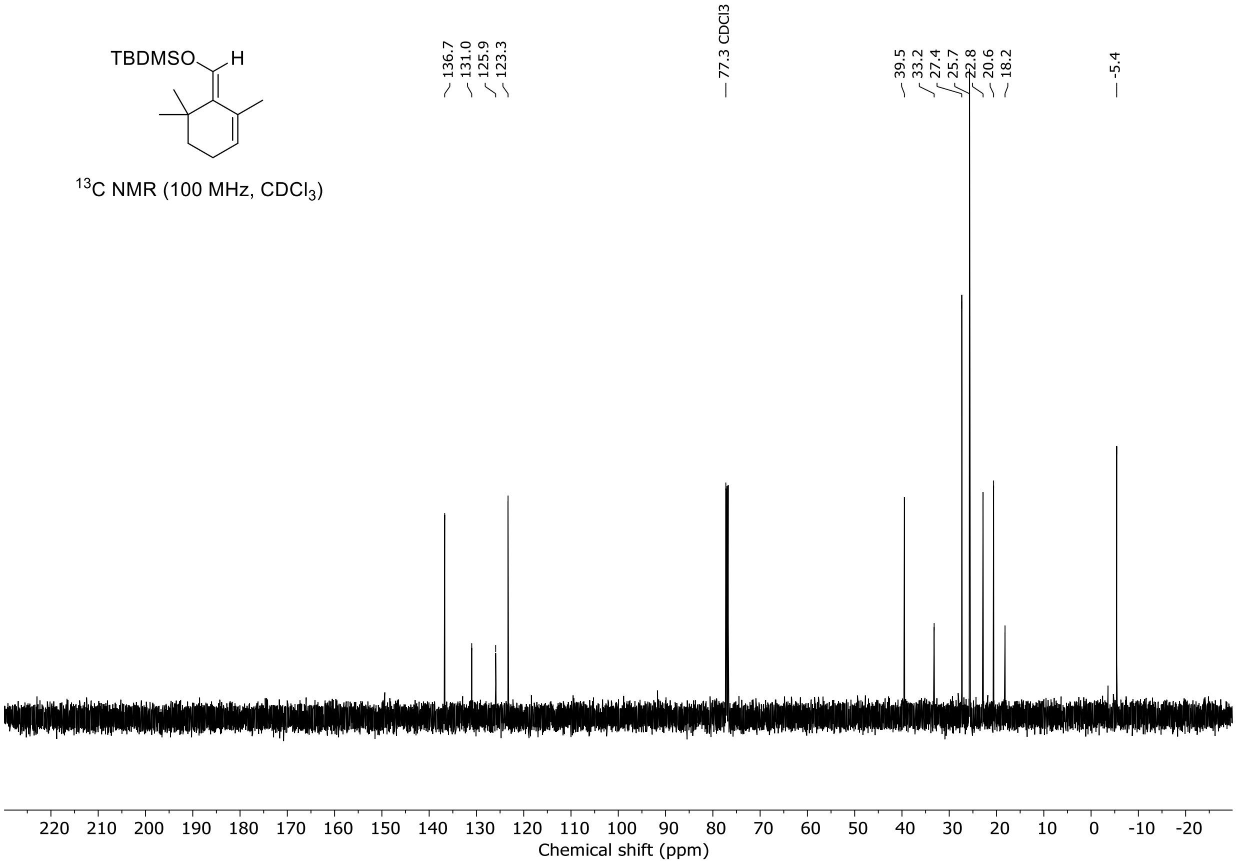

tert-butyldimethyl(2-phenylbuta-1,3-dien-1yl)oxy)silane (S31, crude)

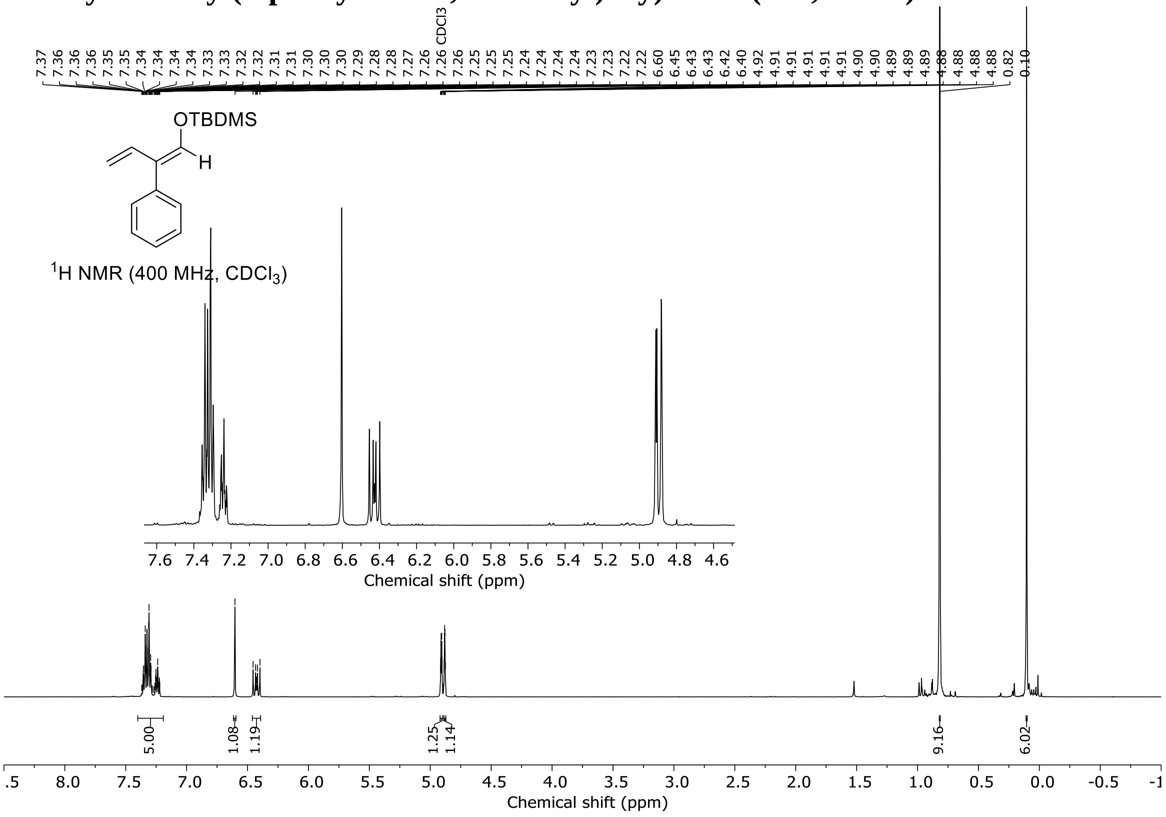

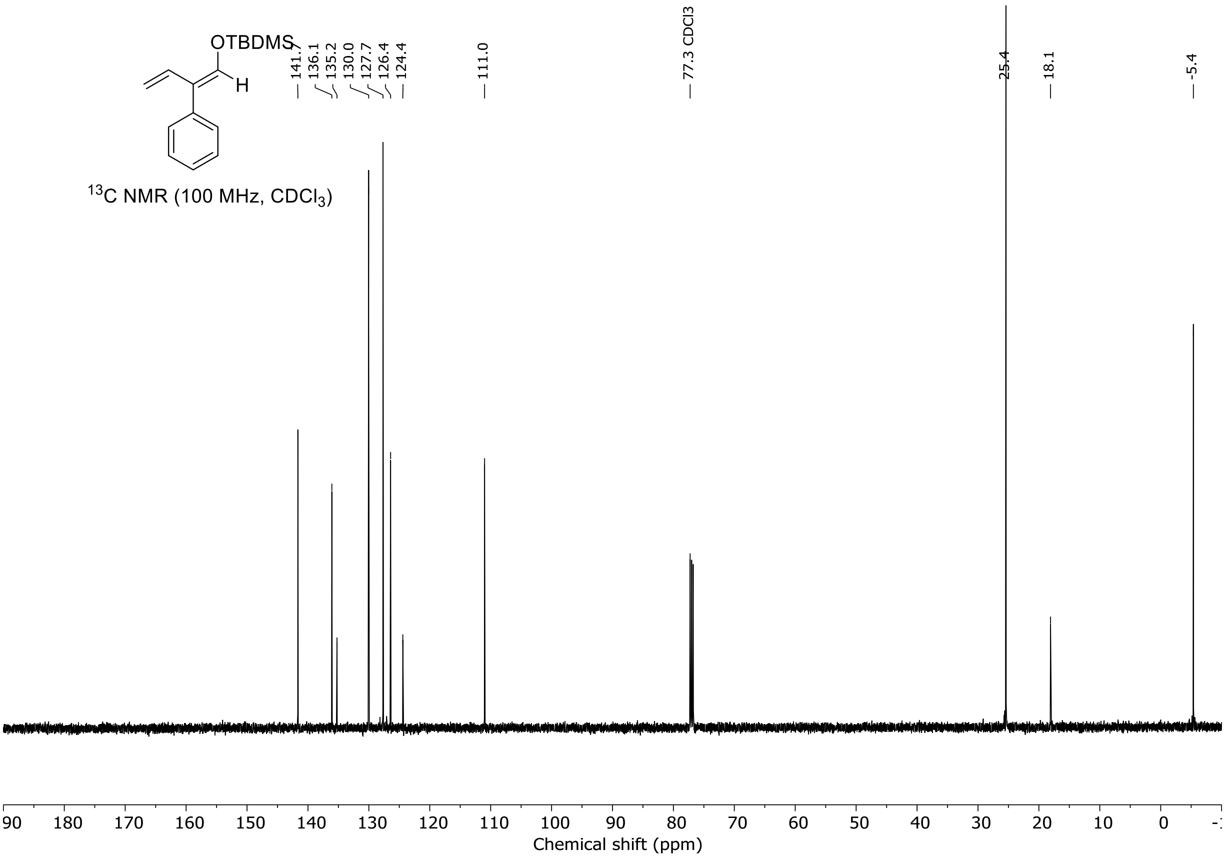

(*E*)-*N*,4-dimethyl-*N*-(4-oxo-4-phenylbut-2-en-1-yl)benzenesulfonamide (3a)

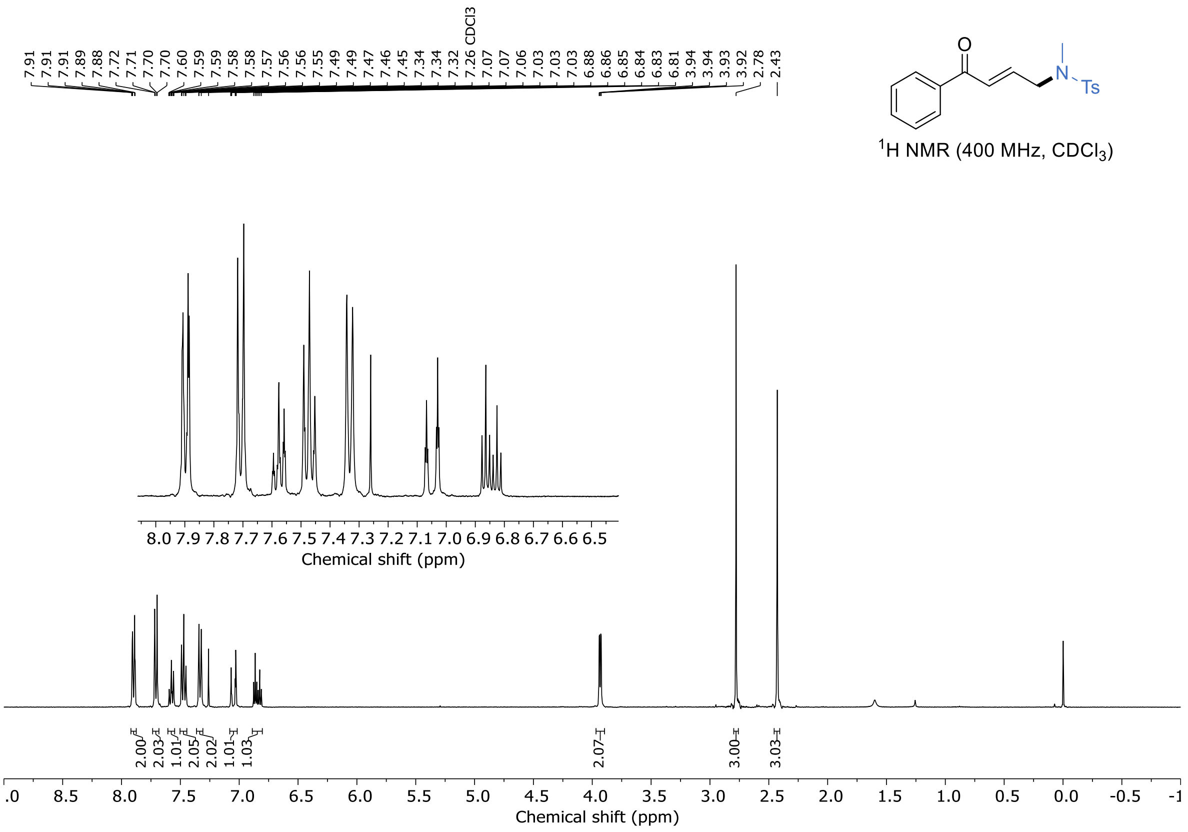

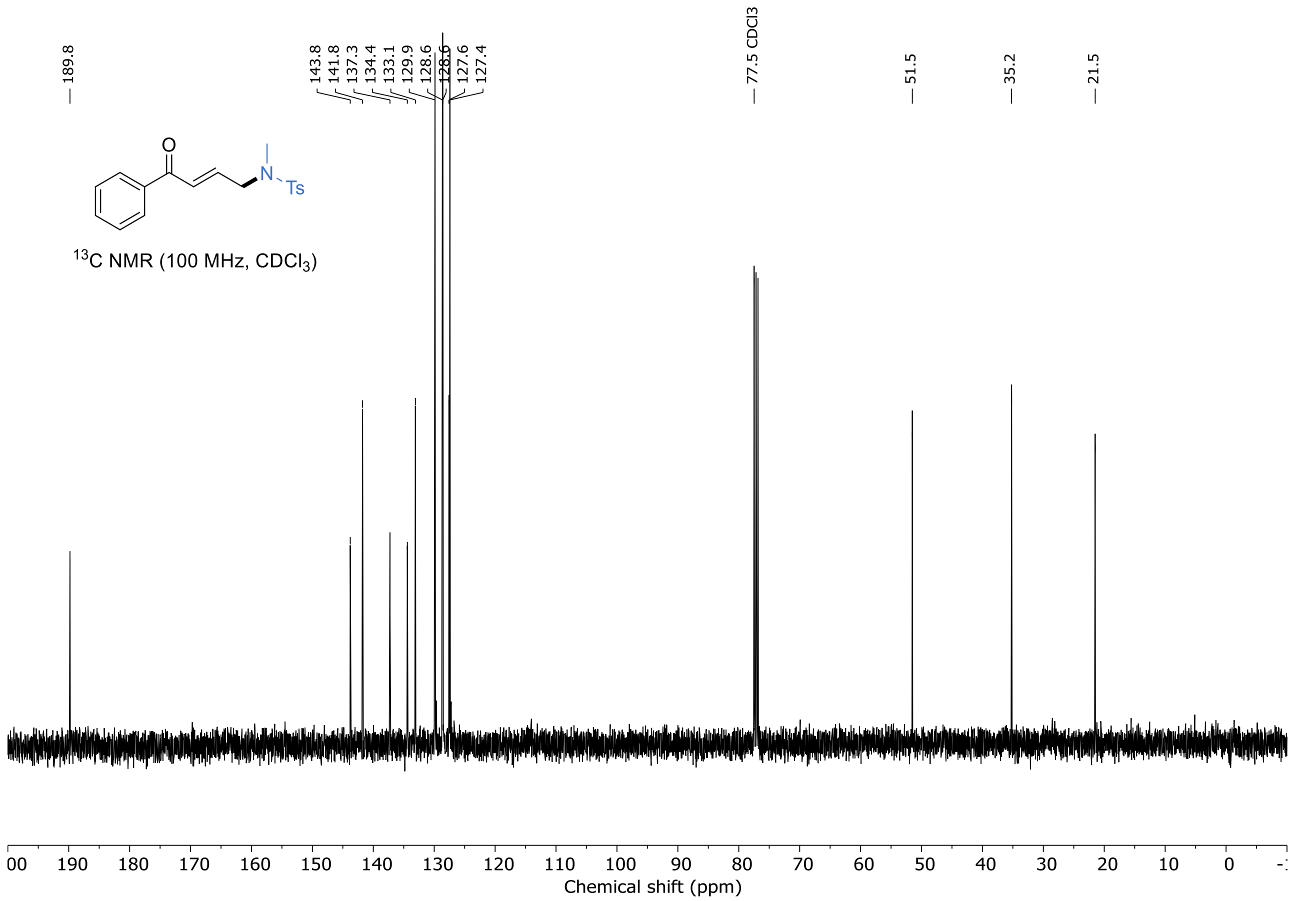

tert-butyl methyl(4-oxo-4-phenylbut-2-en-1-yl)carbamate (_E_/_Z_ mixture) (3b)

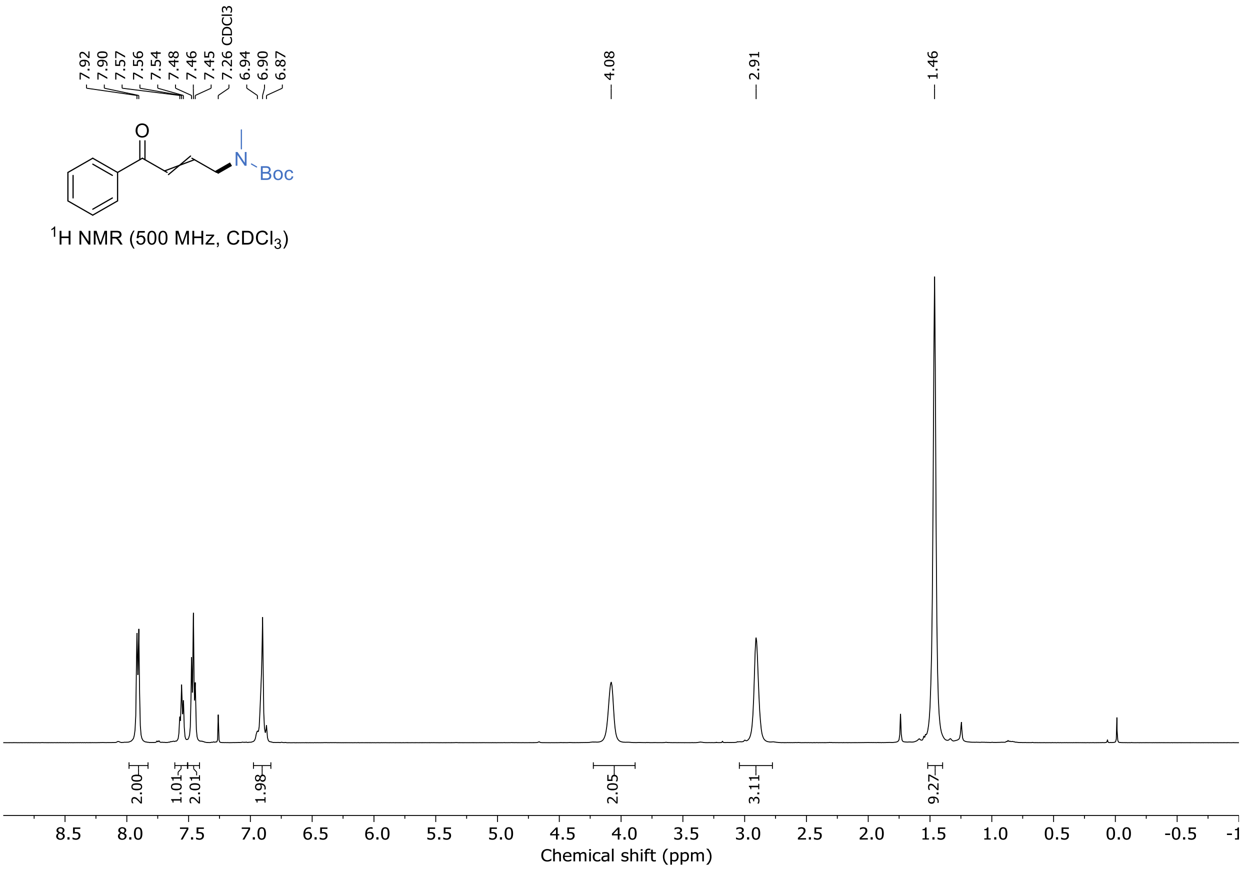

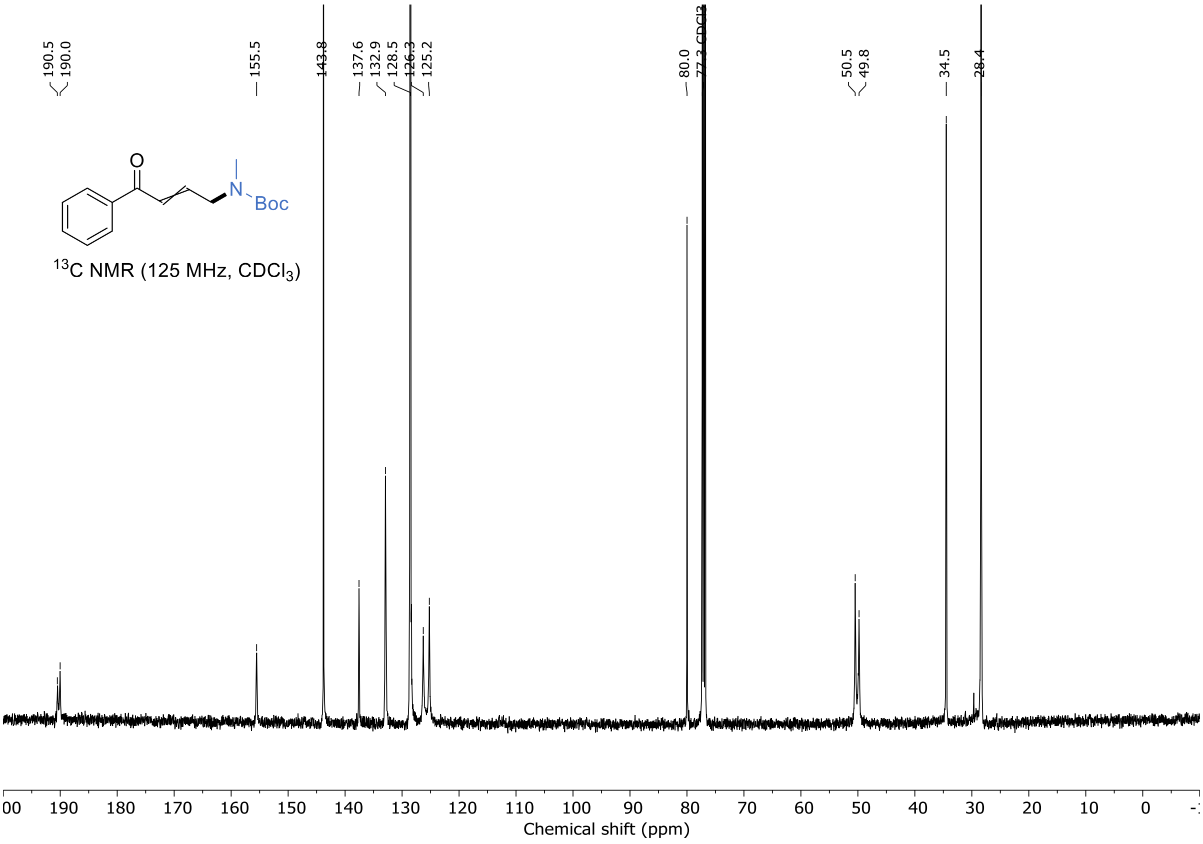

(*E*)-4-methyl-*N*-(4-oxo-4-phenylbut-2-en-1-yl)benzenesulfonamide (3f)

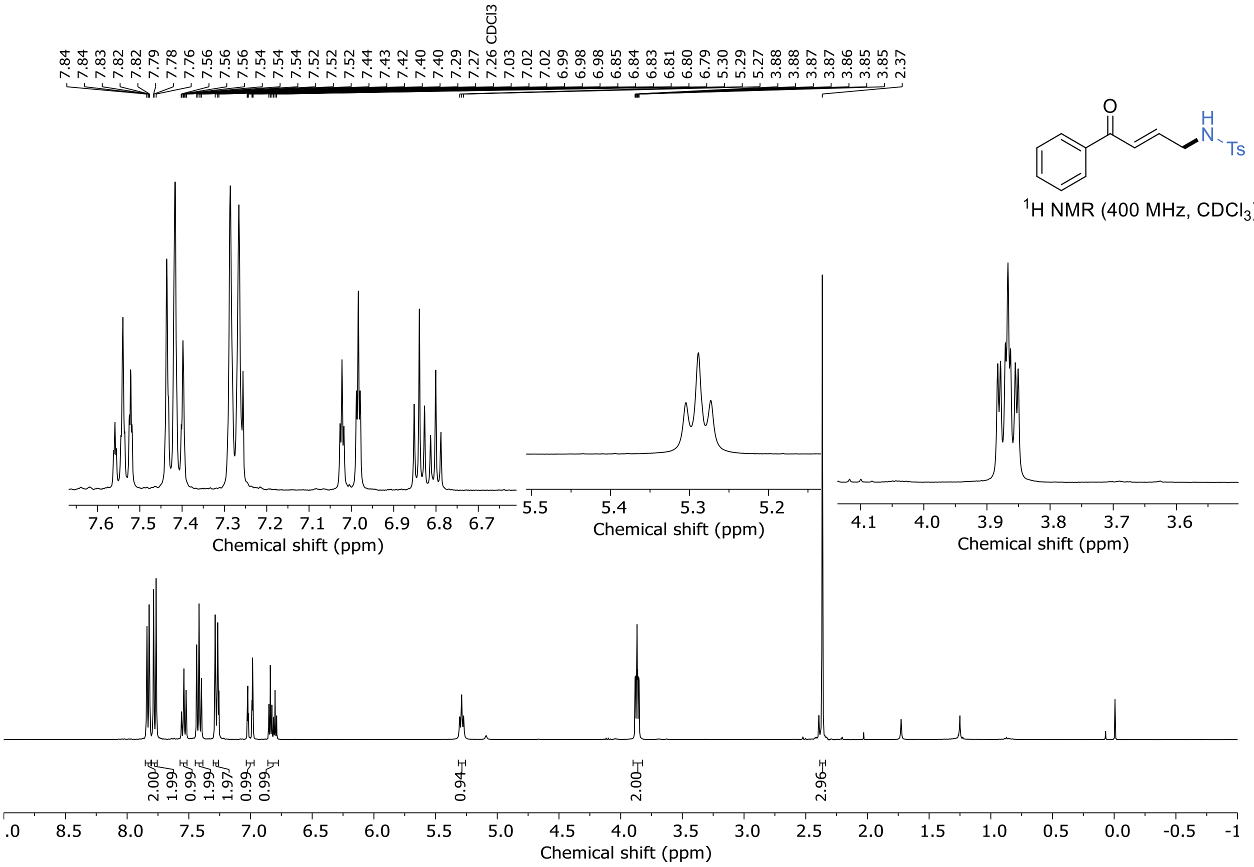

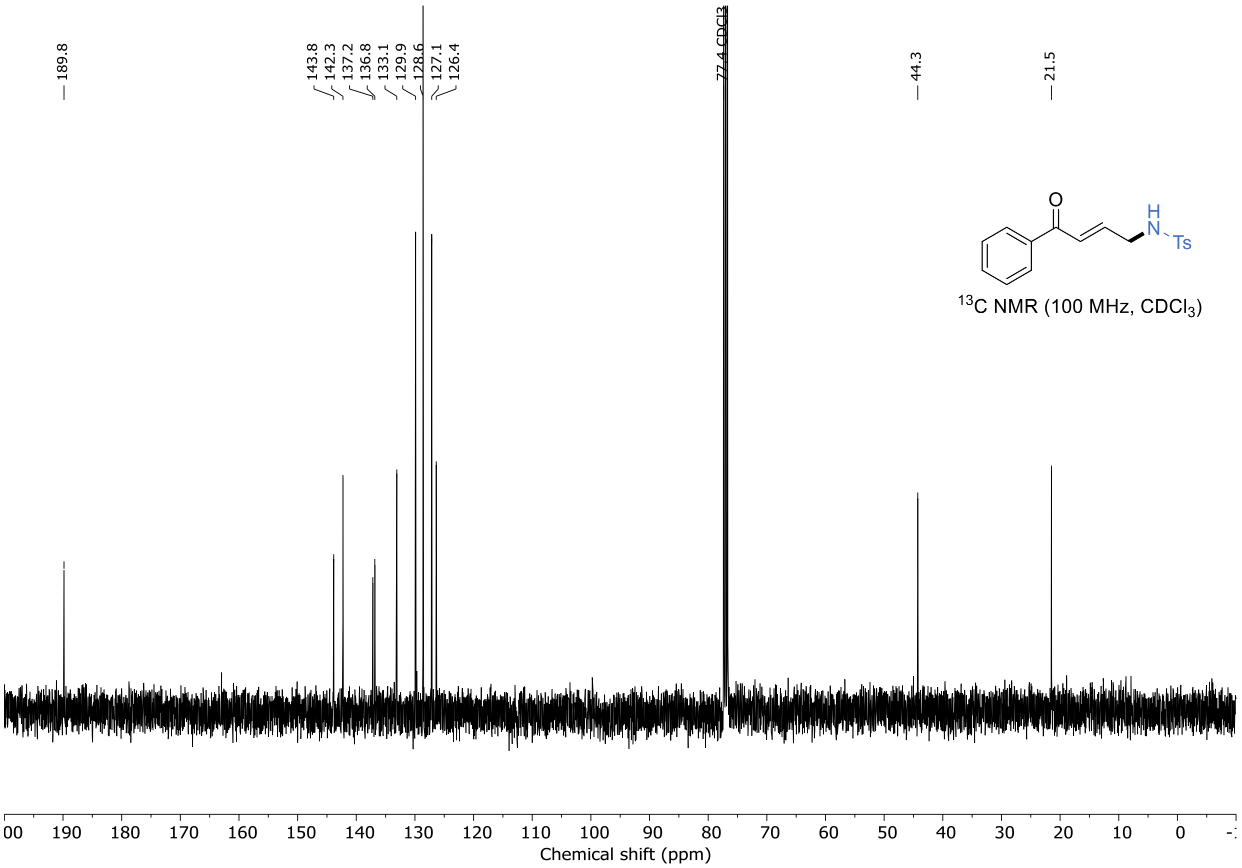

(*E*)-2,3,4,5,6-pentafluoro-*N*-(4-oxo-4-phenylbut-2-en-1-yl)benzamide (3e)

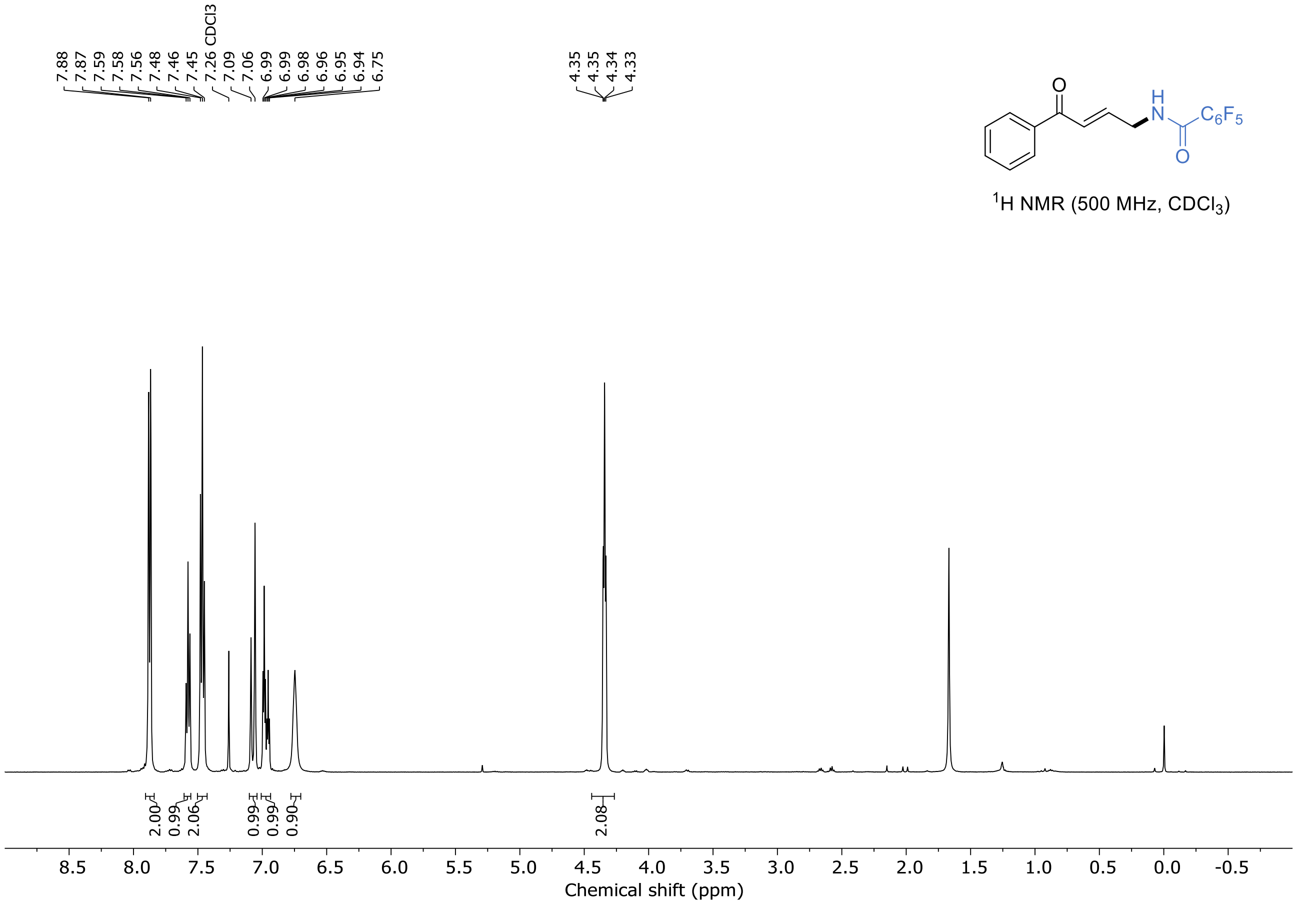

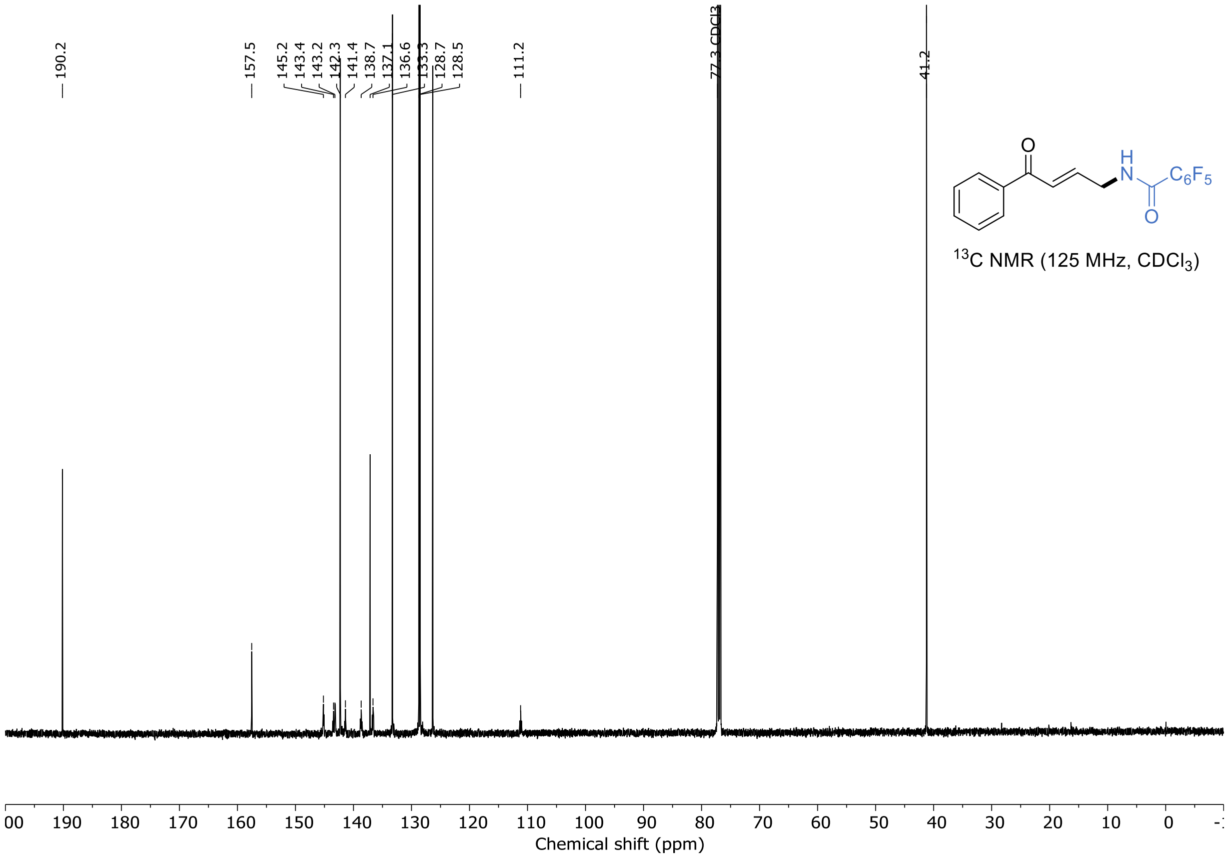

benzyl (*E*)-methyl(4-oxo-4-phenylbut-2-en-1-yl)carbamate (3c)

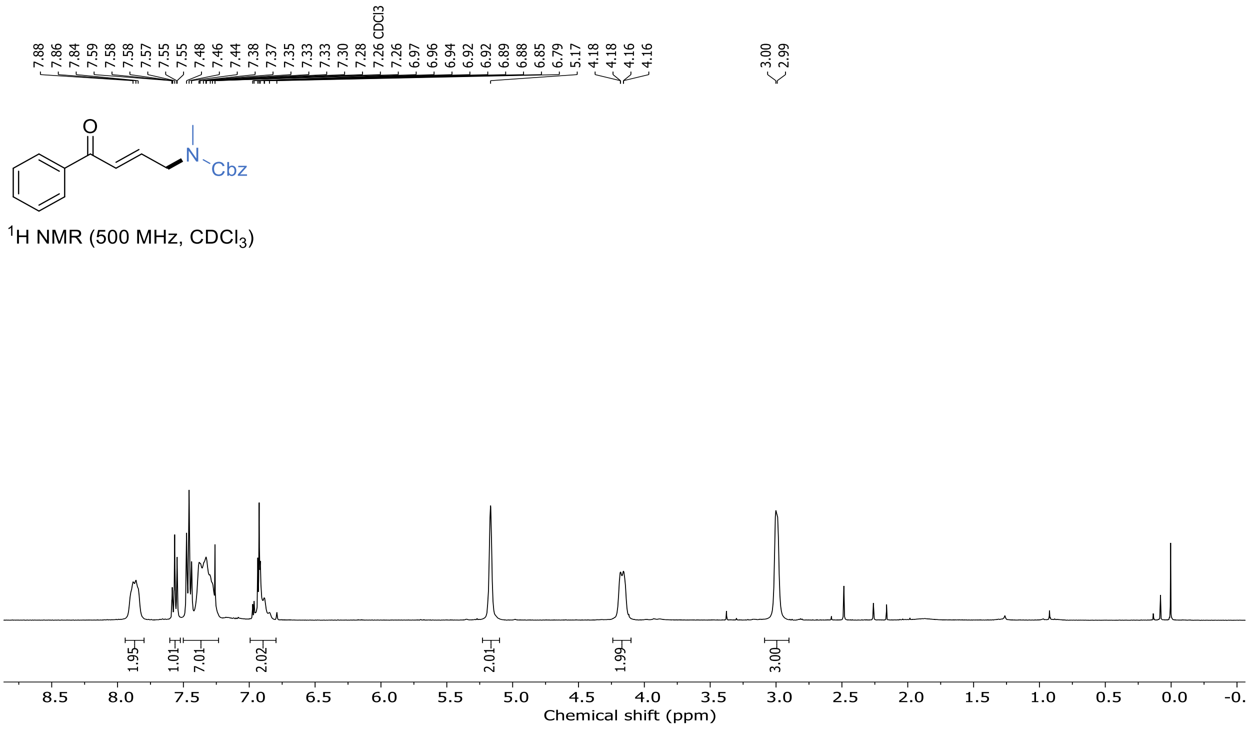

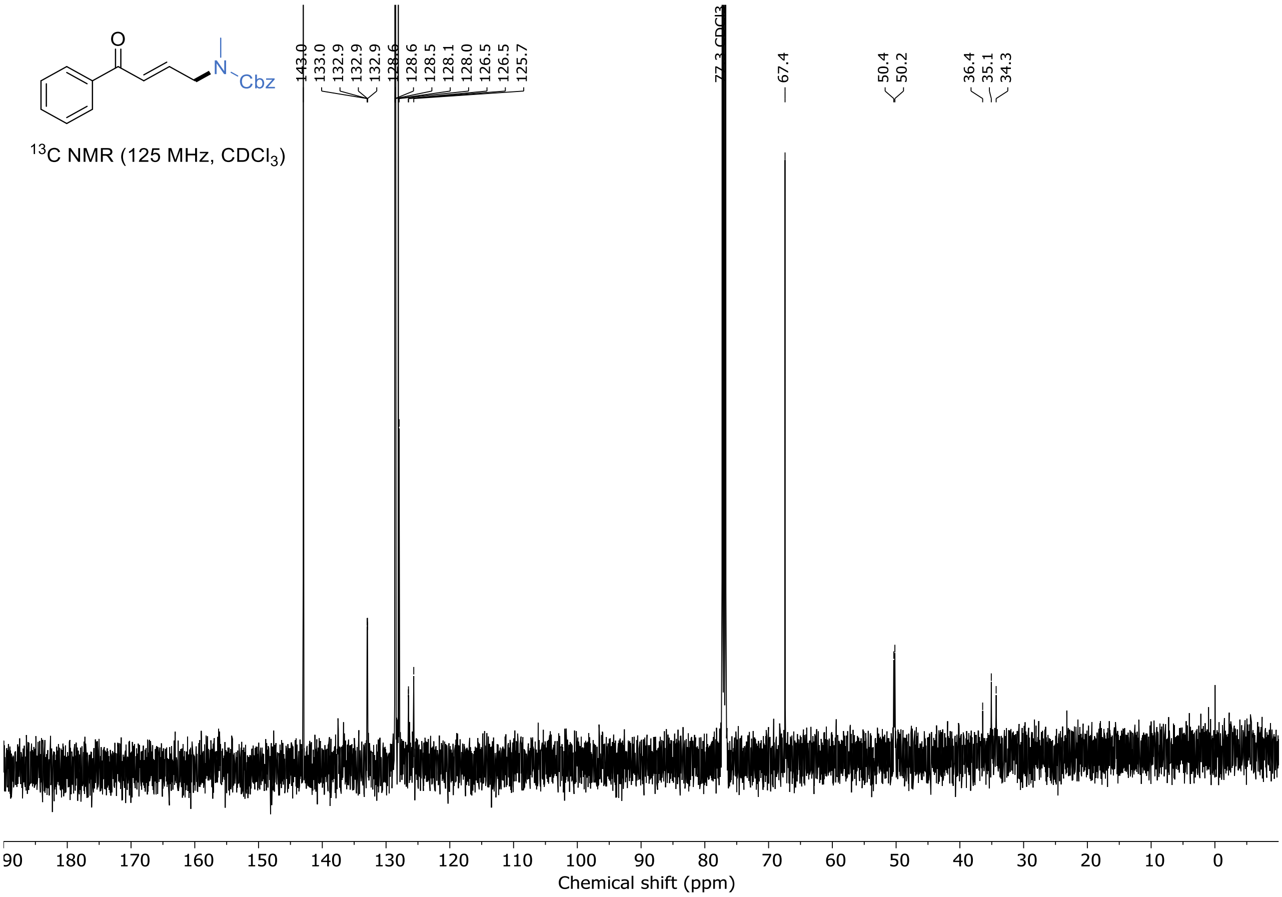

_N_,4-dimethyl-_N_-((2_E_,4_E_)-6-oxo-6-phenylhexa-2,4-dien-1-yl)benzenesulfonamide (19)

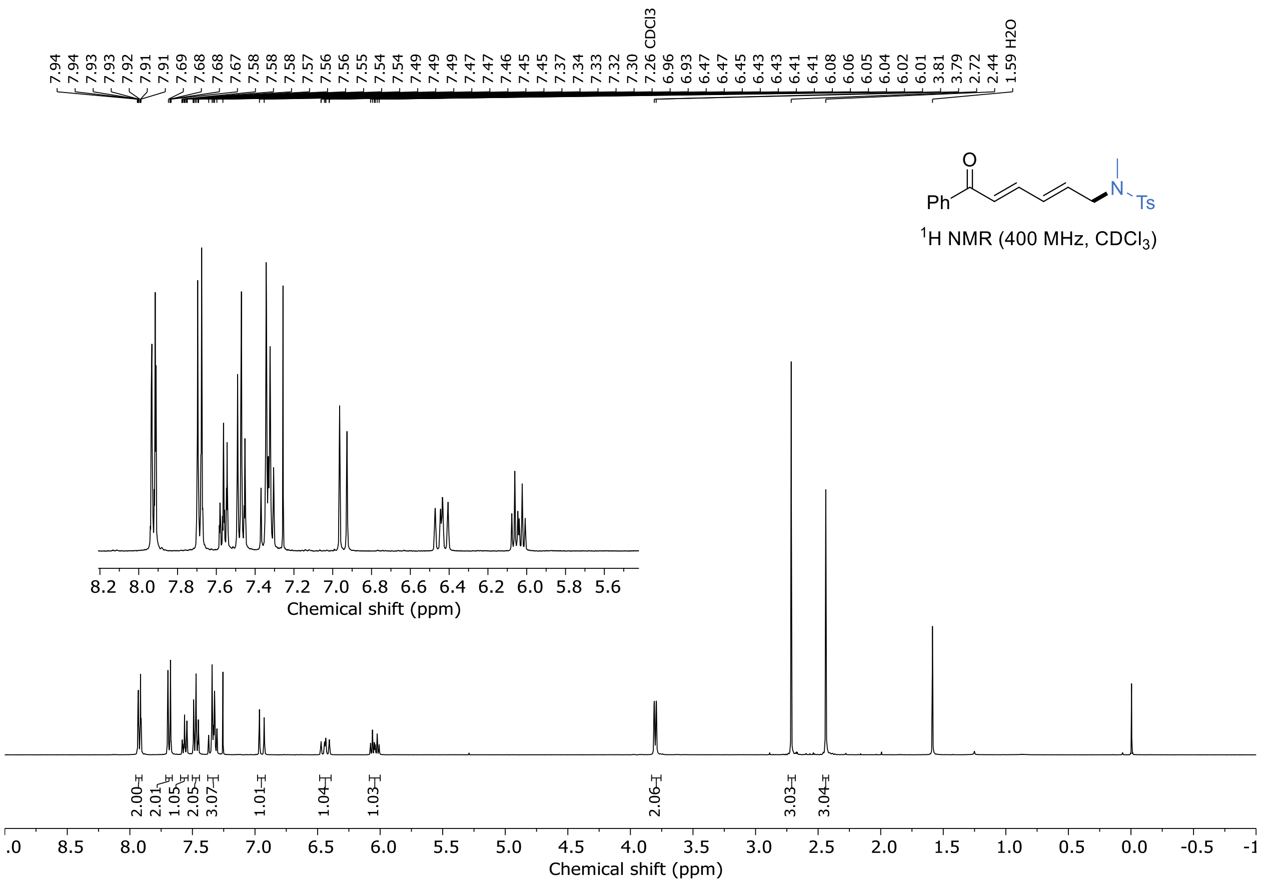

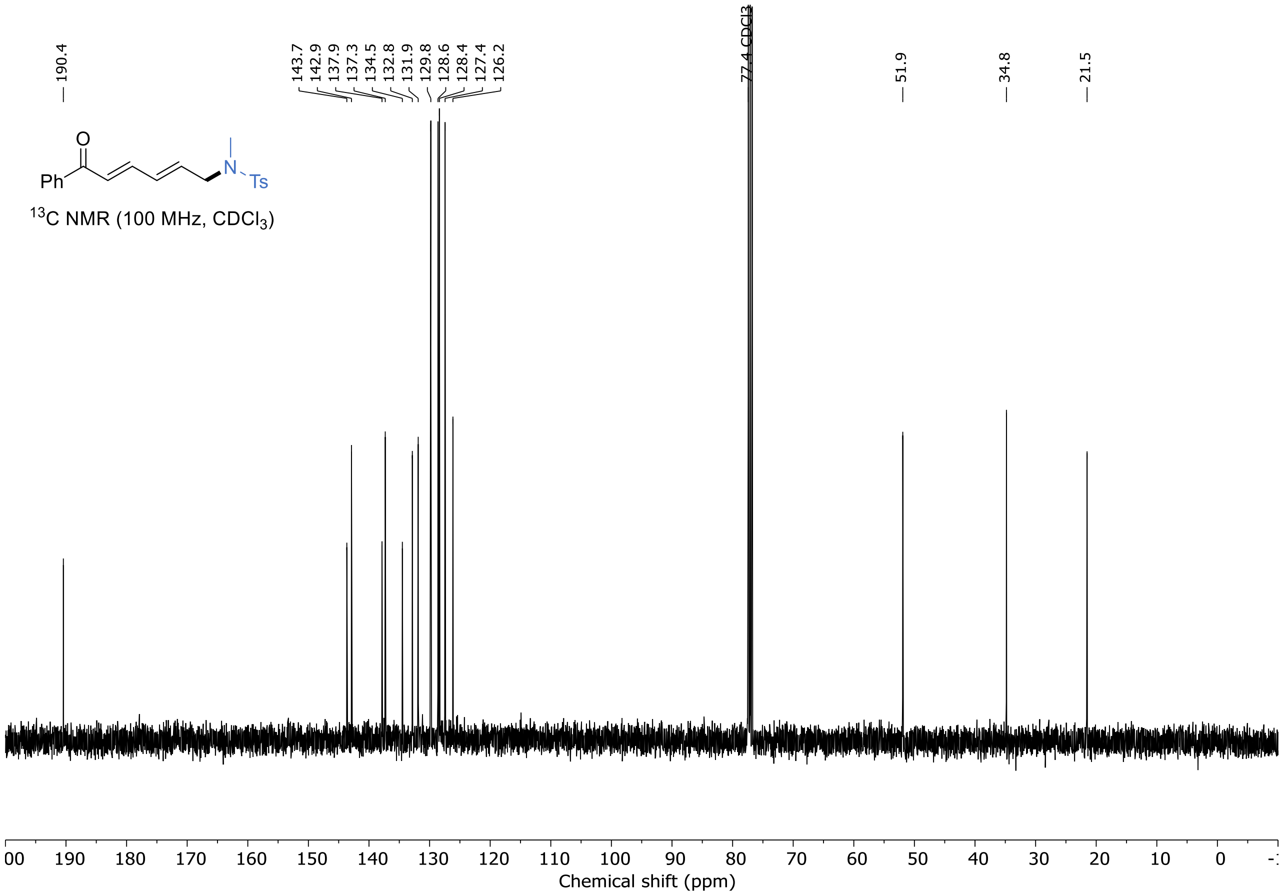

***N*,4-dimethyl-*N*-((2*E*,4*E*,6*E*)-8-oxo-8-phenylocta-2,4,6-trien-1-yl)benzenesulfonamide (20)**

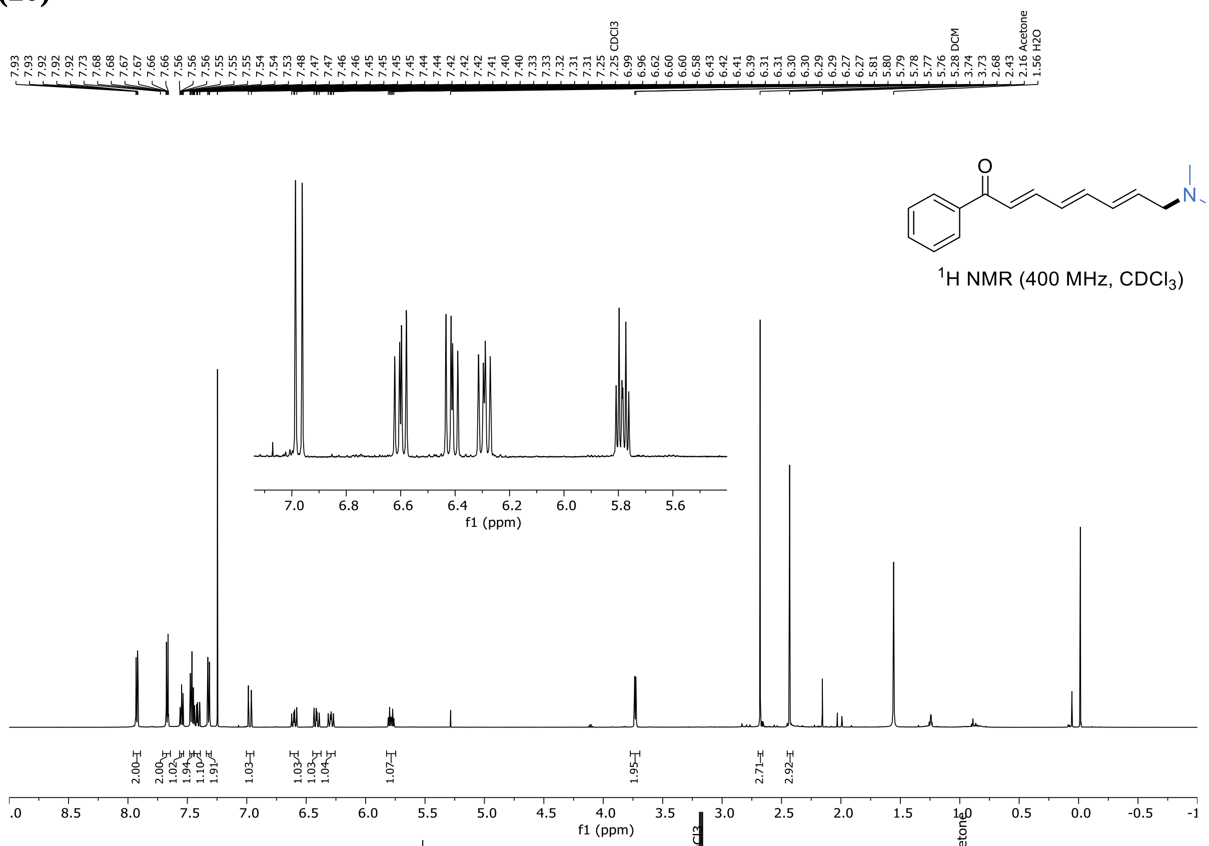

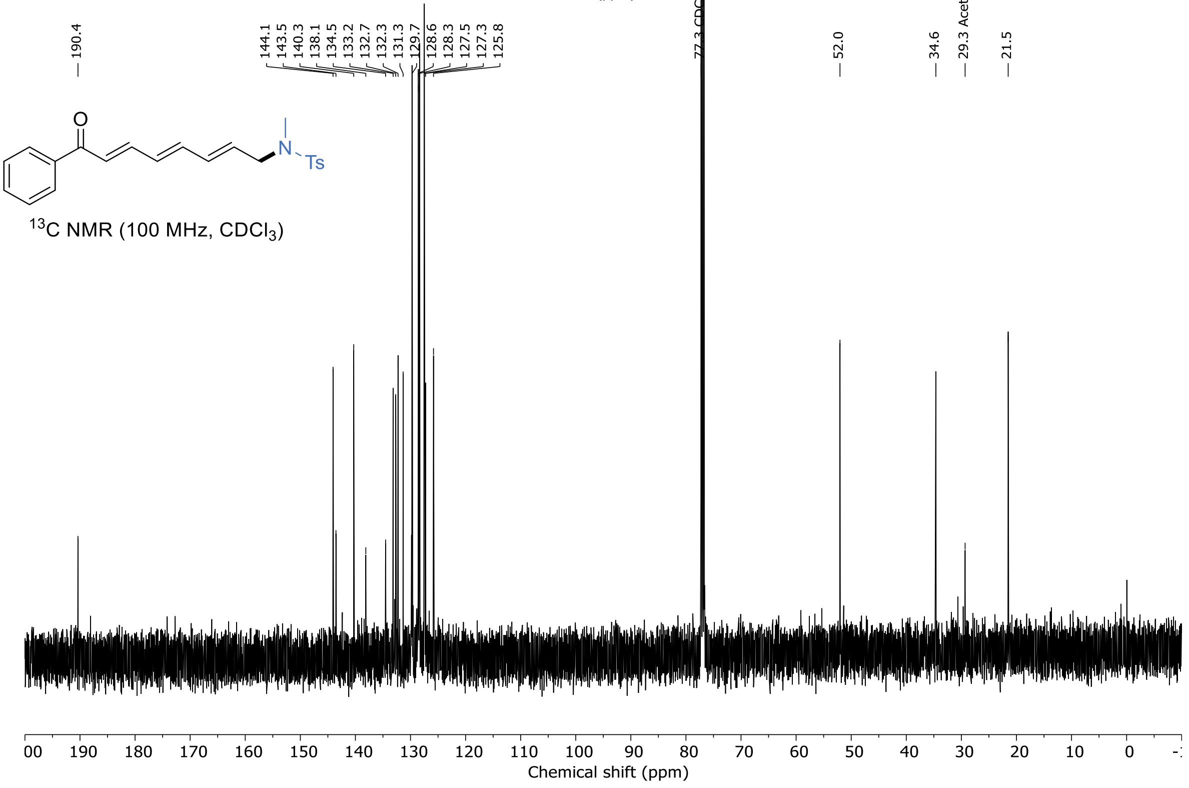

(*E*)-*N*-(6-(4-methoxyphenyl)-6-oxohex-4-en-3-yl)-*N*,4-dimethylbenzenesulfonamide (4)

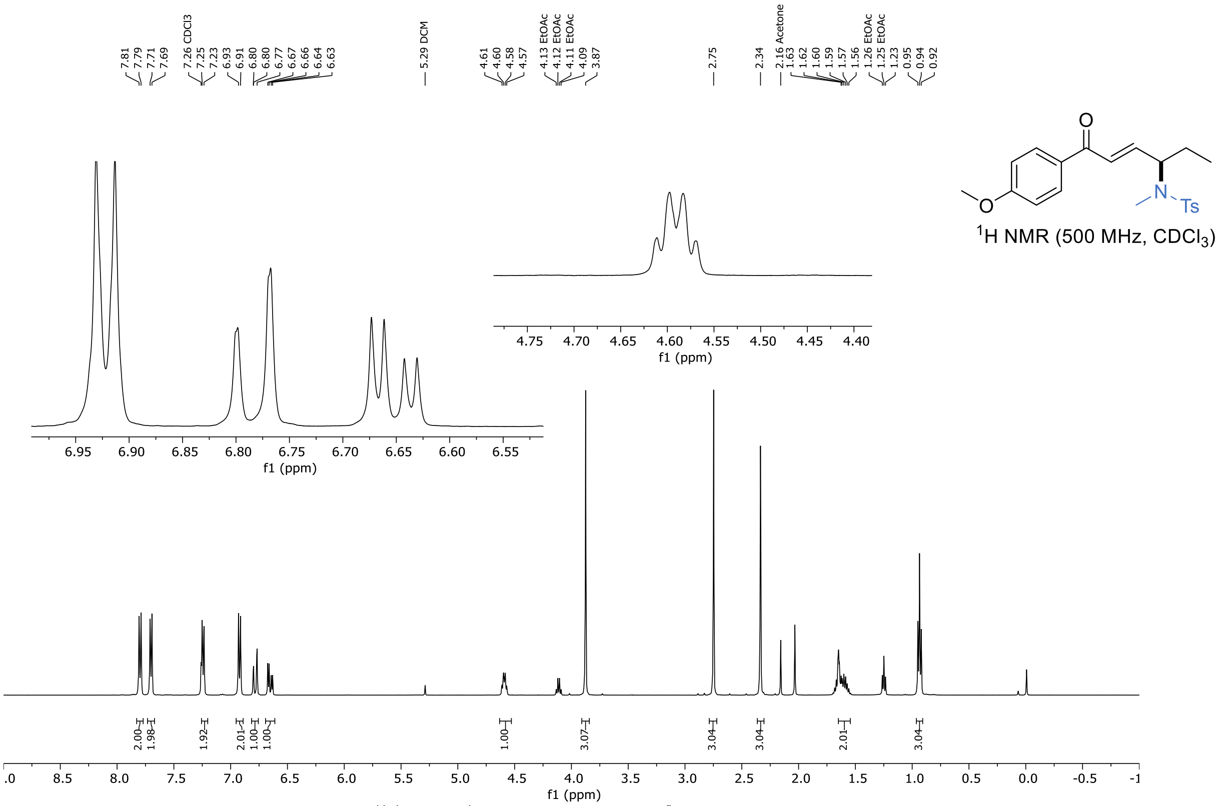

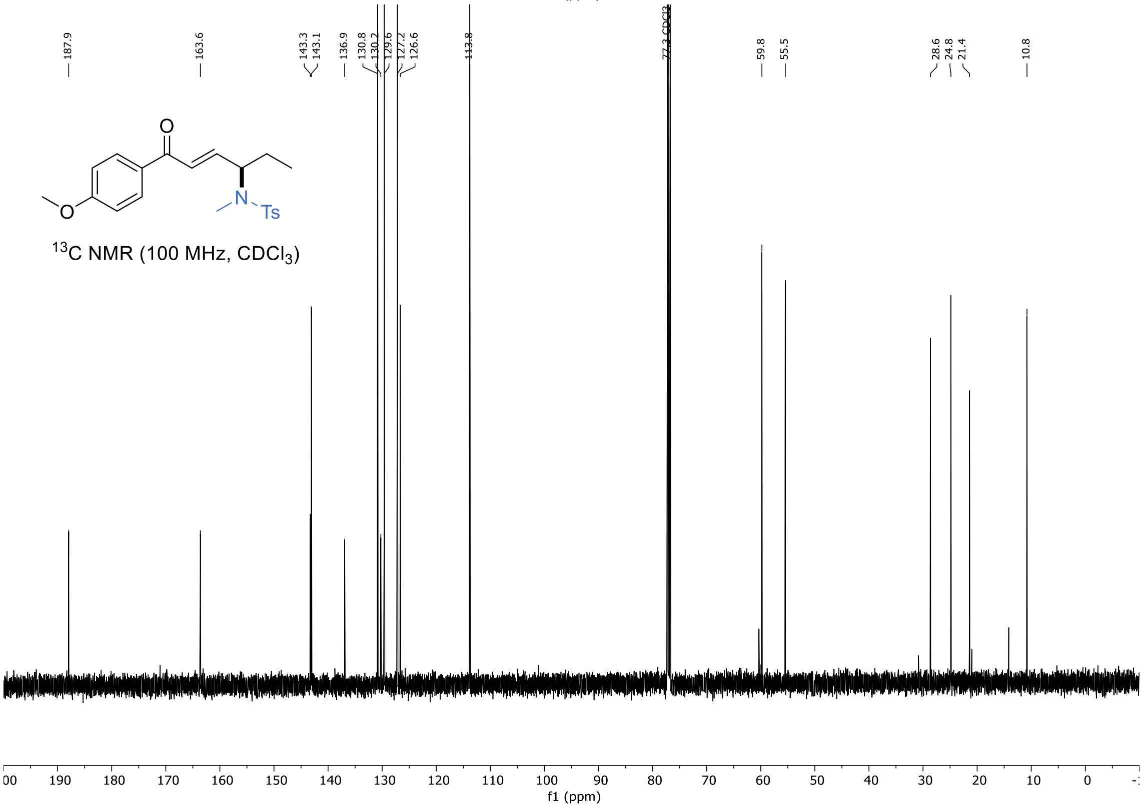

(*E*)-*N*-(6-(4-(*tert*-butyl)phenyl)-6-oxohex-4-en-3-yl)-*N*,4-dimethylbenzenesulfonamide (8)

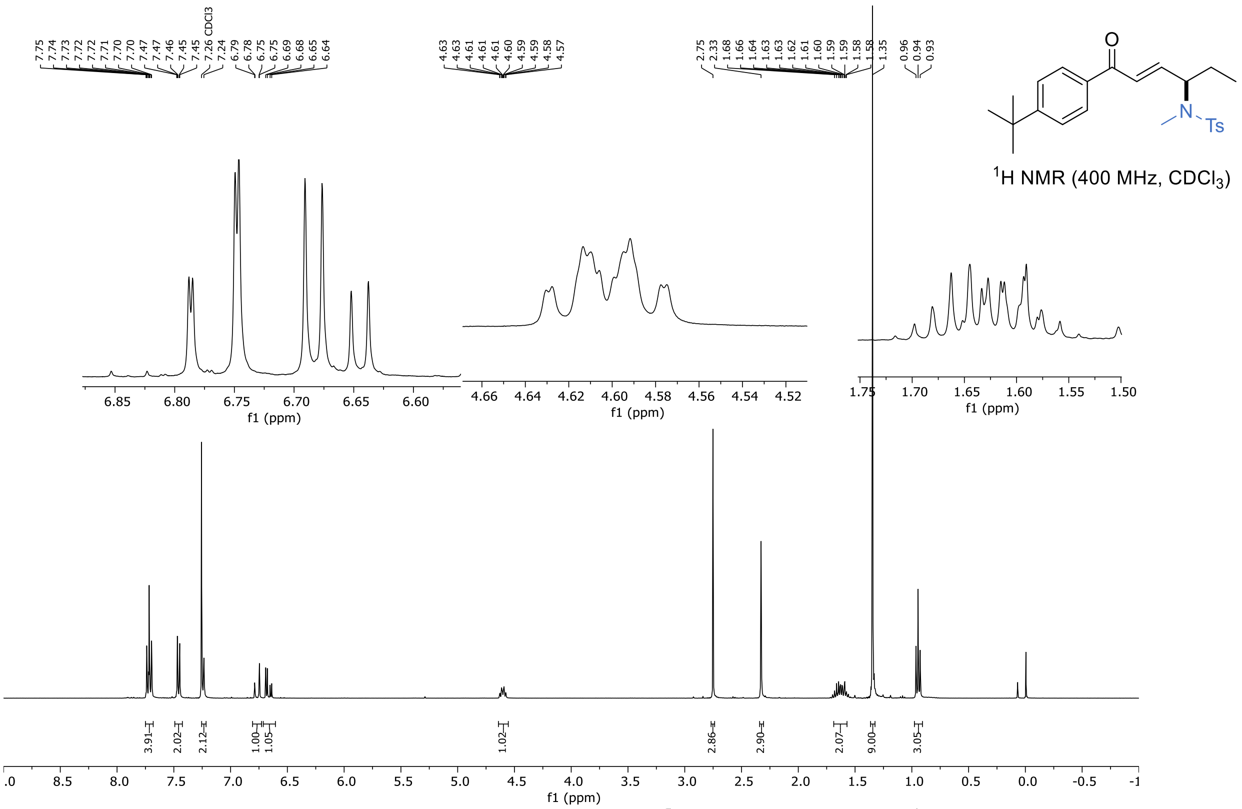

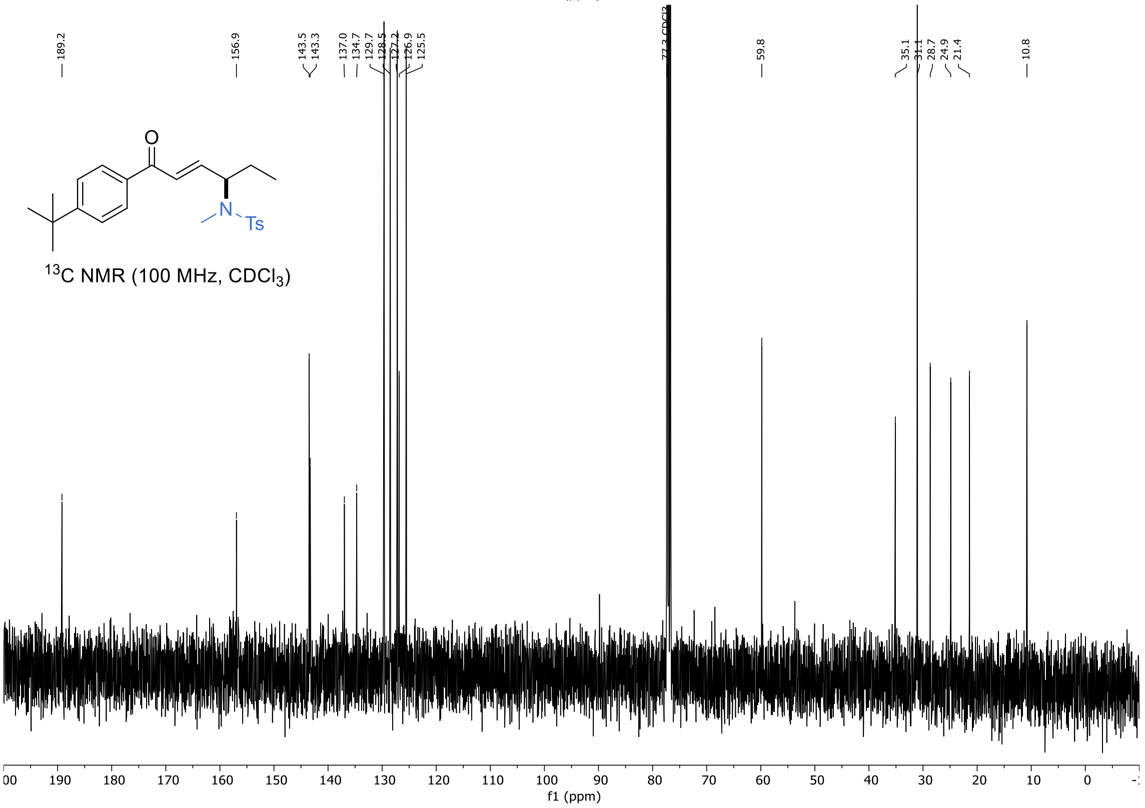

(*E*)-*N*-(6-(4-chlorophenyl)-6-oxohex-4-en-3-yl)-*N*,4-dimethylbenzenesulfonamide (6)

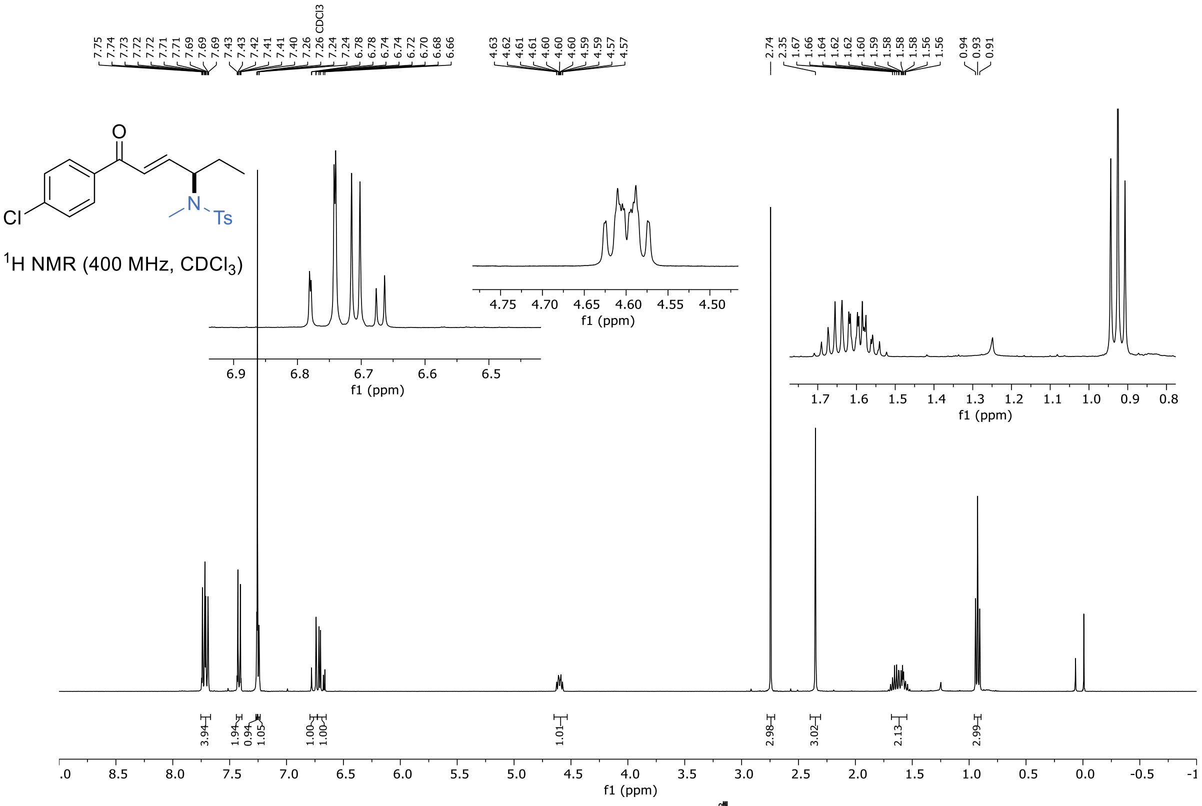

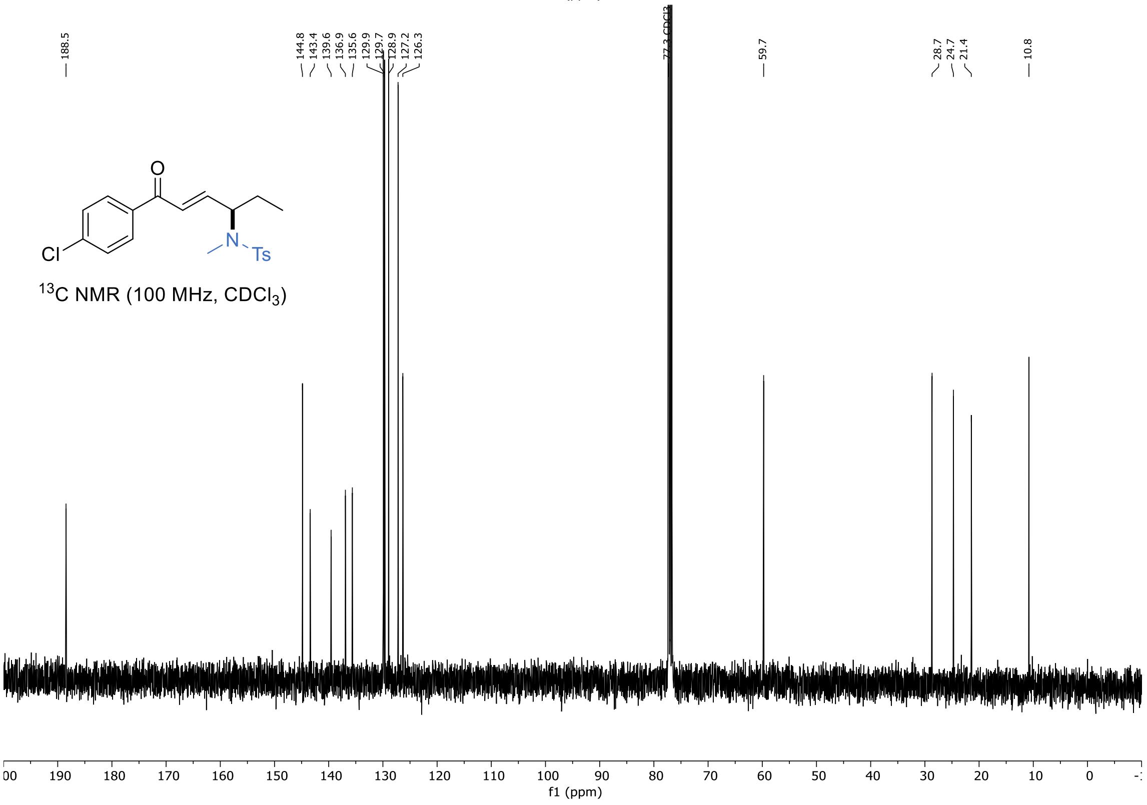

(*E*)-*N*-(6-(4-bromophenyl)-6-oxohex-4-en-3-yl)-*N*,4-dimethylbenzenesulfonamide (7)

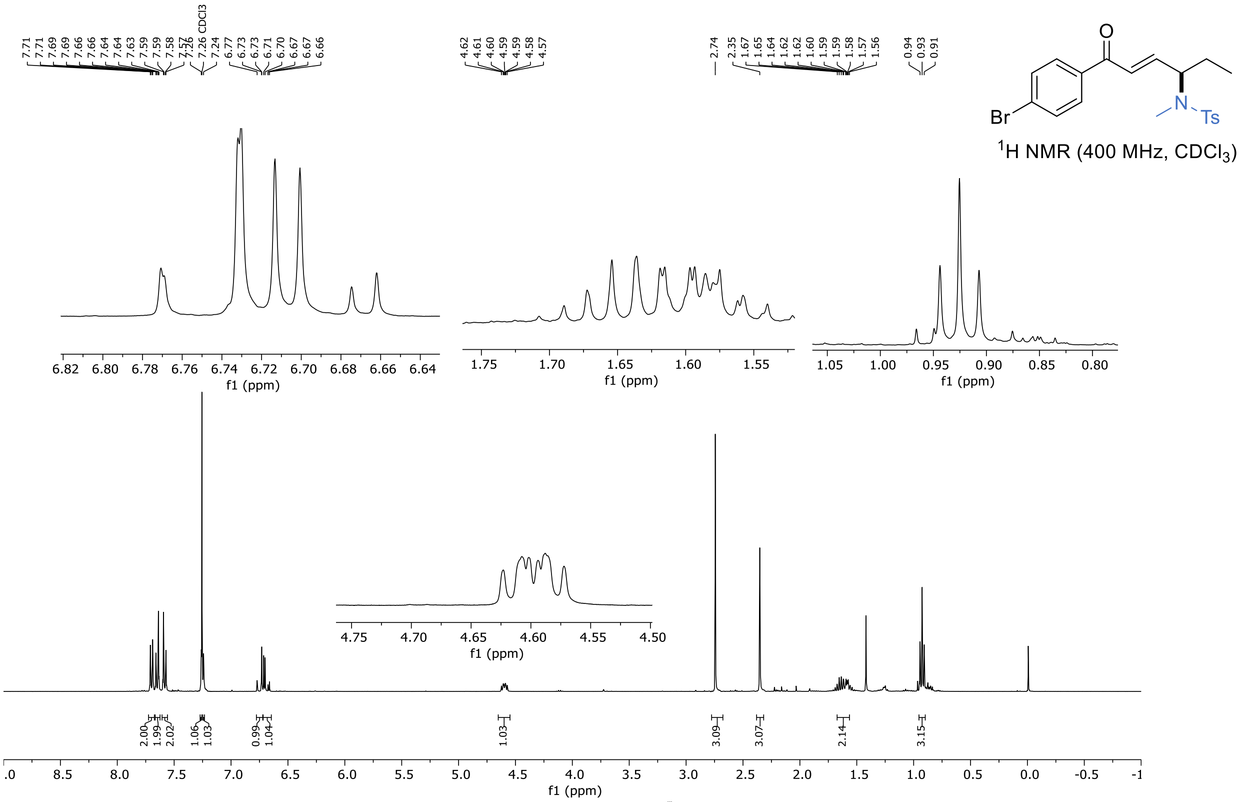

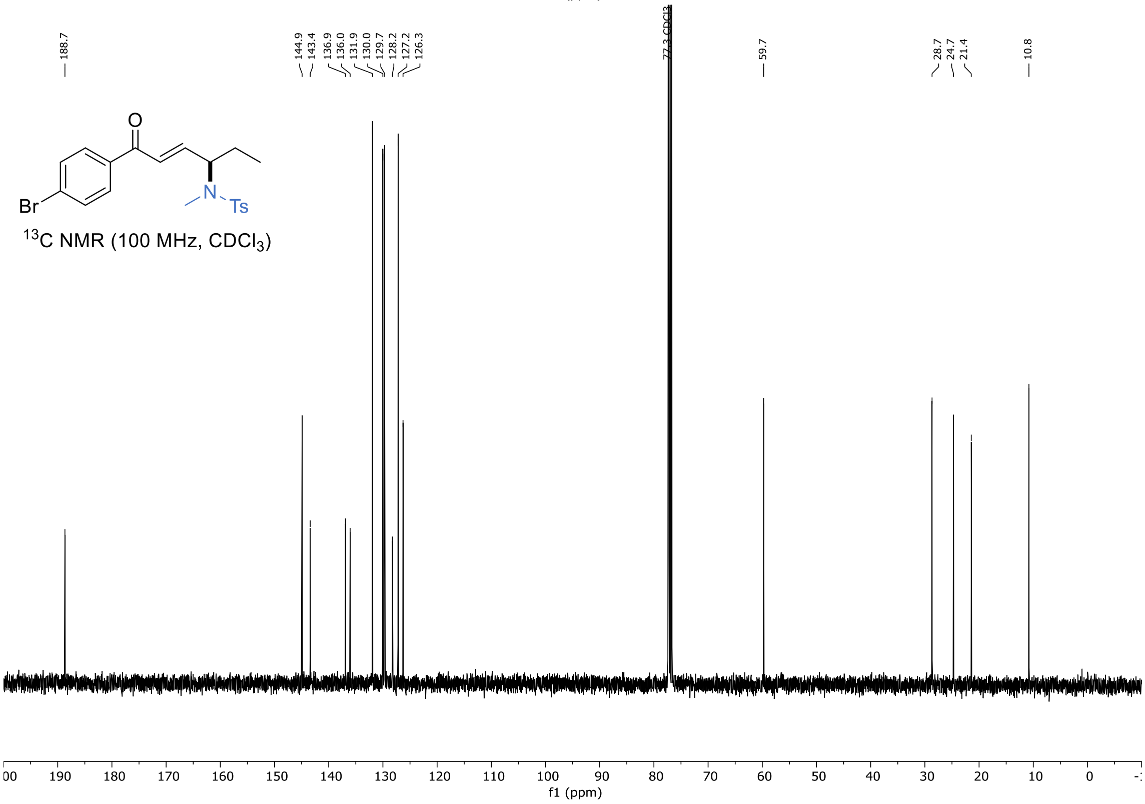

(*E*)-*N*-(6-(benzo[*d*][1,3]dioxol-5-yl)-6-oxohex-4-en-3-yl)-*N*,4-dimethylbenzenesulfonamide (5)

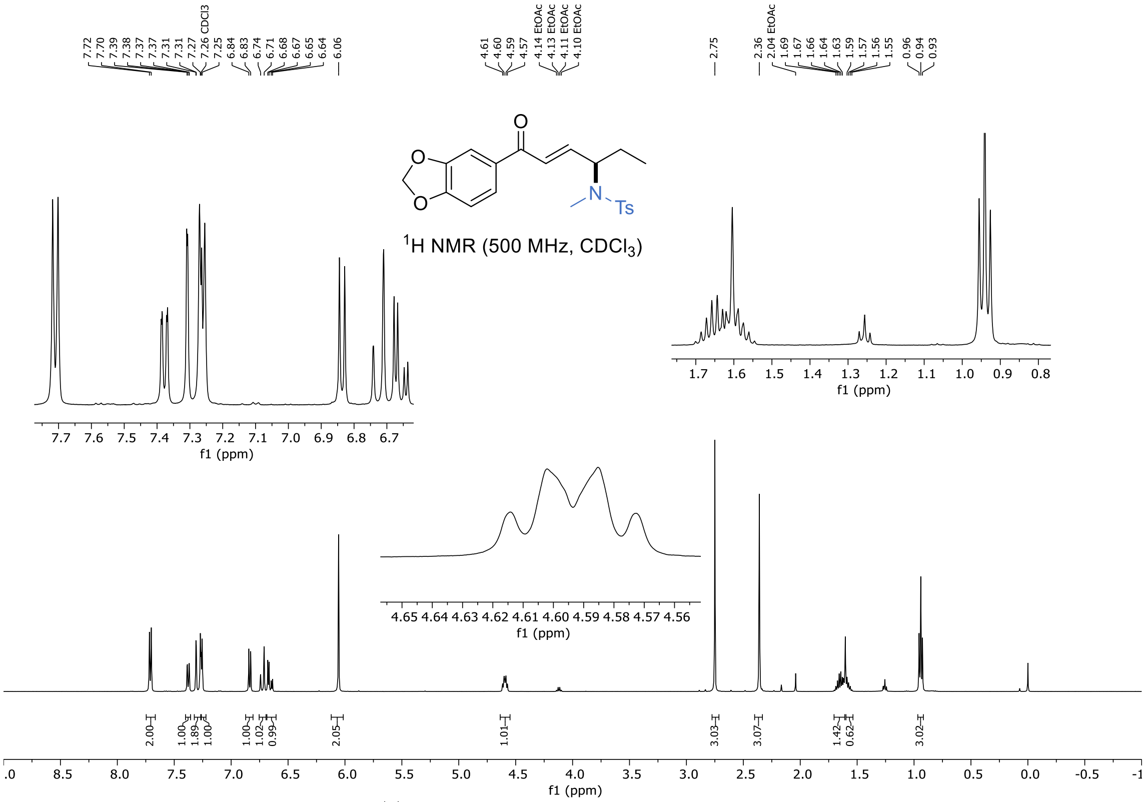

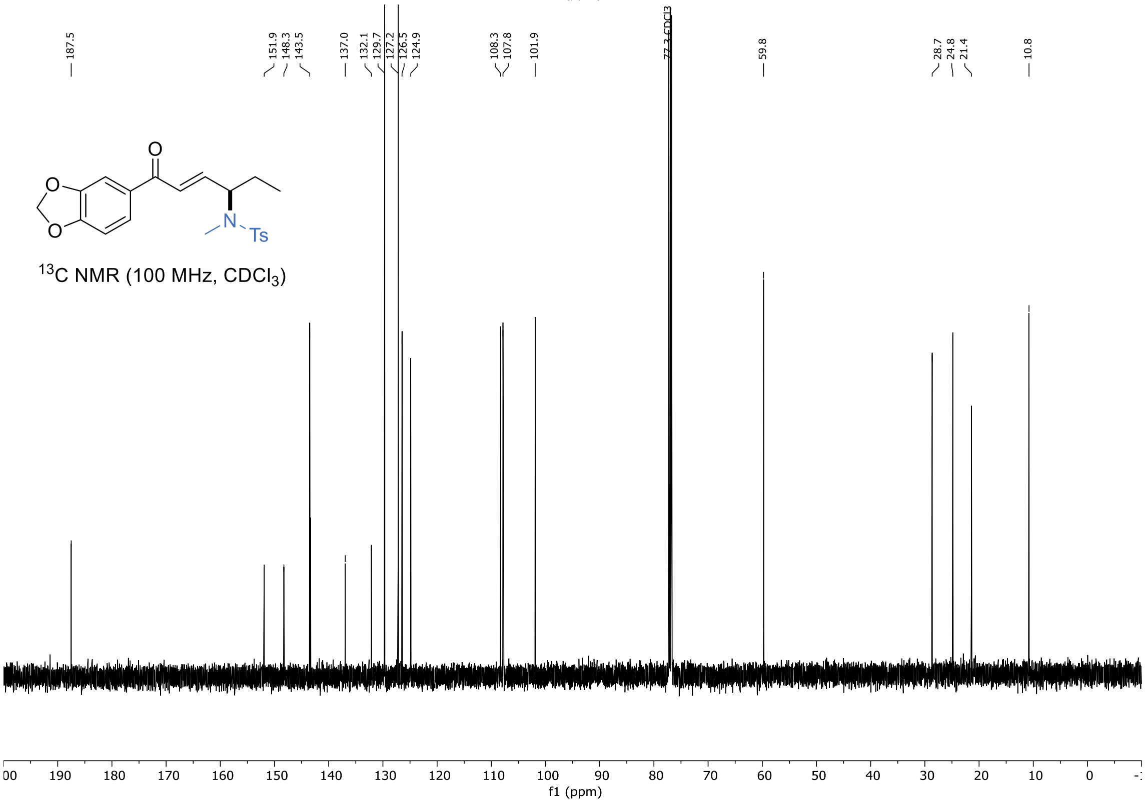

(*E*)-*N*,4-dimethyl-*N*-(6-(4-nitrophenyl)-6-oxohex-4-en-3-yl)benzenesulfonamide (11)

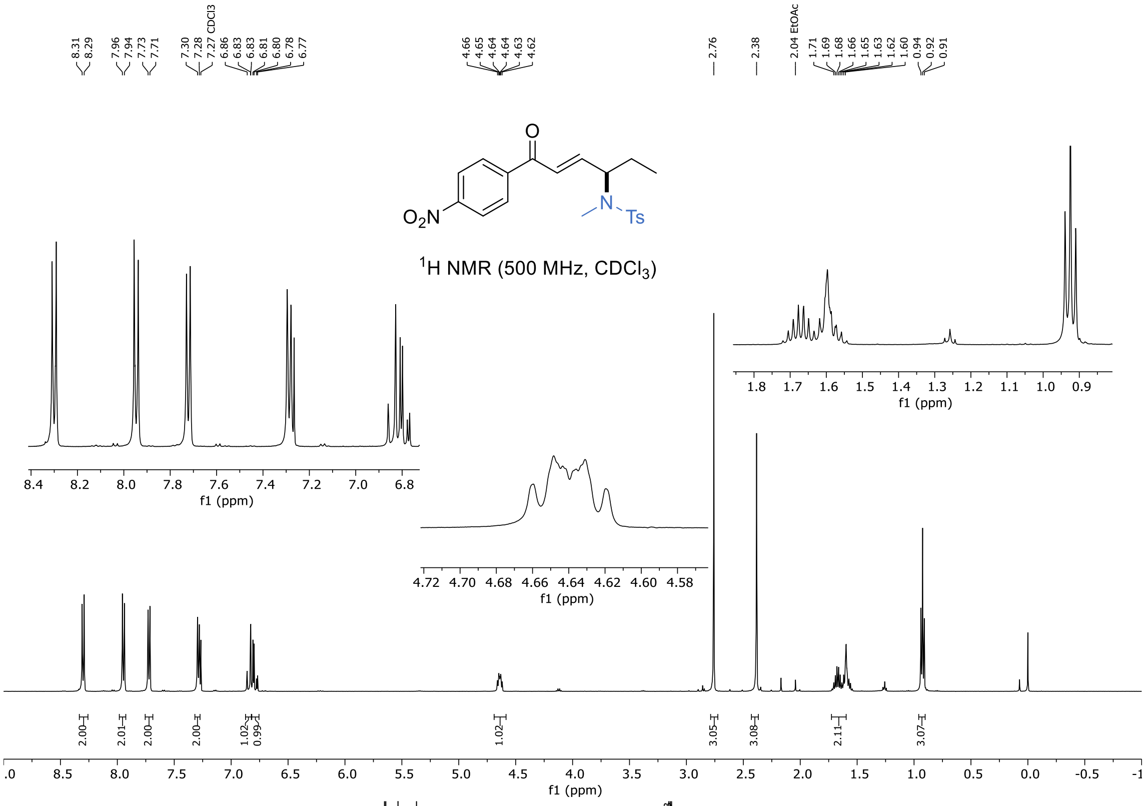

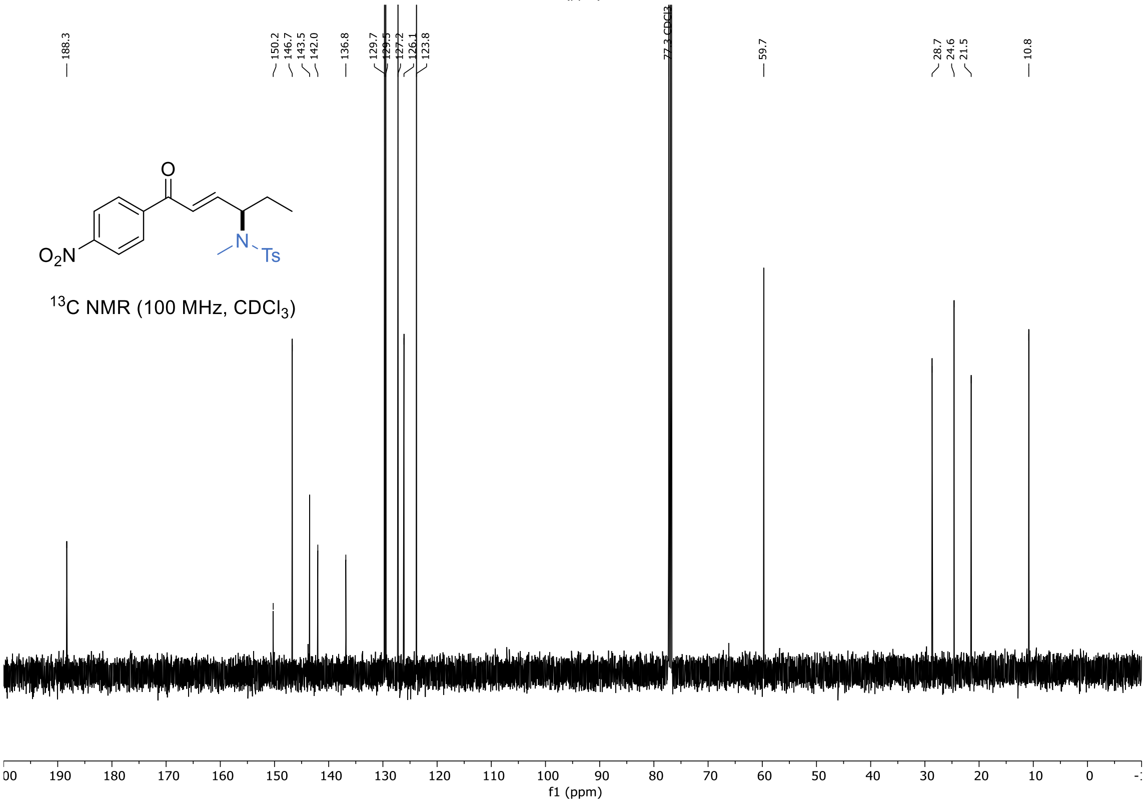

(*E*)-*N*-(6-(4-acetylphenyl)-6-oxohex-4-en-3-yl)-*N*,4-dimethylbenzenesulfonamide (10)

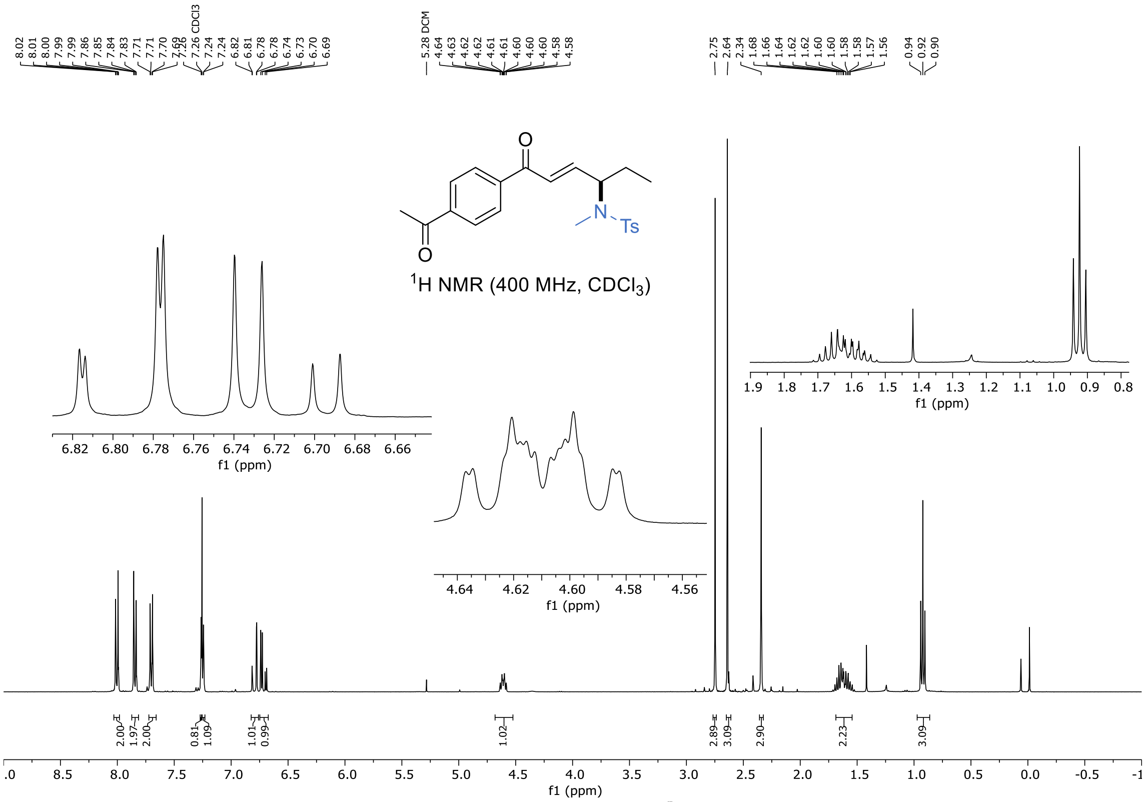

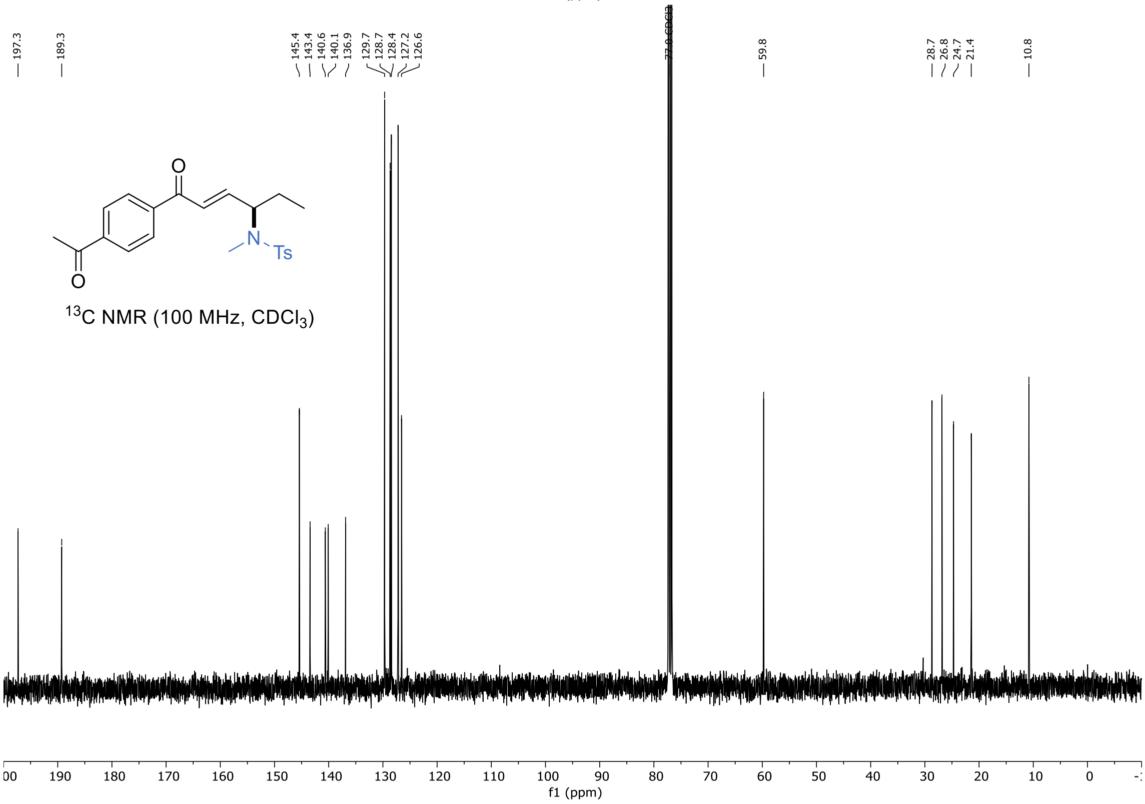

(*E*)-*N*-(6-(4-cyanophenyl)-6-oxohex-4-en-3-yl)-*N*,4-dimethylbenzenesulfonamide (9)

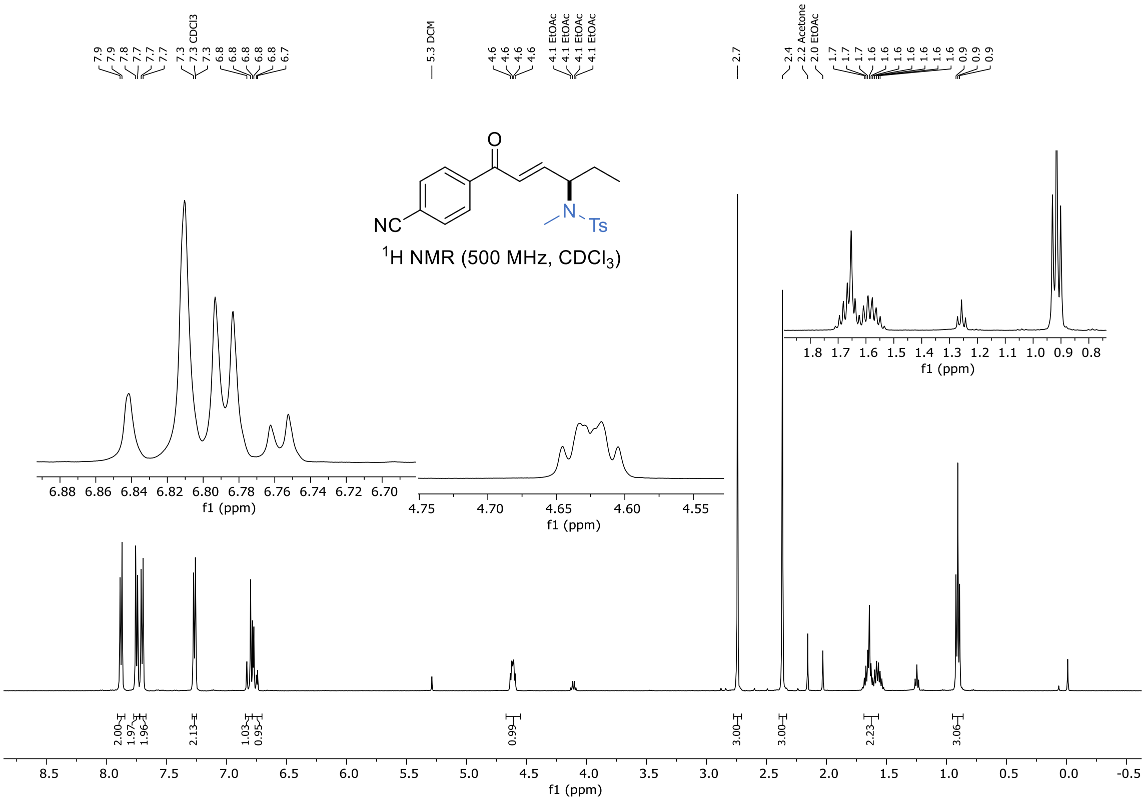

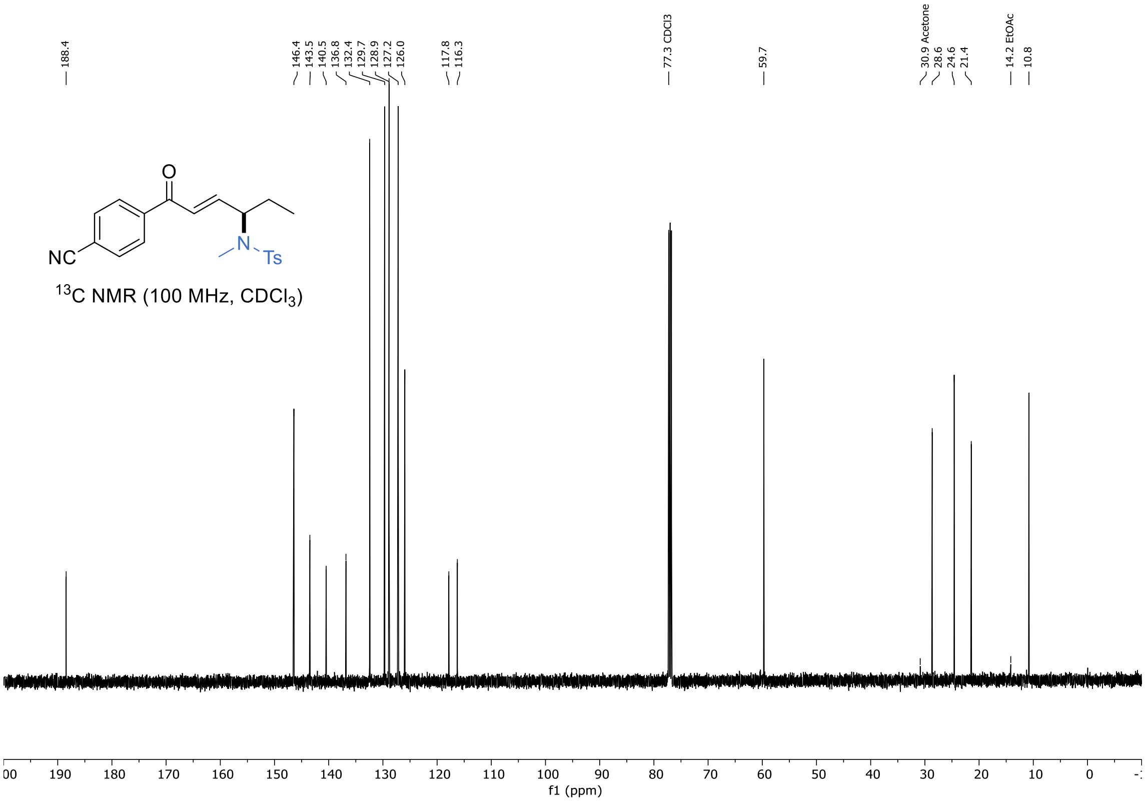

(*E*)-*N*-(1,4-bis(4-methoxyphenyl)-4-oxobut-2-en-1-yl)-*N*,4-dimethylbenzenesulfonamide (13a)

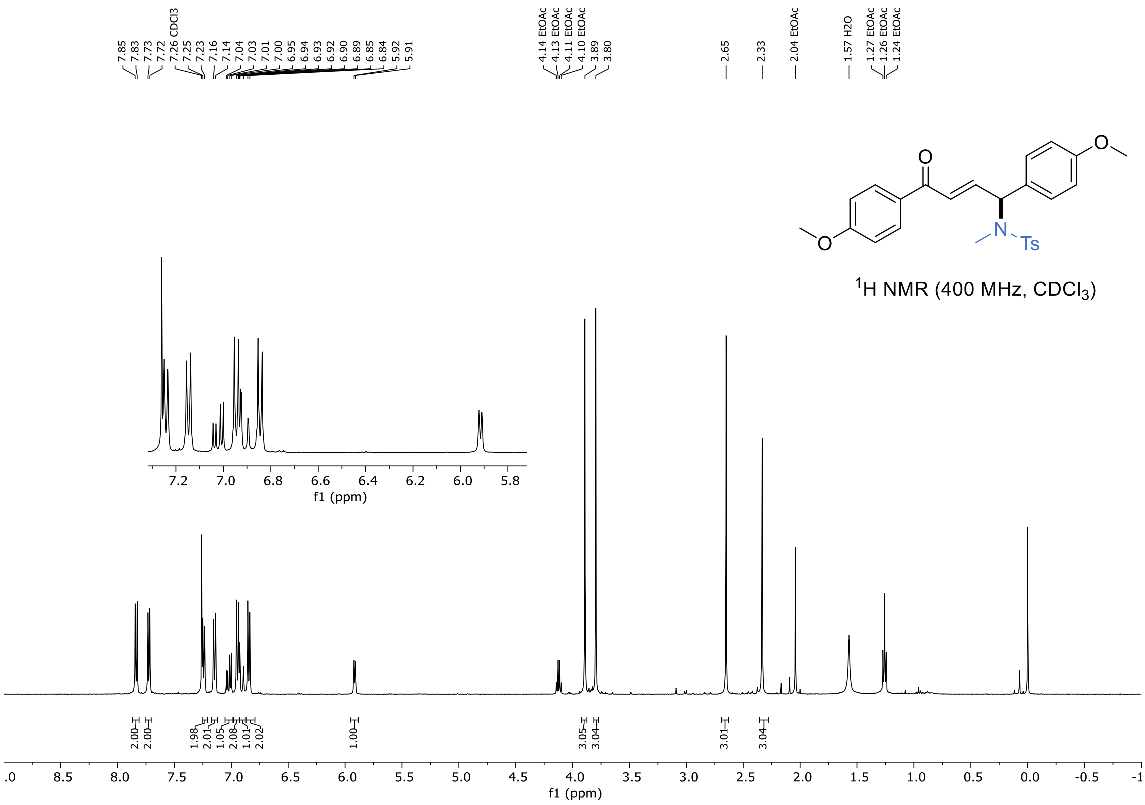

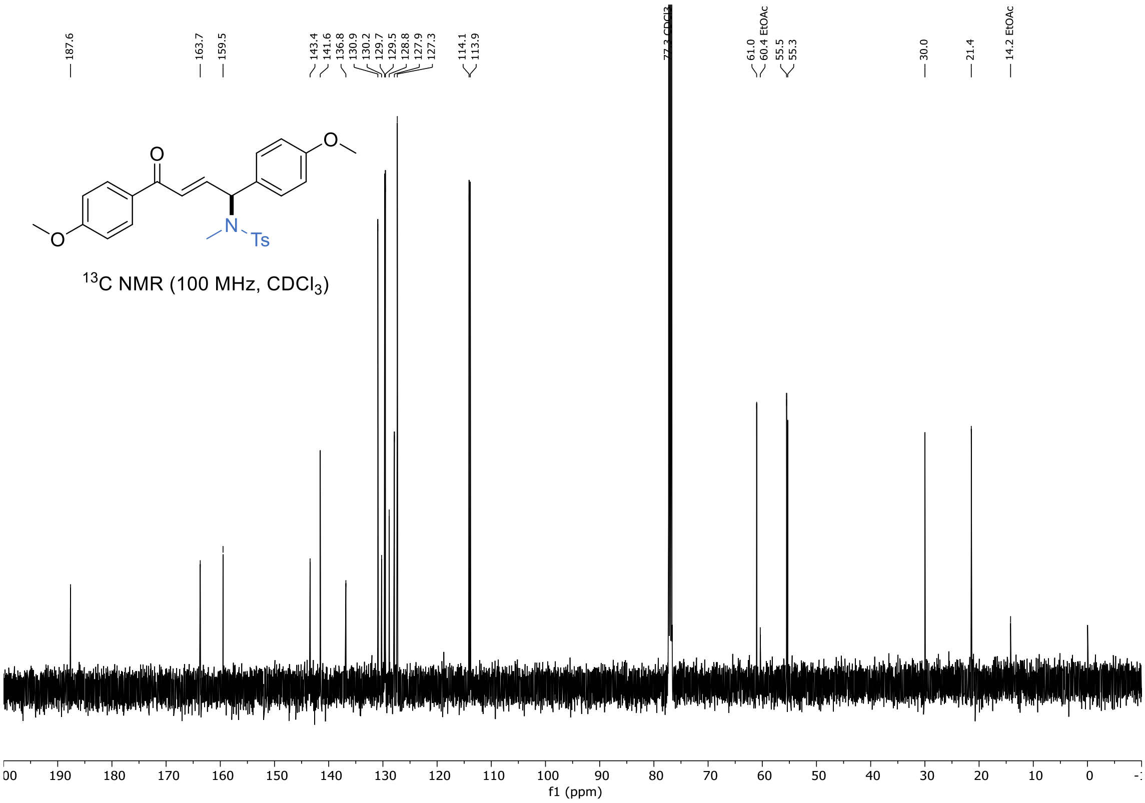

(*Z*)-*N*-(1-(4-methoxyphenyl)-1-oxo-4-(4-(trifluoromethyl)phenyl)but-2-en-2-yl)-*N*,4-dimethylbenzenesulfonamide (14c) (Two isomers)

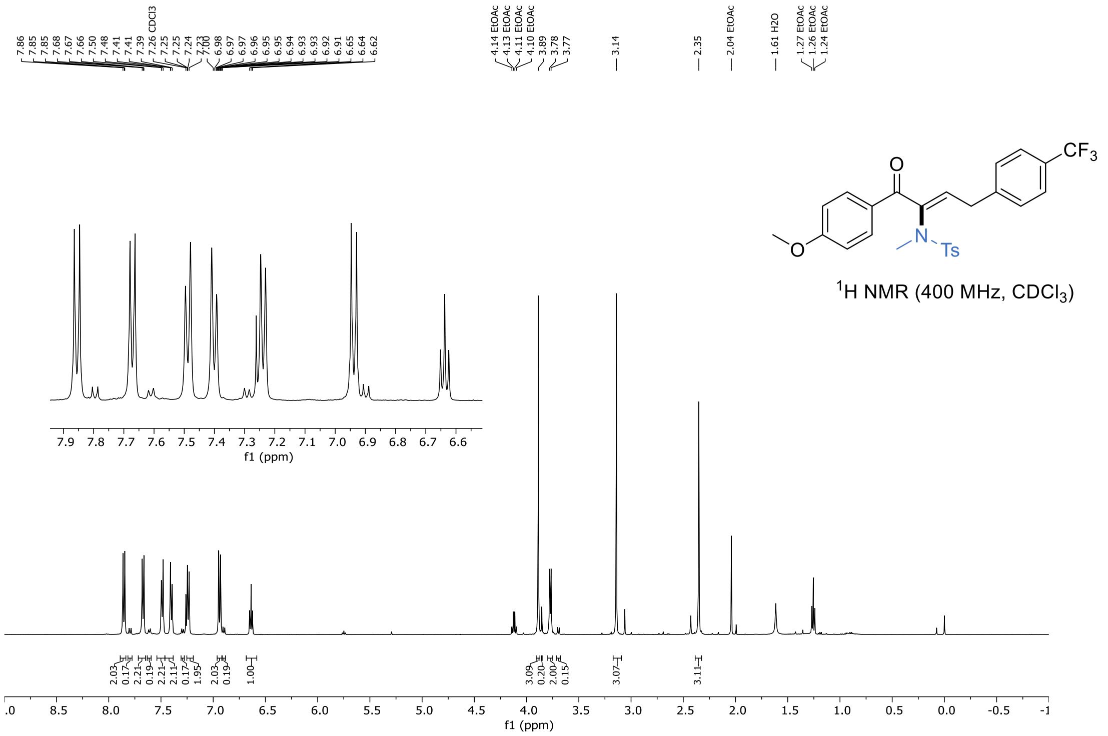

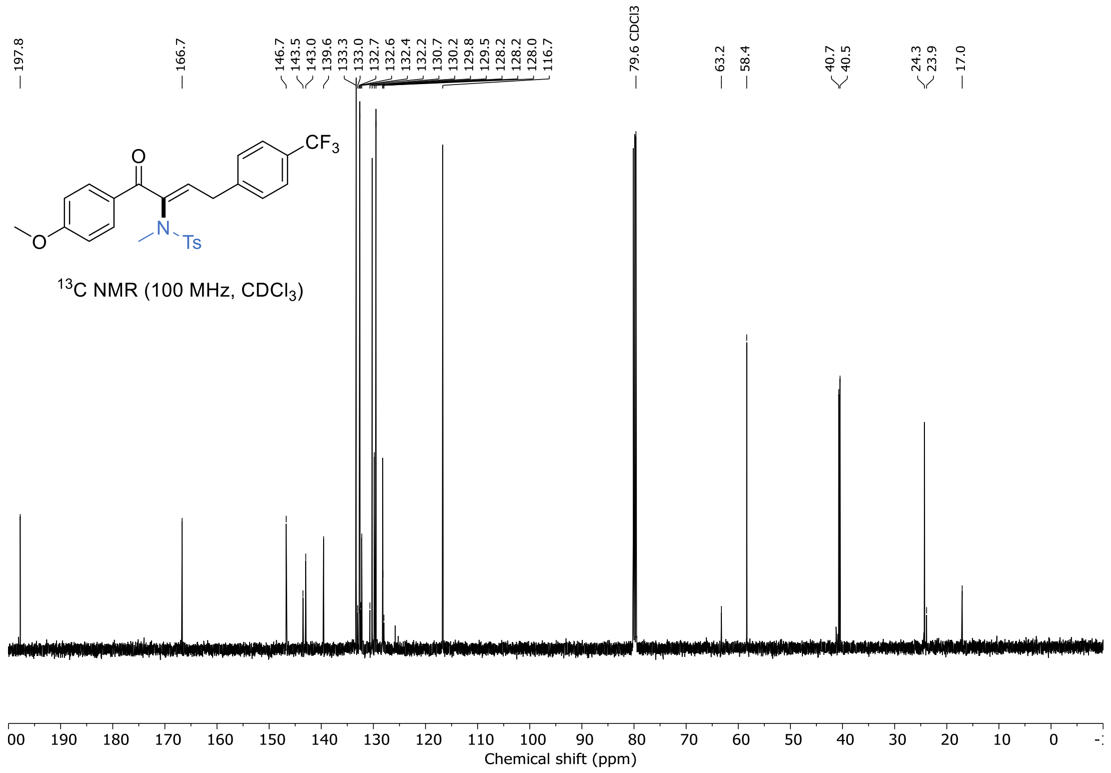

Major α isomer with traces of β-regioisomer

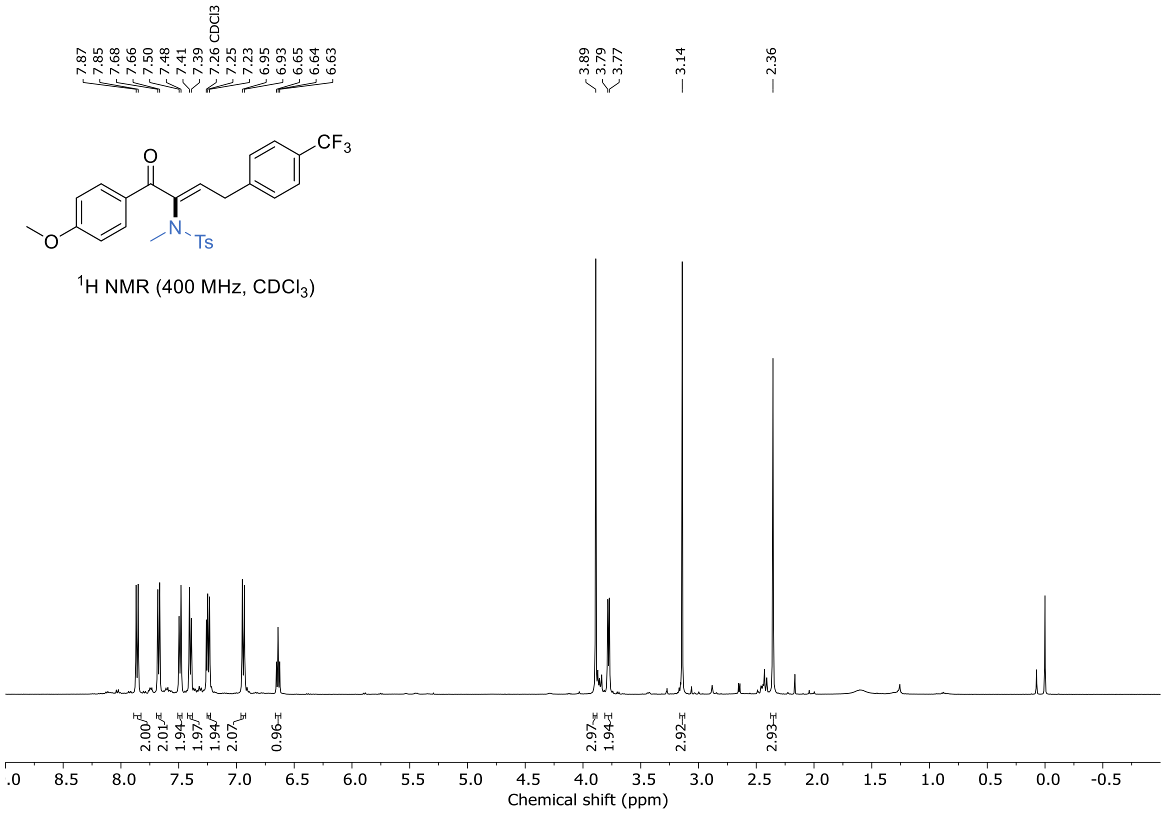

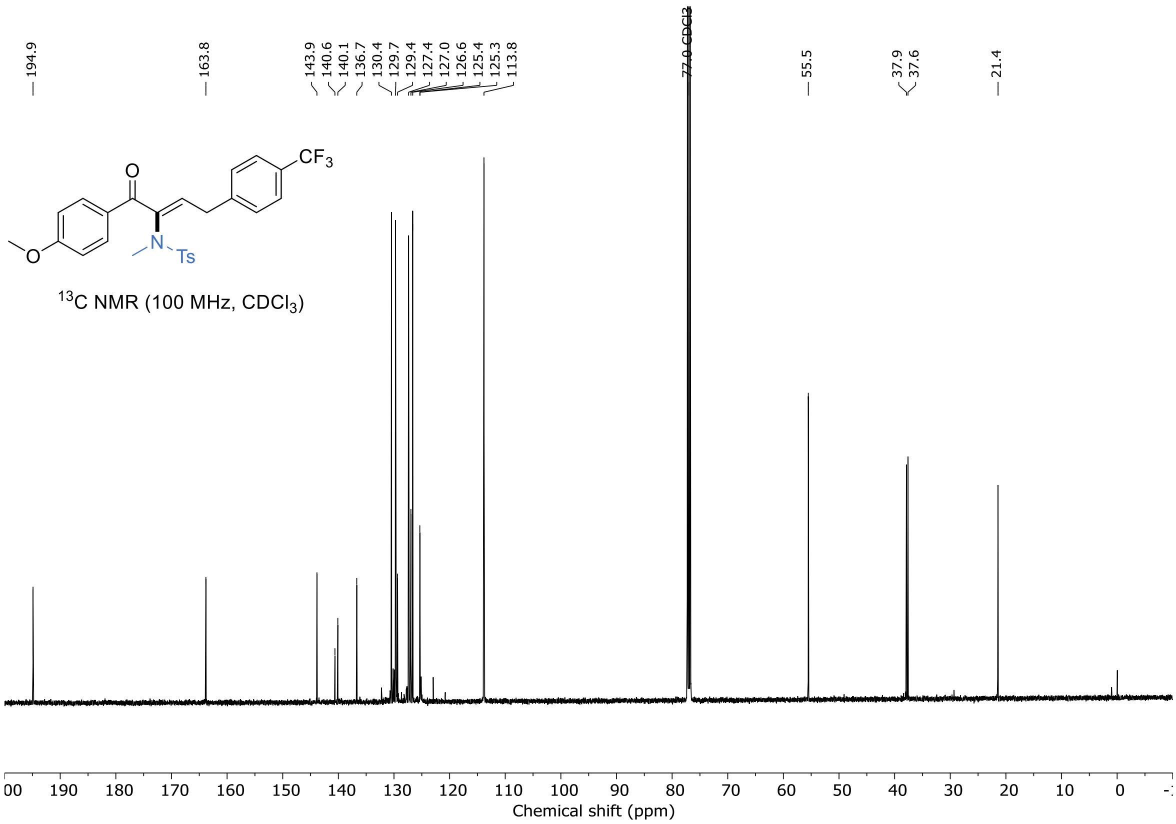

(*Z*)-*N*,4-dimethyl-N-(1-oxo-1,4-bis(4-(trifluoromethyl)phenyl)but-2-en-2-yl)benzenesulfonamide (14b)

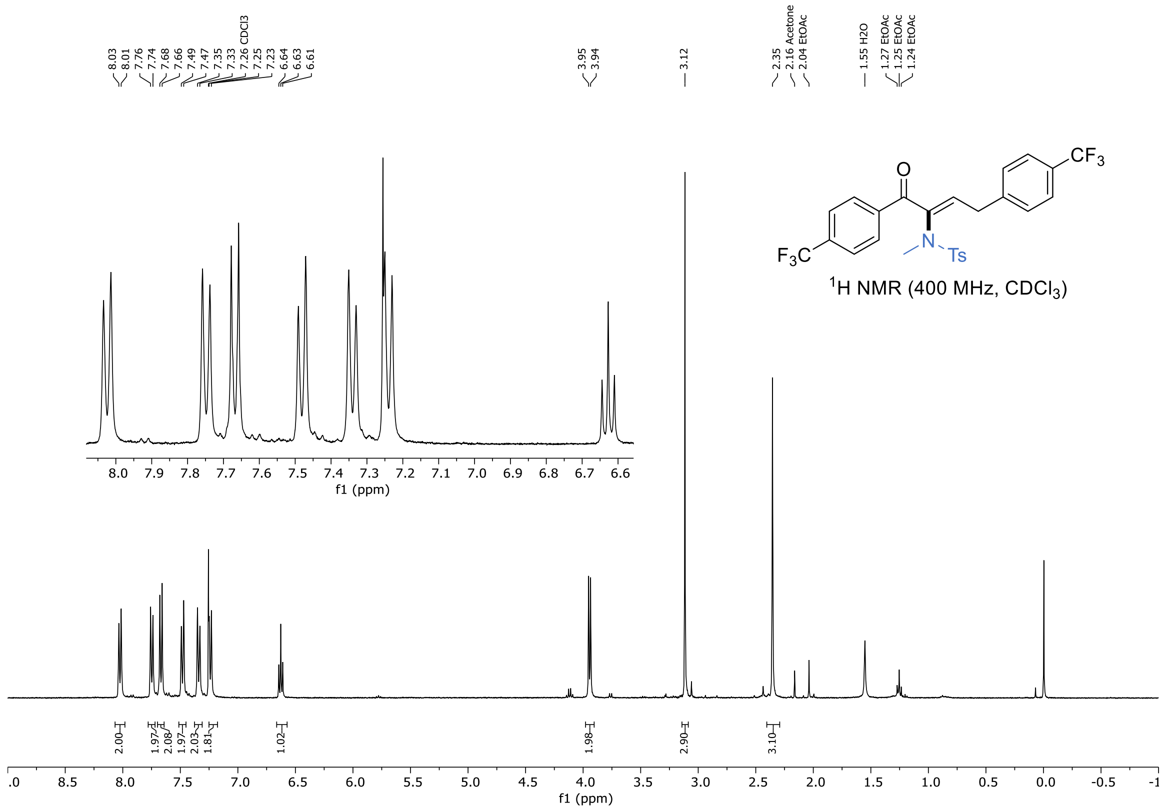

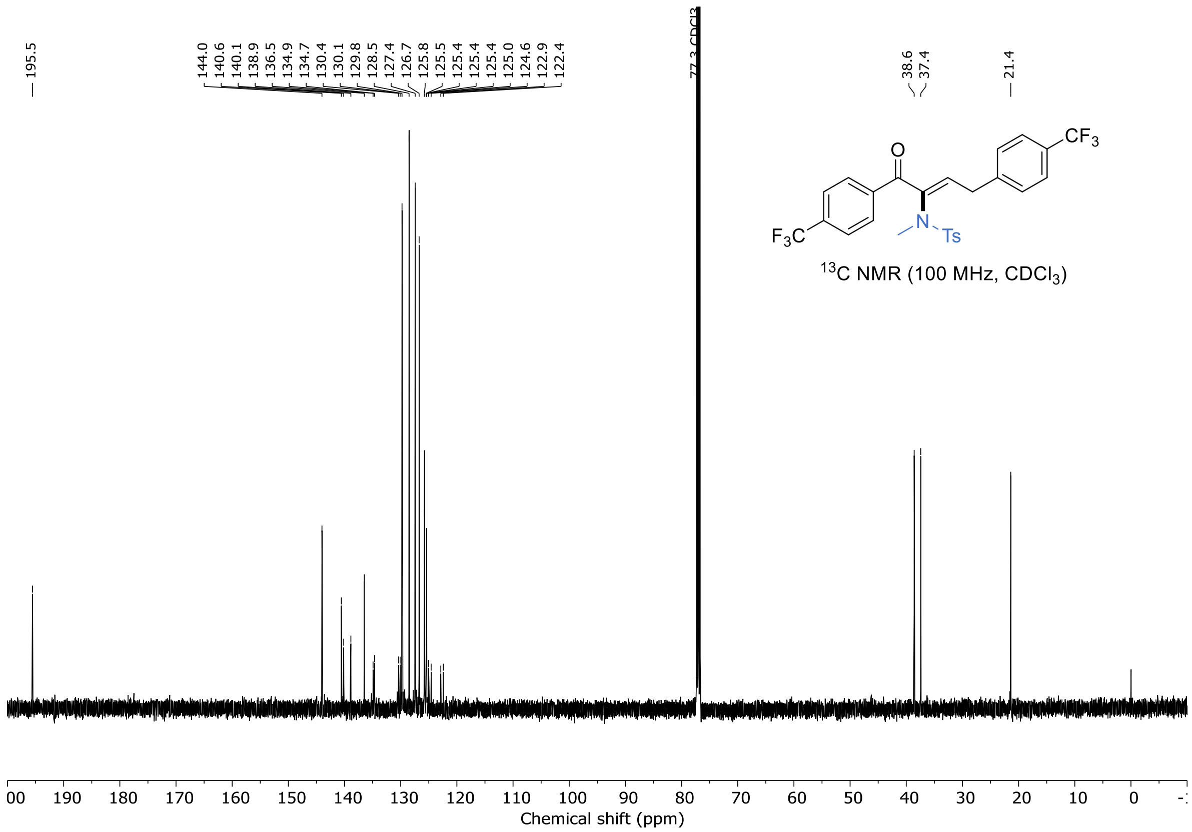

(*Z*)-*N*-(1-(4-methoxyphenyl)-4-oxo-4-(4-(trifluoromethyl)phenyl)but-2-en-1-yl)-*N*,4-dimethylbenzenesulfonamide (13d)

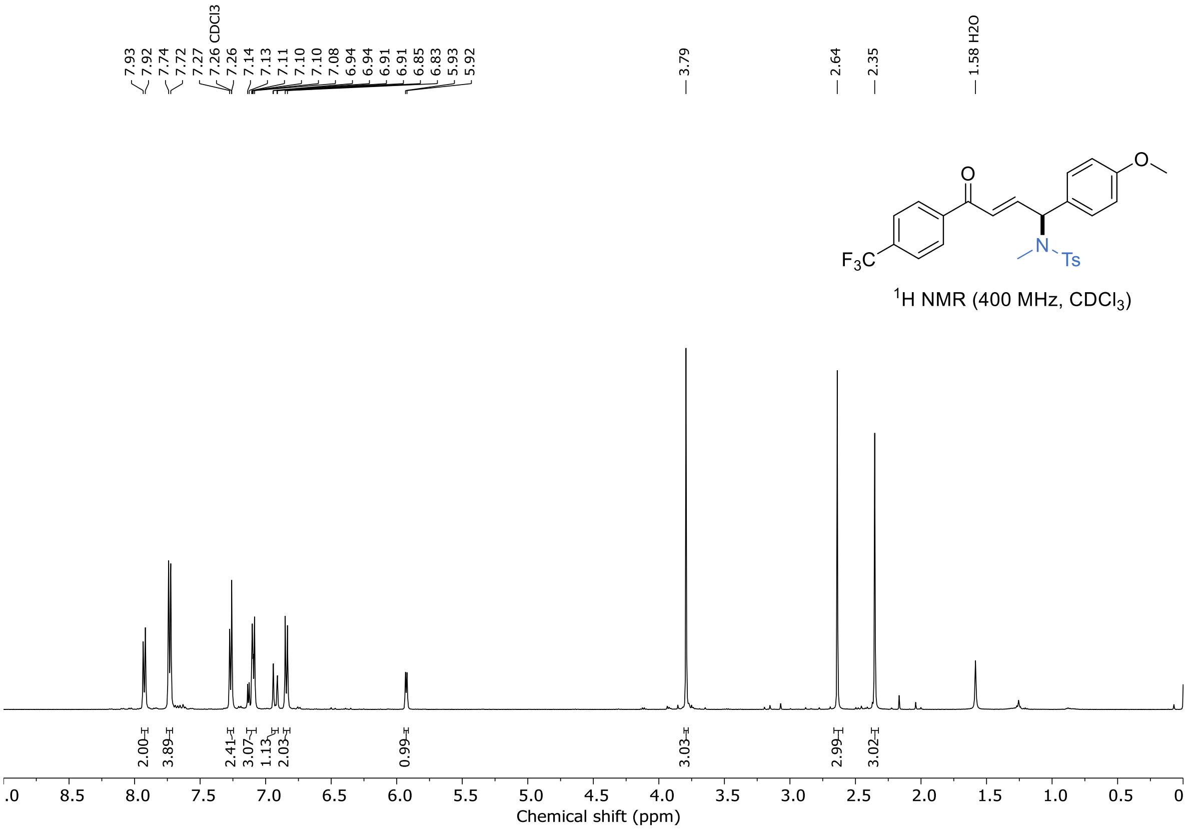

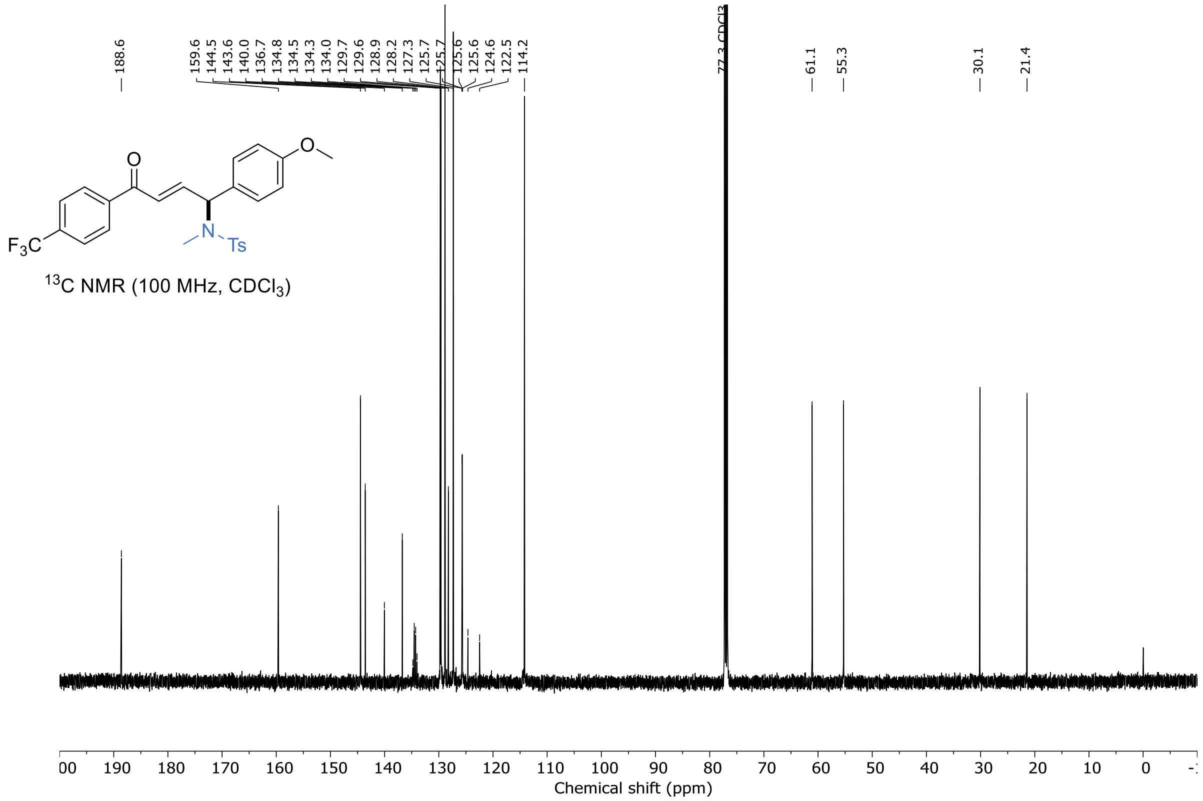

(*E*)-*N*-(1,4-bis(3-methoxyphenyl)-4-oxobut-2-en-1-yl)-*N*,4-dimethylbenzenesulfonamide (13b)

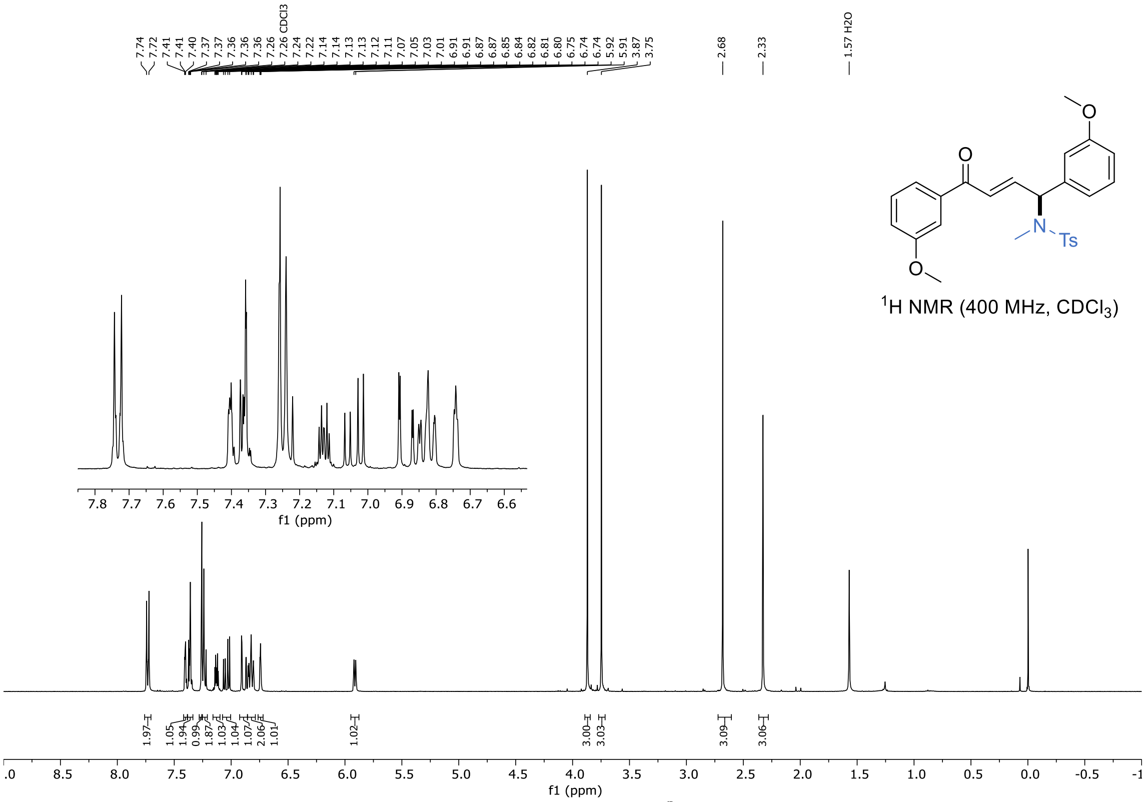

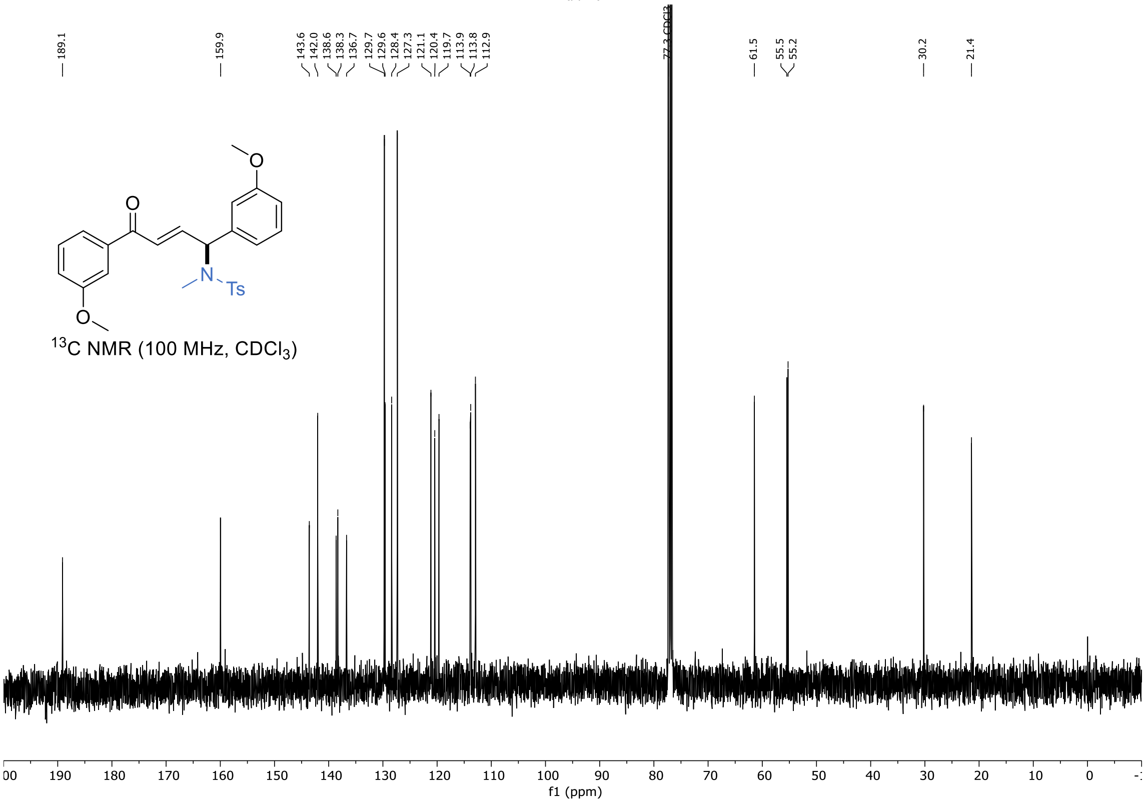

(*Z*)-*N*-(1,4-bis(2-methoxyphenyl)-4-oxobut-2-en-1-yl)-*N*,4-dimethylbenzenesulfonamide (13c)

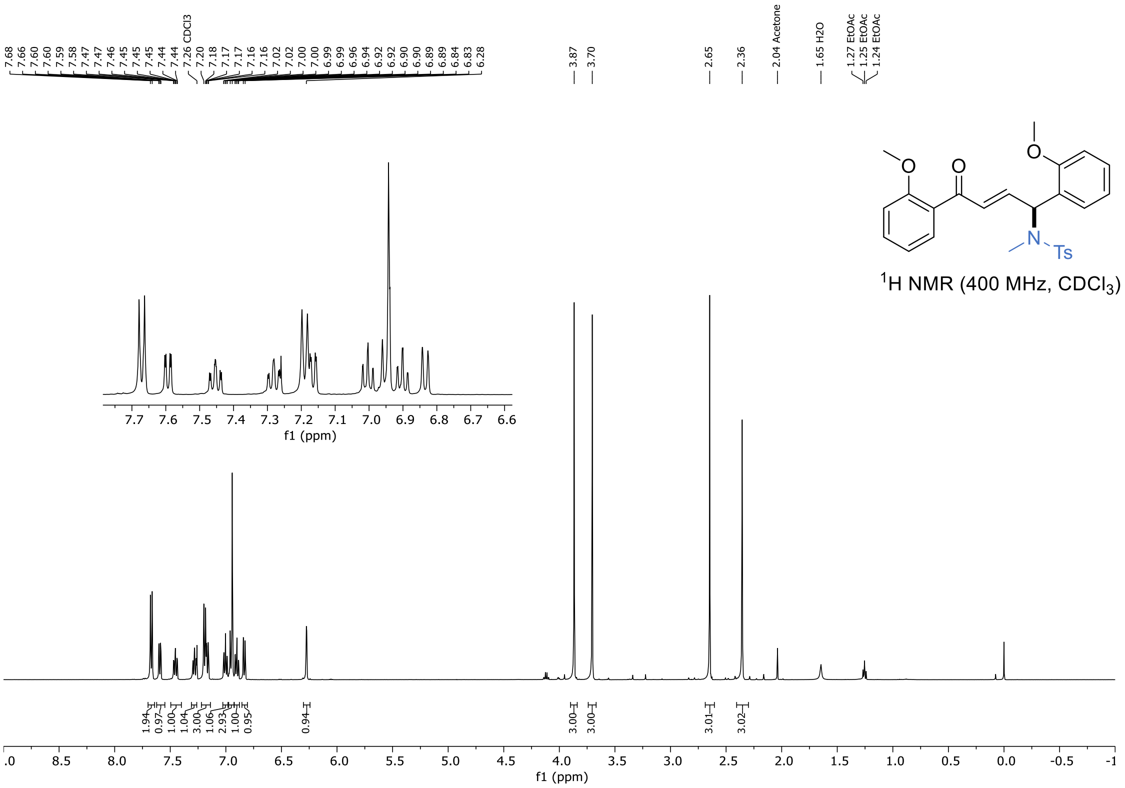

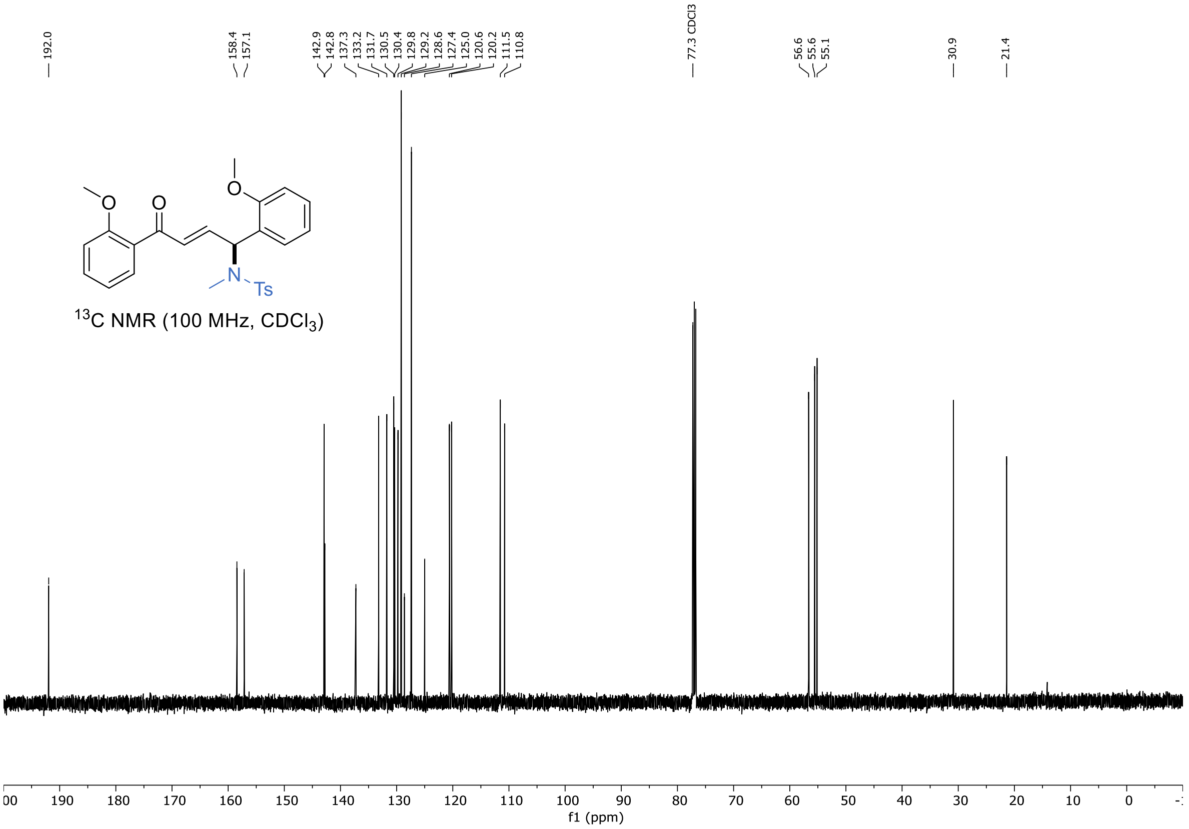

(*Z*)-*N*-(1,4-bis(4-cyanophenyl)-1-oxobut-2-en-2-yl)-*N*,4-dimethylbenzenesulfonamide (14a)

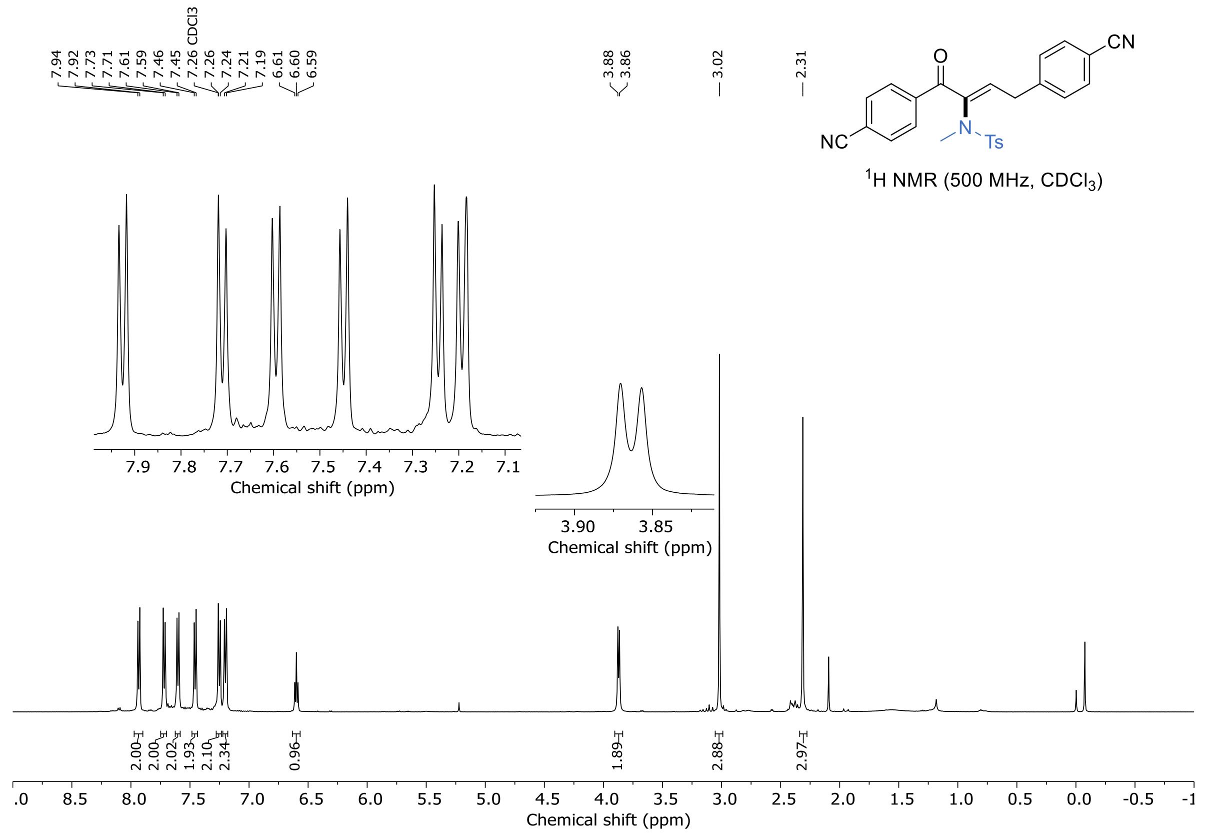

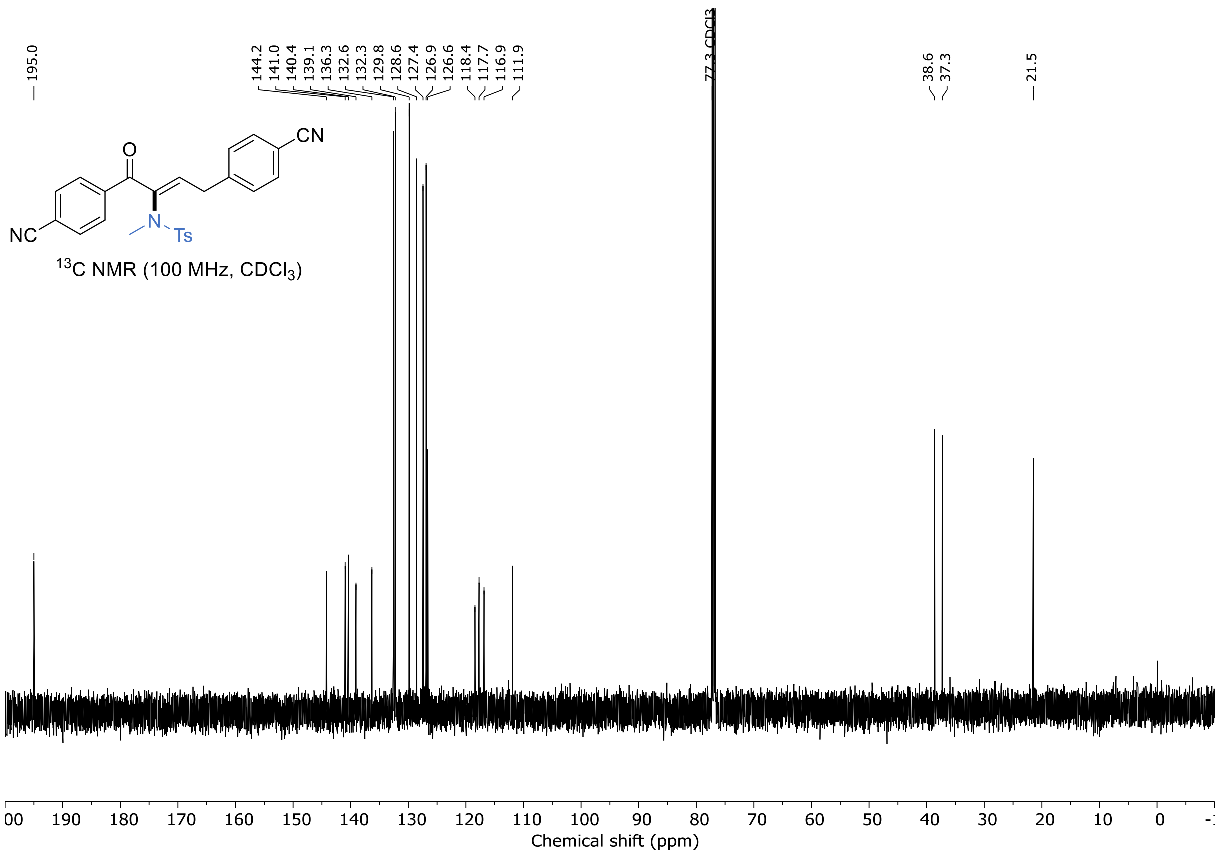

(*E*)-*N*,4-dimethyl-*N*-(4-oxo-1,4-diphenylbut-2-en-1-yl)benzenesulfonamide (12)

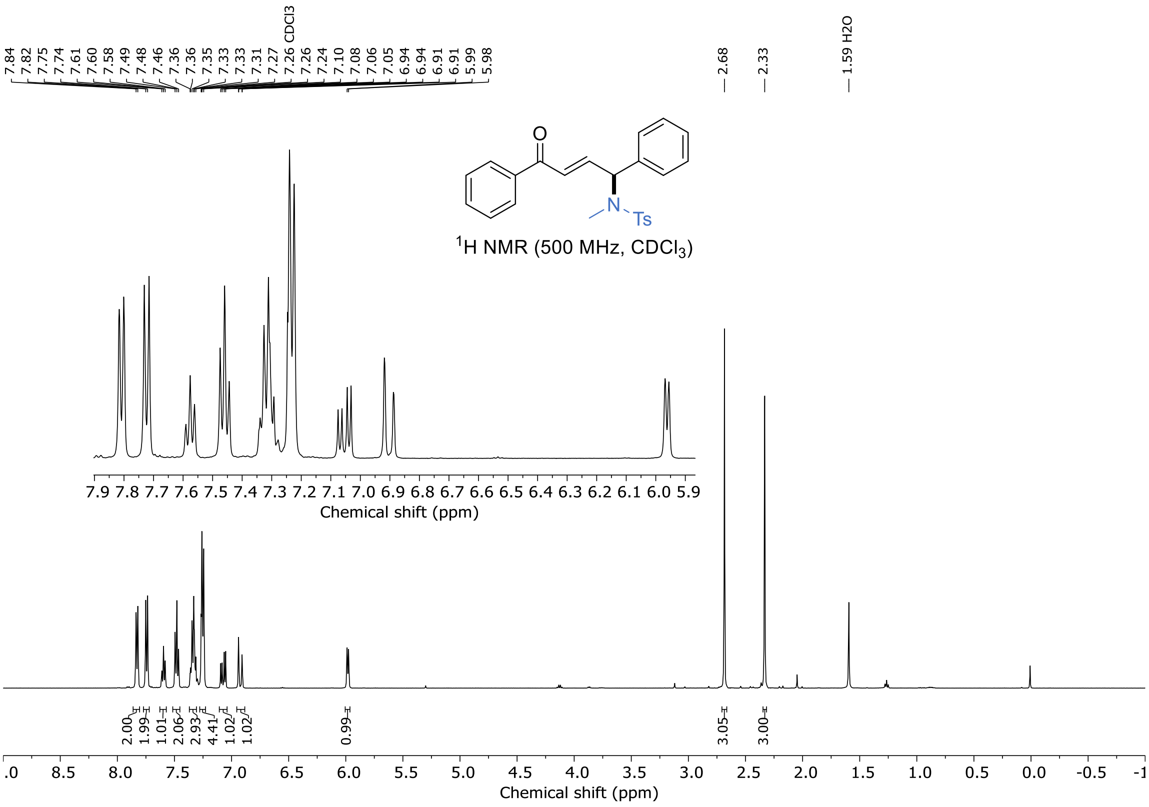

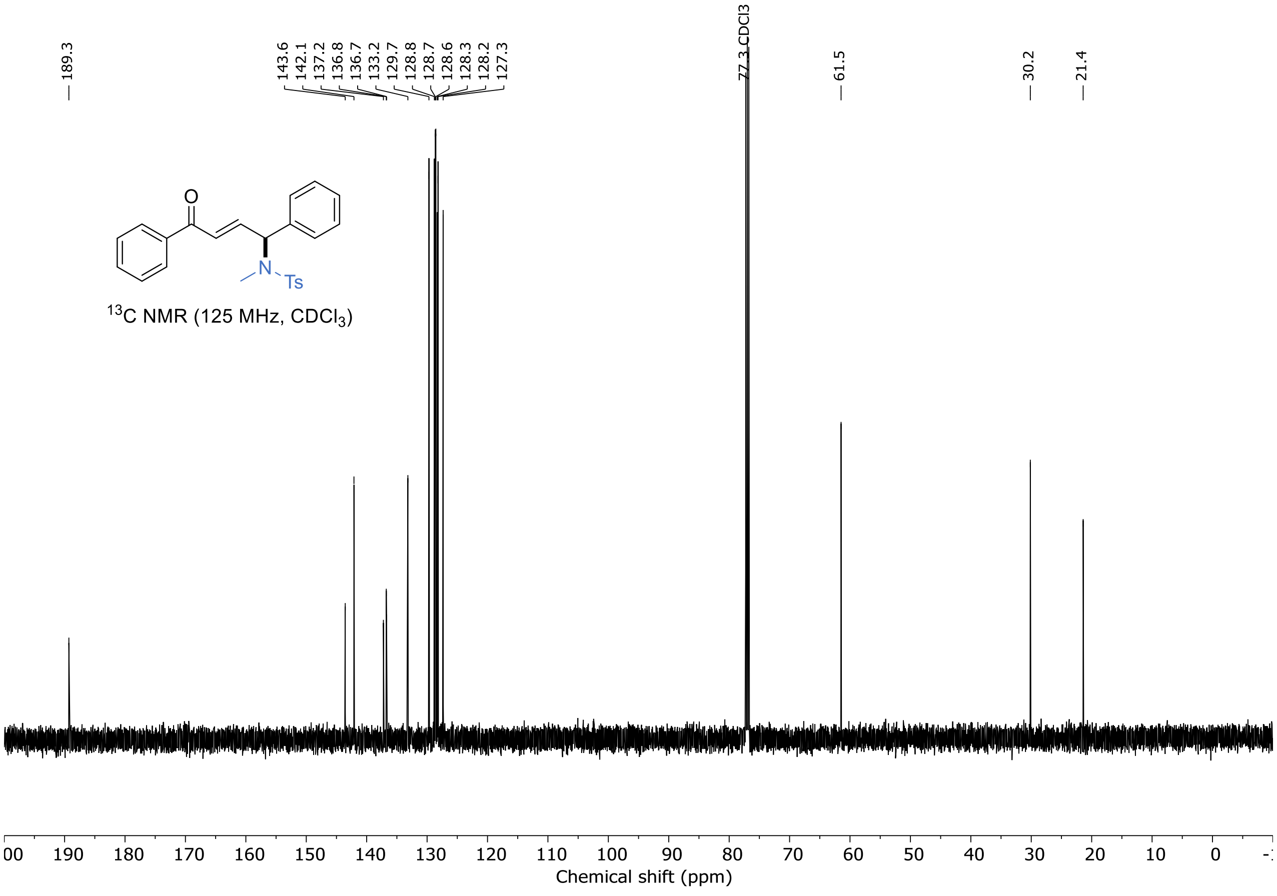

_N_,4-dimethyl-_N_-(4-oxocyclohex-2-en-1-yl)benzenesulfonamide (15)

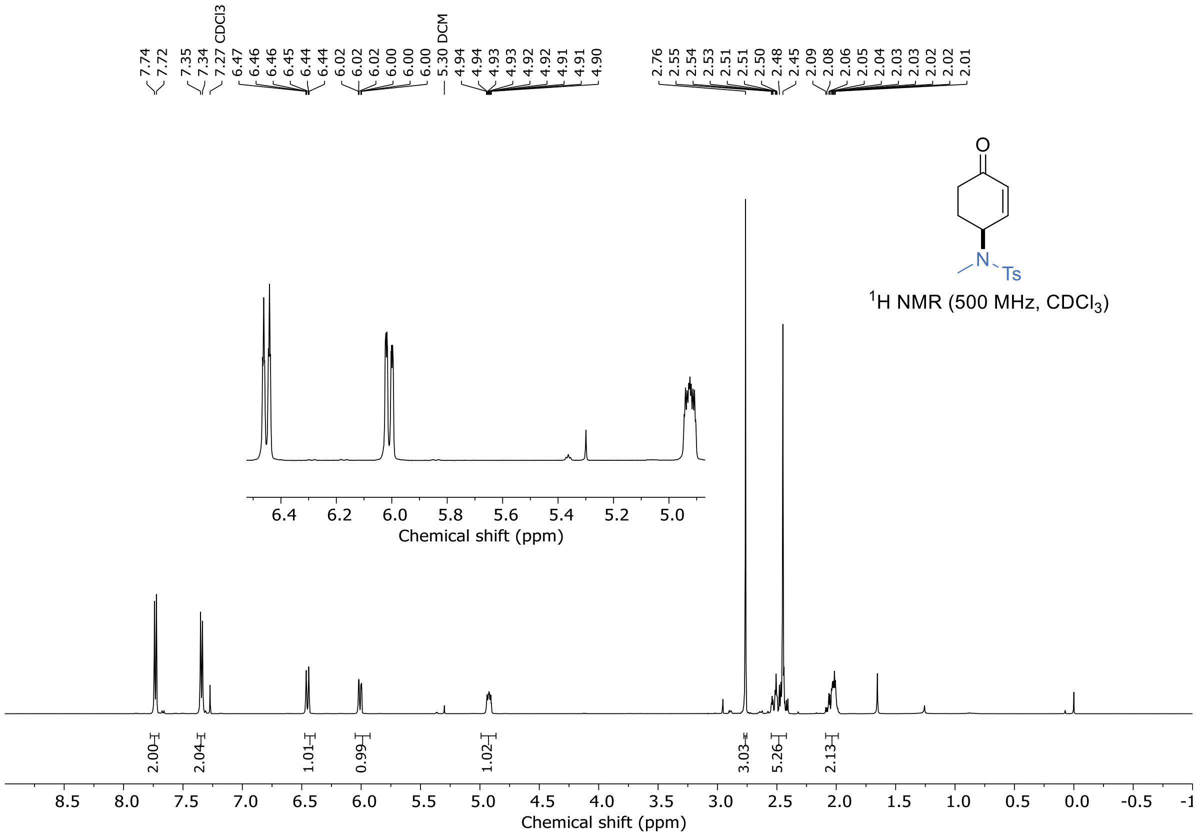

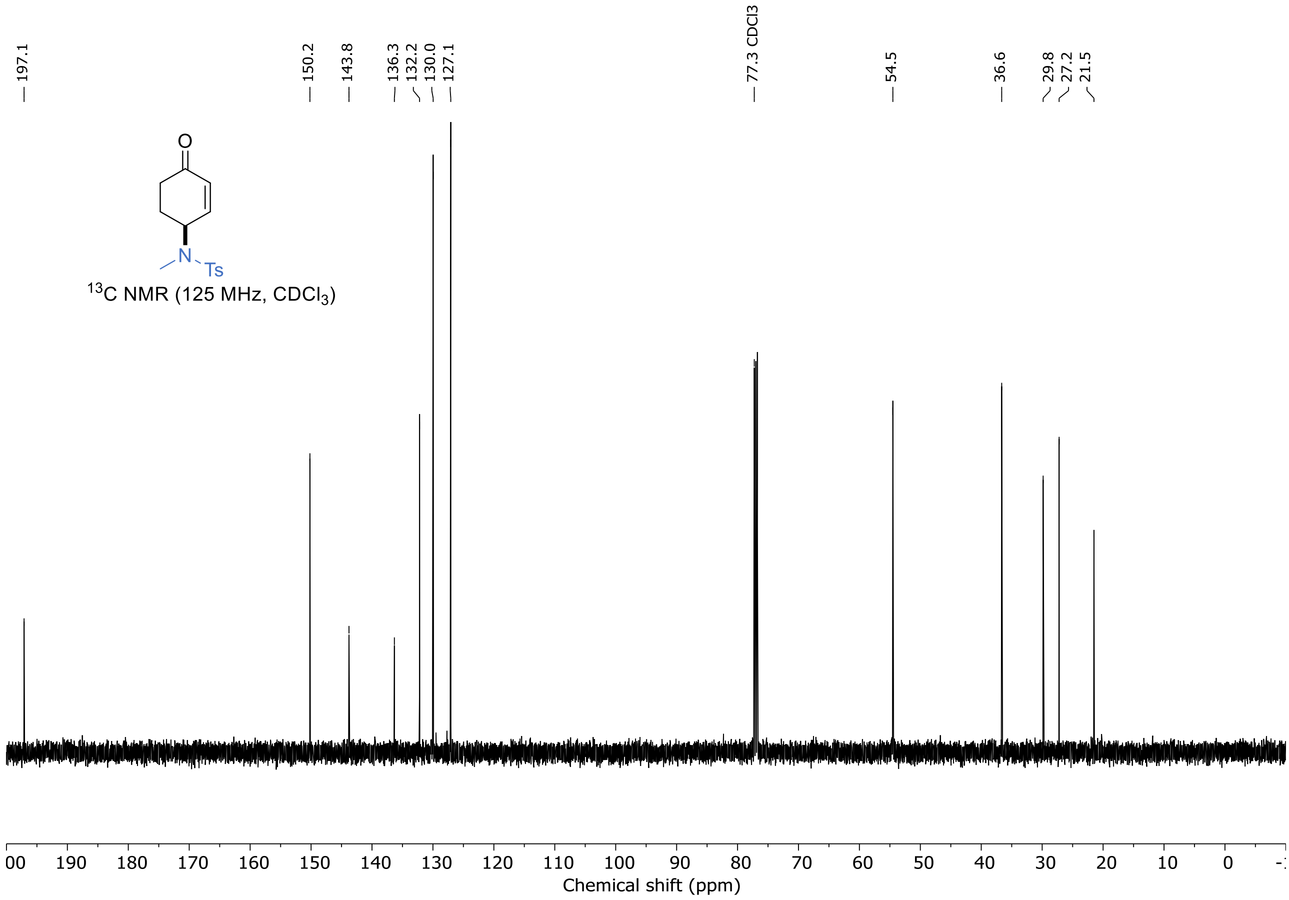

_N_-((5,5-dimethyl-3-oxocyclohex-1-en-1-yl)methyl)-_N_,4-dimethylbenzenesulfonamide (16)

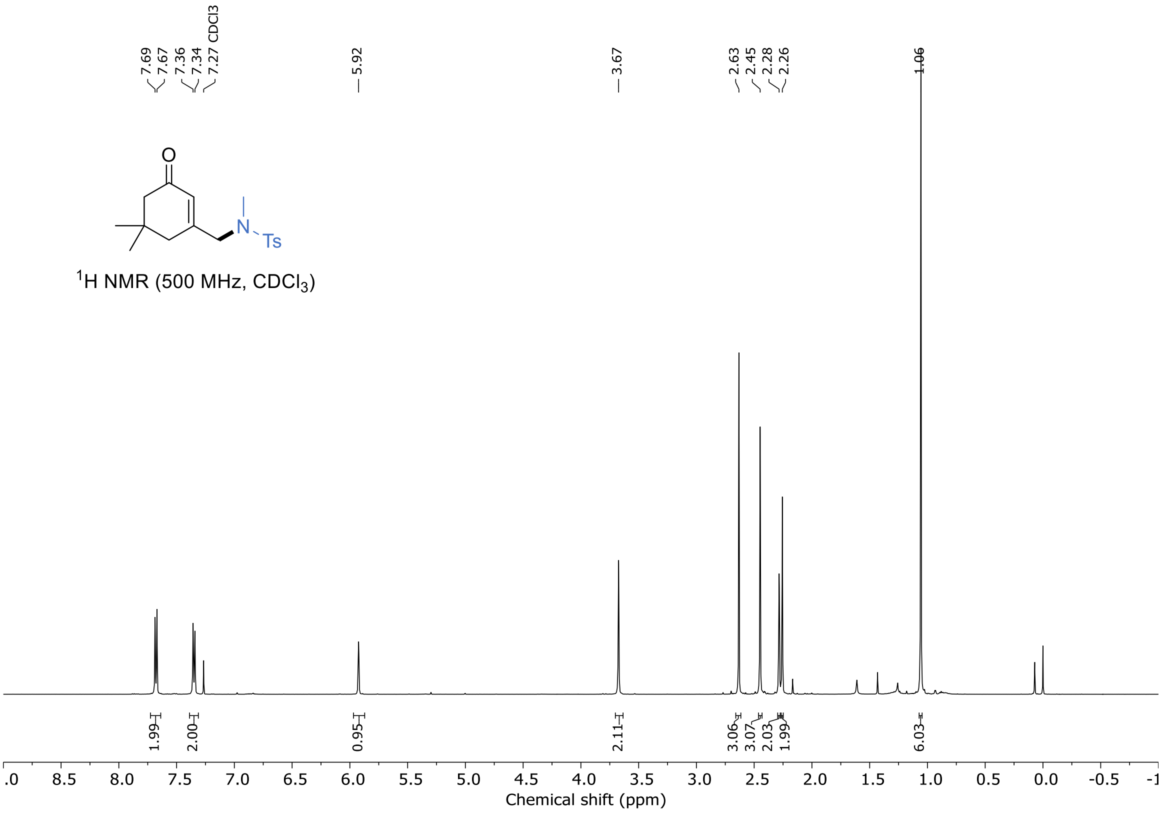

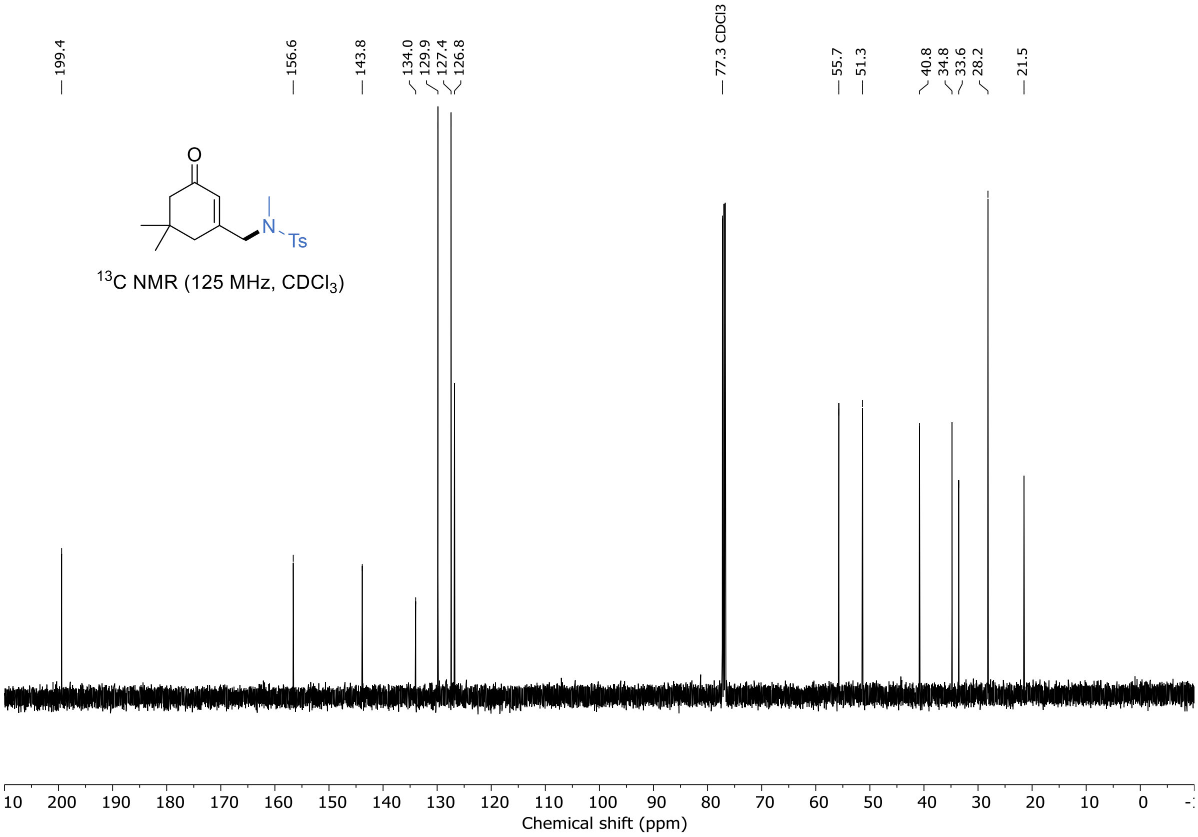

N,4-dimethyl-*N*-(4-oxocyclohept-2-en-1-yl)benzenesulfonamide (17)

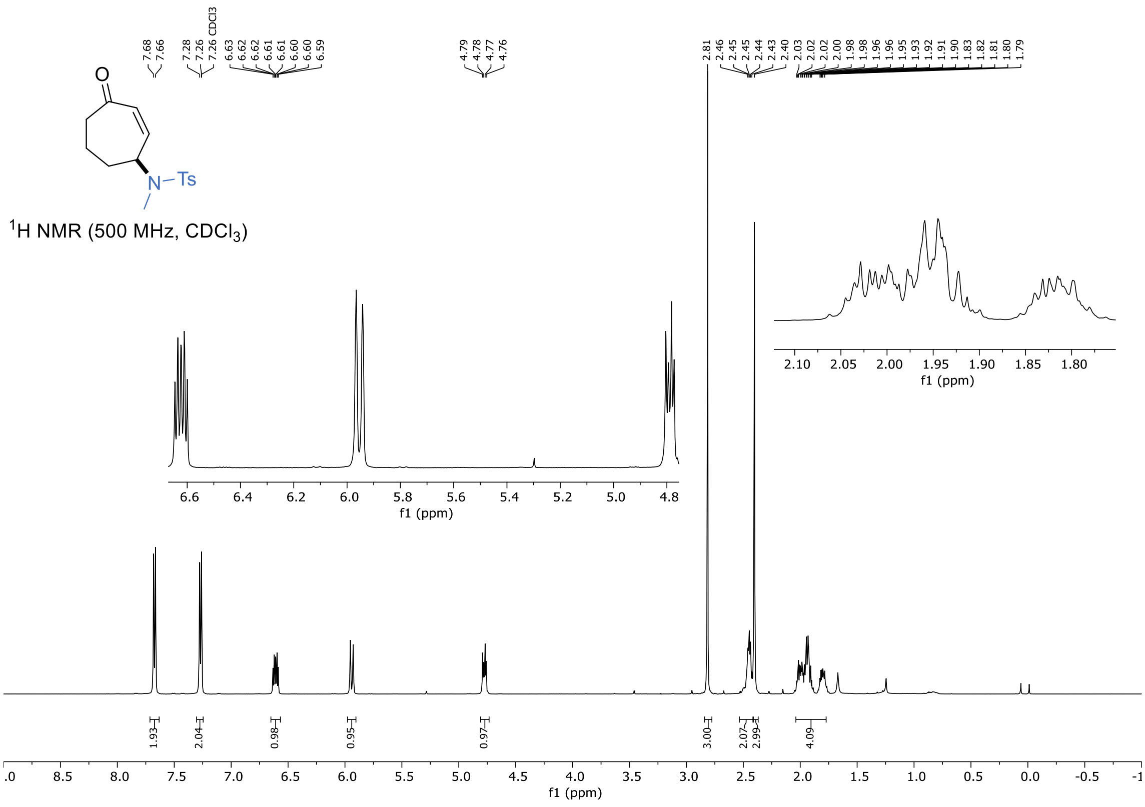

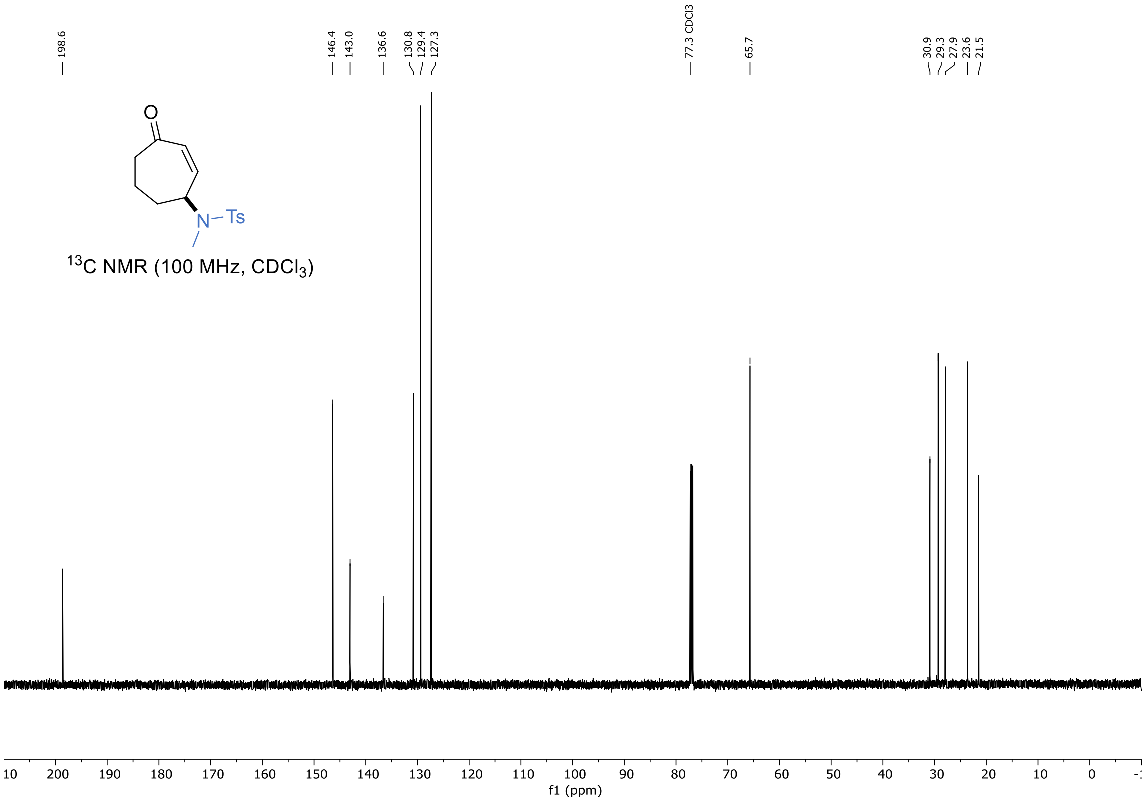

N,4-dimethyl-*N*-(5-oxo-2,5-dihydrofuran-2-yl)benzenesulfonamide (21)

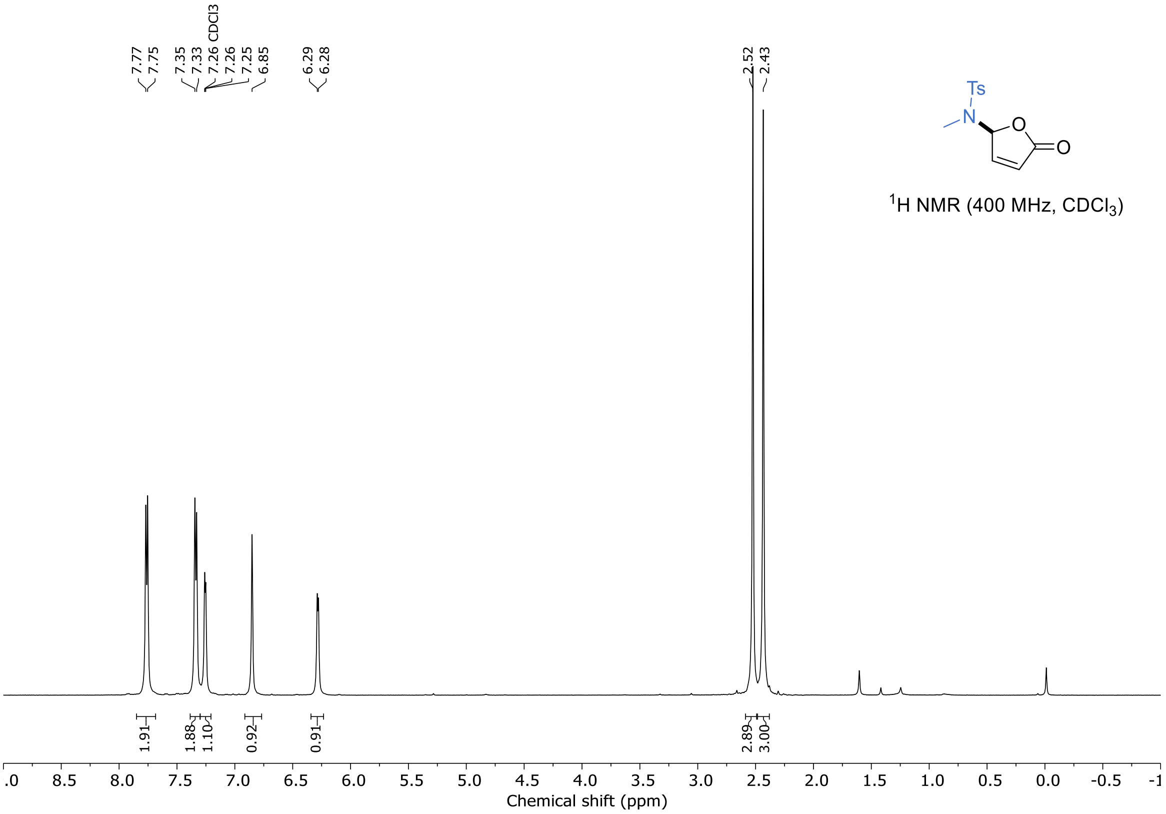

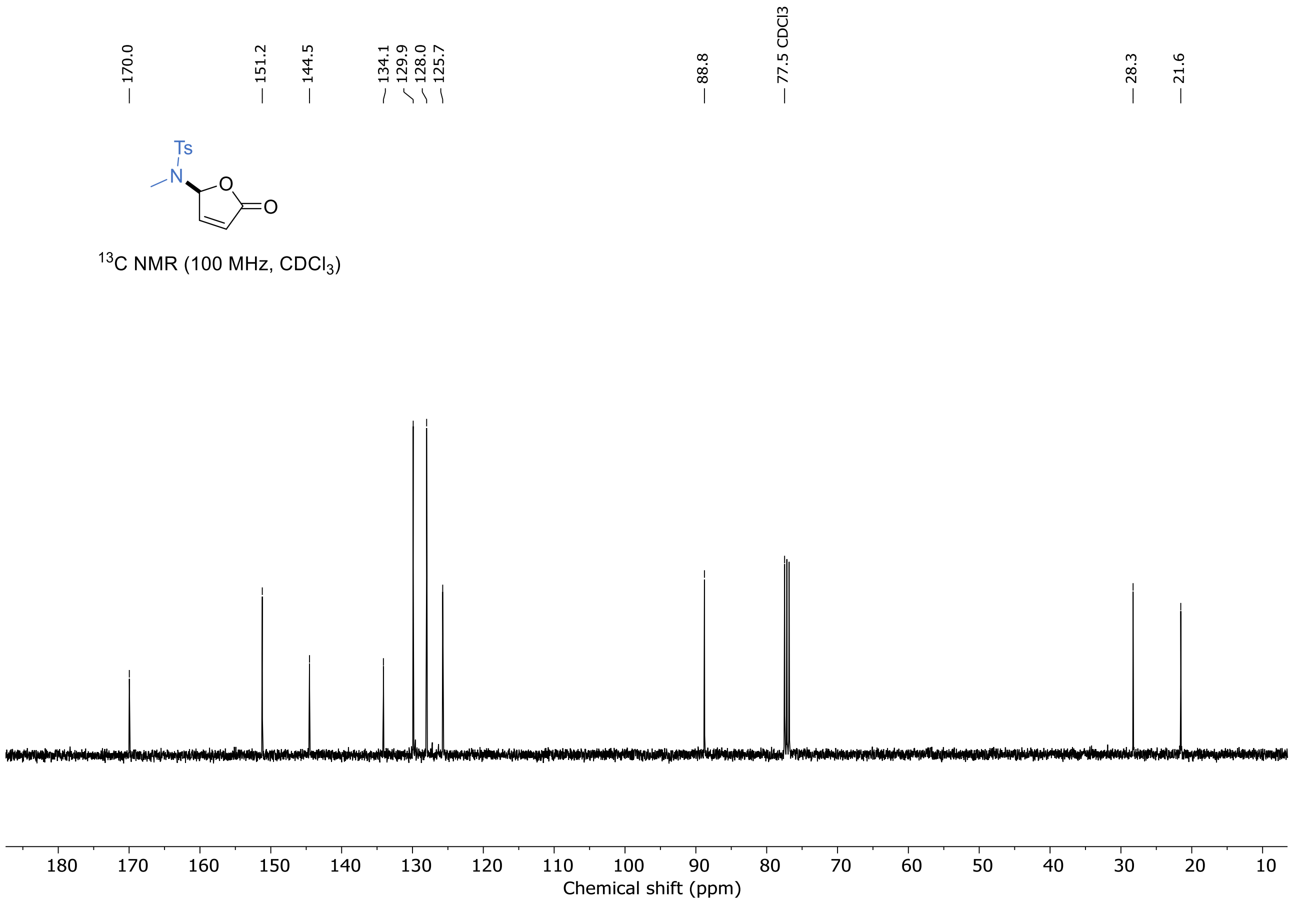

_N_-((3_S_,4a_S_,5_R_)-4a,5-dimethyl-7-oxo-3-(prop-1-en-2-yl)-1,2,3,4,4a,5,6,7-octahydronaphthalen-1-yl)-_N_,4-dimethylbenzenesulfonamide (24)

Major diastereoisomer

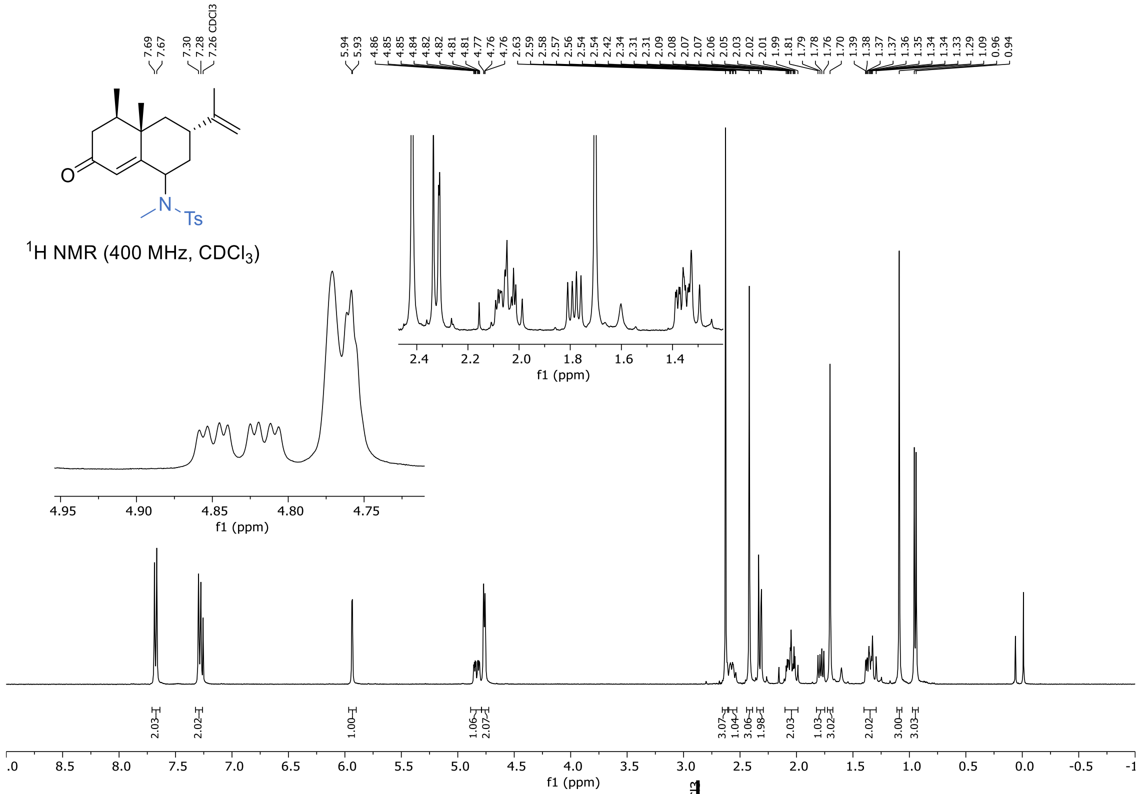

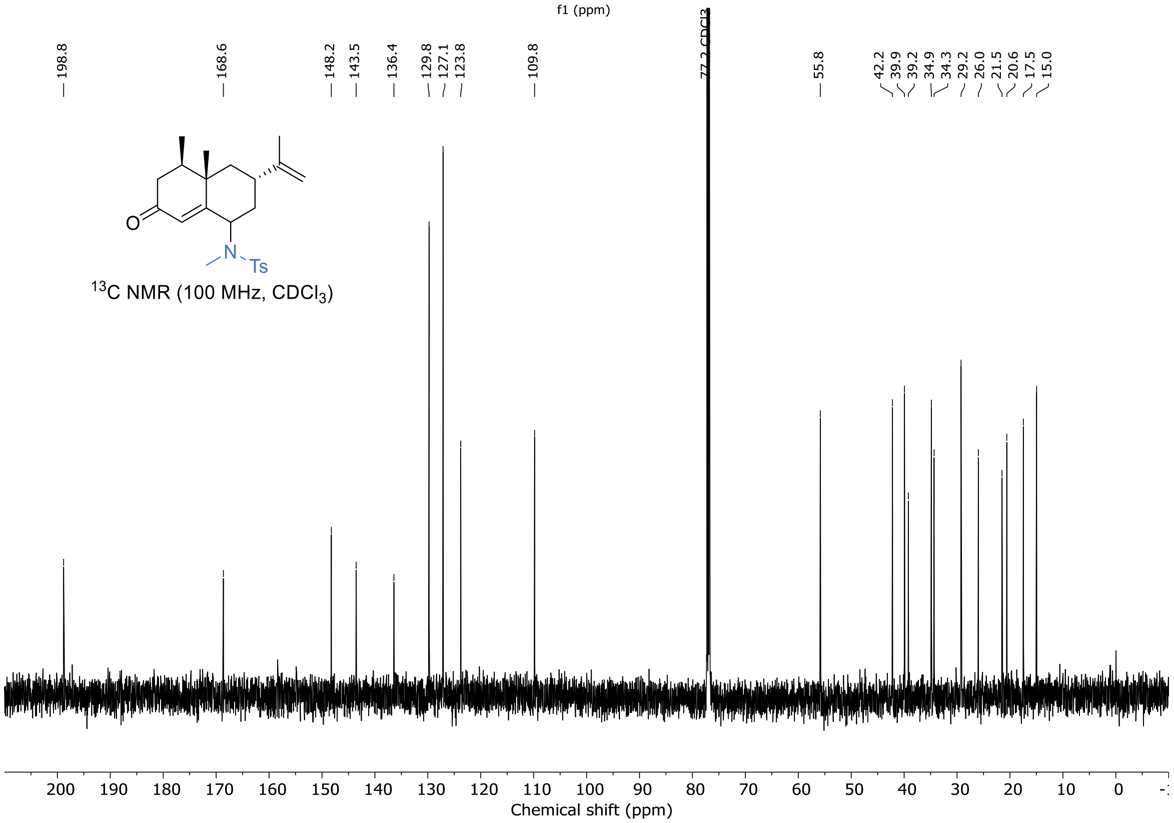

(8*R*,9*S*,10*R*,13*S*,14*S*,17*S*)-6-((*N*,4-dimethylphenyl)sulfonamido)-10,13-dimethyl-3-oxo-2,3,6,7,8,9,10,11,12,13,14,15,16,17-tetradecahydro-1H-cyclopenta[a]phenanthren-17-yl acetate (25)

Major diastereoisomer

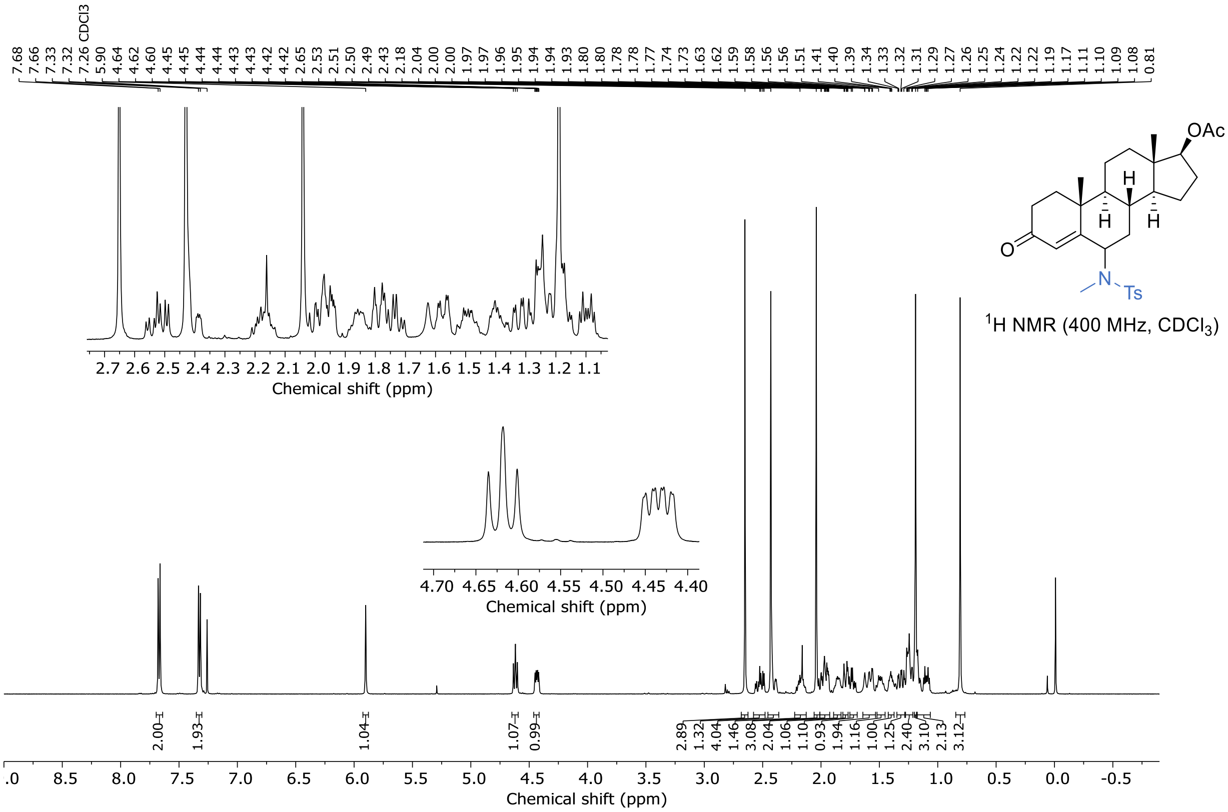

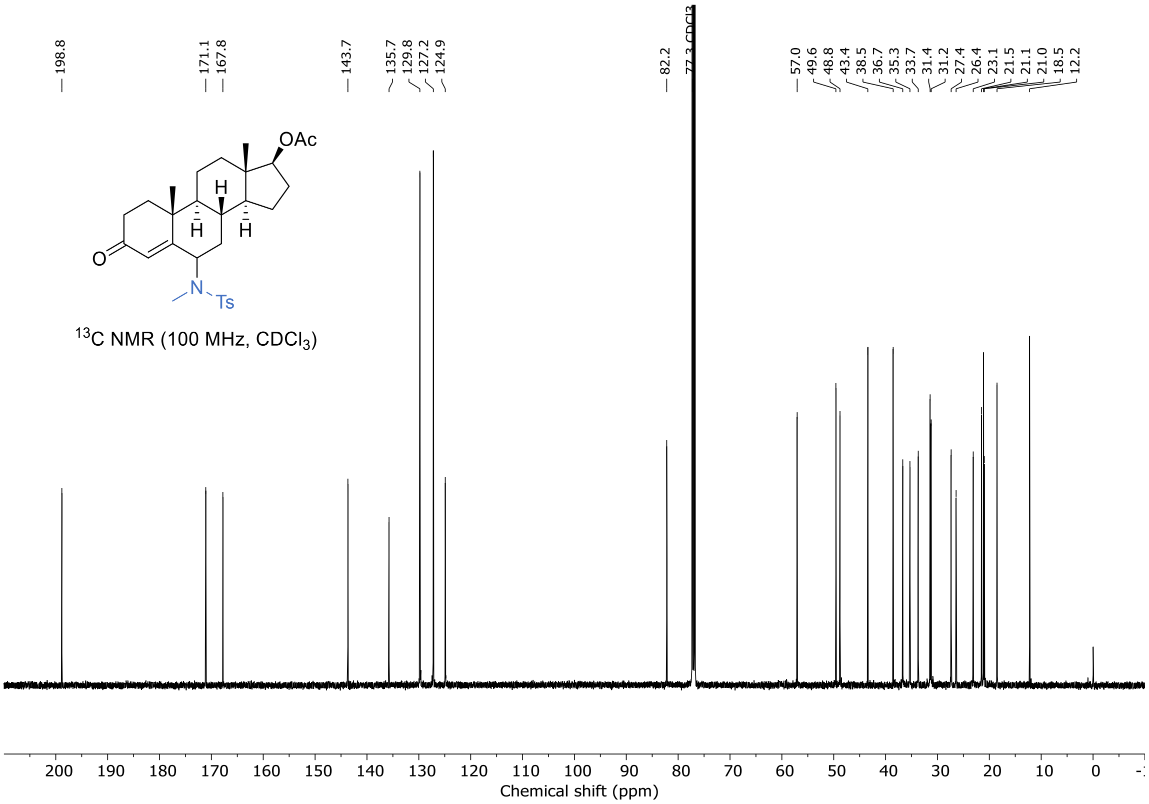

(*E*)-*N*-(7,7-dimethyl-6-oxooct-4-en-3-yl)-*N*,4-dimethylbenzenesulfonamide (18)

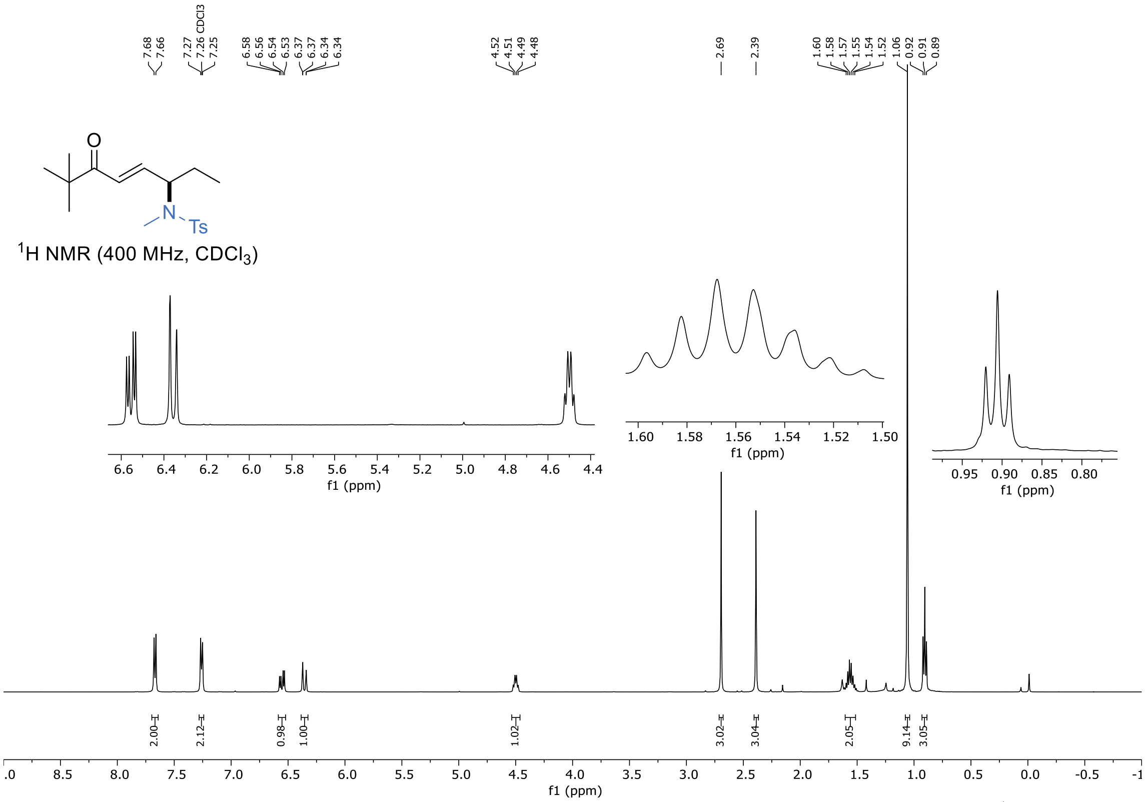

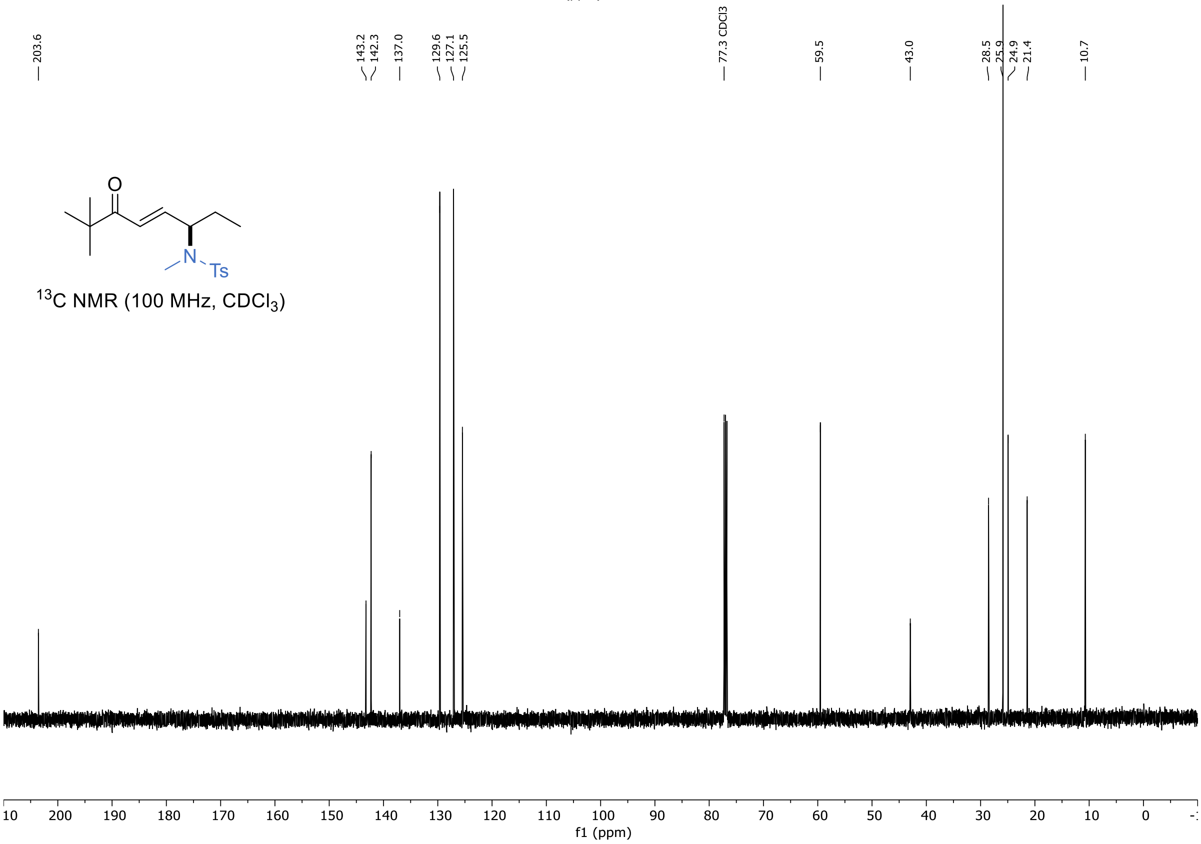

(*E*)-*N*,4-dimethyl-*N*-(6-oxohex-4-en-3-yl)benzenesulfonamide (23)

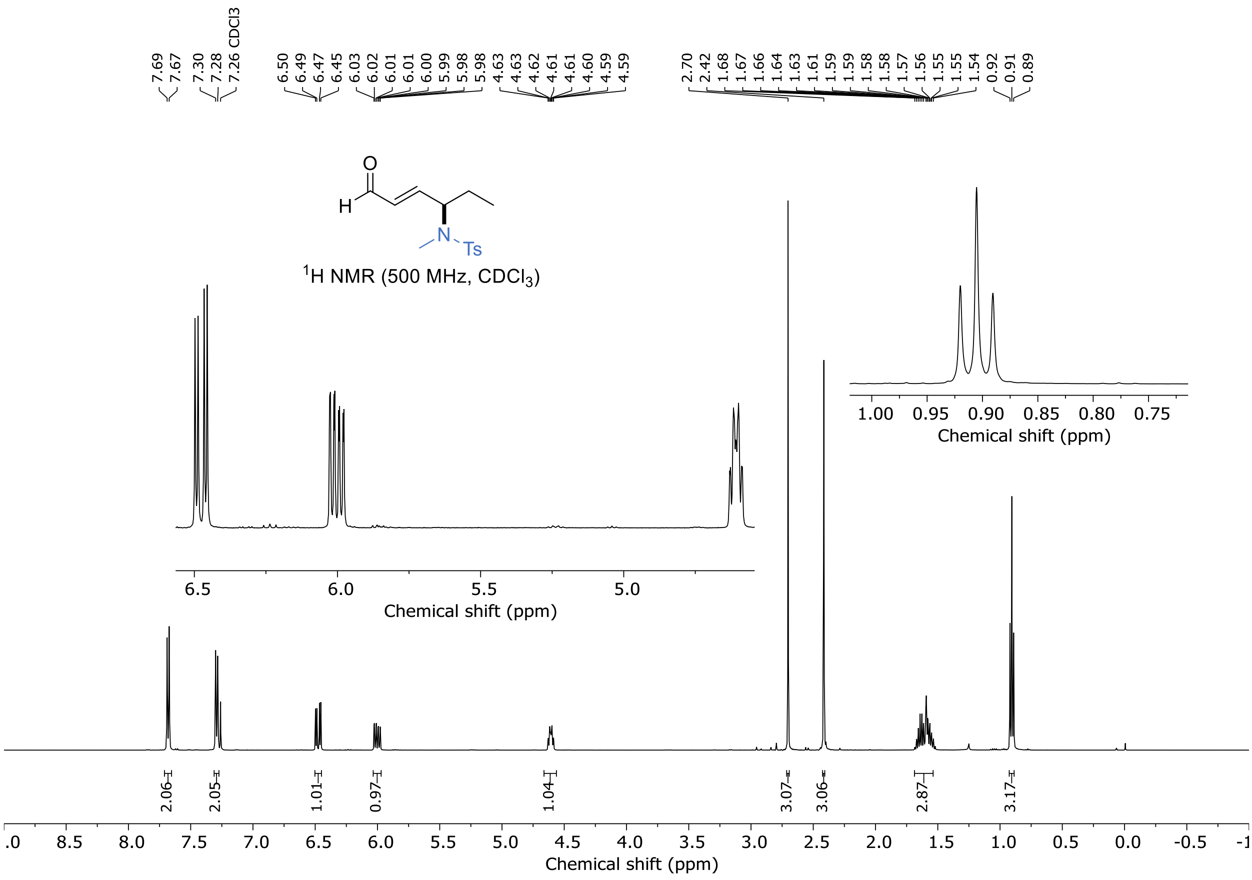

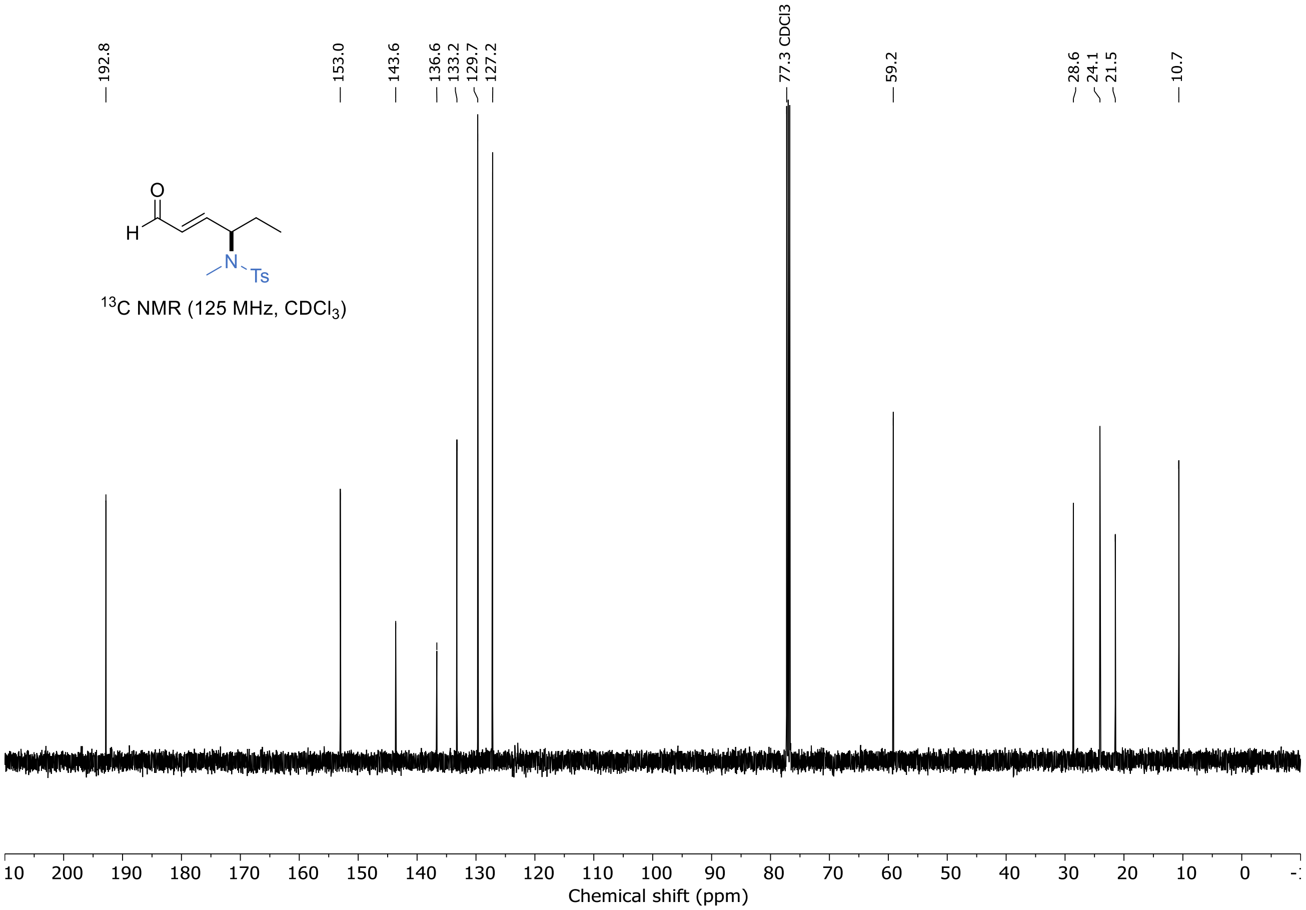

***N*-(3,7-dimethyl-1-oxoocta-2,6-dien-4-yl)-*N*,4-dimethylbenzenesulfonamide (*E*/*Z* mixture) (26)**

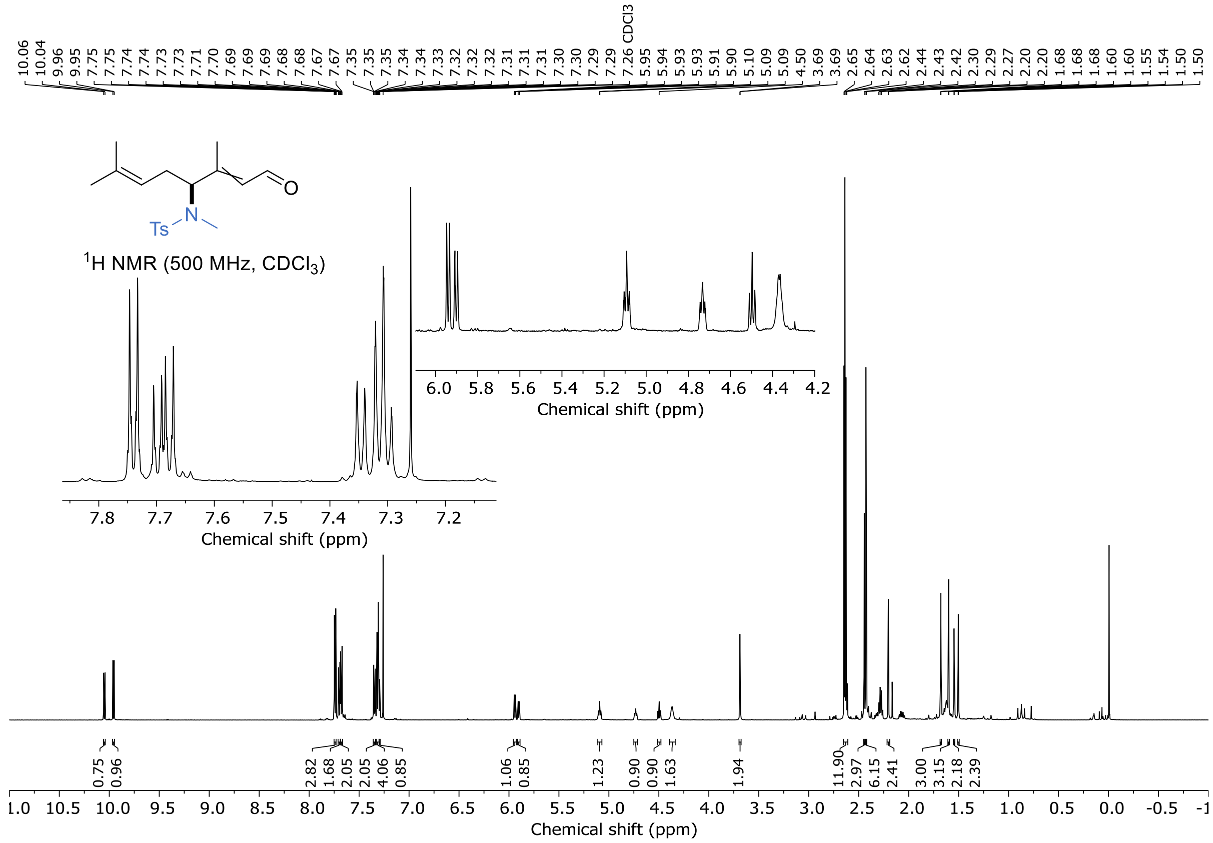

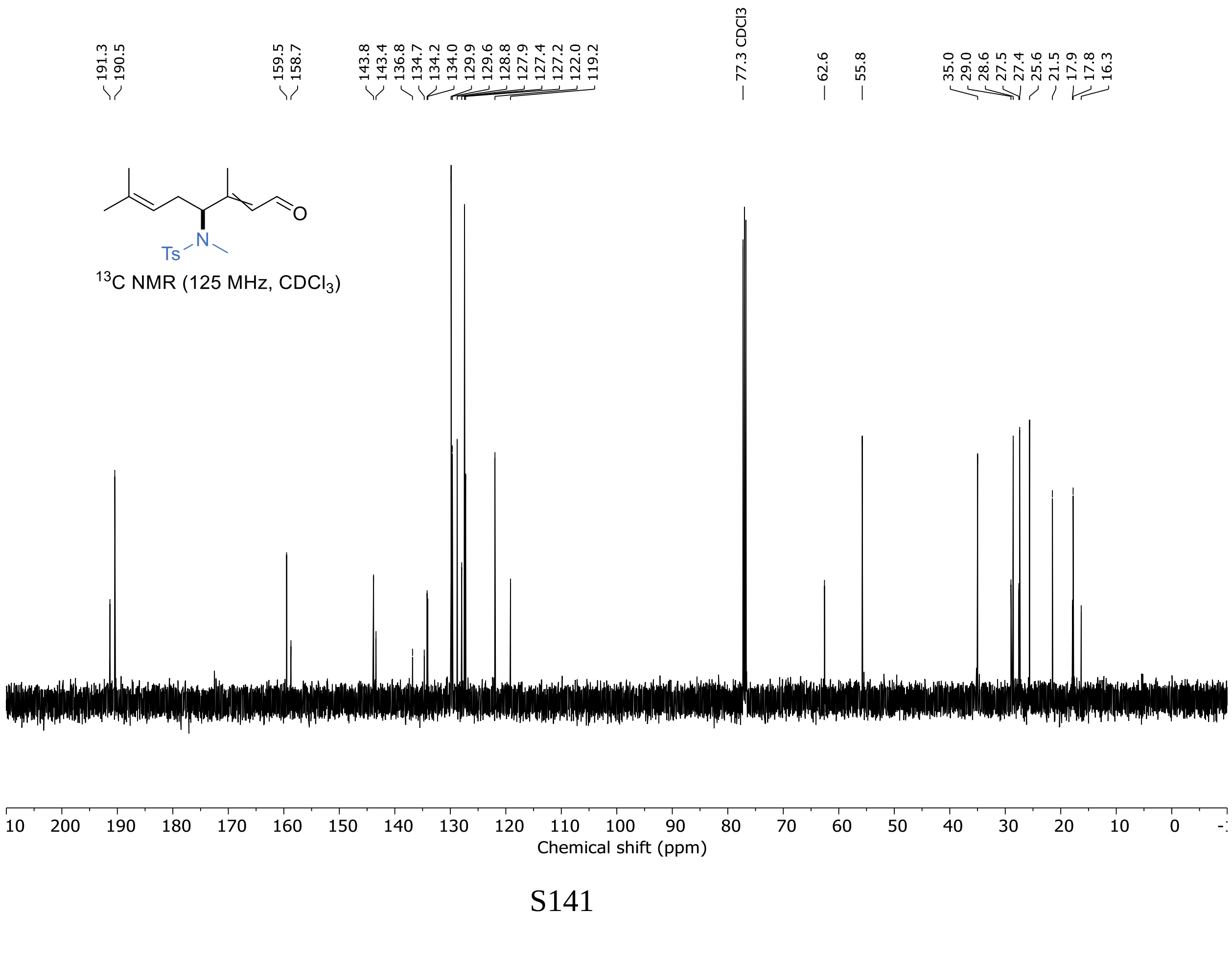

N-(3-formyl-2,4,4-trimethylcyclohex-2-en-1-yl)-*N*,4-dimethylbenzenesulfonamide (27)

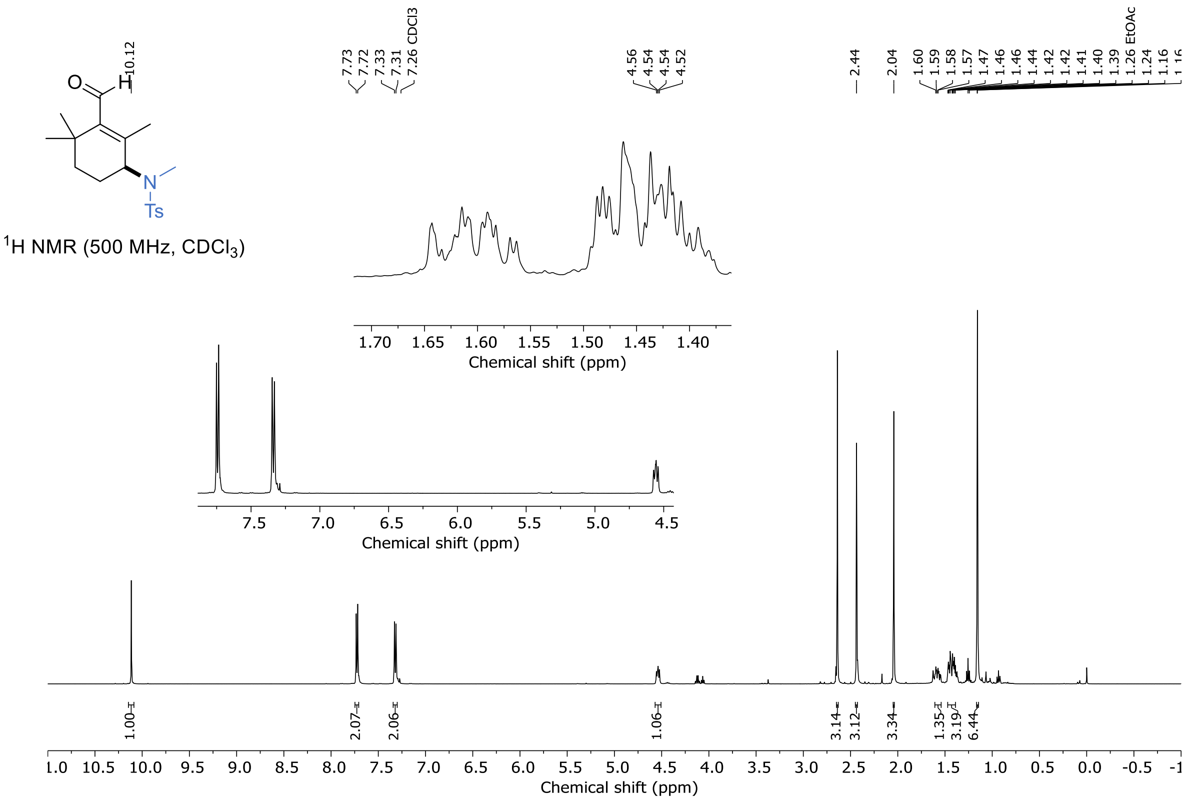

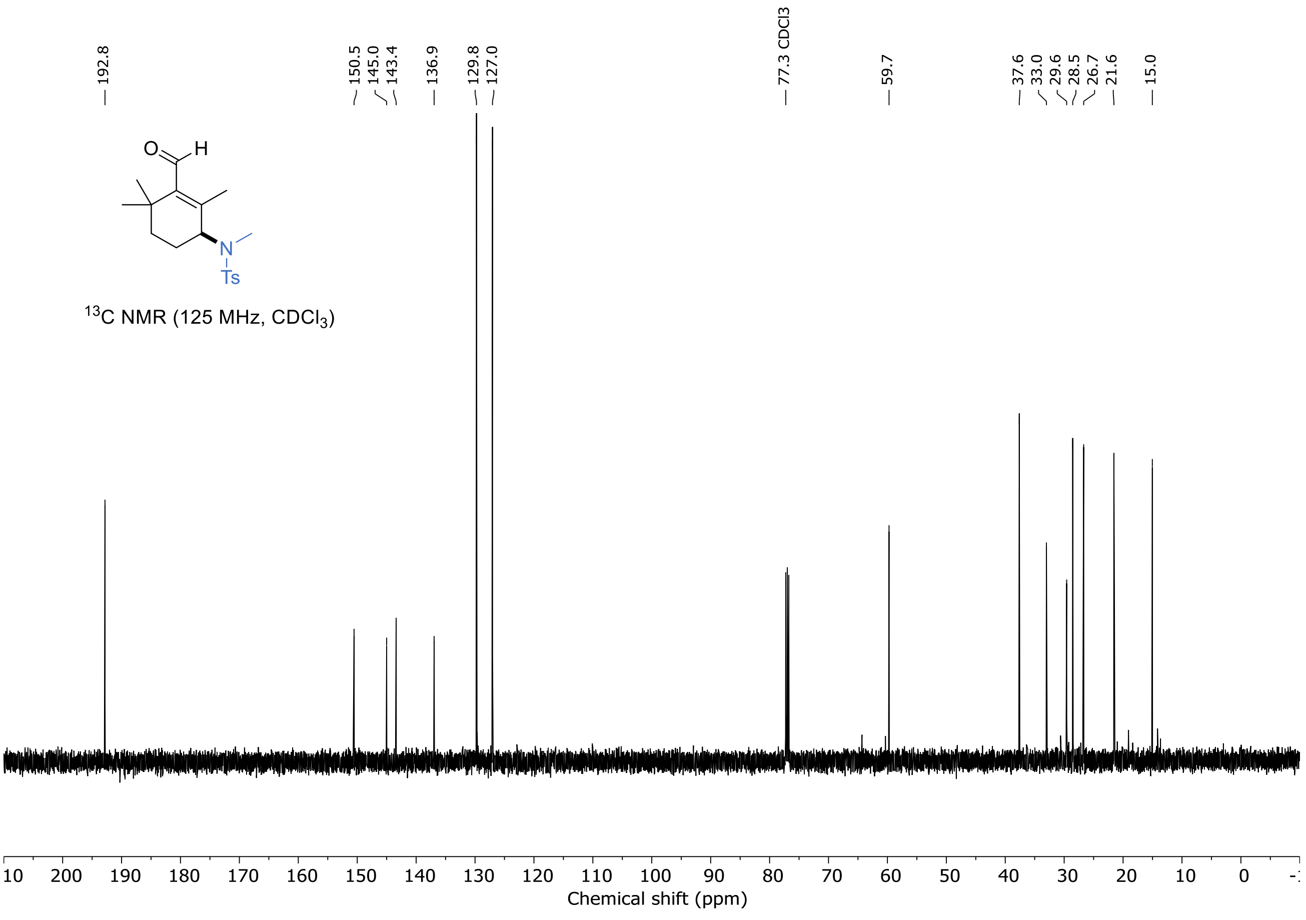

N,4-dimethyl-*N*-(4-oxo-3-phenylbut-2-en-1-yl)benzenesulfonamide (22) + Me-NH-Ts

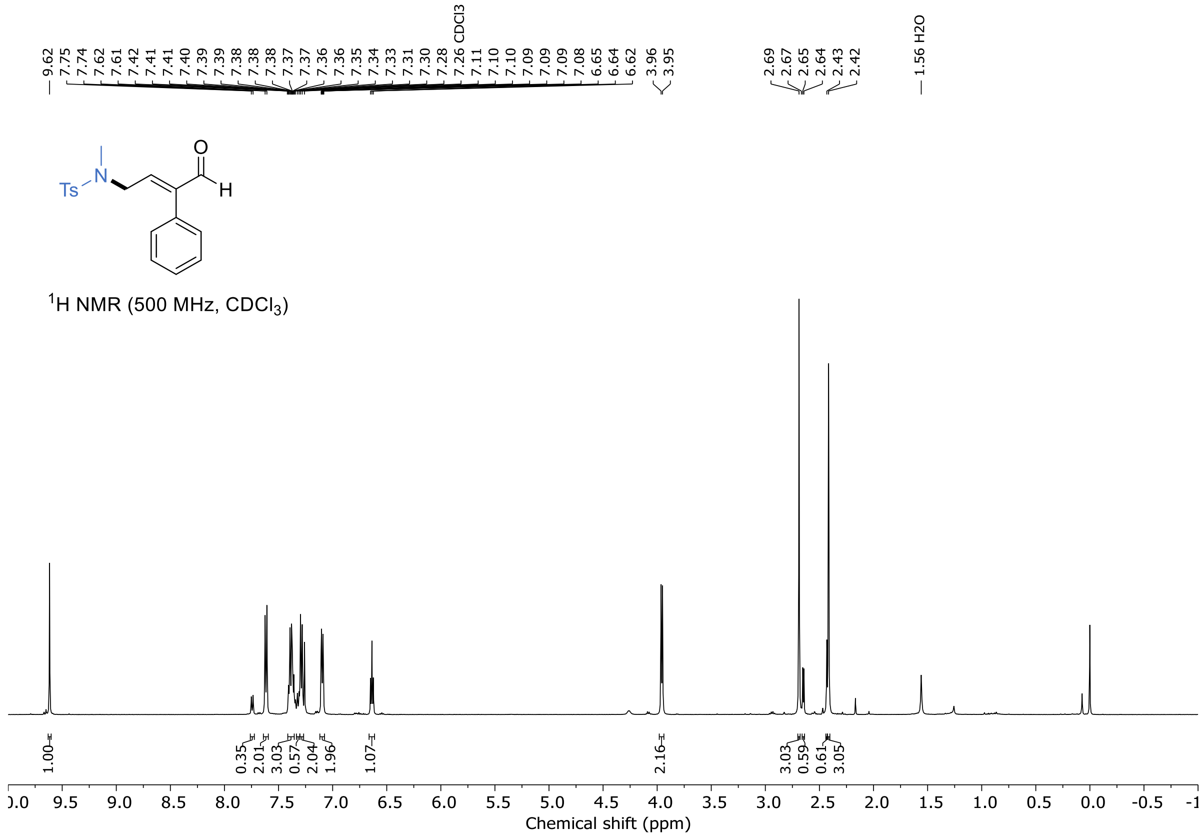

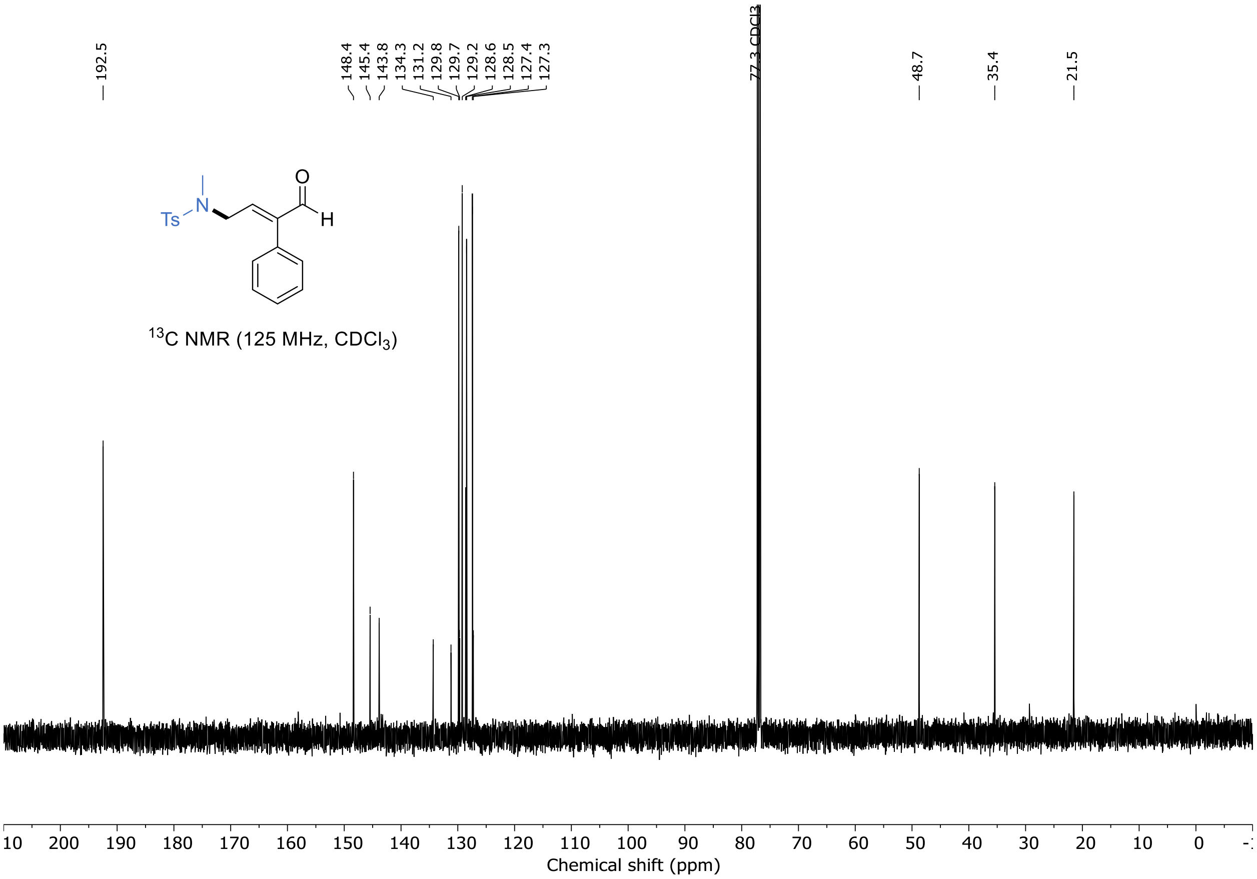

Supplement: Supplementary file 1 — ol2c03161_si_001.pdf [file ol2c03161_si_001.pdf]
